# Supplementary material for: Prevalence of SARS-CoV-2 Variants of Concern and Variants of Interest in COVID-19 Breakthrough Infections in a Hospital in Monterrey, Mexico
Source: Viruses. 2022 Jan 14;14(1):154. doi: 10.3390/v14010154 (PMC8781434; doi:10.3390/v14010154)
Supplement: Supplementary file 1 [file viruses-14-00154-s001.zip › Supplementary Table S2.pdf]

Supplementary Table S2. Clinical characteristics of breakthrough infection cases from this study.

Patient	Group	Age (years)	Sex	Hypertension	Diabetes mellitus type II	Obesity	Smoking	Supplementary oxygen at arrival time	High-flow O_2	Clinical outcome	Vaccine administered	Date of vaccination		Date of onset of symptoms	Percentage of SARS-CoV-2 genome sequenced	Pango linage	Variant
												1st dose	2nd dose				
34797	H	74	F	No	No	No	No	Yes	Yes	Death	Pfizer/ BioNTech	Apr-21	No	4/19/2021	99.6	B.1	
35194	H	68	F	Yes	No	No	No	No	No	Cure	Unspecified	Apr-21	No	4/24/2021	99.6	B.1.1.519	
37613	H	70	M	Yes	Yes	No	No	Yes	Yes	Cure	Sinovac	Unspecified	Apr-21	5/31/2021	92.1	P.1	Gamma
39540	H	69	M	Yes	No	No	Yes	No	Yes	Death	Astra Zeneca/ Oxford	Mar-21	May-21	6/28/2021	99.6	B.1.617.2	Delta
39690	H	54	F	No	No	No	No	Yes	Yes	Cure	Unspecified	May-21	No	6/30/2021	99.6	B.1.628	
40042	H	50	M	Yes	No	No	Yes	No	No	Death	Sinovac	Jun-21	No	7/5/2021	97.8	B.1.621	Mu
40162	H	71	M	Yes	Yes	No	Yes	Yes	Yes	Cure	Sinovac	Apr-21	Apr-21	7/6/2021	98.7	B.1.632	
40363	H	52	M	No	Yes	No	No	Yes	Yes	Cure	CanSino	May-21	NA	7/9/2021	98.7	B.1.621	Mu
40373	H	49	F	No	No	No	No	Yes	Yes	Cure	CanSino	Apr-21	NA	7/9/2021	96.8	AY.3	Delta
40507	H	64	M	Yes	Yes	No	No	Yes	Yes	Cure	Sinovac	Unspecified	Jun-21	7/10/2021	83.9	B.1.1.7	Alpha
40635	H	69	F	No	No	No	No	Yes	Yes	Cure	Pfizer/ BioNTech	Unspecified	Mar-21	7/12/2021	98.7	AY.4	Delta
41494	H	43	M	No	No	No	No	Yes	No	Cure	CanSino	Apr-21	NA	7/20/2021	90.6	AY.3	Delta
41638	H	60	M	Yes	Yes	No	No	Yes	Yes	Cure	Astra Zeneca/ Oxford	Apr-21	May-21	7/21/2021	98.7	AY.11	Delta
41779	H	75	F	Yes	Yes	No	No	Yes	No	Cure	Pfizer/ BioNTech	Jan-21	Jan-21	7/21/2021	98.7	AY.4	Delta
42266	H	70	F	Yes	Yes	No	No	Yes	Yes	Cure	Astra Zeneca/ Oxford	Jun-21	Jun-21	7/26/2021	98.7	B.1.617.2	Delta
42271	H	58	M	No	Yes	No	No	Yes	Yes	Cure	Astra Zeneca/ Oxford	May-21	No	7/26/2021	98.7	AY.3	Delta
42690	H	50	M	No	No	Yes	Yes	Yes	Yes	Cure	CanSino	Apr-21	NA	7/29/2021	98.7	AY.11	Delta
43093	H	72	F	Yes	Yes	No	No	No	No	Cure	Pfizer/ BioNTech	Unspecified	Feb-21	8/1/2021	99.6	P.1	Gamma
43367	H	65	M	No	No	No	No	No	Yes	Cure	Sinovac	Unspecified	Jun-21	8/3/2021	98.7	AY.11	Delta
43383	H	44	F	No	Yes	No	No	Yes	No	Cure	Unspecified	Unspecified	Jul-21	8/3/2021	98.7	AY.11	Delta
43405	H	40	M	No	Yes	No	No	No	No	Cure	Pfizer/ BioNTech	Unspecified	Aug-21	8/3/2021	98.7	AY.11	Delta
43678	H	54	F	No	Yes	No	No	No	No	Cure	Astra Zeneca/ Oxford	Jun-21	No	8/5/2021	98.7	AY.4	Delta
43680	H	52	F	Yes	Yes	No	No	No	No	Death	Sinovac	Unspecified	Unspecified	8/5/2021	98.7	AY.4	Delta
40278	A	42	F	No	No	No	No	NA	NA	NA	CanSino	Apr-21	NA	7/8/2021	90.3	B.1.617.2	Delta
40281	A	71	M	No	No	No	No	NA	NA	NA	Astra Zeneca/ Oxford	Mar-21	Apr-21	7/8/2021	99.6	B.1.621	Mu
40282	A	31	M	No	No	No	No	NA	NA	NA	CanSino	May-21	NA	7/8/2021	99.6	B.1.617.2	Delta
40287	A	37	F	No	No	No	No	NA	NA	NA	CanSino	Apr-21	NA	7/8/2021	98.6	B.1.1.7	Alpha
40292	A	52	F	No	No	Yes	No	NA	NA	NA	CanSino	Apr-21	NA	7/8/2021	99.6	P.1	Gamma
40367	A	51	F	No	No	No	No	NA	NA	NA	CanSino	Mar-21	NA	7/9/2021	98.7	AY.4	Delta
40394	A	24	F	No	No	No	No	NA	NA	NA	CanSino	Apr-21	NA	7/9/2021	90.2	B.1.617.2	Delta

40515	A	28	F	No	No	No	No	NA	NA	NA	CanSino	May-21	NA	7/10/2021	79.2	B.1.1.7	Alpha
40518	A	29	M	No	No	No	Yes	NA	NA	NA	Pfizer/ BioNTech	Feb-21	Mar-21	7/10/2021	91.7	AY.3	Delta
40522	A	53	F	Yes	Yes	No	No	NA	NA	NA	Sinovac	Apr-21	May-21	7/10/2021	86.7	P.1.1	Gamma
40523	A	71	F	Yes	No	No	No	NA	NA	NA	Astra Zeneca/ Oxford	Apr-21	May-21	7/10/2021	96.7	B.1.617.2	Delta
40580	A	43	F	Yes	No	No	No	NA	NA	NA	CanSino	Apr-21	NA	7/11/2021	98.7	B.1.617.2	Delta
40585	A	56	F	No	Yes	No	No	NA	NA	NA	Astra Zeneca/ Oxford	Jun-21	No	7/11/2021	98.7	B.1.617.2	Delta
40614	A	57	F	No	No	No	No	NA	NA	NA	Sinovac	May-21	Jun-21	7/12/2021	98.7	B.1.617.2	Delta
40617	A	28	M	No	No	No	No	NA	NA	NA	CanSino	Apr-21	NA	7/12/2021	96.8	B.1.617.2	Delta
40624	A	34	F	No	No	No	No	NA	NA	NA	CanSino	Apr-21	NA	7/12/2021	92.8	B.1.617.2	Delta
40625	A	27	F	No	No	No	No	NA	NA	NA	CanSino	Mar-21	NA	7/12/2021	99.6	B.1.621	Mu
40627	A	45	F	No	No	Yes	No	NA	NA	NA	CanSino	Apr-21	NA	7/12/2021	98.0	B.1.617.2	Delta
40707	A	20	F	No	No	No	No	NA	NA	NA	CanSino	Apr-21	NA	7/13/2021	98.7	B.1.617.2	Delta
40712	A	24	M	No	No	No	No	NA	NA	NA	CanSino	May-21	NA	7/13/2021	96.8	B.1.617.2	Delta
40732	A	54	M	No	No	No	No	NA	NA	NA	Sinovac	Mar-21	Apr-21	7/13/2021	98.7	B.1.618	
40735	A	58	M	Yes	Yes	No	No	NA	NA	NA	CanSino	Apr-21	NA	7/13/2021	98.7	B.1	
40737	A	27	M	No	No	No	No	NA	NA	NA	CanSino	Apr-21	NA	7/13/2021	96.7	AY.3	Delta
40812	A	29	M	No	No	No	No	NA	NA	NA	CanSino	Apr-21	NA	7/14/2021	97.8	AY.3	Delta
40819	A	40	M	No	No	No	No	NA	NA	NA	CanSino	Apr-21	NA	7/14/2021	98.7	AY.3	Delta
40826	A	71	F	No	Yes	No	No	NA	NA	NA	Pfizer/ BioNTech	Apr-21	May-21	7/14/2021	97.8	AY.3	Delta
40833	A	21	M	No	No	No	No	NA	NA	NA	CanSino	Apr-21	NA	7/14/2021	97.7	B.1.617.2	Delta
40834	A	75	M	Yes	Yes	No	No	NA	NA	NA	Pfizer/ BioNTech	Apr-21	May-21	7/14/2021	98.6	B.1.617.2	Delta
40875	A	59	F	No	No	No	No	NA	NA	NA	Sinovac	Apr-21	May-21	7/14/2021	98.7	AY.3	Delta
40884	A	27	M	No	No	No	No	NA	NA	NA	CanSino	Apr-21	NA	7/14/2021	98.7	B.1.617.2	Delta

H: Hospitalized; A: Ambulatory; M: Male; F: Female; NA: Not applied.
