# Supplementary material for: Prevalence of SARS-CoV-2 Variants of Concern and Variants of Interest in COVID-19 Breakthrough Infections in a Hospital in Monterrey, Mexico
Source: Viruses. 2022 Jan 14;14(1):154. doi: 10.3390/v14010154 (PMC8781434; doi:10.3390/v14010154)
Supplement: Supplementary file 1 [file viruses-14-00154-s001.zip › Supplementary Table S1.pdf]

We gratefully acknowledge the following Authors from the Originating laboratories responsible for obtaining the specimens, as well as the Submitting laboratories where the genome data were generated and shared via GISAID, on which this research is based.

All Submitters of data may be contacted directly via www.gisaid.org

Authors are sorted alphabetically.

Accession ID	Originating Laboratory	Submitting Laboratory	Authors
EPI_ISL_3812643	"AR Dept. of Health-PHL, Molecular Diagnostics"	Centers for Disease Control and Prevention Division of Viral Diseases, Pathogen Discovery	Alex Burgin; Ben Rambo-Martin; Clinton Paden; Dakota Howard; Dave Wentworth; Dhwani Batra; Jasmine Padilla; Justin Lee; Krista Queen; Kristen Knipe; Kristine Lacek; Mark Burroughs; Matthew Schmerer; Meghan Bentz; Mili Sheth; Peter Cook; Sam Shepard; Sarah Nobles; Suxiang Tong; Vivien Dugan; Yvette Unoarumhi
EPI_ISL_707700, EPI_ISL_733499	1-Laboratory of Microbiology, National Reference Lab, Charles Nicolle Hospital; 2-University of Tunis ElManar, Faculty of Medicine of Tunis, LR99ES09, Tunis, Tunisia	1-Clinical and Experimental Pharmacology Lab, LR16SP02, National Center of Pharmacovigilance, University of Tunis El Manar, Tunis, Tunisia. 2-Neurodegenerative diseases and psychiatric troubles, LR18SP03, Razi Hospital, University of Tunis El Manar, Tunis, Tunisia. 3- Ministry of Health, National Observatory of New and Emerging Diseases, 1006, Tunis, Tunisia	Alia Ben Kahla; Asma Ferjani; Gaies Emna; Guedi Ali Barreh; Habiba Ben Romdhane; Hanen El Jebari; Ilhem Boutiba-Ben Boubaker; Jalila Ben Khelil; Maher Kharrat; Mouna Ben Sassi; Mouna Safer; Nissaf Ben Alaya; Riadh Daghfous; Riadh Gouider.; Rouaa Ben Othman; Salma Abid; Sameh Trabelsi; Sana Ferjani; Sarra Chamman; Souissi Amira; Zaineb Hamzaoui
EPI_ISL_2448983	105 Kresowy Szpital Wojskowy SP ZOZ w Żarach Filia w Żaganiu	1. National Institute of Public Health - National Institute of Hygiene, Warsaw, Poland 2. Biobank Lab, University of Lodz 3. Laboratory of Respiratory Viruses, Teaching and Clinical Center of the Medical University of Lodz	Dominik Strapagiel; Izabela Dróżdż; Jakub Lach; Katarzyna Zacharczuk; Klaudyna Królikowska; Maciej Borowiec; Magdalena Nowakowska; Magdalena Traczyk-Borszyńska; Marcin Słomka; Marta Sobalska-Kwapis; Małgorzata Sadkowska-Todys; Tomasz Płoszaj; Tomasz Wołkowicz
EPI_ISL_3545486, EPI_ISL_3545517	34, Alex. Fleming Str, 16672 Vari, Athens, Greece	Central National Laboratory ,Public Health Organization	A.Katsoulidou et al; G.Spanakos; Kyriaki Tryfinopoulou
EPI_ISL_3186960, EPI_ISL_3465560, EPI_ISL_3539394	4Cyte Pathology	NSW Health Pathology - Institute of Clinical Pathology and Medical Research; Westmead Hospital; University of Sydney	Arnott A.; Draper J.; Gall M.; Martinez E.; Rockett R.; Sintchenko V.; on behalf of ICPMR
EPI_ISL_3717101, EPI_ISL_3717111, EPI_ISL_3717113	54gene COVID-19 Laboratory, Nigeria	54gene Molecular Genetics Laboratory, Nigeria	Bankole Johnson; Chiamaka Nwuba; Chinenye Akpulu; Chinyere Anyika; Dr Abasi Ene Obong; Dr Colm O'Dushlaine; Dr Olajumoke Popoola; Dr Olukunke Oluwasemowo; Ifunanya Egoh; Lagos State Government; Nigerian Centre for Disease Control; Ofonime Ebong; Oluwatimilehin Adewumi; Tomiwa Adepetun
EPI_ISL_729367, EPI_ISL_729568	A. Krumbholz, Labor Dr. Krause und Kollegen MVZ GmbH, Kiel	Charité Universitätsmedizin Berlin, Institut für Virologie	Barbara Mühlemann; Christian Drosten; Julia Schneider; Jörn Beheim-Schwarzbach; Talitha Veith; Terry Jones; Victor M Corman
EPI_ISL_3275166	AA. Rasdhoo	Indira Gandhi Memorial Hospital	Dr. Milza Abdul Muhsin; Mr. Ibrahim Nishan Ahmed; Ms. Aishath Shuhudha; Ms. Aminath Nazfa; Ms. Fathimath Zimna
EPI_ISL_498523, EPI_ISL_498534, EPI_ISL_498540, EPI_ISL_498543, EPI_ISL_498546, EPI_ISL_735503, EPI_ISL_1208402, EPI_ISL_1807318, EPI_ISL_3404995			
see above	ACT Pathology	Schwessinger Lab	Ashley Jones; Benjamin Schwessinger; Craig Kennedy; Emma Crean; Karina Kennedy; Kevin Murray; Megan McDonald; Ming-Dao Chia; Robert Lanfear; Robyn N Hall
EPI_ISL_3276535	ADILAB	Universidad Nacional de Colombia - Laboratorio Genómico One Health	Andres F. Cardona-Rios; Carlos Franco-Muñoz; Carolina Muñoz-Arango; Celeny Ortiz; Daniel O. Maldonado-Perez; Diego A. Álvarez-Díaz; Hector Alejandro Ruiz-Moreno; Idabely Betancur Ortiz; Jorge E. Osorio; Juan P. Hernandez-Ortiz; Karl A Ciuoderis; Katherine Laiton-Donato; Laura Silvana Perez; Lina M. Hurtado; Marcela Mercado-Reyes; Maria Angélica Maya; Maria Stella López; Rita Almanza Payares; Sandra Ines Cano; Simón Villegas Velásquez
EPI_ISL_3118670	AFRICA_CDC - Angola	KRISP, KZn Research Innovation and Sequencing Platform	Emmanuel SJ; Giandhari J; Lessells R; Pillay S; Tegally H; Wilkinson E; Yajna R; de Oliveira T
EPI_ISL_778826	AIID	Irish Coronavirus Sequencing Consortium-Teagasc Grange	Aljandro Abner Garcia Leon; Calum Walsh; Fiona Crispie; Gabriel Gonzalez; John Kenny; Matthew McCabe; Michael Carr; Patrick Mallon; Paul Cotter
EPI_ISL_1220052	ANALICEMOS LABORATORIO ESPECIALIZADO	Instituto Nacional de Salud- Dirección de Investigación en Salud Pública	Carlos Franco-Muñoz; Diego A. Álvarez-Díaz; Diego Andrés Prada; Gerardo Santamaría; Hector Alejandro Ruiz-Moreno; Jhonnatan Reales-González; Julian Naizaque; Katherine Laiton-Donato; Magdalena Wiesner; Marcela Mercado-Reyes.; Maria T. Herrera-Sepúlveda; Martha Lucia Ospina Martínez; Sheryll Corchuelo
EPI_ISL_3071138, EPI_ISL_3155073, EPI_ISL_3155074, EPI_ISL_3155339	ANOUAL	ANOUAL	Farah Jouali; Fatima Zahra El ansari; Fatima zahra El ansari; Fatima zahra el ansari; Fekkak Jamal.; Jamal Fekkak.; Mohcine Bennani Mechita; Rachid Benhida; Yassine Kasmi
EPI_ISL_3037812	AREA DE SALUD BAGACES	Inciensa, Instituto Costarricense de Investigación y Enseñanza en Nutrición y Salud	Adriana Godínez; Caterina Guzmán; Claudio Soto-Garita; Estela Cordero; Francisco Duarte; Hebleen Porras; José Luis Vargas; Mariela Gutiérrez Joselyn Prado; Melany Calderón; Nazareth Ruiz & Adriana Bermúdez
EPI_ISL_2827995	AREA DE SALUD CARTAGO	Inciensa, Instituto Costarricense de Investigación y Enseñanza en Nutrición y Salud	Adriana Godínez; Claudio Soto-Garita; Estela Cordero; Francisco Duarte; Hebleen Porras; Joselyn Prado & Monserrat Segura; José Luis Vargas; Mariela Gutiérrez; Melany Calderón
EPI_ISL_2502735	AREA DE SALUD CATEDRAL NORESTE	Inciensa, Instituto Costarricense de InvestigaciOn y Enseñanza en NutriciOn y Salud	Adriana Godínez; Claudio Soto-Garita; Estela Cordero; Francisco Duarte; Hebleen Porras; Jose Luis Vargas; Joselyn Prado & Mariel LOpez; Mariela Gutierrez; Melany CalderOn
EPI_ISL_3639052	AREA DE SALUD CAÑAS	Inciensa, Instituto Costarricense de Investigación y Enseñanza en Nutrición y Salud	Adriana Godínez; Claudio Soto-Garita; Estela Cordero; Francisco Duarte; Hebleen Porras; Joselyn Prado & Adriana Bermúdez; José Luis Vargas; Mariela Gutiérrez; Melany Calderón
EPI_ISL_1201437, EPI_ISL_3464520	AREA DE SALUD COTO BRUS	Inciensa, Instituto Costarricense de Investigación y Enseñanza en Nutrición y Salud	Adriana Godínez; Claudio Soto-Garita; Estela Cordero; Francisco Duarte; Hebleen Porras; Joselyn Prado & Yendri Ramírez Alpízar; José Luis Vargas; Mariela Gutiérrez; Melany Calderón; Melany Calderón & Mónica Charpentier-Artavia
EPI_ISL_2658276	AREA DE SALUD EL GUARCO	Inciensa, Instituto Costarricense de Investigación y Enseñanza en Nutrición y Salud	Adriana Godínez; Claudio Soto-Garita; Estela Cordero; Francisco Duarte; Hebleen Porras; Joselyn Prado & Pamela Serrano Valerín; José Luis Vargas; Mariela Gutiérrez; Melany Calderón
EPI_ISL_3026022	AREA DE SALUD EL GUARCO [EL GUARCO/CARTAGO]	Inciensa, Instituto Costarricense de Investigación y Enseñanza en Nutrición y Salud	Adriana Godínez; Caterina Guzmán; Claudio Soto-Garita; Estela Cordero; Francisco Duarte; Hebleen Porras; Joselyn Prado; José Luis Vargas; Mariela Gutiérrez; Melany Calderón; Nazareth Ruíz & Mónica Charpentier
EPI_ISL_3298354	AREA DE SALUD FORTUNA	Inciensa, Instituto Costarricense de Investigación y Enseñanza en Nutrición y Salud	Adriana Godínez; Claudio Soto-Garita; Estela Cordero; Francisco Duarte; Hebleen Porras; Joselyn Prado & Carolina Arrieta; José Luis Vargas; Mariela Gutiérrez; Melany Calderón
EPI_ISL_2502736	AREA DE SALUD GUATUSO	Inciensa, Instituto Costarricense de InvestigaciOn y Enseñanza en NutriciOn y Salud	Adriana Godínez; Claudio Soto-Garita; Estela Cordero; Francisco Duarte; Hebleen Porras; Jose Luis Vargas; Joselyn Prado & Francisco ChacOn-Valverde; Mariela Gutierrez; Melany CalderOn
EPI_ISL_3639156	AREA DE SALUD LIMON	Inciensa, Instituto Costarricense de Investigación y Enseñanza en Nutrición y Salud	Adriana Godínez; Claudio Soto-Garita; Estela Cordero; Francisco Duarte; Hebleen Porras; Joselyn Prado & Jose Zúñiga; José Luis Vargas; Mariela Gutiérrez; Melany Calderón
EPI_ISL_3037804	AREA DE SALUD OSA	Inciensa, Instituto Costarricense de Investigación y Enseñanza en Nutrición y Salud	Adriana Godínez; Caterina Guzmán; Claudio Soto-Garita; Estela Cordero; Francisco Duarte; Hebleen Porras; José Luis Vargas; Mariela Gutiérrez Joselyn Prado; Melany Calderón; Nazareth Ruiz & Mariamilia Cob
EPI_ISL_2502727	AREA DE SALUD SAN JUAN-SAN DIEGO-CONCEPCION 2	Inciensa, Instituto Costarricense de InvestigaciOn y Enseñanza en NutriciOn y Salud	Adriana Godínez; Claudio Soto-Garita; Estela Cordero; Francisco Duarte; Hebleen Porras; Jose Luis Vargas; Joselyn Prado & Mariel LOpez; Mariela Gutierrez; Melany CalderOn
EPI_ISL_3638820	AREA DE SALUD SAN JUAN-SAN DIEGO-CONCEPCION 2	Inciensa, Instituto Costarricense de Investigación y Enseñanza en Nutrición y Salud	Adriana Godínez; Claudio Soto-Garita; Estela Cordero; Francisco Duarte; Hebleen Porras; José Luis Vargas; Mariela Gutiérrez & Joselyn Prado; Melany Calderón
EPI_ISL_2502737	AREA DE SALUD SIQUIRRES	Inciensa, Instituto Costarricense de InvestigaciOn y Enseñanza en NutriciOn y Salud	Adriana Godínez; Claudio Soto-Garita; Estela Cordero; Francisco Duarte; Hebleen Porras; Jose Luis Vargas; Joselyn Prado & Ileana Cháves-Peraza; Mariela Gutierrez; Melany CalderOn
EPI_ISL_3638878	AREA DE SALUD SIQUIRRES	Inciensa, Instituto Costarricense de Investigación y Enseñanza en Nutrición y Salud	Adriana Godínez; Claudio Soto-Garita; Estela Cordero; Francisco Duarte; Hebleen Porras; José Luis Vargas; Mariela Gutiérrez & Joselyn Prado; Melany Calderón
EPI_ISL_3037839	AREA DE SALUD SIQUIRRES [SIQUIRRES/LIMON]	Inciensa, Instituto Costarricense de Investigación y Enseñanza en Nutrición y Salud	Adriana Godínez; Caterina Guzmán; Claudio Soto-Garita; Estela Cordero; Francisco Duarte; Hebleen Porras; José Luis Vargas; Mariela Gutiérrez Joselyn Prado; Melany Calderón; Nazareth Ruiz & Ileana Chavez Peraza
EPI_ISL_914804	AREA DE SALUD TURRIALBA-JIMENEZ	Inciensa, Instituto Costarricense de Investigación y Enseñanza en Nutrición y Salud	Adriana Godínez; Claudio Soto-Garita; Estela Cordero; Francisco Duarte; Hebleen Porras; Melany Calderón & Mónica Charpentier-Artavia
EPI_ISL_2658269	AREA DE SALUD ZAPOTE-CATEDRAL - CLINICA DR. CARLOS DURAN	Inciensa, Instituto Costarricense de Investigación y Enseñanza en Nutrición y Salud	Adriana Godínez; Claudio Soto-Garita; Estela Cordero; Francisco Duarte; Hebleen Porras; Joselyn Prado & Andony Cordero-Jiménez; José Luis Vargas; Mariela Gutiérrez; Melany Calderón
EPI_ISL_2248967	ARS Algarve - Laboratorio Laura Ayres	Instituto Nacional de Saude (INSA)	Borges et al
EPI_ISL_1811231	AS Alajuela Central	Inciensa, Instituto Costarricense de Investigación y Enseñanza en Nutrición y Salud	Pérez-Corrales C
EPI_ISL_3308820	AULSS 5 Polesana	Istituto Zooprofilattico Sperimentale delle Venezie	Adelaide Milani; Alessia Schivo; Alice Fusaro; Ambra Pastori; Annalisa Salviato; Antonia Ricci; Calogero Terregino; Edoardo Giussani; Elisa Palumbo; Erika Giorgia Quaranta; Isabella Monne; Luca Tassoni
EPI_ISL_3690016	AZ St.Jan Brugge-Oostende	AZ Sint Jan	Jorn Hellemans; Laurien Hoornaert; Marie Madeleine Chabert-Consen; Marijke Reynders; Patrick Descheemaeker; Thomas Van Landschoot
EPI_ISL_517616,	Academic Hospital Paramaribo	Erasmus Medical Center	Bas Oude Munnink; Dion Gajadin; Ed Ijzerman; Emmanuelle Munger; Gary Gummels; Ingrid Krishnadath; Lycke Woittiez; Marion Koopmans; Mireille Van de Veer; Princes Wongsowidjojo; Radjesh Ori; Rohma Banwari; Stephen Vreden

EPI_ISL_517650, EPI_ISL_518812			
EPI_ISL_1445299, EPI_ISL_2042690, EPI_ISL_2145847, EPI_ISL_2145848, EPI_ISL_2145857, EPI_ISL_2145892, EPI_ISL_2146046, EPI_ISL_2146125, EPI_ISL_2146283, EPI_ISL_2146828, EPI_ISL_2147011, EPI_ISL_2148014, EPI_ISL_2148640, EPI_ISL_2159337, EPI_ISL_2187461, EPI_ISL_2243352, EPI_ISL_2528396, EPI_ISL_2528739, EPI_ISL_2528909, EPI_ISL_2687282, EPI_ISL_2875625, EPI_ISL_2877692, EPI_ISL_3220837, EPI_ISL_3321774, EPI_ISL_3752215, EPI_ISL_3752281, EPI_ISL_3812864, EPI_ISL_3819372, EPI_ISL_3820532, EPI_ISL_3820645, EPI_ISL_3822261			
see above	Aegis Sciences Corporation	Centers for Disease Control and Prevention Division of Viral Diseases, Pathogen Discovery	Adrian Paskey; Alec Vest; Benjamin Rambo-Martin; Christopher Gulvick; Clinton Paden; Clinton R. Paden; Cyndi Clark; Dakota Howard; Darlene Wagner; Dhwani Batra; Dillon Nall; Duncan MacCannell; Ethan Sanders; Holly Houdeshell; Jason Caravas; Kara Moser; Matthew Hardison; Matthew Schmerer; Ola Kvalvaag; Patrick Campbell; Peter Cook; Peter W. Cook; Rob Case; Scott Sammons; Shatavia Morrison; Shaun Westlund; Vikramsinha Ghorpade; Yvette Unoarumhi
EPI_ISL_2360250, EPI_ISL_2360251	Afzalipoor Hospital	National Influenza Center	A Nejati; F Ajaminejad and T Mokhtari Azad; J Yavarian; K Sadeghi; N Ghavvami; NZ Shafiei Jandaghi; V Salimi
EPI_ISL_3231382	Airport Health Laboratory/Central Health Laboratory	UMR PIMIT	Bahadoor BS; David Wilkinson; Manraj SS; Patrick Mavingui; Pattoo M; Ramuth M; Sonoo J
EPI_ISL_3217420	Airport Health Laboratory/Central Health Laboratory	Virology Department, Central Health Laboratory ,Victoria Hospital, Candos,Ministry of Health and Wellness, Mauritius	Bahadoor BS; Jannoo N; Manraj SS; Mathur H; Pattoo M; Ramuth M; Sonoo J; Sujeewon C
EPI_ISL_1578662, EPI_ISL_2007366, EPI_ISL_2612074, EPI_ISL_3152652, EPI_ISL_3229858	Akershus University Hospital, Department for Microbiology and Infectious Disease Control	Norwegian Institute of Public Health, Department of Virology	Atiya R Ali; Debech Nadia; Engebretsen Serina Beate; Garcia Llorente Ignacio; Hilde Elshaug; Hilde Vollan; Jon Bråte; Kamilla Heddeland Instefjord; Karoline Bragstad; Kathrine Stene-Johansen; Line Victoria Moen; Marie Paulsen Madsen; Olav Hungnes; Pedersen Benedikte Nevjen; Rasmus Riis Kopperud
EPI_ISL_2136787, EPI_ISL_2187670, EPI_ISL_2626716, EPI_ISL_3118600, EPI_ISL_3654155	Alaska State Virology Laboratory	Alaska State Virology Laboratory	Elva House; Jack Chen; Jacob Zidek; Lisa Smith; Ph.D.; Stephanie DeRonde
EPI_ISL_1385804	Alfa Diagnostica, Republic of Moldova	ONCOGENE LLC	ONCOGENE LLC
EPI_ISL_3691944	Algemeen Medisch Labo	Labo Klinische Biologie, UZA	Basil Britto Xavier; Christine Lammens; Herman Goossens; Ines Verbesselt; Jasmine Coppens; Kathleen Holemans; Marie Le Mercier; Veerle Matheeussen
EPI_ISL_2877016, EPI_ISL_3275395, EPI_ISL_3275415, EPI_ISL_3534240, EPI_ISL_3720837	Allergy, Immunology and Cell Biology Unit (AICBU)	Allergy, Immunology and Cell Biology Unit (AICBU)	Ayesha Wijesinghe; Chandima Jeewandara; Deshan Madhusanka; Deshni Jayathilaka; Dinuka Ariyarathne; Diyanath Ranasinghe; Dumni Gunasinghe; Gathsaurie Neelika Malavige; Tibutius Thanesh
EPI_ISL_2375992	Alma CDC wc AHC	NHLS/UCT	Arash Iranzadeh; Bruna Galvao; Carolyn Williamson; Deelan Doolabh; Diana Hardie; Innocent Mudau; Kruger Marais; Lynn Tyers; Marvin Hsiao; Stephen Korsman
EPI_ISL_1324584	Anteja laboratorija (UAB Diagnostikos laboratorija)	Vilnius University Hospital Santaros Klinikos, Center of Laboratory Medicine	Daniel Naumovas; Dovile Ezerskyte; Gytis Dudas; Ingrida Olendraite; Laimonas Griskevicius; Ligita Raugaite; Mindaugas Stoskus; Monika Katenaite; Rimvydas Norvilas
EPI_ISL_527747	Area De Salud La Cruz	Inciensa, Instituto Costarricense de Investigación y Enseñanza en Nutrición y Salud	Adriana Godinez & Melany Calderon; Claudio Soto-Garita; Estela Cordero; Francisco Duarte; Hebleen Porras
EPI_ISL_770007	Area De Salud San Rafael	Inciensa, Instituto Costarricense de Investigación y Enseñanza en Nutrición y Salud	Adriana Godínez; Claudio Soto-Garita; Estela Cordero; Francisco Duarte; Hebleen Porras; Melany Calderón & Mariel López
EPI_ISL_509499, EPI_ISL_527011	Area of Virology, Serology and Virology Division (SAViD), New South Wales Health Pathology Randwick	Area of Virology, Serology and Virology Division (SAViD), New South Wales Health Pathology Randwick	Rawlinson, W.
EPI_ISL_678293, EPI_ISL_678373, EPI_ISL_812347, EPI_ISL_1911191, EPI_ISL_2404987, EPI_ISL_2404988, EPI_ISL_3568299, EPI_ISL_3568459			
see above	Area of Virology, Serology and Virology Division (SAViD), New South Wales Health Pathology Randwick	Virology Research Laboratory; Area of Virology, Serology and Virology Division (SAViD), New South Wales Health Pathology Randwick	Au, J.; Bull, R.; Deveson, I.; Foster, C.; Rawlinson, W.; Ruiz Silva, M.; Van Hal, S.
EPI_ISL_1113546	Arizona Department of Health Services	TGen North	"Jolene Bowers; Ashlyn Pfeiffer; Chris French; Darrin Lemmer; Dave Engelthaler; Hayley Yaglom; Megan Folkerts; The Arizona COVID Genomics Union (ACGU)"
EPI_ISL_2227487	Arizona State Public Health Laboratory	Arizona State Public Health Laboratory	Jessica Escobar; Katherine Fullerton; Linda Getsinger; Nobuko Fukushima; Stacy White; Trung Huynh; Victor Waddell
EPI_ISL_3505726	Arizona State University	Arizona State University	Ajeet Bains; Efrem S. Lim; Joshua LaBaer; LaRinda A. Holland; Matthew F. Smith; Nathaniel Johnson; Nicholas J. Mellor; Peter T. Skidmore; Rabia Maqsood; Vel Murugan
EPI_ISL_2931041	Arkansas Children's Hospital	Center for Global Health, University of New Mexico Health Sciences Center	Amanda Novack; Darrell Dinwiddie; Daryl Domman; Dirk Haselow; Joshua L. Kennedy; Kurt Schwalm; Valerie Morley
EPI_ISL_3474080	Arkansas Public Health Laboratory, Arkansas Department of Health	University of Minnesota Genomics Center	Corbin Dirkx; Daryl M. Gohl; Jaquelyn Kuriger-Laber; John Garbe
EPI_ISL_3425318	Arlon	Plateforme de testing Namuroise	Degosserie Jonathan; Demars Aurore; Denis Olivier; Lesly Nyinkeu Kemamen; Maschietto Céline; Mullier François; Nicolas Gilliard; Nobis Chloé; Otto Gaetan
EPI_ISL_539811	Asiaworld Expo Command Post	Hong Kong Department of Health	Alan K.L. Tsang; Dominic N.C. Tsang; Edman T.K. Lam; Peter C.W. Yip; Rickjason C.W. Chan
EPI_ISL_413490	Auckland Hospital	Institute of Environmental Science and Research (ESR)	Erasmus Smit; Gary McAuliffe; Joep de Ligt; Lauren Jelly; Matt Storey; Matthew Blakiston; Sally Roberts; Xiaoyun Ren
EPI_ISL_1008159, EPI_ISL_1117860, EPI_ISL_2232806, EPI_ISL_2232858, EPI_ISL_2427023, EPI_ISL_2427077, EPI_ISL_2427157, EPI_ISL_2757972, EPI_ISL_2758172, EPI_ISL_2887671, EPI_ISL_3546555, EPI_ISL_3797127, EPI_ISL_3797156, EPI_ISL_3797222			
see above	Austrian Agency for Health and Food Safety (AGES)	Bergthaler laboratory, CeMM Research Center for Molecular Medicine of the Austrian Academy of Sciences	Andreas Bergthaler; Anna Schedl; Bekir Erguner; Benedikt Agerer; Christoph Bock; Fabian Amman; Jan Laine; Lukas Endler; Maelle Le Moing; Martin Senekowitsch; Matthew Thornton; Michael Schuster; Petr Triska; Thomas Penz
EPI_ISL_3721613	Ayudas Diagnosticas Sura	Universidad Nacional de Colombia - Laboratorio Genómico One Health	Andres F. Cardona-Rios; Carlos Franco-Muñoz; Carolina Muñoz-Arango; Celeny Ortiz; Daniel O. Maldonado-Perez; Diego A. Álvarez-Díaz; Hector Alejandro Ruiz-Moreno; Idabely Betancur Ortiz; Jorge E. Osorio; Juan P. Hernandez-Ortiz; Karl A Ciuoderis; Katherine Laiton-Donato; Laura Silvana Perez; Lina M. Hurtado; Marcela Mercado-Reyes; Maria Angélica Maya; Maria Stella López; Rita Almanza Payares; Sandra Ines Cano; Simón Villegas Velásquez
EPI_ISL_2080337	Azienda Sanitaria dell'Alto Adige Laboratorio Aziendale di Microbiologia e Virologia	Istituto di Genomica Applicata	Davide Scaglione; Eleonora Paparelli; Elisa Masi; Elisabetta Giacobazzi; Elisabetta Pagani; Gabriele Magris; Irena Jurman; Irene Bianconi; Michele Morgante; Stefanie Wieser; Vera Vendramin
EPI_ISL_2710308	BARC / Lancet	KRISP, KZN Research Innovation and Sequencing Platform	Giandhari Jennifer; Naidoo Yeshnee; Pillay Sureshnee; San James; Sisonke; Tegally Houriiyah; Tshabuila Derek; Wilkinson Eduan; Yajna Ramphal; de Oliveira Tulio
EPI_ISL_3138931	BBTKL PP Banjarbaru	National Institute of Health Research and Development	Arie Ardiansyah Nugraha; Fajar Nur Sulistiyohadi; Hana Apsari Pawestri; Hartanti Dian Ikawati; Kartika Dewi Puspa; Krisna Pangesti; Nelly Puspandari; Subangkit; Triyani Soekarso; Vivi Setiawaty
EPI_ISL_2493764	BIOFAST	Instituto Butantan	Antonio Jorge Martins; Claudia Renata dos Santos Barros; David Schlesinger; Debora Botequio Moretti; Dimas Tadeu Covas; Elaine Cristina Marqueze; Elaine Vieira Santos; Evandra Strazza Rodrigues; Heidge Fukumasu; Jayme Augusto de Souza-Neto; José Salvatore Leister Patané; Luiz Alcantara; Luiz Lehmann Coutinho; Maria Carolina Elias; Maurício Lacerda Nogueira; Rafael dos Santos Bezerra; Raul Machado Neto; Rejane Maria Tommasini Grotto; Ricardo Haddad; Sandra Coccuzzo Sampaio Vessoni; Simone Kashima; Svetoslav Nanev Slavov; Vincent Louis Viala
EPI_ISL_2983838	BIOMNIS EUROFINS IVRY	Department of Virology, Henri Mondor University Hospital, Assistance Publique Hôpitaux de Paris, Université Paris-Est Créteil, INSERM U955	Alexandre Soulier; Christophe Rodriguez; Elisabeth Trawinski; Guillaume Gricourt; Jean-Michel Pawlotsky; Melissa N'Debi; Slim Fourati; Vanessa Demontant
EPI_ISL_3130098, EPI_ISL_3130099, EPI_ISL_3130100	BIOMNIS LYON	CNR Virus des Infections Respiratoires - France SUD	Antonin Bal; Bruno Lina; Gregory Destras; Gwendolyne Burfin; Hadrien Regue; Laurence Josset; Martine Valette; Quentin Semanas
EPI_ISL_859610, EPI_ISL_859920, EPI_ISL_860055	BTC, Khalifa University	BTC, Khalifa University	Al Safar et al
EPI_ISL_3070926	BTKLPP Kelas I Batam	Eijkman Institute for Molecular Biology, National Research and Innovation Agency; National Institute of Health Research and Development	Amin Soebandrio; Edison Johar; Frilasita A Yudhaputri; Hana Apsari Pawestri; Hidayat Trimarsanto; Iskandar Adnan; Khin Saw Myint; Lidwina Priliani; Lydia V. Panggalo; Muhammad Rezki Rasyak; Safarina G Malik; Subangkit; Sukma Oktavianthi; Vivi Setiawaty; Willy Agustine
EPI_ISL_2859217	Baguio General Hospital Medical Center (BGHMC)	Philippine Genome Center	Alethea R. de Guzman; Anna Ong-Lim; Arianne A. Zamora; Benedict A. Maralit; Carlo M. Lapid; Celia Carlos; Devon Ray Pacial; Diomedes A. Carino; Edsel Maurice Salvaña; El King D. Morado; Elcid Aaron R. Pangilinan; Eva Maria Cutiongco-de la Paz; Francis A. Tablizo; Henrietta Marie Rodriguez; Jaime C. Montoya; Jan Michael C. Yap; Jarvin E. Nipales; Jo-Hannah S. Llames; John Q. Wong; Joshua Gregor A. Dizon; Juan Antonio R. Magalang; Karol Sophia Agape R. Padilla; Kenneth M. Kim; Kris P. Punayan; Krisitna Patriz Dela Cruz; Lindsay Claire D.L. Carandang; Ma. Exanil Plantig; Marc Edsel C. Ayes; Maria Rosario Singh-Vergeire and Cynthia P. Saloma; Maria Sofia L. Yangzon; Marielle M Gamboa; Marissa Alejandria; Nina Francesca Bustamante; Razel Nikka M. Hao; Renato Jacinto Q. Mantaring; Rianna Patricia S. Cruz; Sheila Mae M. Araiza; Yvonne Valerie Austria; Zipporah Mariebelle R. Enriquez; Zyrel V. Mollejon
EPI_ISL_3394987	Balai Penelitian dan Pengembangan Aceh	National Institute of Health Research and Development	Arie Ardiansyah Nugraha; Fahmi Ichwansyah; Hana Apsari Pawestri; Hartanti Dian Ikawati; Kartika Dewi Puspa; Krisna Pangesti; Nelly Puspandari; Subangkit; Triyani Soekarso; Vivi Setiawaty
EPI_ISL_434693	Bamrasnaradura hospital	National Institute of Health. Department of medical Sciences, Ministry of Public Health, Thailand	Chittaganpitch; Malinee; Okada; Parnmen; Phuygun; Pilailuk; Siripaporn; Sittiporn; Sunthareeya; Thanadachakul; Thanutsapa; Waicharoen; Warawan; Wongboot
EPI_ISL_2801892, EPI_ISL_3118366	Banteay Meanchey Regional Laboratory	Virology Unit, Institut Pasteur du Cambodge	Cecile Troupin; Chau Darapheak; Chin Savuth; Erik A Karlsson; Jurre Y Siegers; Kraing Sidonn; Leakhena Pum; Ly Sovann; Sophoannadedh Rath; Veasna Duong; Yi Sengdoeurn

EPI_ISL_2484708, EPI_ISL_3274242	Baptist Health Medical Center	Center for Global Health, University of New Mexico Health Sciences Center	Amanda Novack; Darrell Dinwiddie; Daryl Domman; Dirk Haselow; Joshua L. Kennedy; Kurt Schwalm; Valerie Morley
EPI_ISL_509714	Belize Ministry of Health	Pathogen Discovery, Respiratory Viruses Branch, Division of Viral Diseases, Centers for Disease Control and Prevention	Anna Uehara; Clinton Paden; Haibin Wang; Jing Zhang; Krista Queen; Suxiang Tong; Yan Li; Ying Tao
EPI_ISL_2802860	BioMoLab	Microbiologia Molecular, Instituto SELADIS, Universidad Mayor de San Andrés	Aneth Vasquez Michel; Carmen Delgado Barrera; Oscar M. Rollano-Peñaloza; Sandra Miranda Sardon
EPI_ISL_2802858, EPI_ISL_2802859, EPI_ISL_2802863	BioMoLab	Molecular Genetics Laboratory, Instituto de Investigaciones Químicas, Universidad Mayor de San Andrés	Aneth Vasquez Michel; Carmen Delgado Barrera; Oscar M. Rollano-Peñaloza; Sandra Miranda Sardon
EPI_ISL_1273096	Biochemistry and Molecular Biology Department-Faculty of Medicine, Al-Quds University	Biochemistry and Molecular Biology Department-Faculty of Medicine, Al-Quds University	Al-Jawabreh, A.; Al-Jawabreh, H.; Dumaidi, K.; Ereqat, S.; Nasereddin, A.
EPI_ISL_924608	Bioinformatics and Biostatistics Lab, Advanced Sequencing Facility	COVID-19 Genomics UK (COG-UK) Consortium	Aengus Stewart; Chelsea Sawyer; Harshil Patel; Jerome Nicod; Laura Cubitt; Margaret Crawford
EPI_ISL_730514, EPI_ISL_730551, EPI_ISL_730555, EPI_ISL_878463, EPI_ISL_878571	Biolab Diagnostic Laboratories	Andersen lab at Scripps Research	Ahmad Tibi; Amid Abdelnour with SEARCH Alliance San Diego; Issa Abu-Dayyeh; Lama Hussein; Lina Mohammad; Zein Naber
EPI_ISL_1970834, EPI_ISL_2105674, EPI_ISL_2658760, EPI_ISL_2868422	Biolab Diagnostic Laboratories	Biolab Diagnostic Laboratories	Ahmad Tibi; Amid Abdelnour; Badia Saddedin; Eiad Atwa; Issa Abu-Dayyeh; Lama Hussein; Shaima Ali; Shayma Ali
EPI_ISL_3446769	Biological Hazard and Health Research Laboratory, Centre for Advanced Research in Science (CARS), University of Dhaka	Genomic Research Lab, BCSIR	Abu Sayeed Mohammad Mahmud; Barna Goswami; Eshrar Osman; Iffat Jahan; Latiful Bari; Md Abdul Malek; Md Mizanur Rahaman; Md. Ahasan Habib; Md. Kamrul Islam; Md. Murshed Hasan Sarkar; Md. Salim Khan; Md. Shaminur Rahman; Mohammad Mohi Uddin; Mohammad Samir Uzzaman; Shahina Akter; Tanjina Akhter Banu
EPI_ISL_526980	Biological prevention, army	Biological prevention, army	A.F.; B.E.; Elhosieny; Gad; Harty; M.D.; M.F.; M.G.; Seadawy; Shamel
EPI_ISL_1524330, EPI_ISL_2931131, EPI_ISL_2931135, EPI_ISL_2931137, EPI_ISL_2931138	Biology Department, College of Science, Al Muthanna University and Public Health Laboratory, Al-Muthanna Health Directorate	Department of Virology, Faculty of Medicine, University of Helsinki, Helsinki, Finland	Alaa Hameed; Ali Jasim; Batool Kadham Salman; Hussein Alburkat; Hussein Riadh Kitab; Murad Munahi; Nihad Al-Rashedi; Olli Vapalahti; Tarja Sironen; Teemu Smura
EPI_ISL_1420639	Biomedical Research Foundation of the Academy of Athens (BRFAA)	Greek Genome Center, Biomedical Research Foundation of the Academy of Athens (BRFAA)	Dimitrios Thanos; Emmanouil Athanasiadis; Ioannis Vatsellas; Katerina Zoi; Theodoros Loupis
EPI_ISL_3145270, EPI_ISL_3145921	BioneXt Lab	Laboratoire national de sante, Microbiology, Microbial Genomics Platform	Anke Wienecke-Baldacchino; Catherine Ragimbeau; Elodie Solarino; Fatu Djabi; Jessica Tapp; Lise Pignon; Raoul Salmon; Tamir Abdelrahman; Thibault Ferrandon; Virginie Jover
EPI_ISL_2464487, EPI_ISL_2983948, EPI_ISL_3390947	Biopole Antilles	Department of Virology, Henri Mondor University Hospital, Assistance Publique Hôpitaux de Paris, Université Paris-Est Créteil, INSERM U955	Alexandre Soulier; Christophe Rodriguez; Elisabeth Trawinski; Guillaume Gricourt; Jean-Michel Pawlotsky; Melissa N'Debi; Slim Fourati; Vanessa Demontant
EPI_ISL_985069, EPI_ISL_985129	Biorepository and Clinical Virology Laboratory	Ozer Lab	Adeola A. Fowotade; Babafemi O. Taiwo; Egon A. Ozer; Ewean C. Omoruyi; Johnson A. Adeniji; Judd F. Hultquist; Lacy M. Simons; Olubusuyi M. Adewumi; Ramon Lorenzo-Redondo
EPI_ISL_3712206	Bioscientia Labor Wermsdorf	Robert Koch Institute	
EPI_ISL_1363123, EPI_ISL_2535741	Borneo Medical Centre	Institute of Health and Community Medicine	Chan Chia Jui; Chua Hock Hin; David Perera; Ooi Mong How; Tonnii Sia Loong Loong; Wong Jyn Shan; Wong Kiing Aik
EPI_ISL_1516860, EPI_ISL_2372273, EPI_ISL_2372301, EPI_ISL_2386154, EPI_ISL_2504068, EPI_ISL_2820447, EPI_ISL_2868344, EPI_ISL_2868350, EPI_ISL_2868357, EPI_ISL_2931811, EPI_ISL_2931830, EPI_ISL_3453911, EPI_ISL_3453913, EPI_ISL_3453914, EPI_ISL_3453916, EPI_ISL_3453917			
see above	Botswana Harvard HIV Reference Laboratory	Botswana Harvard HIV Reference Laboratory	Boitumelo J.L Zuze; Boitumelo Zuze; Botshelo Radibe; David Lawrence; Dorcas Maruapula; Irvin Kgetse; Joseph Makhema; Kelebeletse K. Mokobela; Keoratile Ntshambiwa; Kgomotso Moruisi; Legodile Kooepile; Lindani Bochena; Madisa Mine; Modisa Motswaledi; Mosepele Mosepele; Ontlametse T. Bareng; Pamela Lawrence-Smith; Pamela Smith-Lawrence; Roger Shapiro; Shahin Lockman; Sikhulile Dorcas Maruapula; Sikhulile Moyo; Simani Gaseitsiwe; Thongbotho Mphoyakgosi; Wonderful T. Choga
EPI_ISL_903343, EPI_ISL_3669072	Bozeman Health Deaconess Hospital	Wiedenheft lab, Montana State University	Aidan McVey; Anna Nemudraia; Artem Nemudryi; Blake Wiedenheft; Calvin Cicha; Deann T. Snyder; Diane Bimczok; Helen Lee; Jodi F. Hedges; Joseph Nichols; Karl K. Vanderwood; Mark A. Jutila and Blake Wiedenheft; Tanner Wiegand
EPI_ISL_3482341, EPI_ISL_3484393, EPI_ISL_3489914, EPI_ISL_3602680	British Columbia Centre For Disease Control	BCCDC Public Health Laboratory	Ana Pacagnella; Corrinne Ng; Dan Fornika; John Tyson; Kim Macdonald; Kimia Kamelian; Linda Hoang; Loretta Janz; Mel Krajden; Prystajecky Natalie; Robert Azana; Shannon Russell
EPI_ISL_2991947	Broad Institute Clinical Research Sequencing Platform	Infectious Disease Program, Broad Institute of Harvard and MIT	A.E.; Adams, G.; Anahtar, M.; B.L.; B.W.; Bauer, M.; Birren; Branda, J.; Carter, A.; Cerrato, F.; Chaluvadi, S.; Chapman; Cusick, C.; D.J.; DeRuff, K.; Flowers, K.; Gallagher, G.; Gladden-Young, A.; Gnirke, A.; Harris, J.; J.E.; K.J.; LaRocque, R.; Lagerborg, K.; Lemieux; Lin; Loreth, C.; MacInnis; Neumann, A.; Normandin, E.; P.C.; Park; Pierce, V.; Reilly, S.; Rosenberg, E.; Rudy, M.; Ryan, E.; S.B.; Sabeti; Shaw, B.; Siddle; Slater, D.; Smole, S.; Tomkins-Tinch, C.; Turbett, S.
EPI_ISL_1972075, EPI_ISL_2312317, EPI_ISL_2312487, EPI_ISL_3007479, EPI_ISL_3134329, EPI_ISL_3431656, EPI_ISL_3520234			
see above	Broad Institute Clinical Research Sequencing Platform	Infectious Disease Program, Broad Institute of Harvard and MIT	Adams, G.; B.L.; B.W.; Bauer, M.; Birren; Blumenstiel, B.; Brown, C.; Carter, A.; Chaluvadi, S.; D.J.; DeFelice, M.; DeRuff, K.; Dodge, S.; Gabriel, S.; Gallagher, G.; Gladden-Young, A.; Granger, B.; J.E.; K.J.; Lagerborg, K.; Larkin, K.; Lee, M.; Lemieux; Lennon, N.; Loreth, C.; Madoff, L.; McGovern, S.; Meldrim, J.; Normandin, E.; P.C.; Park; Pearlman, L.; Reilly, S.; Rudy, M.; Sabeti; Siddle; Smole, S.; Tomkins-Tinch, C.; Vicente, G.; and MacInnis
EPI_ISL_1000998, EPI_ISL_1034755, EPI_ISL_1034756	Bundeswehr Institute of Microbiology	Bundeswehr Institute of Microbiology	Alexandra Rehn; Enrico Georgi; Malena Bestehorn-Willmann; Markus Antwerpen; Mathias Walter; Mike Pillukat; Roman Wölfel; Sabine Zange
EPI_ISL_1371897, EPI_ISL_1371900	C H DE LA POLYNESIE FRANCAISE	CNR Virus des Infections Respiratoires - France SUD	Antonin Bal; Bruno Lina; Gregory Destras; Gwendolyne Burfin; Hadrien Regue; Laurence Josset; Martine Valette; Quentin Semanas
EPI_ISL_2170818	CAP TORELLÓ	Banc de Sang i Teixits	Carlos Hobeich; Francisco Vidal; Irene Corrales; Lorena Ramírez; Maria Glòria Soria; Natália Comes; Nina Borràs; Noemí Gonzalez; Silvia Sauleda
EPI_ISL_3121375, EPI_ISL_3500566	CENTOGENE Frankfurt Laboratory: Niederlassung Industriepark Höchst	Robert Koch Institute	
EPI_ISL_3696694, EPI_ISL_3696714, EPI_ISL_3696757	CENTRO DE ENFRENTAMENTO COVID	Instituto Butantan	Antonio Jorge Martins; Claudia Renata dos Santos Barros; David Schlesinger; Debora Botequio Moretti; Dimas Tadeu Covas; Elaine Cristina Marqueze; Elaine Vieira Santos; Evandra Strazza Rodrigues; Heidge Fukumasu; Jayme Augusto de Souza-Neto; José Salvatore Leister Patané; Luiz Alcantara; Luiz Lehmann Coutinho; Maria Carolina Elias; Maurício Lacerda Nogueira; Rafael dos Santos Bezerra; Raul Machado Neto; Rejane Maria Tommasini Grotto; Ricardo Haddad; Sandra Coccuzzo Sampaio Vessoni; Simone Kashima; Svetoslav Nanev Slavov; Vincent Louis Viala
EPI_ISL_3385844	CENTRO DE GENETICA Y BIOLOGIA MOLECULAR UNIVERSIDAD DEL MAGDALENA	Instituto Nacional de Salud- Dirección de Investigación en Salud Pública	Carlos Franco-Muñoz; Carmen Osorio; Diana Malo; Diego A. Álvarez-Díaz; Diego Andrés Prada; Gerardo Santamaría; Hector Alejandro Ruiz-Moreno; Jhonnatan Reales-González; Jorge Rivera; Juan Camilo Martinez; Julian Naizaque; Katherine Laiton-Donato; Lisseth Pardo; Magdalena Wiesner; Marcela Mercado-Reyes; Maria T. Herrera-Sepúlveda; Marta Lopez Blanco; Martha Lucia Ospina Martinez; Paola Rojas; Sergio Gomez; Sheryll Corchuelo; Ángela Alarcon Cruz
EPI_ISL_644261	CEPHR / Vincent's Hospital	Irish Coronavirus Sequencing Consortium - National Virus Reference Laboratory	Alejandro Abner Garcia Leon; Gabriel Gonzalez; Michael Carr; Patrick Mallon
EPI_ISL_3071597	CH Porto - H Sto Antonio	Instituto Nacional de Saude (INSA)	Borges et al
EPI_ISL_3798660	CH Tondela Viseu	Instituto Nacional de Saude (INSA)	Borges et al
EPI_ISL_3758176	CHECK-UP - Medicina e Diagnóstico - Matriz	HLAGYN - Laboratorio de Imunologia de Transplantes de Goias	Elaize Maria Gomes de Paula; Fernando Antonio Vinhal dos Santos; Frederico Rodrigues Vinhal; Gladstone Rodrigues da Cunha Filho; Kamila Oliveira Reis De Freitas; Lucas Carlos Gomes Pereira; Nubia Silva Araújo; Sabrina Sara Moreira Duarte
EPI_ISL_1399890	CHMT	Instituto Nacional de Saude (INSA) and BioSystems & Integrative Sciences Institute (BioISI) Genomics Unit, FCUL	Borges et al
EPI_ISL_3432134	CHTMAD	Instituto Nacional de Saude (INSA)	Borges et al
EPI_ISL_482886	CHU Purpan - Laboratoire de Virologie - Institut Fédératif de Biologie	Laboratoire de virologie - École Nationale Vétérinaire de Toulouse	Guillaume Croville; Jacques Izopet; Jean-Luc Guérin
EPI_ISL_2445741	CHU Sao Joao, Porto	Instituto Nacional de Saude (INSA)	Borges et al
EPI_ISL_2441796	CHU de la Réunion - Nord	Laboratoire de virologie, CNR arbovirus Associé, Chu de la Réunion	Etienne Frumence; Marie-Christine Jaffar Bandjee; Nicolas Traversier; Sabrina Petit Genet
EPI_ISL_683835	CICM	Malaria Research and Training Center (MRTC-Parasito)	Abdoulaye Djimde; Antoine Dara
EPI_ISL_2683873	CICM, Bamako	Malaria Research and Training Center-Bamako	Abdoul Karim Sangare; Abdoulaye Djimde; Amadou Daou; Antoine Dara; Bourema Kouriba
EPI_ISL_2657868	COVID 19- EPS FAMISANAR	Instituto Nacional de Salud- Dirección de	Carlos Franco-Muñoz; Carmen Osorio; Diana Malo; Diego A. Álvarez-Díaz; Diego Andrés Prada; Gerardo Santamaría; Hector Alejandro Ruiz-Moreno; Jhonnatan Reales-González; Jorge Rivera; Juan Camilo Martinez; Julian Naizaque; Katherine Laiton-Donato; Lisseth Pardo; Magdalena

		Investigación en Salud Pública	Wiesner; Marcela Mercado-Reyes; Maria T. Herrera-Sepúlveda; Marta Lopez Blanco; Martha Lucia Ospina Martinez; Paola Rojas; Sergio Gomez; Sheryll Corchuelo; Ángela Alarcon Cruz
EPI_ISL_3600721	CPHL, MOH, EGYPT	CPHL, MOH, EGYPT	Abd Monaem Adel; Amelia nagiub; Dalia Ramadan; Galal Mahmoud; Mohamed A Ali; Mohamed Hassany; Mohamed K. Khalifa; Nancy el guindy; Rabeh ..R. El/Shesheny; Ramy Galal; Shymaa s. Ahmed; Wael H. Roshdy; salma sayed
EPI_ISL_3274151, EPI_ISL_3274155, EPI_ISL_3274156, EPI_ISL_3274159	CPHL/MOH/EGYPT	CPHL/MOH/EGYPT	Wael H. Roshdy/ Mohamed Kamal / Shymaa s. Ahmed/ Ramy Galal/Nancy el guindy/ Amel nagiub/salma sayed/ Abd Monaem Adel/Galal Mahmoud/Dalia Ramadan/Rabeh ..R. El/Shesheny/ Mohamed A Ali/Mohamed Hassany; Wael H. Roshdy/ Mohamed Kamal / Shymaa s. Ahmed/ Ramy Galal/Nancy el guindy/ Amelia nagiub/salma sayed/ Abd Monaem Adel/Galal Mahmoud/Dalia Ramadan/Rabeh ..R. El/Shesheny/ Mohamed A Ali/Mohamed Hassany
EPI_ISL_447859, EPI_ISL_910156, EPI_ISL_910290	CSIR-Centre for Cellular and Molecular Biology	CSIR-Centre for Cellular and Molecular Biology	Archana Bharadwaj Siva; B Himasri; Blessy B John; Dhiviya Vedagiri; Divya Gupta; Divya Tej Sowpati; Karthik Bharadwaj Tallapaka; Krishnan Harinivas Harshan; Lamuk Zaveri; Namami Gaur; Payel Mukherjee; Pratheusa Maccha; Priya Singh; Purushotham Vodnala; Rakesh K Mishra; Sakshi Shambhavi; Santosh Kumar Kuncha; Shagufta Khan; Sofia Banu; Tulasi Nagabandi; Vishal Sah; Viswagithe S L
EPI_ISL_2441676	CSIR-Centre for Cellular and Molecular Biology	CSIR-Centre for Cellular and Molecular Biology-INSACOG	; Amareshwar Vodapalli; Ara Sreenivas; Archana Bharadwaj Siva; B Himasri; Divya Tej Sowpati; Karthik Bharadwaj Tallapaka; Lamuk Zaveri; Onkar Kulkarni; Payel Mukherjee; Priya Nurkurthy; Rakesh K Mishra; Shreekant Verma; Sofia Banu; Sumedha Avadhanula; Tulasi Nagabandi; Valli Nagalakshmi Undamatla; Vidhyadhari Methuku
EPI_ISL_3060676	CSIR-National Environmental Engineering Research Institute	CSIR-Centre for Cellular and Molecular Biology-INSACOG	Amareshwar Vodapalli; Ara Sreenivas; Archana Bharadwaj Siva; B Himasri; Divya Tej Sowpati; Karthik Bharadwaj Tallapaka; Krishna Khairnar; Lamuk Zaveri; Onkar Kulkarni; Payel Mukherjee; Priya Nurkurthy; Rakesh K Mishra; Shreekant Verma; Sofia Banu; Sumedha Avadhanula; Tulasi Nagabandi; Valli Nagalakshmi Undamatla; Vidhyadhari Methuku
EPI_ISL_3320697	CT Department of Public Health	CT Department of Public Health	Claire_Pearson; Tu_N_Nguyen
EPI_ISL_2757857	CURE	Institut Pasteur de Montevideo	Andres Lizasoain; Belén González; Cecilia Alonso; Daiana Mir; Emiliano Pereira; Gonzalo Bello; Ighor Arantes; Juan Zanetti; Lucia Bilbao; Luciana Griffero; Lucía Spangenberg; Mailen Arleo; Mariana Brandes; María José Benítez-Galeano; Matías Castells; Matías Salvo; Matías Victoria; Mauricio Méndez; Melissa Duquía; Natalia Rego; Natalia Reyes; Odhille Chappos; Pablo Smircich; Pía Techera; Rodney Colina; Tamara Fernández-Calero; Tania Possi; Verónica Noya
EPI_ISL_582255, EPI_ISL_582311, EPI_ISL_582484	Cadham Provincial Laboratory	National Microbiology Laboratory (NML)	Anna Majer; Anneliese Landgraff; CanCOGeN's metadata curation team; Darian Hole; David Alexander; Elsie Grudeski; Gary Van Domselaar; Grace Seo; Jared Bullard; Jennifer Tanner; Kerry Dust; Madison Chapel; Morag Graham; Natalie Knox; Nathalie Bastien; Paul Van Caeseele; Philip Mabon; Public Health Agency of Canada CanCOGeN team; Rhiannon Huzarewich; Russell Mandes; Shari Tyson; Timothy Booth; Yan Li
EPI_ISL_2859201	Cagayan Valley Medical Center Molecular Laboratory	Philippine Genome Center	Alethea R. de Guzman; Anna Ong-Lim; Arianne A. Zamora; Benedict A. Maralit; Carlo M. Lapid; Celia Carlos; Devon Ray Pacial; Diomedes A. Carino; Edsel Maurice Salvaña; El King D. Morado; Elcid Aaron R. Pangilinan; Eva Maria Cutiongco-de la Paz; Francis A. Tablizo; Henrietta Marie Rodriguez; Jaime C. Montoya; Jan Michael C. Yap; Jarvin E. Nipales; Jo-Hannah S. Llames; John Q. Wong; Joshua Gregor A. Dizon; Juan Antonio R. Magalang; Karol Sophia Agape R. Padilla; Kenneth M. Kim; Kris P. Punayan; Krisitna Patriz Dela Cruz; Lindsay Claire D.L. Carandang; Ma. Exanil Plantig; Marc Edsel C. Ayes; Maria Rosario Singh-Vergeire and Cynthia P. Saloma; Maria Sofia L. Yangzon; Marielle M Gamboa; Marissa Alejandria; Nina Francesca Bustamante; Razel Nikka M. Hao; Renato Jacinto Q. Mantaring; Rianna Patricia S. Cruz; Sheila Mae M. Araiza; Yvonne Valerie Austria; Zipporah Mariebelle R. Enriquez; Zyrel V. Mollejon
EPI_ISL_907087	Cancer Biology Department, National Cancer Institute	Cancer Biology Department, National Cancer Institute	A.A.; A.E.; A.N.; Abouelhoda, M.; Ahmed; Bahnassy; Elhosieny; F.W.; Gad; H.K.; Hafez; Hamdy; M.G.; M.M.; M.S.; O.S.; Sedawy; Soliman; Soliman, L.; Zekri
EPI_ISL_2652216, EPI_ISL_3021346	Cantacuzino National Military-Medical Institute, Viral Respiratory Infections Laboratory	Cantacuzino Institute Virology	Carmen Cherciu; Luiza Ustea; Mihaela Lazar; Mihaela Oprea; Nicoleta Paraschiv; Sorin Dinu
EPI_ISL_548117, EPI_ISL_548138, EPI_ISL_579066, EPI_ISL_579067, EPI_ISL_579077, EPI_ISL_579087, EPI_ISL_579092, EPI_ISL_579221, EPI_ISL_579426, EPI_ISL_579452, EPI_ISL_579496, EPI_ISL_622782, EPI_ISL_622795, EPI_ISL_622799, EPI_ISL_622827, EPI_ISL_682285, EPI_ISL_755622, EPI_ISL_843200, EPI_ISL_1082255, EPI_ISL_1904855, EPI_ISL_1967890, EPI_ISL_2406488, EPI_ISL_2811947, EPI_ISL_2811950, EPI_ISL_2811951, EPI_ISL_2811952, EPI_ISL_2811953, EPI_ISL_2811958, EPI_ISL_3477088, EPI_ISL_3477089, EPI_ISL_3477094, EPI_ISL_3543460, EPI_ISL_3709213, EPI_ISL_3760091			
see above	Canterbury Health Laboratories	Institute of Environmental Science and Research (ESR)	Anja Werno; Antje van der Linden; Arlo Upton; Chris Mansell; David Hammer; Dragana Drinkovic; Erasmus Smit; Gary McAuliffe; Hana Sofia Andersson; Hermes Perez; James Ussher; Jill Sherwood; Jing Wang; Joep de Ligt; Josh Freeman; Julia Howard; Juliet Elvy; Lauren Jelly; Mary DeAlmeida; Matt Blakiston; Matt Storey; Matthew Rogers; Max Bloomfield; Michael Addidle; Michelle Balm; Muhammad Faisal; Nikki Freed; Olin Silander; Olivia Stroeven; Paula scholes; Rachel Boyle; Sally Roberts; SallyAnn Harbison; Sarah Jefferies; Sharmini Muttaiyah; Susan Lin; Susan Morpeth; Susan Taylor; Timothy Blackmore; Vani Sathyendran; Veronica Playle; Virginia Hope; Xiaoyun Ren
EPI_ISL_1588904, EPI_ISL_1591265, EPI_ISL_2225271	Caribbean Public Health Agency	Carrington Lab, Department of PreClinical Sciences, Faculty of Medical Sciences, The University of the West Indies	Adesh Ramsubhag; Arianne Brown-Jordan; Avery Hinds; Chinna Chinnadurai; Christine V. F. Carrington; Christopher Oura; Gabriel Escobar; Jaya Jayaraman; Jerome Foster; Karla Georges; Marsha Ivey; Naresh Nandram; Narine Singh; Nikita S. D. Sahadeo; Nuno Faria; Oliver Pybus; Rahul Naidu; Rajini Haraksingh; Risha Singh; Roshan Parasram; Sarah Hill; Stanley Giddings; SueMin Nathaniel; Vernie Ramkissoon
EPI_ISL_2835622, EPI_ISL_2835635, EPI_ISL_2835640	Center for Medical and Sanitary Research (CERMES)	CDC Atlanta	Adamou Lagare; Dhwani Batra; Justin Lee
EPI_ISL_3505577, EPI_ISL_3505594, EPI_ISL_3505619, EPI_ISL_3505621	Center for Research on Health in Latin America	Institute of Microbiology, Universidad San Francisco de Quito	Belén Prado-Vivar; Bernardo Gutiérrez; Erika Muñoz; Fernanda Zurita; Gabriel Trueba; Jaime Costales; Juan José Guadalupe; Michelle Grunauer; Monica Becerra-Wong; Patricio Rojas-Silva; Paúl Cárdenas; Sully Márquez; Verónica Barragán
EPI_ISL_853886	Center for Virology, Medical University of Vienna	Bergthaler laboratory, CeMM Research Center for Molecular Medicine of the Austrian Academy of Sciences	Alexander Lercher; Alexandra Popa; Andreas Bergthaler; Anna Schedl; Benedikt Agerer; Christoph Bock; Jakob-Wendelin Genger; Jan Laine; Lukas Endler; Martin Senekowitsch; Michael Schuster; Thomas Penz
EPI_ISL_2232291, EPI_ISL_2232305, EPI_ISL_2232307, EPI_ISL_2380063, EPI_ISL_2380087, EPI_ISL_2380094	Center of Scientific Excellence for Influenza Viruses (CSEIV), National Research Centre	Center of Scientific Excellence for Influenza Viruses (CSEIV), National Research Centre	Ahmed E Kayed; Ahmed El-Taweel; Ahmed Kandeil; Ahmed Mostafa; Ghazi Kayali; Mahmoud Shehata; Mina Kamel; Mohamed Ahmed Ali; Mohamed El Sayes; Mokhtar Gomaa; Noura M Abo Shama; Omnia Kutkat; Rabeh El-Shesheny; Richard Webby; Sara Mahmoud; Yassmin Moatasim
EPI_ISL_539495	Centers for Disease Control and Prevention, Dengue Branch	Centers for Disease Control and Prevention, Dengue Branch	Betzabel Flores; Diego Sainz de la Peña; Gabriela Paz-Bailey; Gilberto A. Santiago; Glenda Gonzalez; Janice Perez; Jorge Bertran; Jorge L. Munoz-Jordan; Keyla Charriez; Vanessa Rivera-Amill
EPI_ISL_406031, EPI_ISL_3000790, EPI_ISL_3040149	Centers for Disease Control, R.O.C. (Taiwan)	Centers for Disease Control, R.O.C. (Taiwan)	Ji-Rong Yang; Jung-Jung Mu; Ming-Tsan Liu; Ming-Tsan-Liu; Shu-Ying Li; Yu-Chi Lin; Yu-Chi-Lin
EPI_ISL_815390	Centogene	Centogene	Krishna Kumar Kandaswamy; Peter Bauer; Vivi Hue-Trang Lieu
EPI_ISL_2612529	Centracare Laboratory Services	Minnesota Department of Health, Public Health Laboratory	Alexandra Lorentz; Jacob Garfin; Matt Plumb; and Xiong Wang
EPI_ISL_2834912, EPI_ISL_2834921	Central Health Laboratory	Central Health Laboratory ,Victoria Hospital, Candos,Ministry of Health and Wellness, Mauritius	Bahadoor BS; Jannoo N; Manraj SS; Mathur H; Ramuth M; Sonoo J; Sujeewon C
EPI_ISL_3217437	Central Health Laboratory	Virology Department, Central Health Laboratory ,Victoria Hospital, Candos,Ministry of Health and Wellness, Mauritius	Bahadoor BS; Jannoo N; Manraj SS; Mathur H; Ramuth M; Sonoo J; Sujeewon C
EPI_ISL_2834925	Central Health Laboratory/Airport Health Laboratory	Central Health Laboratory ,Victoria Hospital, Candos,Ministry of Health and Wellness, Mauritius	Bahadoor BS; Jannoo N; Manraj SS; Mathur H; Patoo M; Ramuth M; Sonoo J; Sujeewon C
EPI_ISL_2502407, EPI_ISL_2502413, EPI_ISL_2502416, EPI_ISL_2502429, EPI_ISL_2502430, EPI_ISL_2502441, EPI_ISL_2502479, EPI_ISL_2502480, EPI_ISL_2502493, EPI_ISL_2502494, EPI_ISL_2502500, EPI_ISL_2502527, EPI_ISL_2502545, EPI_ISL_2502553, EPI_ISL_2502575, EPI_ISL_3462539, EPI_ISL_3462553, EPI_ISL_3462560, EPI_ISL_3462561, EPI_ISL_3462578, EPI_ISL_3462580, EPI_ISL_3462582, EPI_ISL_3462608, EPI_ISL_3462609, EPI_ISL_3462659, EPI_ISL_3462679, EPI_ISL_3462683, EPI_ISL_3462693, EPI_ISL_3462700, EPI_ISL_3462704, EPI_ISL_3462707, EPI_ISL_3462723, EPI_ISL_3462724, EPI_ISL_3462725, EPI_ISL_3462726, EPI_ISL_3462741, EPI_ISL_3464728			
see above	Central Laboratory, Bureau of Public Health (BOG) and Academic Hospital Paramaribo	Erasmus Medical Center	Bas B Oude Munnink; Cherise Beek; Consuella Partowidjojo; Dion Gajadin; Ed PF IJzerman; Emmanuelle Munger; Gary Gummels; Ingrid SK Krishnadath; Lycke Woittiez; Marion PG Koopmans; Mireille Van de Veer; Phyllis Pinas; Princes Wongsowidjojo; Radjesh Ori; Ranisha Doerbalie; Rohma Banwari; Soeradj Harkisoen; Stephen Vreden; Tilotmadebie Ramlal; Verne Nanhoe
EPI_ISL_693475, EPI_ISL_693477, EPI_ISL_693478, EPI_ISL_693482, EPI_ISL_1307644, EPI_ISL_1307674, EPI_ISL_1322328, EPI_ISL_1322330			
see above	Central Public Health Laboratory	National Public Health Laboratory, National Centre for Infectious Diseases	Esorom Daoni; Lin Cui; Raymond Tzer Pin Lin; Sophie Octavia; Theresa Palou; Tze Minn Mak; Zhenyang Zhou
EPI_ISL_1068370	Central Public Health Laboratory - LACEN - Bahia, Salvador, Brazil	Central Public Health Laboratory - LACEN -Bahia, Salvador, Brazil	Arabela Leal; Breno Dominguez; Felicidade Pereira; Jaqueline Gomes; Luciana Oliveira; Luiz Alcantara; Marcela Gómez; Marta Giovanetti; Patrícia Cajado; Stephane Tosta; Vagner Fonseca; Vanessa Nardy
EPI_ISL_3045169, EPI_ISL_3045204	Central Public Health Laboratory, National Public Health Organization	Central Public Health Laboratory, National Public Health Organization	A.Katsoulidou et al; G.Spanakos; Kyriaki Tryfinopoulou
EPI_ISL_2232197	Central Public Health Laboratory, National Public Health Organization	Greek Genome Center, Biomedical Research Foundation of the Academy of Athens (BRFAA)	Dimitrios Thanos; Emmanouil Athanasiadis; Giannis Vatsellas; Katerina Zoi; Kyriaki Tryfinopoulou; Theodoros Loupis
EPI_ISL_2786923	Central Virology Laboratory, Ministry of Health	Central Virology Laboratory, Ministry of Health	Amos Adler; Efrat Bucris; Ella Mendelson; Michal Mandelboim; Moran Shwartswort-Cohen; Neta S. Zuckerman; Noam Protter; Oran Erster; Orna Mor; Saar Burstein
EPI_ISL_1312528	Centrala laboratorija	Latvian Biomedical Research and Study Centre	Davids Fridmanis; Guntars Zarins; Ivars Silamikelis; Jana Osite; Janis Klovins; Janis Pjalkovskis; Jurijs Perevoscikovs; Kaspars Megnis; Laila Silamikele; Lauma Freimane; Laura Ansone; Liga Birzniece; Marta Priedite; Monta Ustinova; Nikita Zrelovs; Uga Dumpis; Una Krumina; Vita Rovite
EPI_ISL_1760556	Centre Hospitalier Universitaire Mère-Enfant, Fondation Jeanne Ebori (CHUMEFJE)	Centre de recherches médicales de Lambaréné (CERMEL)	Ayola A. Adegnika; Ayong Moure; Bertrand Lell; Bénédicte Ndeboko; Emilio Skarwan; Georgelin Nguema Ondo; Gédéon P. Manouana; Haruka Abe; Jiro Yasuda; Joel Fleury Djoba Siawaya; Rodrigue Bikangui; Rotimi Myrabelle Avome Houechenou; Samira Zoa-Assoumou; Yuri Ushijima
EPI_ISL_1001053, EPI_ISL_1001065	Centre Pasteur du Cameroun	Institut Pasteur de Dakar	Njouom Richard
EPI_ISL_788934	Centre de Recherche et de Formation en Infectiologie Guinée	TransVIHMI, IRD/INSERM/Monpellier University	Abdoul Karim SOUMAH; Abdoulaye TOURE; Ahidjo AYOUBA; Alimou CAMARA; Alpha Kabinet KEITA; Bouna Yatassaye; Christelle BUTEL; Eric DELAPORTE; Jean-louis MONEMOU; Joel KOIVOGUI; Kaba KOUROUMA; Laetitia SERRANO; Mamadou Bhoye KEITA; Mamadou Saliou BAH; Mamadou Saliou SOW; Mandiou DIAKITE; Martine PEETERS; Moriba POVOGUI; Penda Malhado DIALLO; Sakoba KEITA
EPI_ISL_1913075, EPI_ISL_2434970, EPI_ISL_2434974, EPI_ISL_2434979,	Centre de Recherches Médicales de Lambaréné (CERMEL)	Centre de Recherches Médicales de Lambaréné (CERMEL)	Anicet Mouity Matoumba; Bertrand Lell and Ayola Akim Adegnika; Georgelin Nguema Ondo; Gédéon Prince Manouana; Jean Bernard Lekana-Douki; Joël-Fleury Djoba Siawaya; Michel Ngonga Dikongo; Moustapha Nzamba Maloum; Rodrigue Bikangui; Sam O’neilla Oye Bingono; Samira Zoa Assoumou; Srinivas reddy Pallerla; Steffen Borrmann; Thirumalaisamy P. Velavan

EPI_ISL_2442277, EPI_ISL_2442363			
EPI_ISL_2968062, EPI_ISL_2968067	Centre de Virologie des Maladies infectueuses Tropicales	Functional Genomic Platform UATRS-biology, CNRST	Ahmed REGGAD; Charifa DRISSI TOUZANI; Elmostafa BENAISSA; Elmostafa EL FAHIME; Hemlali Mouhssine; Hicham EL ANNAZ; Idriss-Amine LAHLOU; Khalid ENNIBI; Marouane MELLOUL; Mly Abdelaziz ELALAOUI; Mostafa ELOUENNASS; Nadia EL MRIMAR; Nadia TOUIL; Rachid ABI; Rida TAGAJDID; Safae ELKOCHRI; Sanaa ALAOUI-Amine; Youssef AKHOUAD; Zohour KASMY
EPI_ISL_428672	Centre for Dengue Research	Centre for Dengue Research	Ananda Wijewickrama; Chandima Jeewandara; Damayanthi Idampitiya; Deshni Jayathilaka; Dinuka Ariyaratne; Diyanath Ranasinghe; Eranga Narangoda; Laksiri Gomes; Neelika Malavige
EPI_ISL_792550, EPI_ISL_1717069, EPI_ISL_1717089, EPI_ISL_1717093, EPI_ISL_1970388, EPI_ISL_2481327, EPI_ISL_2803175, EPI_ISL_2803305			
see above	Centre for Dengue Research and AICBU, Department of Immunology and Molecular Medicine	Centre for Dengue Research and AICBU, Department of Immunology and Molecular Medicine	Chandima Jeewandara; Deshan Madhusanka; Deshni Jayathilaka; Dinuka Ariyaratne; Diyanath Ranasinghe; Gathsaurie Neelika Malavige; Laksiri Gomes; Tibutius Thanesh Pramanayagam
EPI_ISL_602566, EPI_ISL_668449	Centre for Dengue Research, Department of Immunology and Molecular Medicine	Centre for Dengue Research, Department of Immunology and Molecular Medicine	Chandima Jeewandara; Deshni Jayathilaka; Dinuka Ariyaratne; Diyanath Ranasinghe; Gathsaurie Neelika Malavige; Laksiri Gomes
EPI_ISL_413214, EPI_ISL_417030	Centre for Infectious Diseases and Microbiology Laboratory Services	NSW Health Pathology - Institute of Clinical Pathology and Medical Research; Westmead Hospital; University of Sydney	Carter I; Chen SC; Eden J-S; Holmes EC; Kok J and Dwyer DE for the 2019-nCoV Study Group*; Maddocks S; O'Sullivan MV; Rahman H; Rockett R; Sintchenko V
EPI_ISL_837554, EPI_ISL_837556, EPI_ISL_837566, EPI_ISL_837573, EPI_ISL_837576	Centro Nacional de Enfermedades Tropicales (CENETROP)	Laboratory of Respiratory Viruses and Measles, Oswaldo Cruz Institute, FIOCRUZ	Ana Carolina Mendonca; Anna Carolina Paixao; Cinthia Avila; Fernando Motta; Luciana Appolinario; Marilda Siqueira on behalf of the Fiocruz COVID-19 Genomic Surveillance Network; Paola Resende; Roxana Loayza
EPI_ISL_2274034	Centro Nacional de Enfermidades Tropicales (CENETROP)	Laboratory of Respiratory Viruses and Measles, Oswaldo Cruz Institute, FIOCRUZ	Alice Sampaio Rocha; Ana Carolina Mendonca; Anna Carolina Paixao; Cinthia Avila; Elisa Cavalcante Pereira; Fernando Motta; Luciana Appolinario; Marilda Siqueira on behalf of the Fiocruz COVID-19 Genomic Surveillance Network; Paola Resende; Renata Serrano Lopes; Roxana Loayza; Taina Venas
EPI_ISL_1531867, EPI_ISL_1531910	Centro de Diagnostico COVID-19 UABC Tijuana	Andersen lab at Scripps Research	German Ibarra; Jonathan Vincent Baena; Jorge Luis Jimenez Niebla; Manuel Sanchez Alavez; Oscar Efren Zazueta Fierro; SEARCH Alliance San Diego with Idanya Rubi Serafin Higuera
EPI_ISL_2339903	Centro de Estudio de Enfermedades Autoinmunes (CREA), Universidad del Rosario, Bogota, Colombia	Centro de Investigaciones en Microbiología y Biotecnología-UR (CIMBIUR), Facultad de Ciencias Naturales, Universidad del Rosario, Bogotá, Colombia	Carolina Ramírez-Santana; Gustavo Salguero; Juan David Ramírez; Juan Esteban Gallo; Juan-Manuel Anaya; Luz H. Patiño; Marina Muñoz; Nathalia Ballesteros; Sergio Castañeda
EPI_ISL_3825488	Centro de Estudios Moleculares	Laboratorio de Biología Molecular, Instituto de Medicina Regional on behalf of 'Proyecto Argentino Interinstitucional de genomica de SARS-CoV-2' (PAIS Consortium)	Alexia Del Puerto; Bettina Brusés; Cecilia Urquijo; Gerardo Deluca; Griselda Oria; Horacio Lucero.; Javier Mussin; Laura Formichelli; Melina Lorenzini Campos; Raúl Maximiliano Acevedo
EPI_ISL_3568785, EPI_ISL_3805311, EPI_ISL_3805316, EPI_ISL_3805324, EPI_ISL_3805361, EPI_ISL_3805364, EPI_ISL_3805367, EPI_ISL_3805413, EPI_ISL_3805760			
see above	Centro de Investigacion Biomedica de Occidente (CIBO)	Unidad de Genomica Avanzada	; Alejandra Garcia-Gasca; Alejandra Hernandez-Teran; Alejandro Sanchez-Flores; Alfredo Herrera-Estrella; Alicia OcaÃ±a-Mondragon; Alicia Ocaña-Mondragon; Andreu Comas-Garcia; Angel Gustavo Salas-Lais; Antonio Loza Roman; Bernardo Martinez-Miguel; Blanca Taboada; Brenda Irasema Maldonado-Meza; Bruno Gomez-Gil; Carla Ivon Herrera-Najera; Carlos F. Arias; Celia Boukadida; Celida Duque Molina; Celida Martinez- Rodriguez; Clara Esperanza Santacruz-Tinoco; Concepcion Grajales-MuÃ±iz; Concepcion Grajales-Muñiz; Consorcio Mexicano de Vigilancia Genomica (CoViGen-Mex). Authors (in alphabetical order): Julio Elias Alvarado-Yaah; Cristobal Chaidez-Quiroz; Daniel Fregoso-Rueda; Daniel Lira Morales; Eduardo Becerril-Vargas; Fernando Fontove-Herrera; Fidencio Mejia-Nepomuceno; Francisco Pulido; Gloria Elena Espinosa-Ayala; Gloria Maria Molina-Salinas; Gloria Vazquez; Hector Esteban Paz-Juarez; Hector Montoya-Fuentes; Helen Haydee Fernanda Ramirez-Plascencia; Irvin Gonzalez-Lopez; Jean Pierre Gonzalez; Jesus Hernandez; Joel Armando Vazquez-Perez.; Jorge Salas-Hernandez; Jose Antonio Enciso-Moreno; Jose Arturo Martinez-Orozco; Jose Esteban MuÃ±oz-Medina; Jose Esteban Muñoz-Medina; Jose de Jesus NuÃ±ez-Contreras; Jose de Jesus Nuñez-Contreras; Juan Bautista Chale-Dzul; Julissa Enciso-Ibarra; Luis Alberto Ochoa-Carrera; Margarita Matias-Florentino; Maria Guadalupe Santiago-Mauricio; Maria Guadalupe de Jesus Mireles-Rivera; Mario Mujica-Sanchez; Marissa Perez-Garcia; Nelly Selem-Mojica; Pavel Isa; Ricardo Ciria Merce; Ricardo Grande; Rosa Maria Gutierrez Rios; Santiago avila-Rios; Selene Zarate; Susana Lopez; Veronica Mata-Haro; Victor Eduardo Garcia-Arias; Victor Hugo Borja-Aburto
EPI_ISL_2671512, EPI_ISL_2942501, EPI_ISL_2942768, EPI_ISL_3805422, EPI_ISL_3805435, EPI_ISL_3805440, EPI_ISL_3805444, EPI_ISL_3805449			
see above	Centro de Investigacion Biomedica del Noreste (CIBIN)	Unidad de Genomica Avanzada	; Alejandra Garcia-Gasca; Alejandra Hernandez-Teran; Alejandro Sanchez-Flores; Alfredo Herrera-Estrella; Alicia Ocaña-Mondragon; Andreu Comas-Garcia; Angel Gustavo Salas-Lais; Antonio Loza Roman; Bernardo Martinez-Miguel; Blanca Taboada; Brenda Irasema Maldonado-Meza; Bruno Gomez-Gil; Carla Ivon Herrera-Najera; Carlos F. Arias; Celia Boukadida; Celida Duque Molina; Celida Martinez- Rodriguez; Clara Esperanza Santacruz-Tinoco; Concepcion Grajales-Muñiz; Consorcio Mexicano de Vigilancia Genomica (CoViGen-Mex). Authors (in alphabetical order): Julio Elias Alvarado-Yaah; Cristobal Chaidez-Quiroz; Daniel Fregoso-Rueda; Daniel Lira Morales; Eduardo Becerril-Vargas; Fernando Fontove-Herrera; Fidencio Mejia-Nepomuceno; Francisco Pulido; Gloria Elena Espinosa-Ayala; Gloria Maria Molina-Salinas; Gloria Vazquez; Hector Esteban Paz-Juarez; Hector Montoya-Fuentes; Helen Haydee Fernanda Ramirez-Plascencia; Irvin Gonzalez-Lopez; Jean Pierre Gonzalez; Jesus Hernandez; Joel Armando Vazquez-Perez.; Jorge Salas-Hernandez; Jose Antonio Enciso-Moreno; Jose Arturo Martinez-Orozco; Jose Esteban Muñoz-Medina; Jose de Jesus Nuñez-Contreras; Juan Bautista Chale-Dzul; Julissa Enciso-Ibarra; Luis Alberto Ochoa-Carrera; Margarita Matias-Florentino; Maria Guadalupe Santiago-Mauricio; Maria Guadalupe de Jesus Mireles-Rivera; Mario Mujica-Sanchez; Marissa Perez-Garcia; Nelly Selem-Mojica; Pavel Isa; Ricardo Ciria Merce; Ricardo Grande; Rosa Maria Gutierrez Rios; Santiago avila-Rios; Selene Zarate; Susana Lopez; Veronica Mata-Haro; Victor Eduardo Garcia-Arias; Victor Hugo Borja-Aburto
EPI_ISL_941127	Centro de Investigaciones en Microbiología y Biotecnología-UR (CIMBIUR), Facultad de Ciencias Naturales, Universidad del Rosario, Bogotá, Colombia	Centro de Investigaciones en Microbiología y Biotecnología-UR (CIMBIUR), Facultad de Ciencias Naturales, Universidad del Rosario, Bogotá, Colombia Icahn School of Medicine at Mount Sinai, New York, USA	Adriana van de Guchte; Alberto Paniz-Mondolfi; Alejandro Feged-Rivadeneira; Ana S. Gonzalez-Reiche; Andrés Angel; Carolina Flórez; Carolina Hernández; Emilia Mia Sordillo; Felipe González-Casabianca; Hala Alejel Alshammary; Harm van Bakel; Iván Carroll; Jaime Cascante; Jayeeta Dutta; Juan David Ramírez; Luz Helena Patiño; Marina Muñoz; Matthew M. Hernandez; Mauricio Santos-Vega; Mónica Palma-Cuero; Nathalia Ballesteros; Sergio Gomez; Viviana Simon; Zenab Khan
EPI_ISL_941946, EPI_ISL_941996	Centro de Investigaciones en Microbiología y Biotecnología-UR (CIMBIUR), Facultad de Ciencias Naturales, Universidad del Rosario, Bogotá, Colombia	Centro de Investigaciones en Microbiología y Biotecnología-UR (CIMBIUR), Facultad de Ciencias Naturales, Universidad del Rosario, Bogotá, Colombia Instituto Nacional de Salud, Bogotá, Colombia Icahn School of Medicine at Mount Sinai, New York, USA	Adriana van de Guchte; Alberto Paniz-Mondolfi; Ana S. Gonzalez-Reiche; Carolina Flórez; Carolina Hernández; Emilia Mia Sordillo; Hala Alejel Alshammary; Harm van Bakel; Jayeeta Dutta; Juan David Ramírez; Luz Helena Patiño; Marina Muñoz; Matthew M. Hernandez; Nathalia Ballesteros; Sergio Gomez; Viviana Simon; Zenab Khan
EPI_ISL_697797	Centro de Investigaciones, Universidad de Especialidades Espíritu Santo	Institute of Microbiology, Universidad San Francisco de Quito	Belén Prado-Vivar; Bernardo Gutiérrez; Derly Andrade; Edith Lopez; Fernando Espinoza; Gabriel Morey; Gabriel Trueba; Jose Pedro Barberan; Juan Carlos Fernandez; Juan José Guadalupe; Michelle Grunauer; Monica Becerra-Wong; Patricio Rojas-Silva; Paúl Cárdenas; Ruben Armas; Sully Márquez; Verónica Barragán
EPI_ISL_1351549	Centro de InvestigaciÃ³n BiomÃ©dica de Occidente (CIBO)	Unidad de Genomica Avanzada	Alejandro Sanchez-Flores; Alfredo Herrera-Estrella; Alicia Ocana-Mondragon; Angel Gustavo Salas-Lais; Bernardo Martinez-Miguel; Blanca Taboada; Brenda Irasema Maldonado-Meza; Carla Ivon Herrera-Najera; Carlos F. Arias; Celia Boukadida; Clara Esperanza Santacruz-Tinoco; Concepcion Grajales-Muniz; Consorcio Mexicano de Vigilancia Genomica (CoViGen-Mex). Authors (in alphabetical order): Julio Elias Alvarado-Yaah; Fernando Fontove-Herrera; Francisco Pulido; Gloria Elena Espinoza-Ayala; Gloria Maria Molina-Salinas; Gloria Vazquez; Hector Esteban Paz-Juarez; Hector Montoya-Fuentes; Helen Haydee Fernanda Ramirez-Plascencia; Jorge Ivan Salinal-Nevarez; Jose Antonio Enciso-Moreno; Jose Esteban Munoz-Medina; Jose de Jesus Nunez-Contreras; Juan Bautista Chale-Dzul; Luis Alberto Ochoa-Carrera; Margarita Matias-Florentino; Maria Guadalupe Santiago-Mauricio; Maria Guadalupe de Jesus Mireles-Rivera; Nelly Selem-Mojica; Pavel Isa; Ricardo Grande; Santiago Avila-Rios; Victor Hugo Borja-Aburto
EPI_ISL_1351426	Centro de InvestigaciÃ³n BiomÃ©dica del Noreste (CIBIN)	Unidad de Genomica Avanzada	Alejandro Sanchez-Flores; Alfredo Herrera-Estrella; Alicia Ocana-Mondragon; Angel Gustavo Salas-Lais; Bernardo Martinez-Miguel; Blanca Taboada; Brenda Irasema Maldonado-Meza; Carla Ivon Herrera-Najera; Carlos F. Arias; Celia Boukadida; Clara Esperanza Santacruz-Tinoco; Concepcion Grajales-Muniz; Consorcio Mexicano de Vigilancia Genomica (CoViGen-Mex). Authors (in alphabetical order): Julio Elias Alvarado-Yaah; Fernando Fontove-Herrera; Francisco Pulido; Gloria Elena Espinoza-Ayala; Gloria Maria Molina-Salinas; Gloria Vazquez; Hector Esteban Paz-Juarez; Hector Montoya-Fuentes; Helen Haydee Fernanda Ramirez-Plascencia; Jorge Ivan Salinal-Nevarez; Jose Antonio Enciso-Moreno; Jose Esteban Munoz-Medina; Jose de Jesus Nunez-Contreras; Juan Bautista Chale-Dzul; Luis Alberto Ochoa-Carrera; Margarita Matias-Florentino; Maria Guadalupe Santiago-Mauricio; Maria Guadalupe de Jesus Mireles-Rivera; Nelly Selem-Mojica; Pavel Isa; Ricardo Grande; Santiago Avila-Rios; Victor Hugo Borja-Aburto
EPI_ISL_732778	Centro de Investigación Biomédica de La Rioja - Hospital San Pedro Logroño	SeqCOVID-SPAIN consortium/IBV(CSIC)	José Manuel Azcona Gutiérrez; María Pilar Bea Escudero; María de Toro; Miriam Blasco Alberdi and SeqCOVID-SPAIN consortium
EPI_ISL_2490494	Centro de Investigación Biomédica de Occidente (CIBO)	Centro de Investigación en Enfermedades Infecciosas (CIENI), Instituto Nacional de Enfermedades Respiratorias (INER)	; Alejandra Garcia-Gasca; Alejandra Hernández-Terán; Alejandro Sanchez-Flores; Alfredo Herrera-Estrella; Alicia Ocaña-Mondragón; Andreu Comas-García; Angel Gustavo Salas-Lais; Antonio Loza Román; Bernardo Martínez-Miguel; Blanca Taboada; Brenda Irasema Maldonado-Meza; Bruno Gomez-Gil; Carla Ivón Herrera-Najera; Carlos F. Arias; Celia Boukadida; Clara Esperanza Santacruz-Tinoco; Concepción Grajales-Muñiz; Consorcio Mexicano de Vigilancia Genómica (CoViGen-Mex). Authors (in alphabetical order): Julio Elias Alvarado-Yaah; Cristóbal Cháidez-Quiróz; Célida Duque Molina; Célida Martínez- Rodríguez; Daniel Fregoso-Rueda; Daniel Lira Morales; Eduardo Becerril-Vargas; Fernando Fontove-Herrera; Fidencio Mejía-Nepomuceno; Francisco Pulido; Gloria Elena Espinosa-Ayala; Gloria María Molina-Salinas; Gloria Vazquez; Hector Esteban Paz-Juárez; Hector Montoya-Fuentes; Helen Haydee Fernanda Ramírez-Plascencia; Irvin González-López; Jean Pierre González; Joel Armando Vázquez-Pérez.; Jorge Salas-Hernández; José Antonio Enciso-Moreno; José Arturo Martínez-Orozco; José Esteban Muñoz-Medina; José de Jesús Nuñez-Contreras; Juan Bautista Chale-Dzul; Julissa Enciso-Ibarra; Luis Alberto Ochoa-Carrera; Margarita Matías-Florentino; Mario Mújica-Sánchez; Marissa Perez-Garcia; María Guadalupe Santiago-Mauricio; María Guadalupe de Jesús Mireles-Rivera; Nelly Sélem-Mojica; Pavel Isa; Ricardo Ciria Merce; Ricardo Grande; Rosa María Gutierrez Rios; Santiago Ávila-Ríos; Selene Zárate; Susana Lopez; Victor Eduardo Garcia-Arias; Víctor Hugo Borja-Aburto
EPI_ISL_2801708, EPI_ISL_2801721, EPI_ISL_2801737, EPI_ISL_2801746	Centro de Investigación Biomédica de Occidente (CIBO)	Instituto de Biotecnología de la UNAM	; Alejandra García-Gasca; Alejandra Hernández-Terán; Alejandro Sánchez-Flores; Alfredo Herrera-Estrella; Alicia Ocaña-Mondragón; Andreu Comas-García; Angel Gustavo Salas-Lais; Antonio Loza Román; Bernardo Martínez-Miguel; Blanca Taboada; Brenda Irasema Maldonado-Meza; Bruno Gómez-Gil; Carla Ivón Herrera-Najera; Carlos F. Arias; Celia Boukadida; Clara Esperanza Santacruz-Tinoco; Concepción Grajales-Muñiz; Consorcio Mexicano de Vigilancia Genómica (CoViGen-Mex). Authors (in alphabetical order): Julio Elias Alvarado-Yaah; Cristóbal Cháidez-Quiróz; Célida Duque Molina; Célida Martínez- Rodríguez; Daniel Fregoso-Rueda; Daniel Lira Morales; Eduardo Becerril-Vargas; Fernando Fontove-Herrera; Fidencio Mejía-Nepomuceno; Francisco Pulido; Gloria Elena Espinosa-Ayala; Gloria María Molina-Salinas; Gloria Vazquez; Hector Esteban Paz-Juárez; Hector Montoya-Fuentes; Helen Haydee Fernanda Ramírez-Plascencia; Irvin González-López; Jean Pierre González; Jesús Hernández; Joel Armando Vázquez-Pérez.; Jorge Salas-Hernández; José Antonio Enciso-Moreno; José Arturo Martínez-Orozco; José Esteban Muñoz-Medina; José de Jesús Nuñez-Contreras; Juan Bautista Chale-Dzul; Julissa Enciso-Ibarra; Luis Alberto Ochoa-Carrera; Margarita Matías-Florentino; Mario Mújica-Sánchez; Marissa Perez-Garcia; María Guadalupe Santiago-Mauricio; María Guadalupe de Jesús Mireles-Rivera; Nelly Sélem-Mojica; Pavel Isa; Ricardo Ciria Merce; Ricardo Grande; Rosa María Gutiérrez Rios; Santiago Ávila-Ríos; Selene Zárate; Susana Lopez; Verónica Mata-Haro; Victor Eduardo García-Arias; Víctor Hugo Borja-Aburto
EPI_ISL_2402174, EPI_ISL_2402178	Centro de Investigación Biomédica de Occidente (CIBO)	Unidad de Genomica Avanzada	Alejandro Sanchez-Flores; Alfredo Herrera-Estrella; Alicia Ocana-Mondragon; Angel Gustavo Salas-Lais; Bernardo Martinez-Miguel; Blanca Taboada; Brenda Irasema Maldonado-Meza; Carla Ivon Herrera-Najera; Carlos F. Arias; Celia Boukadida; Clara Esperanza Santacruz-Tinoco; Concepcion Grajales-Muniz; Consorcio Mexicano de Vigilancia Genomica (CoViGen-Mex). Authors (in alphabetical order): Julio Elias Alvarado-Yaah; Fernando Fontove-Herrera; Francisco Pulido; Gloria Elena Espinoza-Ayala; Gloria Maria Molina-Salinas; Gloria Vazquez; Hector Esteban Paz-Juarez; Hector Montoya-Fuentes; Helen Haydee Fernanda Ramirez-Plascencia; Jorge Ivan Salinal-Nevarez; Jose Antonio Enciso-Moreno; Jose Esteban Munoz-Medina; Jose de Jesus Nunez-Contreras; Juan Bautista Chale-Dzul; Luis Alberto Ochoa-Carrera; Margarita Matias-Florentino; Maria Guadalupe Santiago-Mauricio; Maria Guadalupe de Jesus Mireles-Rivera; Nelly Selem-Mojica; Pavel Isa; Ricardo Grande; Santiago Avila-Rios; Victor Hugo Borja-Aburto
EPI_ISL_2490340, EPI_ISL_2490442, EPI_ISL_3155408, EPI_ISL_3556953	Centro de Investigación Biomédica del Noreste (CIBIN)	Centro de Investigación en Enfermedades Infecciosas (CIENI), Instituto Nacional de Enfermedades Respiratorias (INER)	; Alejandra Garcia-Gasca; Alejandra García-Gasca; Alejandra Hernández-Terán; Alejandro Sanchez-Flores; Alejandro Sánchez-Flores; Alfredo Herrera-Estrella; Alicia Ocaña-Mondragón; Andreu Comas-García; Angel Gustavo Salas-Lais; Antonio Loza Román; Bernardo Martínez-Miguel; Blanca Taboada; Brenda Irasema Maldonado-Meza; Bruno Gomez-Gil; Bruno Gómez-Gil; Carla Ivón Herrera-Najera; Carlos F. Arias; Celia Boukadida; Clara Esperanza Santacruz-Tinoco; Concepción Grajales-Muñiz; Consorcio Mexicano de Vigilancia Genómica (CoViGen-Mex). Authors (in alphabetical order): Julio Elias Alvarado-Yaah; Cristóbal Cháidez-Quiróz; Célida Duque Molina; Célida Martínez- Rodríguez; Daniel Fregoso-Rueda; Daniel Lira Morales; Eduardo Becerril-Vargas; Fernando Fontove-Herrera; Fidencio Mejía-Nepomuceno; Francisco Pulido; Gloria Elena Espinosa-Ayala; Gloria María Molina-Salinas; Gloria Vazquez; Hector Esteban Paz-Juárez; Hector Montoya-Fuentes; Helen Haydee Fernanda Ramírez-Plascencia; Irvin González-López; Jean Pierre González; Jesús Hernández; Joel Armando Vázquez-Pérez.; Jorge Salas-Hernández; José Antonio Enciso-Moreno; José Arturo Martínez-Orozco; José Esteban Muñoz-Medina; José de Jesús Nuñez-Contreras; Juan Bautista Chale-Dzul; Julissa Enciso-Ibarra; Kathia Elizabeth Tapia-Diaz; Luis Alberto Ochoa-Carrera; Margarita Matías-Florentino; Mario Mújica-Sánchez; Marissa Perez-Garcia; María Guadalupe Santiago-Mauricio; María Guadalupe de Jesús Mireles-Rivera; Nelly Sélem-Mojica; Pavel Isa; Ricardo Ciria Merce; Ricardo Grande; Rosa María Gutierrez Rios; Rosa María Gutiérrez Rios; Santiago Ávila-Ríos; Selene Zárate; Susana Lopez; Verónica Mata-Haro; Victor Eduardo Garcia-Arias; Victor Eduardo García-Arias; Víctor Hugo Borja-Aburto
EPI_ISL_2801671, EPI_ISL_3347599, EPI_ISL_3347663, EPI_ISL_3347665	Centro de Investigación Biomédica del Noreste (CIBIN)	Instituto de Biotecnología de la UNAM	; Alejandra García-Gasca; Alejandra Hernández-Terán; Alejandro Sánchez-Flores; Alfredo Herrera-Estrella; Alicia Ocaña-Mondragón; Andreu Comas-García; Angel Gustavo Salas-Lais; Antonio Loza Román; Bernardo Martínez-Miguel; Blanca Taboada; Brenda Irasema Maldonado-Meza; Bruno Gómez-Gil; Carla Ivón Herrera-Najera; Carlos F. Arias; Celia Boukadida; Clara Esperanza Santacruz-Tinoco; Concepción Grajales-Muñiz; Consorcio Mexicano de Vigilancia Genómica (CoViGen-Mex). Authors (in alphabetical order): Julio Elias Alvarado-Yaah; Cristóbal Cháidez-Quiróz; Célida Duque Molina; Célida Martínez- Rodríguez; Daniel Fregoso-Rueda; Daniel Lira Morales; Eduardo Becerril-Vargas; Fernando Fontove-Herrera; Fidencio Mejía-Nepomuceno; Francisco Pulido; Gloria Elena Espinosa-Ayala; Gloria María Molina-Salinas; Gloria Vazquez; Hector Esteban Paz-Juárez; Hector Montoya-Fuentes; Helen Haydee Fernanda Ramírez-Plascencia; Irvin González-López; Jean Pierre González; Jesús Hernández; Joel Armando Vázquez-Pérez).; Joel Armando Vázquez-Pérez.; Jorge Salas-Hernández; José Antonio Enciso-Moreno; José Arturo Martínez-Orozco; José Esteban Muñoz-Medina; José de Jesús Nuñez-Contreras; Juan Bautista Chale-Dzul; Julissa Enciso-Ibarra; Kathia Elizabeth Tapia-Diaz; Luis Alberto Ochoa-Carrera; Margarita Matías-Florentino; Mario Mújica-Sánchez; Marissa Perez-Garcia; María Guadalupe Santiago-Mauricio; María Guadalupe de Jesús Mireles-Rivera; Nelly Sélem-Mojica; Pavel Isa; Ricardo Ciria Merce; Ricardo Grande; Rosa María Gutiérrez Rios; Santiago Ávila-Ríos; Selene Zárate; Susana Lopez; Verónica Mata-Haro; Victor Eduardo García-Arias; Víctor Hugo Borja-Aburto
EPI_ISL_2401935, EPI_ISL_2402005	Centro de Investigación Biomédica del Noreste (CIBIN)	Unidad de Genomica Avanzada	Alejandro Sanchez-Flores; Alfredo Herrera-Estrella; Alicia Ocana-Mondragon; Angel Gustavo Salas-Lais; Bernardo Martinez-Miguel; Blanca Taboada; Brenda Irasema Maldonado-Meza; Carla Ivon Herrera-Najera; Carlos F. Arias; Celia Boukadida; Clara Esperanza Santacruz-Tinoco; Concepcion Grajales-Muniz; Consorcio Mexicano de Vigilancia Genomica (CoViGen-Mex). Authors (in alphabetical order): Julio Elias Alvarado-Yaah; Fernando Fontove-Herrera; Francisco Pulido; Gloria Elena Espinoza-Ayala; Gloria Maria Molina-Salinas; Gloria Vazquez; Hector Esteban

			Paz-Juarez; Hector Montoya-Fuentes; Helen Haydee Fernanda Ramirez-Plascencia; Jorge Ivan Salinal-Nevarez; Jose Antonio Enciso-Moreno; Jose Esteban Munoz-Medina; Jose de Jesus Nunez-Contreras; Juan Bautista Chale-Dzul; Luis Alberto Ochoa-Carrera; Margarita Matias-Florentino; Maria Guadalupe Santiago-Mauricio; Maria Guadalupe de Jesus Mireles-Rivera; Nelly Selem-Mojica; Pavel Isa; Ricardo Grande; Santiago Avila-Rios; Victor Hugo Borja-Aburto
EPI_ISL_2442109	Centro de Investigação em Saúde de Manhiça (CISM) & ISGlobal, Institut de Salut Global de Barcelona	Instituto de Biomedicina de València - CSIC	Alfredo Mayor; Arsenia Massinga; Inacio Mandomando; Irving Cancino; Iñaki Comas; Manoli Torres Puente; Santiago Jiménez-Serrano
EPI_ISL_1396492, EPI_ISL_1396493	Centro de Tecnología en Salud Pública de la Universidad Nacional de Rosario	Laboratorio Mixto de Biotecnología Acuática (LMBA) on behalf of 'Proyecto Argentino Interinstitucional de genomica de SARS-CoV-2' (PAIS Consortium)	Adriana Giri; Agustina Cerri; Ana Cavatorta; Ana Paletta; Diego Chouhy; Elisa Bolatti; Elizabeth Tapia (argenTAG); Federico Remes Lenicov; Flavio Spetale; Gastón Viarengo; Ignacio García Labari; Javier Murillo; Joaquín Ezpeleta; Julian Acosta; Laura Angelone; Leandro Ciappina; María Re; Pablo Casal; Pilar Bulacio; Silvana Spinelli; Silvia Arranz; Sofía Lavista Llanos; Vanina Villanova; Victoria Posner
EPI_ISL_2798497, EPI_ISL_2798902	Centrālā Laboratorija, SIA	Riga East University Hospital, National Microbiology Reference Laboratory; Eurofins Genomics Europe Sequencing GmbH	Arzu Algulieva; Diāna Dušacka; Dārta Pūpola; Ilva Pole; Jana Osīte; Jevgenijs Bodrenko; Jūlija Čevere; Reinis Vangravs; Reinis Zeltmatis; Sergejs Ņikišins; Stella Lapiņa; Ģirts Šķenders
EPI_ISL_2646234	Cerballiance-IDF	Cerba lab	Aude Lessenne; Bénédicte Roquebert; Emmanuel Lecorche; Kader Merah; Laura Verdurme; Patrice Herisson; Sabine Trombert-Paolantoni; Stéphanie Haïm-Boukobza; Thierry Collin
EPI_ISL_753879, EPI_ISL_754192	Charité Universitätsmedizin Berlin, Institut für Virologie/Labor Berlin	Charité Universitätsmedizin Berlin, Institut für Virologie	Barbara Mühlemann; Christian Drosten; Julia Schneider; Jörn Beheim-Schwarzbach; Talitha Veith; Terry Jones; Victor M Corman
EPI_ISL_3191217	Chiba Prefectural Institute of Public Health	Division of Virology and Zoology, Chiba Prefectural Institute of Public Health	Akihiro Takemura; Haruna Nishijima; Kentaro Itokawa; Makoto Kuroda; Mari Ohta; Masakatsu Taira; Masanori Hashino; Mika Takeuchi; Rina Tanaka; Shigenori Sato; Tsuyoshi Sekizuka; Yuji Hanada; Yuki Fujinuma
EPI_ISL_1931972	Chiba Prefectural Institute of Public Health	Pathogen Genomics Center, National Institute of Infectious Diseases	Kentaro Itokawa; Makoto Kuroda; Masanori Hashino; Rina Tanaka; Tsuyoshi Sekizuka
EPI_ISL_700333	Child Health Research Foundation	Child Health Research Foundation	Afroza Akter Tanni; Arif Mohammad Tanmoy; Md Hafizur Rahman; Roly Malaker; Samir K Saha; Senjuti Saha; Sharmistha Goswami; Syed Muktadir Al Sium
EPI_ISL_2445989, EPI_ISL_2445997	Chittagong General Hospital	Child Health Research Foundation	Adnan Mannan; CHRF Bangladesh Genomics Team; H. M. Hamidullah Mehedi; Md. Abdur Rob; Md. Minhazul Hoque; Rajdeep Biswas
EPI_ISL_3118694	Chris Hani Baragwanath Academic Hospital POC laboratory	KRISP, KZn Research Innovation and Sequencing Platform	Brown MJ; Emmanuel SJ; Giandhari J; Lessells R; Omar S; Pillay S; Tegally H; Wadula J; Wilkinson E; Yajna R; de Oliveira T
EPI_ISL_2433537	Chulabhorn Hospital	Division of Genomic Medicine and Innovation support,Department of Medical Sciences, Ministry of Public Health, Thailand	Archawin Rojanawiwat; Jirapha Pakdee; Natthakul Bunneang; Nuanjun Wichukchinda; Penpitcha Thawong; Pilailuk Akkapaiboon Okada; Pundharika Piboonsiri; Surakameth Mahasirimongkol; Waritta Sawaengdee
EPI_ISL_2523839	City Hospital No 40	WHO National Influenza Centre Russian Federation	Andrey Komissarov; Artem Fadeev; Daria Danilenko; Dmitry Lioznov; Elena Nabieva; Georgii Bazykin; Kirill Varchenko; Ksenia Safina; Kseniya Komissarova; Maria Pisareva; Maria Timofeeva; Mikhail Bakaev; Nikita Yolshin; Olga Shneider; Oula Mansour; Sergey Scherbak; Tamila Musaeva; Veronika Eder
EPI_ISL_636981	City of Chimoio	KRISP, KZN Research Innovation and Sequencing Platform	Giandhari J; Ismael N; Nadia Sitoe; Nedio Mabunda; Paulo Arnaldo; Pillay S; Tegally H; Wilkinson E; de Oliveira T
EPI_ISL_1250842	Clin & Gen Lab	Molecular Genetics Laboratory, Instituto de Investigaciones Químicas, Universidad Mayor de San Andrés	Aneth Vasquez Michel; Oscar M. Rollano-Peñaloza
EPI_ISL_3545676	Clinic of infective diseases	Laboratory of virology and molecular diagnostics, Institute of Public Health	Boshevska G; Buzharova T; Janchevska E.; Kuzmanovska M
EPI_ISL_2467214, EPI_ISL_2490303, EPI_ISL_3242537	Clinica INDISA	"Facultad de Ciencias de la Vida, UNAB"	"Claudio Meneses; Ariel Orellana"; Claudio Olmos; Daniel Leon; Dayan Sanhueza; Eduardo Castro; Gonzalo Campaña; Macarena Bastias; Paola Pidal; Ricardo Yusta; Sebastian Wolter; Susana Saez; Víctor Monreal; Waldo Diaz
EPI_ISL_1300656, EPI_ISL_1300657, EPI_ISL_2938118	Clinical Center, University of Sarajevo; Unit for Clinical Microbiology	Clinical Center, University of Sarajevo; Unit for Clinical Microbiology	Amela Dedeić-Ljubović; Edina Zahirović; Golubinka Boshevska; Irma Salimović-Bešić; Maja Kuzmanovska; Sandra Vegar-Zubović; Sebija Izetbegović; Suzana Arapčić
EPI_ISL_911927	Clinical Diagnostics Laboratory, Diagnostic & Experimental Pathology, Lilly Research Laboratories	Clinical Diagnostics Laboratory, Diagnostic & Experimental Pathology, Lilly Research Laboratories	Andrew Schade; Angie Fulford; Erin Wray; Jeff Fill; Joe Oakley; John Calley; John McElwee; Leslie ONeill Reising; Mayuri Vaidya; Pat Finnegan; Phil Ebert; Rachael Redmond; Sam McNeely; Tim Holzer
EPI_ISL_3832905	Clinical Lab, Aga Khan University Hospital	Infectious Diseases Research Laboratory, Department of Pediatrics and Child Health	Fatima Aziz; Furqan Kabir; Fyezah Jehan; Imran Nisar; Samiah Kanwar; Waqasuddin Khan; Zahra Hasan
EPI_ISL_3799753, EPI_ISL_3799757, EPI_ISL_3799772	Clinical Microbiology Lab, King Abdulaziz Medical City, Ministry of National Guard – Health Affairs, Riyadh, Saudi Arabia	Infectious Diseases Research, King Abdullah International Medical Research Center (KAIMRC), Riyadh, Saudi Arabia	A.A.; Alghoribi; Alhayli, S.; Aljohani; Alswaji; Bu Ali, Z.; M.F.; Okdah, L.; S.M. and Doumith, M.
EPI_ISL_3534520	Clinical Microbiology Laboratory, Faculty of Medicine, Universitas Indonesia	Faculty of Medicine, Universitas Indonesia	Andi Yasmon; Anis Karuniawati; Ari Fahrial Syam; Augustine Natasha; Badriul Hegar; Beti Ernawati; Budi Wiweko; Fadilah; Fera Ibrahim; Fithriyah; Khaerunissa Anbar Istiadi; Linda Erlina; Pratiwi Sudarmono; Rafika Indah Paramita
EPI_ISL_3155137	Clinical Microbiology, Infection Prevention and Control	Section for Molecular Diagnostics	Björn Hallström; Jonas Björkman
EPI_ISL_2535722	Clinical Research Centre Hospital Sibu	Institute of Health and Community Medicine	Chan Chia Jui; Chua Hock Hin; David Perera; Ooi Mong How; Tonnii Sia Loong Loong; Wong Jyn Shan; Wong Kiing Aik
EPI_ISL_582000, EPI_ISL_830822	Clinical Virology	Clinical Bacteriology	Adrian Egli; Alexander Gensch; Alfredo Mari; Christian Nickel; Hans Hirsch; Hans Pargger; Helena MB Seth-Smith; Julia Bielicki; Karoline Leuzinger; Kirstine K. Soegaard; Madlen Stange; Manuel Battegay; Martin Siegemund; Michael Osthoff; Michael Schweitzer; Myrta Brunner; Rita Schneider-SliFemalea; Rita Schneider-Sliwa; Roland Bingisser; Sarah Tschudin-Sutter; Simon Fuchs; Stefano Bassetti; Tim Roloff
EPI_ISL_2035753	Clinical Virology	Clinical Virology	Anissa Chouikha; Henda Triki; Kais Ghedira; Mariem Gdoura; Sondos Haddad; Wasfi Fares
EPI_ISL_3276958	Clinical Virology Laboratory, Institute of Liver and Biliary Sciences	ILBS	Amit Pandey; Chhagan Bihari Sharma; Diptanu Paul; Ekta Gupta; Reshu Agarwal; Shiv Kumar Sarin; Varun Suroliya
EPI_ISL_1169735, EPI_ISL_2194558, EPI_ISL_2311053, EPI_ISL_3842885	Colorado Department of Public Health and Environment	Colorado Department of Public Health and Environment	Alexandria Rossheim; Diana Ir; Emily A. Travanty; Laura Bankers; Mandy Waters; Michael Martin; Molly C. Hetherington-Rauth; Sarah Elizabeth Totten; Shannon Ely; Shannon R. Matzinger
EPI_ISL_3127898, EPI_ISL_3303926	Commonwealth Healthcare Center	Centers for Disease Control and Prevention Division of Viral Diseases, Pathogen Discovery	Alex Burgin; Ben L. Rambo-Martin; Clinton R. Paden; Dakota Howard; Dave Wentworth; Dhwani Batra; Jasmine Padilla; Justin Lee; Krista Queen; Kristen Knipe; Kristine Lacek; Mark Burroughs; Matthew Schmerer; Meghan Bentz; Mili Sheth; Peter Cook; Sam Shepard; Sarah Nobles; Suxiang Tong; Vivien Dugan; Yvette Unoarumhi
EPI_ISL_632262, EPI_ISL_632272, EPI_ISL_682301, EPI_ISL_1660437, EPI_ISL_1660464, EPI_ISL_2622085, EPI_ISL_2715288, EPI_ISL_2812555, EPI_ISL_2932621, EPI_ISL_2992105, EPI_ISL_3568653, EPI_ISL_3663309			
see above	Communicable Disease Laboratory, Public Health Directorate	Communicable Disease Laboratory, Public Health Directorate	AlAbbas, Z.; AlHujairi, Z.; AlTaif, Z.; AlWasti, H.; Almoamen, G.; Altaif, Z.; Alwasti, H.; Marhoon, A.; Touq, M.
EPI_ISL_2562567	Creighton University Medical Center	Creighton COVID Consortium	Cynthia Watson; Holly Stessman; Jake Siedlik; Michael Belshan; Richard Goering
EPI_ISL_3105882	Cruz-Rabe Maternity and General Hospital	Research Institute for Tropical Medicine	Clyde Dapat; Deana Mae Ocampo; Emmanuel Kagning Tsinda; Francisco Gerardo Polotan; Hitoshi Oshitani; Inez Andrea Medado; Jefferson Earl Halog; Joana Ina Manalo; Lei Lanna Dancel; Ma Angelica Tujan; Mariko Saito-Obata; Mayuko Saito; Michiko Okamoto; Samantha Louise Bado
EPI_ISL_2784152	Curative Labs	Curative Labs	Elias L. Salfati; Eugenia Khorosheva; George Way; J.Cesar Ignacio-Espinoza; Janet Chen; Mikhail Hanewich-Hollatz; Nabjot Sandhu; Sophia Quasem; Vladimir Slepnev; Zhiyi Xie
EPI_ISL_3664264	DASA	DASA	Adriano Bonaldi; Angelica Hristov; Annelise Lopes; Bianca Cota; Cristina Oliveira; Jose Levi; Lidia Yamamoto; Paulo Pierry; Rodrigo Guarischi; Rodrigo Salazar
EPI_ISL_2383998	DC Public Health Lab/ Dept. of Forensic Sciences	Centers for Disease Control and Prevention Division of Viral Diseases, Pathogen Discovery	Alison Laufer Halpin; Ben L. Rambo-Martin; Clinton R. Paden; Dakota Howard; Darlene Wagner; Dave Wentworth; Dhwani Batra; Jasmine Padilla; Justin Lee; Katie Dillon; Krista Queen; Kristen Knipe; Kristine Lacek; Mark Burroughs; Matthew Schmerer; Mili Sheth; Peter Cook; Sam Shepard; Sarah Nobles; Shoshona Le; Suxiang Tong; Vivien Dugan; Yvette Unoarumhi
EPI_ISL_2176242, EPI_ISL_2176257, EPI_ISL_3031753	DC Public Health Lab/ Dept. of Forensic Sciences	DC Public Health Lab/ Dept. of Forensic Sciences	Brittany Hamilton; Connie Maza; David Payne; Elizabeth Zelaya; Janis Doss; Jocelyn Hauser; Monica Mann; Sarah Scott; Scott Nguyen
EPI_ISL_2840443	DE Public Health Laboratory	Centers for Disease Control and Prevention Division of Viral Diseases, Pathogen Discovery	Alison Laufer Halpin; Ben L. Rambo-Martin; Clinton R. Paden; Dakota Howard; Darlene Wagner; Dave Wentworth; Dhwani Batra; Jasmine Padilla; Justin Lee; Katie Dillon; Krista Queen; Kristen Knipe; Kristine Lacek; Mark Burroughs; Matthew Schmerer; Mili Sheth; Peter Cook; Sam Shepard; Sarah Nobles; Shoshona Le; Suxiang Tong; Vivien Dugan; Yvette Unoarumhi
EPI_ISL_2983222, EPI_ISL_2983224, EPI_ISL_2987211, EPI_ISL_2987214	DNA Labs	National Reference Laboratory, Nigeria Centre for Disease Control	Anthony Ahumibe; Catherine Okoi; Chimaobi Chukwu; Dr Chikwe Ihekweazu; Dr Ndodo Nnaemeka; Dr Omoare Adesuyi; Nwando Mba; Olusola Anuoluwapo Akanbi
EPI_ISL_3216489	DOHMH Corona	New York City Public Health Laboratory	Jade Wang; et al.
EPI_ISL_2383735, EPI_ISL_3692737	DPHL	Delaware Public Health Lab	Rebecca Savage
EPI_ISL_2595860, EPI_ISL_2596803, EPI_ISL_2597312, EPI_ISL_2612300	DSMRC	DSMRC	Khine Zaw Oo; Ko Ko Lwin; Ko Ko Win; Kyee Myint; Nay Myo Aung; Pai Khant Kyaw; Phyo Kyaw Aung; Thet Wai Oo; Zaw Win Htun
EPI_ISL_416542	Dasman Diabetes Institute	Dasman Diabetes Institute	Ebaa AlOzairi; Fahd Al-Mulla; Motasem Melhem; Qais Al-Duwairi; Rasheeba Iqbal; Sara Al-Qabandi; Sumi John

EPI_ISL_2648218	Debswana Orapa Mine Hospital Laboratory	Botswana Harvard HIV Reference Laboratory	Boitumelo Zuze; Botshelo Radibe; Dorcas Maruapula; Joseph Makhema; Keoratile Ntshambiwa; Koketso Maotwe; Legodile Kooepile; Lesedi Magama; Madisa Mine; Mosepele Mosepele; Mpo Molapisi; Ontlametse T. Bareng; Roger Shapiro; Shahin Lockman; Sikhulile Moyo; Simani Gaseitsiwe; Thongbotho Mphoyakgosi; Wonderful T. Choga
EPI_ISL_476894	Defence Research & Development Establishment (DRDE)	Defence Research & Development Establishment (DRDE)	Ambuj Shrivastava; Jyoti S. Kumar; Paban Kumar Dash; Shashi Sharma; Sushil Kumar Sharma
EPI_ISL_693707	Delaware Public Health Laboratory	Delaware Public Health Laboratory	Gregory Hovan
EPI_ISL_2432953	Demy Health	National Reference Laboratory, Nigeria Centre for Disease Control	Anthony Ahumibe; Catherine Okoi; Chimaobi Chukwu; Dr Chikwe Ihekweazu; Dr Ndodo Nnaemeka; Dr Omoare Adesuyi; Grace Esebanmen; Naidoo Dhamari; Nwando Mba; Olusola Anuoluwapo Akanbi
EPI_ISL_3631654	DemyHealth Laboratory	National Reference Laboratory, Nigeria Centre for Disease Control	Anthony Ahumibe; Catherine Okoi; Celestina Obiekea; Chimaobi Chukwu; Dr Chikwe Ihekweazu; Dr Ndodo Nnaemeka; Dr Omoare Adesuyi; Nwando Mba; Olusola Anuoluwapo Akanbi
EPI_ISL_1340751, EPI_ISL_1340753, EPI_ISL_1340755, EPI_ISL_1340758, EPI_ISL_1340761, EPI_ISL_1340764	Departamento de Virologia, Laboratorio Central de Salud Pública, Avenida Venezuela y Teniente Escurra, Asunción, Paraguay	Laboratory of Respiratory Viruses and Measles, Oswaldo Cruz Institute, FIOCRUZ	Alice Sampaio Rocha; Ana Carolina Mendonca; Anna Carolina Paixao; Cynthia Vazquez; Fernando Motta; Luciana Appolinario; Marilda Siqueira on behalf of the Fiocruz COVID-19 Genomic Surveillance Network; Paola Resende; Renata Serrano Lopes
EPI_ISL_516927	Department for Molecular Diagnostics, Centre for Medical Microbiology, Institute of Public Health of Montenegro	Charité Universitätsmedizin Berlin, Institut für Virologie	Barbara Muehlemann; Christian Drosten; Julia Schneider; Jörn Beheim-Schwarzbach; Marija Govedarica and Danijela Vujošević; Talitha Veith; Terry Jones; Victor M Corman
EPI_ISL_471529, EPI_ISL_754180, EPI_ISL_1914599, EPI_ISL_1914784, EPI_ISL_1914785, EPI_ISL_3076898, EPI_ISL_3076933, EPI_ISL_3098700, EPI_ISL_3204249, EPI_ISL_3204255, EPI_ISL_3204260, EPI_ISL_3208827, EPI_ISL_3209246			
see above	Department for Virology, Molecular Biology and Genome Research, R. G. Lugar Center for Public Health Research, National Center for Disease Control and Public Health (NCDC) of Georgia.	Department for Virology, Molecular Biology and Genome Research, R. G. Lugar Center for Public Health Research, National Center for Disease Control and Public Health (NCDC) of Georgia.	Adam Kotorashvili; Amiran Gamkrelidze.; Ana Papkiauri; Ann Machablishvili; Anna Kasradze; Davit Tsaguria; Ekaterine Khmaladze; Ekaterine Zangaladze; Ekaterine Zhghenti; Giorgi Gogoladze; Giorgi Tomashvili; Gvantsa Brachveli; Gvantsa Chanturia; Irma Burjanadze; Ketevan Sidamonidze; Khatuna Zakhashvili; Lela Sabadze; Lela Urushadze; Magda Dgebuadze; Maia Alkhazashvili; Mari Gavashelidze; Mariam Zakalashvili; Marine Murtskhvaladze; Meri Pantsulaia; Nato Kotaria; Nino Berishvili; Nino Chikhovani; Paata Imnadze; Roena Sukhiashvili; Salome Javashvili; Tamar Jashiashvili; Tata Imnadze; Tea Tevdoradze
EPI_ISL_2723564, EPI_ISL_2840859, EPI_ISL_2863931	Department of Acute Infectious Diseases Control and Prevention, Yunnan Provincial Center for Disease Control and Prevention	Department of Acute Infectious Diseases Control and Prevention, Yunnan Provincial Center for Disease Control and Prevention	Jienan Zhou; Meiling Zhang; Senquan Jia; Xiaoqing Fu; Zhaosheng Liu
EPI_ISL_2666982, EPI_ISL_2668207, EPI_ISL_2669198, EPI_ISL_2744063, EPI_ISL_2992629, EPI_ISL_3103998, EPI_ISL_3141987, EPI_ISL_3530600, EPI_ISL_3531615, EPI_ISL_3806268			
see above	Department of Bacteria, Parasites and Fungi, Statens Serum Institut, Copenhagen, Denmark	Statens Serum Institut Bioinformatics and Microbial Genomics	Danish Covid-19 Genome Consortium
EPI_ISL_2483896	Department of Clinical Microbiology	GIGA Medical Genomics	Bouchra Boujemla; Cécile Meex; Keith Durkin; Maria Artesi; Marie-Pierre Hayette; Nathalie Renotte; Pierrette Melin; Raphaël Boreux; Sébastien Bontems; Vincent Bours
EPI_ISL_452074	Department of Clinical Microbiology, Copenhagen University Hospital, Hvidovre, Kettegaard Alle 30, 2650 Hvidovre.	Albertsen lab, Department of Chemistry and Bioscience, Aalborg University, Denmark	Rasmus Kirkegaard
EPI_ISL_419250	Department of Clinical Pathology, Pamela Youde Nethersole Eastern Hospital	Department of Health Technology and Informatics, Faculty of Health and Social Science, The Hong Kong Polytechnic University	Alan Ka-Lun WU; Alex Yat-Man HO; Barry Kin-Chung WONG; David Ho-Keung SHUM; Eugene Yuk-Keung TSO; Gilman Kit-Hang SIU; Hiu-Yin LAO; Kam-Tong YIP; Kenneth Siu-Sing LEUNG; Kingsley King-Gee TAM; Kit-Man SIN; Kitty Sau-Chun FUNG; Kwok-Cheung LUNG; Lam-Kwong LEE; Man-Chun CHAN; Ming-Pan CHOI; Miranda Chong-Yee YAU; Raymond Wai-To LIU; Sandy Ka-Yee CHAU; Shea Ping YIP; Tak-Lun QUE; Timothy Ting-Leung NG; Wai-Shing LEUNG; Wing Cheong YAM; Wing-Kin TO; Yuk-Yung NG
EPI_ISL_2378703, EPI_ISL_2378704	Department of Health Technology and Informatics, The Hong Kong Polytechnic University	Department of Health Technology and Informatics, The Hong Kong Polytechnic University	A.K.-L.; A.Y.-M.; B.H.-S.; B.P.-H.; C.T.-M.; Chan; Chen, H.; Cheng; D.C.; D.S.-H.; E.Y.-K.; E.Y.-W.; G.K.-H.; H.-C.; H.-Y.; H.W.-H.; Ho; J.S.-L.; J.Y.-W.; Jim; K.-Y.; K.K.; K.K.-G.; K.K.-S.; K.K.-W.; K.N.; K.S.-S.; L.-K.; Lai; Lam; Lao; Lee; Leung; Lo; Lu; Luk, S.; Lung; M.-C.; M.C.-Y.; Mok; Ng; R.A.; S.-C.; Siu; T.T.-L.; Tam; To; Tse, H.; V.C.-C.; W.-K.; W.-S.; Wong; Wu; Y.W.-M.; Yau; Yeh; Yuen
EPI_ISL_2713174, EPI_ISL_2713218, EPI_ISL_2713220, EPI_ISL_2713266, EPI_ISL_3105863	Department of Health Technology and Informatics, The Hong Kong Polytechnic University	Department of Health Technology and Informatics, The Hong Kong Polytechnic University	Alan Ka-Lun Wu; Alex Yat-Man Ho; Barry Kin-Chung Wong; Chloe Toi-Mei Chan; David Ho-Keung Shum; Denise Sze-Hang Wong; Gilman Kit-Hang Siu; Hiu-Yin Lao; Hoi-Ching Jim; Ivan Tak-Fai Wong; Jake Siu-Lun Leung; Kam-Tong Yip; Kenneth Siu-Sing Leung; Kingsley King-Gee Tam; Kitty Sau-Chun Fung; Kristine Luk; Lam-Kwong Lee; Miranda Chong-Yee Yau; Sandy Ka-Yee Chau; Shea Ping Yip; Tak-Lun Que; Timothy Ting-Leung Ng; Wing Cheong Yam; Wing-Hei Lo; Wing-Kin To; Yvette Wai-Man Lai
EPI_ISL_417444	Department of Healthcare Biotechnology, National University of Sciences and Technology (NUST)	Department of Healthcare Biotechnology, National University of Sciences and Technology (NUST)	Corman; Ghani, E.; H.A.; Janjua; Javed, A.; Niazi; S.K.; Saqib, M.; V.M. and Zohaib, A.
EPI_ISL_1007671	Department of Internal Medicine, College of Medicine, Chosun University	Department of Internal Medicine, College of Medicine, Chosun University	Dong-Min Kim
EPI_ISL_1495831	Department of Laboratory Medicine, Division of Clinical Virology, University of Medicine, Vienna	Bergthaler laboratory, CeMM Research Center for Molecular Medicine of the Austrian Academy of Sciences	Andreas Bergthaler; Anna Schedl; Bekir Erguner; Benedikt Agerer; Christoph Bock; Fabian Amman; Jan Laine; Lukas Endler; Maelle Le Moing; Martin Senekowitsch; Michael Schuster; Petr Triska; Thomas Penz
EPI_ISL_538462, EPI_ISL_648670	Department of Laboratory Medicine, Tan Tock Seng Hospital	Department of Laboratory Medicine, Tan Tock Seng Hospital	Barkham TMS; Chen YYC; Li C; Lim JX; Maurer-Stroh S; Nagarajan N; Sessions OM; Tang WY; Zair X
EPI_ISL_1678810	Department of Medical Microbiology & Infection prevention, Amsterdam University Medical Centers location AMC	Department of Medical Microbiology & Infection prevention, Amsterdam University Medical Centers location AMC	Fokla Zorgdrager; Janke Schinkel; Marcel Jonges; Matthijs Welkers; Menno de Jong; Robin van Houdt; Sebastien Matamoros; Sjoerd Rebers
EPI_ISL_2925800, EPI_ISL_3673837	Department of Medical Microbiology - section Molde, Molde Hospital	Norwegian Institute of Public Health, Department of Virology	Atiya R Ali; Debech Nadia; Engebretsen Serina Beate; Garcia Llorente Ignacio; Hilde Elshaug; Hilde Vollan; Jon Bråte; Kamilla Heddeland Instefjord; Karoline Bragstad; Kathrine Stene-Johansen; Line Victoria Moen; Marie Paulsen Madsen; Olav Hungnes; Pedersen Benedikte Nevjen; Rasmus Riis Kopperud
EPI_ISL_3162220	Department of Medical Microbiology, Hospital Pengajar Universiti Putra Malaysia	Malaysia Genome Institute	Avisha Richards; Azrin Ahmad; Enizza Kasim; Hui-Yee Chee; Irni Suhayu Sapian; Mohd Anuar Jonet; Mohd Faizal Abu Bakar; Mohd Noor Mat Isa; Muhammad MI; Narcisse Joseph; Nor Azfa Johari; Nor Zahrin Hasran; Nurhezreen Md Iqbal; Shamsidar Sopie; Siti Noraini Othman; Syafinaz Amin-Nordin; Yusuf Muhammad Noor
EPI_ISL_2547429	Department of Medical Virology, School of Medicine Ahvaz Jundishapur University of Medical sciences	Genetics Research Center, University of Social Welfare and Rehabilitation Sciences	Ahmad Tavakoli; Azarakhsh Azaran; Farid yousefi; Hossein Najmabadi.; Kimia Kahrizi; Maryam Beheshtian; Marzieh Mohseni; Mohammad Farahmand; Mohsen Savaie; Seyed Mohammad Jazayeri; Zohreh Fattahi
EPI_ISL_3066150	Department of Microbiology	Department of Microbiology	Paul K.S. Chan; Zigui Chen
EPI_ISL_2833647, EPI_ISL_2833648, EPI_ISL_2833653, EPI_ISL_2833655, EPI_ISL_2833661, EPI_ISL_2833664, EPI_ISL_2833665, EPI_ISL_3505785, EPI_ISL_3505791, EPI_ISL_3505796, EPI_ISL_3505803, EPI_ISL_3505804			
see above	Department of Microbiology, National Institute for Public Health of Kosova	Charité Universitätsmedizin Berlin, Institut für Virologie	Aferdita Hyseni; Barbara Mühlemann; Blendi Jerliu; Christian Drosten; Donjeta Hajdari; Julia Schneider; Jörn Beheim-Schwarzbach; Nazmi Mehmeti; Pranvera Abazi; Talitha Veith; Terry Jones; Victor M Corman; Xhevat Jakupi; Zana Deva
EPI_ISL_1027639, EPI_ISL_1027645	Department of Microbiology, National Institute for Public Health of Kosova	Charité Universitätsmedizin Berlin, Institut für Virologie	Barbara Mühlemann; Christian Drosten; Donjeta Hajdari; Julia Schneider; Jörn Beheim-Schwarzbach; Talitha Veith; Terry Jones; Victor M Corman; Xhevat Jakupi; Zana Deva
EPI_ISL_1034496	Department of Microbiology, The University of Hong Kong	Department of Microbiology, The University of Hong Kong	Kelvin K.W. To; Kwok-Yung Yuen
EPI_ISL_2232960	Department of Microbiology, University Innsbruck	Bergthaler laboratory, CeMM Research Center for Molecular Medicine of the Austrian Academy of Sciences	Andreas Bergthaler; Anna Schedl; Bekir Erguner; Benedikt Agerer; Christoph Bock; Fabian Amman; Jan Laine; Lukas Endler; Maelle Le Moing; Martin Senekowitsch; Michael Schuster; Petr Triska; Thomas Penz
EPI_ISL_463743, EPI_ISL_463746, EPI_ISL_1164628, EPI_ISL_1164640, EPI_ISL_1164679, EPI_ISL_1164746, EPI_ISL_1164749			
see above	Department of Molecular Virology, Cyprus Institute of Neurology and Genetics	Department of Molecular Virology, Cyprus Institute of Neurology and Genetics	Anastasis Oulas; Andreas Hadjisavvas; Christina Christodoulou; Christina Tryfonos; Dana Koptides; Denise Alexandrou; George Krashias; George Spyrou; Jan Richter; Maria Loizidou; Mihalis Panayiotidis; Olga Kalakouta; Pavlos Fanis; Stavros Bashiardes
EPI_ISL_853819	Department of Pathology, Landeskrankenhaus Graz II, Medical University Graz	Bergthaler laboratory, CeMM Research Center for Molecular Medicine of the Austrian Academy of Sciences	Alexander Lercher; Alexandra Popa; Andreas Bergthaler; Anna Schedl; Benedikt Agerer; Christoph Bock; Jakob-Wendelin Genger; Jan Laine; Lukas Endler; Martin Senekowitsch; Michael Schuster; Thomas Penz
EPI_ISL_417177	Department of Pathology, Princess Margaret Hospital	Department of Health Technology and Informatics, Faculty of Health and Social Science, The Hong Kong Polytechnic University	Alan Ka-Lun WU; Alex Yat-Man HO; Barry Kin-Chung WONG; David Ho-Keung SHUM; Eugene Yuk-Keung TSO; Gilman Kit-Hang SIU; Hiu-Yin LAO; Kam-Tong Yip; Kenneth Siu-Sing LEUNG; Kingsley King-Gee TAM; Kit-Man SIN; Kitty Sau-Chun FUNG; Kwok-Cheung LUNG; Lam-Kwong LEE; Man-Chun CHAN; Ming-Pan CHOI; Miranda Chong-Yee YAU; Raymond Wai-To LIU; Sandy Ka-Yee CHAU; Shea Ping YIP; Tak-Lun Que; Timothy Ting-Leung NG; Wai-Shing LEUNG; Wing Cheong YAM; Wing-Kin TO; Yuk-Yung NG
EPI_ISL_2692982	Department of Pathology, School of Medicine, Imam Hospital Complex, Tehran University of Medical Sciences, Tehran, Iran	Genetics Research Center, University of Social Welfare and Rehabilitation Sciences	Ali Jafarpour; Alireza Abdollahi; Azam Ghaziasadi; Azar Hadadi; Hossein Najmabadi.; Khadijeh Jalalvand; Kimia Kahrizi; Marzieh Mohseni; Reza Najafipour; Saber Soltani; Seyed Mohammad Jazayeri; Seyedeh elham Mortazavi; Zohreh Fattahi
EPI_ISL_2687994	Department of Public Health Bucharest	National Institute of Infectious Diseases-Prof. Dr. Matei Bals Molecular Diagnostics Laboratory	Corina Casangiu; Dan Otelea; Leontina Banica; Marius Surleac; Petre Milu; Robert Hohan; Simona Paraschiv
EPI_ISL_3045843	Department of Public Health Buzau	National Institute of Infectious Diseases-Prof. Dr. Matei Bals Molecular Diagnostics Laboratory	Corina Casangiu; Dan Otelea; Leontina Banica; Marius Surleac; Petre Milu; Robert Hohan; Simona Paraschiv

EPI_ISL_3857079	Department of Public Health Constanta	National Institute of Infectious Diseases-Prof. Dr. Matei Bals Molecular Diagnostics Laboratory	Corina Casangiu; Dan Otelea; Leontina Banica; Marius Surleac; Ovidiu Vlaicu; Petre Milu; Robert Hohan; Simona Paraschiv
EPI_ISL_3342031	Department of Public Health Iasi	National Institute of Infectious Diseases-Prof. Dr. Matei Bals Molecular Diagnostics Laboratory	Corina Casangiu; Dan Otelea; Leontina Banica; Marius Surleac; Ovidiu Vlaicu; Petre Milu; Robert Hohan; Simona Paraschiv
EPI_ISL_2324621	Department of Public Health Microbiology Ljubljana, National Laboratory for Health, Environment and Food	Department of Public Health Microbiology Ljubljana, National Laboratory for Health, Environment and Food	José Gonçalves; Katarina Prosenc; Marija Trkov; Martin Bosilj; Metka Paragi; Tom Koritnik; Verica Mioč
EPI_ISL_3386688	Department of Public Health Suceava	National Institute of Infectious Diseases-Prof. Dr. Matei Bals Molecular Diagnostics Laboratory	Corina Casangiu; Dan Otelea; Leontina Banica; Marius Surleac; Ovidiu Vlaicu; Petre Milu; Robert Hohan; Simona Paraschiv
EPI_ISL_576147, EPI_ISL_576148	Department of Respiratory & Other Viral Infections of L.V. Gromashevsky Institute of Epidemiology & Infectious Diseases NAMS of Ukraine	Department of Respiratory & Other Viral Infections of L.V. Gromashevsky Institute of Epidemiology & Infectious Diseases NAMS of Ukraine, JSC "Farmak"	Alla Mironenko; Ihor Kravchuk; Larysa Radchenko; Liudmyla Bolotova; Nataliia Teteriuk
EPI_ISL_450200, EPI_ISL_1385800, EPI_ISL_1711672, EPI_ISL_1732172, EPI_ISL_2757749, EPI_ISL_3827533, EPI_ISL_3827545			
see above	Department of Virology	Department of Virology	Aamer Ikram; Abdul Ahad; Ackermann, N.; Adnan Haider; Ammar Amjad; Antwerpen, M.; Austin Leach; Bengs, K.; Berger, A.; Boehm, S.; Boehmer; Boender; Buchholz, U.; Cai, W.; Corman; D.V.; Dangel, A.; Drosten, C.; Eberle, U.; Fingerle, V.; Grahl, A.; Haas, W.; Hamouda, O.; Hoch, M.; Hoermansdorfer, S.; Ippisch, S.; Joel Montgomery; John Klena; Jones; Katz, K.; Ketan Patel; Konrad, R.; Liebl, B.; M.M.; Marosevic; Massab Umair; Melissa Mobley; Muehlemann, B.; Muhammad Ammar; Muhammad Salman; Muller, N.; Nazish Badar; Poertner, K.; Protzer, U.; Qasim Ali; Reich, A.; Rexroth, U.; Sana Tamim; Schneider, J.; Shannon Whitmer; Sing, A.; Syed Adnan Haider; T.C.; T.S.; Treis, B.; V.M.; Veith, T.; Walter, M.; Wicklein, B.; Woelfel, R.; Woudenberg, T.; Zaira Rehman; Zapf, A.; Zeitlmann, N.; an der Heiden, M.
EPI_ISL_755975, EPI_ISL_756165, EPI_ISL_902877, EPI_ISL_1497156, EPI_ISL_2608827, EPI_ISL_3014554, EPI_ISL_3015014, EPI_ISL_3644543, EPI_ISL_3644734, EPI_ISL_3645103, EPI_ISL_3645153, EPI_ISL_3645226			
see above	Department of Virology and Immunology, University of Helsinki and Helsinki University Hospital, Huslab Finland	Department of Virology, Faculty of Medicine, University of Helsinki, Helsinki, Finland	Essi Korhonen; Hanna Jarva; Hanna Liimatainen; Hannimari Kallio-Kokko; Harri Kangas; Hussein Alburkat; Jenni Virtanen; Maija Lappalainen; Maija Suvanto; Olli Vapalahti; Pekka Ellonen; Phuoc Truong; Ravi Kant; Sari Hannula; Satu Kurkela; Teemu Smura
EPI_ISL_2896259, EPI_ISL_2896261	Department of Virology, Molecular Biology and Genome Research	Department of Virology, Molecular Biology and Genome Research	Kotaria, N.; Kotorashvili, A.; Murtskhvaladze, M.
EPI_ISL_2301241	Department of Virus and Microbiological Special Diagnostics, Statens Serum Institut, Copenhagen, Denmark	Aalborg University	Danish Covid-19 Genome Consortium
EPI_ISL_818831	Department of Virus and Microbiological Special Diagnostics, Statens Serum Institut, Copenhagen, Denmark	Albertsen Lab, Department of Chemistry and Bioscience, Aalborg University, Denmark	Danish Covid-19 Genome Consortium
EPI_ISL_615736, EPI_ISL_615971, EPI_ISL_616111, EPI_ISL_622353	Department of Virus and Microbiological Special Diagnostics, Statens Serum Institut, Denmark	Albertsen lab, Department of Chemistry and Bioscience, Aalborg University, Denmark	Danish Covid-19 Genome Consortia
EPI_ISL_416143	Department of Virus and Microbiological Special diagnostics, Statens Serum Institut, Copenhagen, Denmark.	ViFU	Anders Fomsgaard; Maiken Worsoe Rosenstierne; Morten Rasmussen
EPI_ISL_3394968	Dept. of Laboratory Medicine	Dept. of Laboratory Medicine	Anna Gschaider; Claudia Weber; Fabian Konig; Harald Esterbauer; Oswald Wagner; Petra Jurkowitsch; Robert Strassl; Sabina Plumer
EPI_ISL_3542702	Dept. of Medical Microbiology, Stavanger University Hospital, Helse Stavanger HF	Norwegian Institute of Public Health, Department of Virology	Atiya R Ali; Debech Nadia; Engebretsen Serina Beate; Garcia Llorente Ignacio; Hilde Elshaug; Hilde Vollan; Jon Bråte; Kamilla Heddeland Instefjord; Karoline Bragstad; Kathrine Stene-Johansen; Line Victoria Moen; Marie Paulsen Madsen; Olav Hungnes; Pedersen Benedikte Nevjen; Rasmus Riis Kopperud
EPI_ISL_665259, EPI_ISL_2438754, EPI_ISL_3030519	Dept. of Microbiology and Infection Control, Akershus University Hospital HF	Dept. of Microbiology and Infection Control, Akershus University Hospital HF	Alexander Hesselberg Løvestad; Hege Vangstein Aamot; Nina Handal; Ole Herman Ambur; Silje Bakken Jørgensen
EPI_ISL_2171041, EPI_ISL_3239839	Dhulikhel Hospital, Kathmandu University Hospital	Molecular and Genomics Research Lab, Dhulikhel Hospital, Kathmandu University Hospital	Dipesh Tamrakar; Manu Vanaerschot; Meghnath Dhimal; Navin Adhikari; Nishan Katuwal; Pradip Gyanwali; Rajeev Shrestha; Saroj Bhattarai; Surendra Kumar Madhup
EPI_ISL_3800765	Diagnostyka. Laboratoria Medyczne.	1. ViroGenetics - BSL3 Laboratory of Virology, Małopolska Centre of Biotechnology, Jagiellonian University; 2. Diagtron Laboratoria Łukasz Rąbalski	Gromowski, T.; Kowalski, M.; Labaj; Maciej Kosinski; Mazur-Panasiuk, N.; Natalia Derewonko; P.P.; Pyrc, K.; Rabalski L.; Rogalska-Kupiec M.; Swadzba J.; Sylwia Januszczak; Szulc, P.; Wydmanski, W.
EPI_ISL_2319144	Diagnostyka. Laboratoria Medyczne.	1. ViroGenetics - BSL3 Laboratory of Virology, Małopolska Centre of Biotechnology, Jagiellonian University; 2. genXone SA, Research & Development Laboratory	Brylak A; Drweska-Matelska N; Gidlewicz A; Grabowski J; Gromowski, T.; Januszczak S; Kaszuba M; Kowalski, M.; Krych L; Labaj; Mazur-Panasiuk, N.; Nowicki G; P.P.; Pyrc, K.; Sykulski M; Szeszko K; Szulc, P.; Wydmanski W.
EPI_ISL_3050665, EPI_ISL_3372592, EPI_ISL_3372593, EPI_ISL_3372595, EPI_ISL_3372604	Dirección regional de salud del Callao (DIRESA-CALLAO)	Centro de Investigaciones Tecnológicas, Biomédicas y Medioambientales (CITBM)	B; Huaman; J. Alarcon; M. Cuellar; M. Ramirez; M. Sovero
EPI_ISL_747356, EPI_ISL_760242, EPI_ISL_850403, EPI_ISL_1063648, EPI_ISL_1587549, EPI_ISL_2284658, EPI_ISL_2332354, EPI_ISL_2332355, EPI_ISL_2332464, EPI_ISL_2332520, EPI_ISL_2754661, EPI_ISL_2967328, EPI_ISL_3026048, EPI_ISL_3026566, EPI_ISL_3369227, EPI_ISL_3545218, EPI_ISL_3772919			
see above	Division of Emerging Infectious Diseases, Bureau of Infectious Diseases Diagnosis Control, Korea Disease Control and Prevention Agency	Division of Emerging Infectious Diseases, Bureau of Infectious Diseases Diagnosis Control, Korea Disease Control and Prevention Agency	Ae Kyung Park; Chae Young Lee; Chaeyoung Lee; Eun-Jin Kim; Heui Man Kim; Il-Hwan Kim; Jeong-Ah Kim; Jeong-Min Kim; Jin Sun No; Namjoo Lee; Sang Hee Woo
EPI_ISL_2348803	Division of Epidemiology	COVID-19 Network Investigations (CONI) Alliance	Angkana Huang; Anthony R. Jones; Arporn Wangwiwatsin; Arthicha Wongkumma; Bhakbhoom Panthan; Chonticha Klungtong; Duangkamon Loesbanluechai; Ekawat Pasomsub; Elizabeth Batty; Insee Sensorn; Janjira Thaipadungpanit; Kawinna Kerdsalung; Khajohn Joonlasak; Kingkan Rakmanee; Krittikorn Kumpornsin; Namfon Kotanan; Pantila Taweewigyakarn; Prangsiri Nalaem; Stefan Fernandez; Thanat Chookajorn; Theerarat Kochakarn; Treewat Watthanachockchai; Wasun Chantratita; Wudtichai Manasatienkij
EPI_ISL_3827662, EPI_ISL_3827702	Division of Medical Virology, National Health Laboratory Service (NHLS), Tygerberg Hospital / Stellenbosch University	CERI, Centre for Epidemic Response and Innovation, Stellenbosch University and CERI-KRISP, KZN Research Innovation and Sequencing Platform	Alvera Vorster; Bronwyn Kleinhans; Carel J van Heerden; Gert van Zyl; Giandhari Jennifer; Kamela Mahlakwane; Karabo Phadu; Mathilda Claassen; Naidoo Yeshnee; Pillay Sureshnee; Ren Veikondis; San James; Shannon Wilson; Susan Engelbrecht; Tania Stander; Tegally Houriiyah; Tongai Maponga; Tshiabuila Derek; Wilkinson Eduan; Wolfgang Preiser; Yajna Ramphal; de Oliveira Tulio
EPI_ISL_3666165	Division of Medical Virology, National Health Laboratory Service (NHLS), Tygerberg Hospital / Stellenbosch University	Division of Medical Virology, National Health Laboratory Service (NHLS), Tygerberg Hospital / Stellenbosch University	Bronwyn Kleinhans; Gert van Zyl; Kamela Mahlakwane; Shannon Wilson; Susan Engelbrecht; Tongai Maponga; Wolfgang Preiser
EPI_ISL_3068079, EPI_ISL_3068086	Division of Medical Virology, National Health Laboratory Service (NHLS), Tygerberg Hospital / Stellenbosch University	Division of Medical Virology, Stellenbosch University and NHLS Tygerberg Hospital	Bronwyn Kleinhans; Gert van Zyl; Kayla Delaney; Susan Engelbrecht; Wolfgang Preiser
EPI_ISL_412871, EPI_ISL_498004, EPI_ISL_498013, EPI_ISL_510580, EPI_ISL_514925	Division of Viral Diseases, Center for Laboratory Control of Infectious Diseases, Korea Centers for Diseases Control and Prevention	Division of Viral Diseases, Center for Laboratory Control of Infectious Diseases, Korea Centers for Diseases Control and Prevention	Daesang Lee; Dong Hyun Song; Heui Man Kim; Hye-Jun Jo; Jeong-Min Kim; Jun-Sub Kim; Mi-Seon Kim; Myung Guk Han; Namjoo Lee; Sang Hee Woo; Sehee Park; Seong Tae Jeong; Yoon-Seok Chung
EPI_ISL_2724045	Dr S raju, Director of public heath and preventive medicine	inStem NCBS – INSACOG	Uma Ramakrishnan Dasaradhi Palakodeti Aswin SaiNarain
EPI_ISL_3388900, EPI_ISL_3570031, EPI_ISL_3570032, EPI_ISL_3570034, EPI_ISL_3570055, EPI_ISL_3578112, EPI_ISL_3578139, EPI_ISL_3578161, EPI_ISL_3578188, EPI_ISL_3635701, EPI_ISL_3798358			
see above	Dr. Risch Ostschweiz AG	Microbiology, Dr. Risch	Dominique Fabien Hilti; Faina Wehrli; Lorenz Risch; Martin Risch; Nadia Wohlwend; Sinem Kas; Thomas Bodmer
EPI_ISL_417212	Dunedin Hospital	University of Otago	B. Lawley; J. Grant; J. Ussher; M.E. Quiñones-Mateu; R. Harfoot
EPI_ISL_460805, EPI_ISL_523164, EPI_ISL_2145740	Dutch COVID-19 response team	Erasmus Medical Center	Anne van der Linden; Annemiek van der Eijk; Aura Timen; Bas Oude Munnink; Claudia Schapendonk; Corien Swaan; Corine GeurtsvanKessel; David Nieuwenhuijse; Emmanuelle Munger; Irina Chestakova; Jeroen van Kampen; Jolanda Voermans; Madelief Mollers; Manon Haverkate; Marion Koopmans; Marjan Boter; Mark Pronk; Mart Stein; Pascal Lexmond; Reina Sikkema; Richard Molenkamp; Sandra Kengne Kamga Mobou; Stefan van Nieuwkoop; Theo Bestebroer; on behalf of the Dutch national COVID-19 respo; on behalf of the Dutch national COVID-19 response team.
EPI_ISL_547446, EPI_ISL_547451, EPI_ISL_636515, EPI_ISL_636519, EPI_ISL_723233, EPI_ISL_728677, EPI_ISL_1014561, EPI_ISL_1014567, EPI_ISL_1014576, EPI_ISL_1014585, EPI_ISL_1014666, EPI_ISL_1089780, EPI_ISL_1165619, EPI_ISL_1233007, EPI_ISL_1288132, EPI_ISL_1288133, EPI_ISL_1370872, EPI_ISL_1371268, EPI_ISL_1457573, EPI_ISL_1522182, EPI_ISL_1522202, EPI_ISL_1705301, EPI_ISL_1961912, EPI_ISL_2093403, EPI_ISL_2093435, EPI_ISL_2093442, EPI_ISL_2093657, EPI_ISL_2093754, EPI_ISL_2093761, EPI_ISL_2093767, EPI_ISL_2093769, EPI_ISL_2094339, EPI_ISL_2094342, EPI_ISL_2094345, EPI_ISL_2094347, EPI_ISL_2217866, EPI_ISL_2218097, EPI_ISL_2218273, EPI_ISL_2220430, EPI_ISL_2302857, EPI_ISL_2303447, EPI_ISL_2303450, EPI_ISL_2303978, EPI_ISL_2405481, EPI_ISL_2405550, EPI_ISL_2405561, EPI_ISL_2405573, EPI_ISL_2405584, EPI_ISL_2405608, EPI_ISL_2405609, EPI_ISL_2405889, EPI_ISL_2406317, EPI_ISL_2474832, EPI_ISL_2475181, EPI_ISL_2475386, EPI_ISL_2475681, EPI_ISL_2476276, EPI_ISL_2476284, EPI_ISL_2610471, EPI_ISL_2610646, EPI_ISL_2610658, EPI_ISL_2672546, EPI_ISL_2673213, EPI_ISL_2673225, EPI_ISL_2673252, EPI_ISL_2673270, EPI_ISL_2787142, EPI_ISL_2787322, EPI_ISL_2787813, EPI_ISL_2787946, EPI_ISL_2787992, EPI_ISL_2788182, EPI_ISL_2788186, EPI_ISL_2788368, EPI_ISL_2862718, EPI_ISL_2862790, EPI_ISL_2862792, EPI_ISL_2862796, EPI_ISL_2981892, EPI_ISL_2981897, EPI_ISL_2981900, EPI_ISL_2981901, EPI_ISL_3056793, EPI_ISL_3057212, EPI_ISL_3058076, EPI_ISL_3136562, EPI_ISL_3136572, EPI_ISL_3136586, EPI_ISL_3136605, EPI_ISL_3136624, EPI_ISL_3137106, EPI_ISL_3138367, EPI_ISL_3138394, EPI_ISL_3138405, EPI_ISL_3253259, EPI_ISL_3257896, EPI_ISL_3257913, EPI_ISL_3257938, EPI_ISL_3257951, EPI_ISL_3257963, EPI_ISL_3257978, EPI_ISL_3259189, EPI_ISL_3389278, EPI_ISL_3389741, EPI_ISL_3389772, EPI_ISL_3389776, EPI_ISL_3389781, EPI_ISL_3390570, EPI_ISL_3390704, EPI_ISL_3390705, EPI_ISL_3731397, EPI_ISL_3731651, EPI_ISL_3731770, EPI_ISL_3731802, EPI_ISL_3732335, EPI_ISL_3732369, EPI_ISL_3732378, EPI_ISL_3732380, EPI_ISL_3732390, EPI_ISL_3732391, EPI_ISL_3732432, EPI_ISL_3732848, EPI_ISL_3732904, EPI_ISL_3732925, EPI_ISL_3732926, EPI_ISL_3732934, EPI_ISL_3732935, EPI_ISL_3732942, EPI_ISL_3732962, EPI_ISL_3732965, EPI_ISL_3732966, EPI_ISL_3733698, EPI_ISL_3733731, EPI_ISL_3733853, EPI_ISL_3733872, EPI_ISL_3737183, EPI_ISL_3771759			
see above	Dutch COVID-19 response team	National Institute for Public Health and the Environment (RIVM)	Adam Meijer; AnneMarie van den Brandt; Annelies Kroneman; Bas van der Veer; Chantal Reusken; Dennis Schmitz; Dirk Eggink; Euníce Then; Florian Zwagemaker; Harry Vennema; James Groot; Jeroen Cremer; Jolienke Hardeman; Karim Hajji; Kim Freriks; Linda van de Nes; Lisa Wijsman; Lynn Aarts; Melissa van Tuil; Robert Kohl; Ryanne Jaarsma; Sanne Bos; Sharon van den Brink; Sjoerd Kuiling; Stijn van Rossum; on behalf of the national COVID-19 response team

EPI_ISL_1137609	Département de Maladies Infectieuses, CHU Farhat Hached Sousse, Tunisie	Laboratoire des Procédés de Criblage Moléculaire et Cellulaire-Centre de Biotechnologie de Sfax	A. and Masmoudi, S.; Abdelmoulah, F.; Abid, M.; Abid, N.; Ajili, F.; Aouni, M.; Ben Ayed, I.; Bensaid, M.; Chawech, H.; Chtourou, A.; Elargoubi, A.; Fki-berrajah, L.; Gaaloul, I.; Gargouri, S.; Hammami, A.; Kamoun, S.; Karray Hakim, H.; Kharat, N.; Letaief, A.; Mastouri, M.; Mhalla, S.; Nabli, A.; Rebai; Smeti, I.; Souissi, A.; Stambouli, N.; Turki, M.
EPI_ISL_450518, EPI_ISL_501275, EPI_ISL_501829	E. Gulbja Laboratorija	Latvian Biomedical Research and Study Centre	Dmitrijs Perminovs; Ivars Silamiķelis; Jānis Kloviņš; Kaspars Megnis; Mikus Gavars; Monta Ustinova; Uga Dumpis; Vita Rovīte; Ņikita Zrelovs
EPI_ISL_1312490	E. Gulbja laboratorija	Latvian Biomedical Research and Study Centre	Davids Fridmanis; Dmitrijs Perminovs; Guntars Zarins; Ivars Silamikelis; Janis Klovins; Janis Pjalkovskis; Jurijs Perevoscikovs; Kaspars Megnis; Laila Silamikele; Lauma Freimane; Laura Ansone; Liga Birzniece; Mikus Gavars; Monta Ustinova; Nikita Zrelovs; Uga Dumpis; Una Krumina; Vita Rovite
EPI_ISL_3838369	EAST LONDON LABORATORY	National Institute for Communicable Diseases of the National Health Laboratory Service	Amoako DG; Bhiman JN; Everatt J; Ismail A; Mahlangu B; Mnguni A; Mohale T; Ntuli N; Scheepers C
EPI_ISL_3259913	EHNV	Laboratory of genomics and metagenomics	Claire Bertelli; Damien Jacot; Gilbert Greub; Sébastien Aeby; Trestan Pillonel
EPI_ISL_2370352, EPI_ISL_2370859	ETHNIKO KENTRO AIMODOSIAS E.KE.A.	Greek Genome Center, Biomedical Research Foundation of the Academy of Athens (BRFAA)	Dimitrios Thanos; Efthimia Petinaki; Emmanouil Athanasiadis; Giannis Vatsellas; Katerina Zoi; Kostas Stamoulis; Theodoros Loupis
EPI_ISL_593531	Eastern Ontario Regional Laboratory Association	McMaster University	Ahmed Draia; Andrew G. McArthur; Emily Panousis; Hooman Derakhshani; Jalees Nasir; Leanne Mortimer; Robert Slinger
EPI_ISL_2478881, EPI_ISL_2479300, EPI_ISL_3116423, EPI_ISL_3524202, EPI_ISL_3525416	Edmonton Provincial Lab	Public Health Agency of Canada (PHAC) National Microbiology Laboratory	Buss; Croxen M; Deo A; Dieu P; E; Ferrato C; Gill K; Khan F; Koleva P; Li V; Lloyd C; Lynch T; Ma R; Murphy S; Pabbaraju K; Shokoples S; Thayer J; Tipples G; Whitehouse M; Wong A; Yu C; Zelyas N
EPI_ISL_2029260	Ekstralab Tuzla	Alea Genetic Centre	Adis Kandic; Dino Pecar; Enis Kandic; Lana Salihefendic; Nusret Butkovic; Rijad Konjhodzic
EPI_ISL_3655288	Elisabeth Pharmacon	University Hospital Brno, CMBG	Jan Svaton; Kristyna Dufkova; Martina Lengerova; Matej Bezdicek; Pavlina Volfova
EPI_ISL_2932514	Emergency County Hospital Targu Mures	National Institute of Infectious Diseases-Prof. Dr. Matei Bals Molecular Diagnostics Laboratory	Corina Casangiu; Dan Otelea; Leontina Banica; Marius Surleac; Petre Milu; Robert Hohan; Simona Paraschiv
EPI_ISL_1122017	Emergency department of COVID-19, Beijing Ditan Hospital, Capital Medical University, Beijing, China.	National Institute for Viral Disease Control and Prevention	3*; Di Tian1; Gang Liu1; Gang Wan1; Jianbo Tan1; Long Liu1; Pan Xiang1; Shuangli Zhu2; Shuping Cui1; Tingyu Zhang1; Wei Zhang1#; Wenbo Xu2# and Zhihai Chen1#; Xianbo Wang1; Xiang Zhao2; Yang Song2*; Yanhai Wang2; Yanli Xu1; Yu Wang1; Ziruo Ge1*
EPI_ISL_1897884, EPI_ISL_1898554, EPI_ISL_2241623	Ethiopian Biotechnology Institute (EBTI)	International Centre for Genetic Engineering and Biotechnology (ICGEB) and ARGO Open Lab for Genome Sequencing	Alessandro Marcello; Danilo Licastro; Emanuele Orsini; Getnet Hailu; Hailu Dadi; Kassahun Tesfaye; Keyru Tuki; Kominist Asmamaw; Molalegne Bitew; Simeone Dal Monego; Yakob Gebregziabher Tsegay
EPI_ISL_2844313	Europe/Sweden/Vastragotaland/Unilabs	Unilabs/Eskilstuna/Sweden	Emma Arvidsson
EPI_ISL_1712253	FSBI «NATIONAL MEDICAL RESEARCH CENTER FOR OBSTETRICS, GYNECOLOGY AND PERINATOLOGY NAMED AFTER ACADEMICIAN V.I.KULAKOV» MINISTRY OF HEALTHCARE OF THE RUSSIAN FEDERATION	Center for Precision Genome Editing and Genetic Technologies for Biomedicine, Pirogov Medical University, Moscow, Russian Federation	Anastasia Shut; Andrey Krivoy; Dmitriy Korostin; Margarita Korzhanova; Vera Belova; Yegor Botsmanov
EPI_ISL_3398808	FUNDACION CARDIOVASCULAR DE COLOMBIA	Instituto Nacional de Salud	Carlos Franco-Muñoz; Carmen Osorio; Diana Malo; Diego A. Álvarez-Díaz; Diego Andrés Prada; Gerardo Santamaría; Hector Alejandro Ruiz-Moreno; Jhonnatan Reales-González; Jorge Rivera; Juan Camilo Martinez; Julian Naizaque; Katherine Laiton-Donato; Lisseth Pardo; Magdalena Wiesner; Marcela Mercado-Reyes; Maria T.Herrera-Sepúlveda; Marta Lopez Blanco; Martha Lucia Ospina Martinez; Paola Rojas; Sergio Gomez; Sheryll Corchuelo; Ángela Alarcon Cruz
EPI_ISL_3066769, EPI_ISL_3459379	FUNDACION VALLE DE LILI	Instituto Nacional de Salud	Carlos Franco-Muñoz; Carmen Osorio; Diana Malo; Diego A. Álvarez-Díaz; Diego Andrés Prada; Gerardo Santamaría; Hector Alejandro Ruiz-Moreno; Jhonnatan Reales-González; Jorge Rivera; Juan Camilo Martinez; Julian Naizaque; Katherine Laiton-Donato; Lisseth Pardo; Magdalena Wiesner; Marcela Mercado-Reyes; Maria T. Herrera-Sepúlveda; Maria T.Herrera-Sepúlveda; Marta Lopez Blanco; Martha Lucia Ospina Martinez; Paola Rojas; Sergio Gomez; Sheryll Corchuelo; Ángela Alarcon Cruz
EPI_ISL_804016	Facultad de Medicina, Universidad de Atacama	Facultad de Ciencias de la Vida, UNAB	Claudio Meneses; César Echeverría; Dayán Sanhueza; Eduardo Castro; Jorge Olivares; Macarena Bastías; Sebastián Wolter; Waldo Díaz
EPI_ISL_1273101	Faculty of Medicine, Al-Quds University	Faculty of Medicine, Al-Quds University	Al-Jawabreh, A.; Dumaidi, K.; Ereqat, S.; Nasereddin, A.
EPI_ISL_3020619, EPI_ISL_3020842, EPI_ISL_3020943	Fimlab Laboratoriot Oy Tampere	Expert Microbiology, National Institute for Health and Welfare	Carita Savolainen-Kopra; Erika Lindh; Haider al-Hello; Jani Halkilahti; Kirsi Liitsola; Niina Ikonen; Olli Vapalahti; Pekka Ellonen; Phuoc Truong; Päivi Laurila; Ravi Kant; Sari Hannula; Soile Blomqvist; Teemu Smura
EPI_ISL_594319	Florida Bureau of Public Health Laboratories	Florida Bureau of Public Health Laboratories	Jason Blanton; Sarah Schmedes
EPI_ISL_2889836, EPI_ISL_2889841, EPI_ISL_2889844	Fondation Congolaise pour la recherche medicale (FCRM)	Fondation Congolaise pour la Recherche Médicale	Abel Lissom; Batchi-Bouyou Armel Landry; Francine Ntoumi; Jean Claude Djontu; Mfoutou Mapanguy Claujens Chastel; Thirumalaisamy P. Velavan
EPI_ISL_3040125, EPI_ISL_3040126, EPI_ISL_3040131, EPI_ISL_3040134, EPI_ISL_3040136	Fondation Congolaise pour la recherche medicale (FCRM), Francine Ntoumi	Fondation Congolaise pour la Recherche Médicale	Batchi-Bouyou Armel Landry; Dr. Abel Lissom; Dr. Jean Claude Djontu; Mfoutou Mapanguy Claujens Chastel; Prof. Dr. Thirumalaisamy P. Velavan; Prof. Francine Ntoumi
EPI_ISL_1671929, EPI_ISL_1671930	Fondation Congolaise pour la recherche medicale (FCRM), Francine Ntoumi	Institute of Tropical Medicine	Prof. Dr. Thirumalaisamy P. Velavan and Prof. Francine Ntoumi
EPI_ISL_912387	Fondation Congolaise pour la recherche medicale (FCRM), Francine Ntoumi	NGS Competence Center Tuebingen, Institut für Medizinische Mikrobiologie und Hygiene, Universitaetsklinikum Tübingen	Angel Angelov
EPI_ISL_3006797	Fujian Center for Disease Control and Prevention	Fujian Center for Disease Control and Prevention	Huang Zhimiao; Lin Qi; Weng Yuwei
EPI_ISL_1555799, EPI_ISL_1839632, EPI_ISL_1993055, EPI_ISL_2179869, EPI_ISL_3208946, EPI_ISL_3350475, EPI_ISL_3352391, EPI_ISL_3523878, EPI_ISL_3661476			
see above	Fulgent Genetics	Centers for Disease Control and Prevention Division of Viral Diseases, Pathogen Discovery	Adrian Paskey; Becky Tsai; Benafsh Sapra; Benjamin Rambo-Martin; Christopher Gulvick; Clinton Paden; Clinton R. Paden; Dakota Howard; Darlene Wagner; Dhwani Batra; Doreen Ng; Duncan MacCannell; Harry Gao; James Xie; Jason Caravas; John Gao; Joseph Fierro; Kara Moser; Matthew Schmerer; Mickey Li; Peter Cook; Peter W. Cook; Scott Sammons; Shatavia Morrison; Yan Meng; Yvette Unoarumhi
EPI_ISL_812176	GA Department of Public Health Laboratory	Pathogen Discovery, Respiratory Viruses Branch, Division of Viral Diseases, Centers for Disease Control and Prevention	Anna Montmayeur; Anna Uehara; Brian Lynch; Clinton R. Paden; Haibin Wang; Jing Zhang; Krista Queen; Peter Cook; Rachel Marine; Suxiang Tong; Yan Li; Ying Tao
EPI_ISL_730577	Gazi University Faculty of Medicine, Medical Virology Laboratory	Gazi University Faculty of Medicine, Medical Virology Laboratory	Erdem Şahin; Gülendam Bozdayı; Hager Muftah; Işıl Fidan; Kayhan Çağlar; Murat Dizbay; Selin Yiğit; Shaknoza Sarzhanova; Özlem Güzel Tunçcan
EPI_ISL_3026020	Geelong Centre for Emerging Infectious Diseases, Deakin University	Geelong Centre for Emerging Infectious Diseases, Deakin University	Alexandersen, S.; Bhatta; Chamings, A.; T.R.
EPI_ISL_3491807	Gencore - Universidad de los Andes	Gencore - Universidad de los Andes	Catherine Jaller; Cristian Barrera; David González; Luisa Sacristan; Marcela Guevara; Silvia Restrepo
EPI_ISL_467300	General Hospital "Abdulah Nakas"	Alea Genetic Center	Damir Marjanovic; Dino Pecar; Enis Kandic; Lana Salihefendic; Nihad Fejzic; Rijad Konjhodzic; Sead Jazic; Teufik Goletic
EPI_ISL_2029122	General Hospital "Abdulah Nakas"	Alea Genetic Centre	Adis Kandix; Dino Pecar; Enis Kandic; Lana Salihefendic; Rijad Konjhodzic
EPI_ISL_677725, EPI_ISL_954746	General Hospital - Ohrid	Research Center for Genetic Engineering and Biotechnology "Georgi D. Efremov" , Macedonian Academy of Sciences and Arts	Aleksandar J. Dimovski; Dijana Plasheska-Karanfilska; Gjorgji Bozinovski; Milena Jakimovska; Predrag Noveski; RCGEB - MASA
EPI_ISL_1972223	General Hospital - Prilep	Research Center for Genetic Engineering and Biotechnology "Georgi D. Efremov" , Macedonian Academy of Sciences and Arts	Aleksandar J. Dimovski; Dijana Plasheska-Karanfilska; Gjorgji Bozinovski; Milena Jakimovska; Predrag Noveski
EPI_ISL_2839548	General Hospital - Veles	Research Center for Genetic Engineering and Biotechnology "Georgi D. Efremov" , Macedonian Academy of Sciences and Arts	Aleksandar J. Dimovski; Dijana Plasheska-Karanfilska; Gjorgji Bozinovski; Milena Jakimovska; Predrag Noveski
EPI_ISL_406798	General Hospital of Central Theater Command of People's Liberation Army of China	BGI & Institute of Microbiology, Chinese Academy of Sciences & Shandong First Medical University & Shandong Academy of Medical Sciences & General Hospital of Central Theater Command of People's Liberation Army of China	Weifeng Shi and Zhenhong Hu; Weijun Chen; Yuhai Bi
EPI_ISL_746504, EPI_ISL_746585, EPI_ISL_746650, EPI_ISL_1167708, EPI_ISL_1167729, EPI_ISL_1300455, EPI_ISL_1321509, EPI_ISL_1321544, EPI_ISL_1470544, EPI_ISL_1712375, EPI_ISL_2391112, EPI_ISL_2391123, EPI_ISL_2391231, EPI_ISL_2391354, EPI_ISL_2391451, EPI_ISL_2391462, EPI_ISL_2508616, EPI_ISL_2509182, EPI_ISL_2659133, EPI_ISL_2756072, EPI_ISL_2756105, EPI_ISL_2756151, EPI_ISL_2756237, EPI_ISL_2894930, EPI_ISL_3132695, EPI_ISL_3149084, EPI_ISL_3185690, EPI_ISL_3364940, EPI_ISL_3368432, EPI_ISL_3369293, EPI_ISL_3369363, EPI_ISL_3536108, EPI_ISL_3537145, EPI_ISL_3537336, EPI_ISL_3537360			
see above	Genetica Molecular and Subdepartamento de Virologia ISP Chile	Instituto de Salud Publica de Chile	Andres Castillo; Barbara Parra; Constanza Campano; Gisselle Barra; Jaime Lagos; Javier Tognarelli; Jorge Fernandez; Karen Orostica; Loredana Arata; Patricia Bustos; Rodrigo Fasce; Soledad Ulloa
EPI_ISL_3857568	Genome Analysis Center, Yamanashi Central	Genome Analysis Center, Yamanashi Central Hospital	Yosuke Hirotsu

	Hospital		
EPI_ISL_735337	Genomic Laboratory (GLAB) (Conjoint lab of Health Directorate of Istanbul and Istanbul Technical University)	Genomic Laboratory (GLAB), Istanbul Technical University	Arzu Irvem; Ayse Serra Ozel; Betsi Kose; Gizem Alkurt; Gizem Dinler Doganay; Ilker Karacan; Jale Yildiz; Levent Doganay; Mehtap Aydin; Nihat Bugra Agaoglu; Nilsun Altunal; Nisan Denizce Can; Ozlem Akgun Dogan; Payam Zolfagharian; Tugba Kizilboga Akgun; Yasemin Kendir Demirkol
EPI_ISL_632908	Genomic Sciences, Rehman Medical Institute	Genomic Sciences, Rehman Medical Institute	Afridi; Ali, J.; H. and Jehanzeb, V.; Haider; Jan; S.A.; Sabiha, B.; U.K.
EPI_ISL_3215994	Genomica Lab Molecular, Mexico	Andersen lab at Scripps Research	Jose Horacio Reyna Verdugo; Jose Roman Chavez Mendez; Luis Alberto Rangel Gonzalez; Martin Gonzalez Ibarra; SEARCH Alliance San Diego with Jonathan Gonzalez Garcia
EPI_ISL_812791, EPI_ISL_812834, EPI_ISL_2566467, EPI_ISL_2566470	Genomics Program, Children Cancer Hospital	Genomics Program, Children Cancer Hospital	Abdelaziz, H.; Abdo, I.; Abouelnaga, S.; Almeldin, A.; Amer, K.; Bakry, U.; Diab, A.; El-Shaqnqery, H.; El-Zayat, M.; ElHaddad, A.; ElHalafawy, A.; Elnaqeeb, M.; Farawela, H.; Farawyla, H.; Gomaa, C.; Hadad, A.; Halafawy, A.; Hammad, M.; Hassan, R.; Hassan, W.; Hatem, A.; Hossam, M.; Hussein, S.; Ismail, J.; Jalal, D.; Magdeldin, S.; Mansour, T.; Monuir, G.; Saaid, M.; Said, D.; Salah, H.; Samir, O.; Sayed, A.; Shalaby, L.; Soliman, M.; Soliman, S.; Yahia, A.; shalaby, L.
EPI_ISL_3230192, EPI_ISL_3385483	Genomik Solidaritas Indonesia Laboratorium	Genomik Solidaritas Indonesia Laboratorium	Anna Christina Brazia; Annisa Muthiah Sukirman; Anuraj Shankar; Ariel Pradipta; Carissa Sintca Wijaya; Dhahlia Agustina Cahyono; Gracia Felias Enos Korompis; Himawan Masyhuri; Louisa Markus; Meutia Ayuputeri Kumaheri; Vania Gavrila Wikasa
EPI_ISL_539557	Gerencia del área de salud de Badajoz, Llerena y Zafra	Instituto de Salud Carlos III	A. Monzón; C. Pazos; F. Casas; I; I. Jiménez; Iglesias-Caballero; M. Camarero; M. Cuesta; M. González-Esguevillas; M. Molinero Calamita; M. Zaballos; P. Jiménez; S. Juliá; S. Pozo; S. Varona
EPI_ISL_745664	Ginkgo Bioworks Clinical Laboratory	Utah Public Health Laboratory	Alex Plocik; Becky Schilling; Birgitte Simen; David R. Hillyard; E. Susan Slechta; Erin L. Young; James McGann; Jeffrey B. Stevenson; Jim Griffin; Keith Robison; Kelly Oakeson; Malaika McKenzie-Bennett; Martha Pierson; Melanie A. Mallory; Michael T. Pyne; Michelle Spencer; Rebecca Littlefield; Salika M. Shakir; Tara Gallagher
EPI_ISL_3866891, EPI_ISL_3866893	Global Medical Center	Virology Lab, Jaber Al Ahmad Hospital	Dr. Ebaa Al-Awadhi; Dr. Zahrah Buhamad; Estabraq Kathim; Haroon Masih; Khubaid-ur-Rehman
EPI_ISL_775020	Gonoshasthya-RNA Molecular Research Center	Gonoshasthya-RNA Molecular Research Center	Firoz Ahmed; Maha Jamiruddin; Mahfuza Marzan; Md. Ahsanul Haq; Mohd. Raeed Jamiruddin; Mohib Ullah Khondoker; Mousumi Chaity; Mumtarin Jannat Oishee; Nafisa Azmuda; Nihad Adnan; Nowshin Jahan; Salma Akter; Sayeda Moriam Liza; Shahad Saif Khandker; Shahana Sharmin; Tamanna Ali; Taslin Jahan Mou
EPI_ISL_496643, EPI_ISL_496914, EPI_ISL_1225441, EPI_ISL_1225458, EPI_ISL_1225481, EPI_ISL_1225505, EPI_ISL_1225511, EPI_ISL_1225513, EPI_ISL_1225547, EPI_ISL_1225556, EPI_ISL_1225558, EPI_ISL_1225577, EPI_ISL_1502855, EPI_ISL_1502919, EPI_ISL_1503107			
see above	Gorgas Memorial Laboratory of Health Studies	Gorgas Memorial Laboratory of Health Studies	Adriana Weeden; Alejandra Valoy; Alexander A Martinez; Alexander Martinez; Ambar Moreno; Anyuri Ortiz; Brechla Moreno; Castillo Jorge; Claudia Gonzalez; Claudia Gonzalez Sandra Lopez-Verges; Daniel Castillo; Danilo Franco; Davis Beltran; Dimelza Arauz; Elimelec Valdespino; Franco Danilo; Gonzalez Claudia; Gretel Vasquez; Ilka Guerra; Isela Guerrero; Jessica Gondola; Jim Chang; Juan Miguel Pascale; Layda Abrego; Leyda Abrego; Lisseth Saenz; Lopez-Verges Sandra; Mabel Martinez-Montero; Maria Chen-German; Marlene Castillo; Marlenne Castillo; Martinez Alexander; Melissa Gaitan; Moreno Ambar; Moreno Brechla; Oris Chavarria; Ortiz Alma; Rita Corrales; Rita Rodriguez; Sandra Lopez-Verges; Yamilka Diaz; Yaneth Pitti; Zumara Chaverra
EPI_ISL_3507501	Government General Hospital	Centre for DNA Fingerprinting and Diagnostics	Ashwin Dalal; Asmita Gupta; Divya Vashisht; Murali Bashyam; Nagamani Kammili; Reelina Basu; Vinay Donipadi
EPI_ISL_3507520	Government Maternity Hospital	Centre for DNA Fingerprinting and Diagnostics	Ashwin Dalal; Asmita Gupta; Divya Vashisht; Murali Bashyam; Nagamani Kammili; Reelina Basu; Vinay Donipadi
EPI_ISL_2105484	Governor Celestino Gallares Memorial Hospital	Philippine Genome Center	Alethea R. de Guzman; Anna Ong-Lim; Arianne A. Zamora; Asia Louisa U. Chong; Benedict A. Maralit; Candice Francheska B. Tambaoan; Carlo M. Lapid; Celia Carlos; Devon Ray Pacial; Edsel Maurice Salvaña; El King D. Morado; Eva Maria Cutiongco-de la Paz; Francis A. Tablizo; Irish Coleen A. Asin; Jaime C. Montoya; Jan Michael C. Yap; Jo-Hannah S. Llames; John Q. Wong; Joshua Gregor A. Dizon; Juan Antonio R. Magalang; Karol Sophia Agape R. Padilla; Kenneth M. Kim; Kris P. Punayan; Marc Edsel C. Ayes; Marc Jerrone R. Castro; Maria Rosario Singh-Vergeire and Cynthia P. Saloma; Maria Sofia L. Yangzon; Marissa Alejandria; Razel Nikka M. Hao; Rianna Patricia S. Cruz; Sheila Mae M. Araiza
EPI_ISL_2602199	Gravity Diagnostics, LLC	Gravity Diagnostics, LLC	Gravity Diagnostics
EPI_ISL_2364885, EPI_ISL_2371359	Greek Genome Center, Biomedical Research Foundation of the Academy of Athens (BRFAA)	Greek Genome Center, Biomedical Research Foundation of the Academy of Athens (BRFAA)	Dimitrios Thanos; Emmanouil Athanasiadis; Giannis Vatsellas; Katerina Zoi; Theodoros Loupis
EPI_ISL_698265	Group 42 (G42) Healthcare, Abu Dhabi, United Arab Emirates; Department of Health, The United Arab Emirates	G42 Healthcare	Ashish Koshy; Budoor Alqarni; Denghui Liu; Fang Chen; Hanif Khalak; Huanming Yang; Javier Quilez; Jian Wang; Junhua Li; Ke Liang; Long Lin; Mohammed Saifuddin Fasihuddin; Nan Qiao; Nawal Ahmed Mohamed Al Kaabi; Pauline Ogrodzki; Pei Wu; Peng Xiao; Pengjuan Liu; Rong Liu; Sally Mahmoud; Siyang Liu; Stephen S. Francis; Tao Ma; Vinay Kusuma; Walid Abbas Zaher; Weibin Liu; Wenjun He; Xavier Anton; Xin Jin; Xin Meng; Xinyu Huang; Xun Xu; Zhaorong Yuan
EPI_ISL_2339779	Grupo de Investigación en Enfermedades Tropicales del Ejército (GINETEJ), Laboratorio de Referencia e Investigación, Dirección de Sanidad Ejército, Bogotá, Colombia	Centro de Investigaciones en Microbiología y Biotecnología-UR (CIMBIUR), Facultad de Ciencias Naturales, Universidad del Rosario, Bogotá, Colombia	Camilo A. Correa-Cárdenas; Carolina Oliveros; Claudia Méndez; Elizabeth K. Márquez; Frank de los Santos Ortiz; Juan David Ramírez; Julie Pérez; Lorena Albarracín; Luz H. Patiño; Maria Clara Duque; Marina Muñoz; María Teresa Alvarado; Nathalia Ballesteros; Sergio Castañeda; Sergio Gutíerrez-Riveros; Yanira Romero; Zulma Cucunubá
EPI_ISL_1273073, EPI_ISL_3241987, EPI_ISL_3355147	Guam Public Health Laboratory	Centers for Disease Control and Prevention Division of Viral Diseases, Pathogen Discovery	Alex Burgin; Anna Montmayeur; Anna Uehara; Ben L. Rambo-Martin; Ben Rambo-Martin; Clinton Paden; Clinton R. Paden; Dakota Howard; Dave Wentworth; Dhwani Batra; Haibin Wang; Jasmine Padilla; Jing Zhang; Justin Lee; Katie Dillon; Krista Queen; Kristen Knipe; Kristine Lacek; Lori Rowe; Mark Burroughs; Matthew Schmerer; Meghan Bentz; Mili Sheth; Peter Cook; Peter W. Cook; Rachel Marine; Sam Shepard; Sarah Nobles; Shoshona Le; Suxiang Tong; Vivien Dugan; Yan Li; Ying Tao; Yvette Unoarumhi
EPI_ISL_547610, EPI_ISL_2376254	Gundersen Clinical Microbiology Laboratory	Kabara Cancer Research Institute	Craig S. Richmond; Paraic A. Kenny
EPI_ISL_547722, EPI_ISL_660987	Gundersen Molecular Diagnostics Laboratory	Kabara Cancer Research Institute	Craig S. Richmond; Paraic A. Kenny
EPI_ISL_3636981	H Braga	Instituto Nacional de Saude (INSA)	Borges et al
EPI_ISL_1117012	H Garcia de Orta	Instituto Nacional de Saude (INSA)	Borges et al
EPI_ISL_693535	H Vila Franca Xira	Instituto Nacional de Saude (INSA)	Borges et al
EPI_ISL_1023463	HD Figueira Foz	Instituto Nacional de Saude (INSA)	Borges et al
EPI_ISL_2984904	HELEN JOSEPH LABORATORY	National Institute for Communicable Diseases of the National Health Laboratory Service	Amoako DG; Bhiman JN; Everatt J; Ismail A; Mahlangu B; Mnguni A; Mohale T; Ntuli N; Scheepers C
EPI_ISL_1400557, EPI_ISL_2385288, EPI_ISL_2899778	HELIX LLC	WHO National Influenza Centre Russian Federation	Andrey Komissarov; Anna Ivanova; Artem Fadeev; Daria Danilenko; Dmitry Bazhenov; Dmitry Lioznov; Elena Nabieva; Georgii Bazykin; Kirill Varchenko; Ksenia Safina; Kseniya Komissarova; Maria Pisareva; Maria Timofeeva; Mikhail Bakaev; Nikita Yolshin; Oula Mansour; Oula Masour; Tamila Musaeva; Veronika Eder
EPI_ISL_1117929	HG Pharma GmbH	Bergthaler laboratory, CeMM Research Center for Molecular Medicine of the Austrian Academy of Sciences	Andreas Bergthaler; Anna Schedl; Bekir Erguner; Benedikt Agerer; Christoph Bock; Jan Laine; Lukas Endler; Maelle Le Moing; Martin Senekowitsch; Michael Schuster; Thomas Penz
EPI_ISL_3536479, EPI_ISL_3536485	HLAGYN - Laboratorio de Imunologia de Transplantes de Goias	HLAGYN - Laboratorio de Imunologia de Transplantes de Goias	Alessandro Leonardo Alvares Magalhaes; Erika Lopes Rocha Batista; Fernando Antonio Vinhal dos Santos; Frederico Rodrigues Vinhal; Kamila Oliveira Reis De Freitas.; Lucas Carlos Gomes Pereira; Sabrina Sara Moreira Duarte
EPI_ISL_2611665	HOME QUARANTINE TASKFORCE	Hong Kong Department of Health	Alan K.L. Tsang; Dominic N.C. Tsang; Edman T.K. Lam; Ken H.L. Ng; Peter C.W. Yip; Rickjason C.W. Chan
EPI_ISL_3267066, EPI_ISL_3452811	HOPITAL NOSTRA SENYORA MERITXELL	CHU Purpan - Laboratoire de Virologie - Institut Fédératif de Biologie	Boyer P.; Carcenac R.; Ferrer V.; Harter A.; Izopet J.; Jeanne N.; Latour J.; Ranger N.; Tremeaux R.
EPI_ISL_1336649	HOPITAL PRINCESSE GRACE	CNR Virus des Infections Respiratoires - France SUD	Antonin Bal; Bruno Lina; Gregory Destras; Gwendolyne Burfin; Hadrien Regue; Laurence Josset; Martine Valette; Quentin Semanas
EPI_ISL_2674339	HOSPITAL COMUNAL MALVINAS	Instituto Nacional de Salud- Dirección de Investigación en Salud Pública	Carlos Franco-Muñoz; Carmen Osorio; Diana Malo; Diego A. Álvarez-Díaz; Diego Andrés Prada; Gerardo Santamaría; Hector Alejandro Ruiz-Moreno; Jhonnatan Reales-González; Jorge Rivera; Juan Camilo Martinez; Julian Naizaque; Katherine Laiton-Donato; Lisseth Pardo; Magdalena Wiesner; Marcela Mercado-Reyes; Maria T. Herrera-Sepúlveda; Marta Lopez Blanco; Martha Lucia Ospina Martinez; Paola Rojas; Sergio Gomez; Sheryll Corchuelo; Ángela Alarcon Cruz
EPI_ISL_2502738	HOSPITAL DR. MAX TERAN VALLS	Inciensa, Instituto Costarricense de InvestigaciOn y Enseñanza en NutriciOn y Salud	Adriana Godínez; Claudio Soto-Garita; Estela Cordero; Francisco Duarte; Hebleen Porras; Jose Luis Vargas; Joselyn Prado & María Jose GOmez-Umaña; Mariela Gutierrez; Melany CalderOn
EPI_ISL_3638835	HOSPITAL DR. RAFAEL A. CALDERON GUARDIA	Inciensa, Instituto Costarricense de Investigación y Enseñanza en Nutrición y Salud	Adriana Godínez; Claudio Soto-Garita; Estela Cordero; Francisco Duarte; Hebleen Porras; Joselyn Prado & Fabian Salas; José Luis Vargas; Mariela Gutiérrez; Melany Calderón
EPI_ISL_2502733	HOSPITAL GUAPILES	Inciensa, Instituto Costarricense de InvestigaciOn y Enseñanza en NutriciOn y Salud	Adriana Godínez; Claudio Soto-Garita; Estela Cordero; Francisco Duarte; Hebleen Porras; Jose Luis Vargas; Joselyn Prado & Cesar Cerdas-Quesada; Mariela Gutierrez; Melany CalderOn
EPI_ISL_2502729	HOSPITAL LA ANEXION	Inciensa, Instituto Costarricense de InvestigaciOn y Enseñanza en NutriciOn y Salud	Adriana Godínez; Claudio Soto-Garita; Estela Cordero; Francisco Duarte; Hebleen Porras; Jose Luis Vargas; Joselyn Prado & Ivanna Krize-Morún; Mariela Gutierrez; Melany CalderOn
EPI_ISL_3797835	HOSPITAL MANACOR	HOSPITAL UNIVERSITARIO SON ESPASES	Dr. Antonio Oliver; Dr. Carla López-Causapé; Dr. Gabriel Cabot; Hospital Universitario Son Espases; on behalf of Servicio de Microbiología
EPI_ISL_3037844, EPI_ISL_3274355	HOSPITAL METROPOLITANO	Inciensa, Instituto Costarricense de Investigación y Enseñanza en Nutrición y Salud	Adriana Godínez; Caterina Guzmán; Claudio Soto-Garita; Estela Cordero; Francisco Duarte; Hebleen Porras; José Luis Vargas; Mariela Gutiérrez Joselyn Prado; Melany Calderón; Nazareth Ruiz & Margarita Lee
EPI_ISL_2502746	HOSPITAL MEXICO	Inciensa, Instituto Costarricense de InvestigaciOn y Enseñanza en NutriciOn y Salud	Adriana Godínez; Claudio Soto-Garita; Estela Cordero; Francisco Duarte; Hebleen Porras; Jose Luis Vargas; Joselyn Prado & Juan Vilallobos; Mariela Gutierrez; Melany CalderOn
EPI_ISL_3639151	HOSPITAL MONSEÑOR SANABRIA	Inciensa, Instituto Costarricense de Investigación y Enseñanza en Nutrición y Salud	Adriana Godínez; Claudio Soto-Garita; Estela Cordero; Francisco Duarte; Hebleen Porras; Joselyn Prado & Andrea Moreno; José Luis Vargas; Mariela Gutiérrez; Melany Calderón
EPI_ISL_3761771, EPI_ISL_3761786	HOSPITAL NACIONAL DE NIÑOS	Inciensa, Instituto Costarricense de Investigación y Enseñanza en Nutrición y Salud	Cristian Pérez-Corrales & Valeria Peralta-Barquero
EPI_ISL_3639005	HOSPITAL SAN FRANCISCO DE ASIS	Inciensa, Instituto Costarricense de Investigación y Enseñanza en Nutrición y Salud	Adriana Godínez; Claudio Soto-Garita; Estela Cordero; Francisco Duarte; Hebleen Porras; Joselyn Prado & Adrián Fallas Mora; José Luis Vargas; Mariela Gutiérrez; Melany Calderón
EPI_ISL_2502724, EPI_ISL_2502760	HOSPITAL SAN JUAN DE DIOS	Inciensa, Instituto Costarricense de InvestigaciOn y Enseñanza en NutriciOn y Salud	Adriana Godínez; Claudio Soto-Garita; Estela Cordero; Francisco Duarte; Hebleen Porras; Jose Luis Vargas; Joselyn Prado & Andony Cordero-Jimenez; Joselyn Prado & Marco Cháves-Otárola; Mariela Gutierrez; Melany CalderOn
EPI_ISL_2103373, EPI_ISL_3037808	HOSPITAL SAN JUAN DE DIOS	Inciensa, Instituto Costarricense de Investigación y Enseñanza en Nutrición y Salud	Adriana Godinez & Melany Calderon; Adriana Godínez; Caterina Guzmán; Claudio Soto-Garita; Estela Cordero; Francisco Duarte; Hebleen Brenes; Hebleen Porras; José Luis Vargas; Mariela Gutiérrez Joselyn Prado; Melany Calderón; Nazareth Ruiz & Juliana Mora

EPI_ISL_3026021	HOSPITAL SAN RAFAEL DE ALAJUELA [ALAJUELA/ALAJUELA]	Inciensa, Instituto Costarricense de Investigación y Enseñanza en Nutrición y Salud	Adriana Godínez; Caterina Guzmán; Claudio Soto-Garita; Estela Cordero; Francisco Duarte; Hebleen Porras; Joselyn Prado; José Luis Vargas; Mariela Gutiérrez; Melany Calderón; Nazareth Ruíz & Christian Sanchez
EPI_ISL_3298345	HOSPITAL SAN VICENTE DE PAUL	Inciensa, Instituto Costarricense de Investigación y Enseñanza en Nutrición y Salud	Adriana Godínez; Claudio Soto-Garita; Estela Cordero; Francisco Duarte; Hebleen Porras; Joselyn Prado & Silvia Sáenz; José Luis Vargas; Mariela Gutiérrez; Melany Calderón
EPI_ISL_2096775	Hadassah Medical Center Clinical Virology Laboratory, Hadassah Ein Kerem	Hadassah Hebrew University Viral Sequencing Group, Hadassah Hebrew University Medical Center	Dana G. Wolf; Esther Oiknine-Djian; Hadar Golan Berman; Mila Rivkin; Sheera Adar
EPI_ISL_1919419, EPI_ISL_3542756, EPI_ISL_3797746, EPI_ISL_3834456	Haukeland University Hospital, Dept. of Microbiology	Norwegian Institute of Public Health, Department of Virology	Atiya R Ali; Debech Nadia; Engebretsen Serina Beate; Garcia Llorente Ignacio; Hilde Elshaug; Hilde Vollan; Jon Bråte; Kamilla Heddeland Instefjord; Karoline Bragstad; Kathrine Stene-Johansen; Line Victoria Moen; Marie Paulsen Madsen; Olav Hungnes; Pedersen Benedikte Nevjen; Rasmus Riis Kopperud
EPI_ISL_2938098	Health Center Novi Grad Sarajevo	Clinical Center, University of Sarajevo; Unit for Clinical Microbiology	Amela Dedeić-Ljubović; Edina Zahirović; Irma Salimović-Bešić; Sandra Vegar-Zubović; Sebija Izetbegović
EPI_ISL_796015	Hebei Provincial Center for Disease Control and Prevention, Shijiazhuang, Hebei Province; National Institute for Viral Disease Control and Prevention, China CDC	Hebei Provincial Center for Disease Control and Prevention, Shijiazhuang, Hebei Province; National Institute for Viral Disease Control and Prevention, China CDC	George F. Gao; Nankun Liu; Qi Li; Shunxiang Qi; Wenbo Xu; Xiang Zhao; Yang Song
EPI_ISL_1088999, EPI_ISL_1089203, EPI_ISL_1089366	Helix / Illumina	Respiratory Viruses Branch, Division of Viral Diseases, Centers for Disease Control and Prevention	Alexandre Bolze; Ary Ascencio; Ben L. Rambo-Martin; Brad Sickler; Charlotte Rivera-Garcia; Christine Tran; Clinton R. Paden; Dakota Howard; David Becker; Dhwani Batra; Duncan MacCannell; Efren Sandoval; Eileen de Feo; Elizabeth Cirulli; Eric Allen; Geraint Levan; James Lu; Jan Antico; Jason Nguyen; Jimmy Ramirez; Jingtao Liu; Kelly Schiabor Barrett; Kim Gietzen; Magnus Isaksson; Marc Laurent; Matthew Tolentino; Nicole L. Washington; Peter W. Cook; Phil Febbo; Ryan Cho; Shannon Wickline; Sherry Wang; Simon White; Summer Galloway; Suxiang Tong; Tyler Cassens; William Lee
EPI_ISL_2159479, EPI_ISL_2159835, EPI_ISL_2159875, EPI_ISL_2159947, EPI_ISL_2160000, EPI_ISL_2247823, EPI_ISL_2248033, EPI_ISL_2248323, EPI_ISL_2270556			
see above	Helix/Illumina	Centers for Disease Control and Prevention Division of Viral Diseases, Pathogen Discovery	Adrian Paskey; Alexandre Bolze; Ary Ascencio; Benjamin Rambo-Martin; Brad Sickler; Charlotte Rivera-Garcia; Christine Tran; Christopher Gulvick; Clinton R. Paden; Dakota Howard; Darlene Wagner; David Becker; Dhwani Batra; Duncan MacCannell; Efren Sandoval; Eileen de Feo; Elizabeth Cirulli; Eric Allen; Geraint Levan; James Lu; Jan Antico; Jason Caravas; Jason Nguyen; Jimmy Ramirez; Jingtao Liu; Kara Moser; Kelly Schiabor Barrett; Kim Gietzen; Magnus Isaksson; Marc Laurent; Matthew Schmerer; Matthew Tolentino; Nicole L. Washington; Peter W. Cook; Phil Febbo; Ryan Cho; Scott Sammons; Shannon Wickline; Shatavia Morrison; Sherry Wang; Simon White; Tyler Cassens; William Lee; Yvette Unoarumhi
EPI_ISL_755616	Helix/Illumina	Genomics and Discovery, Respiratory Viruses Branch, Division of Viral Diseases, Centers for Disease Control and Prevention	Alexandre Bolze; Ary Ascencio; Brad Sickler; Charlotte Rivera-Garcia; Christine Tran; Clinton R. Paden; David Becker; Dhwani Batra; Duncan MacCannell; Efren Sandoval; Eileen de Feo; Elizabeth Cirulli; Eric Allen; Geraint Levan; James Lu; Jan Antico; Jason Nguyen; Jimmy Ramirez; Jingtao Liu; Kelly Schiabor Barrett; Kim Gietzen; Magnus Isaksson; Marc Laurent; Matthew Tolentino; Nicole L. Washington; Peter W. Cook; Phil Febbo; Ryan Cho; Shannon Wickline; Sherry Wang; Simon White; Summer Galloway; Suxiang Tong; Tyler Cassens; William Lee
EPI_ISL_3689330	Hellenic National Blood Transfusion Center - EKEA	Greek Genome Center, Biomedical Research Foundation of the Academy of Athens (BRFAA)	Dimitrios Thanos; Efthimia Petinaki; Emmanouil Athanasiadis; Giannis Vatsellas; Katerina Zoi; Kostas Stamoulis; Theodoros Loupis
EPI_ISL_700051, EPI_ISL_723048	Hematopathology Laboratory, ACTREC, TMC	Hematopathology Laboratory, ACTREC, TMC	ACTREC; Hematopathology Laboratory
EPI_ISL_2678286	HerpeZ	Institute of Tropical Medicine	Alimuddin Zumla; Edgar Simulundu; Franklyn Egbe Nkongho; John Tembo; Kangwa Mulonga; Kwitaka Maluzi; Le Thi Kieu Linh; Lloyd Mulenga; Matthew Bates; Moses Chilufya; Nathan Kapata; Prof. Dr. Thirumalaisamy P Velavan; Sivaramakrishna Rachakonda; Sombo Fwoloshi; Srinivas Reddy Pallerla; Victor Mukonka
EPI_ISL_2811991, EPI_ISL_3502483, EPI_ISL_3833786, EPI_ISL_3834069	Histopath	NSW Health Pathology - Institute of Clinical Pathology and Medical Research; Westmead Hospital; University of Sydney	Arnott A.; CIDM-PH et al.; Draper J.; Gall M.; Martinez E.; Rockett R.; Sintchenko V.; on behalf of ICPMR
EPI_ISL_3547107	Home Quarantine Taskforce	Hong Kong Department of Health	Alan K.L. Tsang; Edman T.K. Lam; Ken H.L. Ng; Peter C.W. Yip; Rickjason C.W. Chan
EPI_ISL_412029	Hong Kong Department of Health	The University of Hong Kong	Daniel K.W. Chu; Dominic N.C. Tsang; Leo L.M. Poon; Malik Peiris
EPI_ISL_3149731, EPI_ISL_3825477	Hosp. Pediatrico Dr. Avelino Castelán	Laboratorio de Biología Molecular, Instituto de Medicina Regional on behalf of 'Proyecto Argentino Interinstitucional de genomica de SARS-CoV-2' (PAIS Consortium)	Bettina Brusés; Gioia Lucia Marino; Griselda Oria; Horacio Lucero.; Javier Mussin; Julieta Ronchi; Laura Formichelli; María Andrea Gili; María Cecilia López; Melina Lorenzini Campos; Raúl Maximiliano Acevedo
EPI_ISL_2462907, EPI_ISL_2462927, EPI_ISL_2532300, EPI_ISL_2628275, EPI_ISL_2757668, EPI_ISL_2790982, EPI_ISL_2790983, EPI_ISL_2835907, EPI_ISL_3058248, EPI_ISL_3542891, EPI_ISL_3542957, EPI_ISL_3542958, EPI_ISL_3542964, EPI_ISL_3690259, EPI_ISL_3801691			
see above	Hospital	National Reference Center for Viruses of Respiratory Infections, Institut Pasteur, Paris	Amaury Vaysse; Angela Brisebarre; Antoine Talarmin; Camille Capel; Christophe Malabat; Corinne Maufrais; CéCile Hermann; Didier Mattera; Emmanuelle Permal; Etienne Simon-Lorière; Frédéric Lemoine; Hub Bioinformatique Biostatistiques; Hub de Bioinformatique et Biostatistique; JéRôMe Durivault; Louise Lefrançois; MOUDJAHED Haciba; Marion Barbet; Maud Vanpeene; Méline Bizard; Olivier Dejoux; Sylvaine Bastian; Sylvie Behillil; Sylvie Van der Werf; Vincent Enouf
EPI_ISL_1383905	Hospital Center Emile Mayrisch	Laboratoire national de sante, Microbiology, Microbial Genomics Platform	Anke Wienecke-Baldacchino; Catherine Ragimbeau; Cynthia Oxacelay; Fatu Djabi; Jessica Tapp; Lise Pignon; Raoul Salmon; Tamir Abdelrahman
EPI_ISL_2965568, EPI_ISL_2965583, EPI_ISL_2965584, EPI_ISL_2965585	Hospital Español	Centro de Innovación en Vigilancia Epidemiológica (CiVE), Institut Pasteur Montevideo, Uruguay	Alicia Costábile; Alvaro Fajardo; Ana Moller; Andrés Lizasoain; Belén González; Bernardina Rivera; Cecilia Alonso; Cecilia Salazar; Gonzalo Bello; Gonzalo Moratorio; Gregorio Iraola; Henry Albornoz; Ignacio Ferrés; Javier Hurtado; Juan Zanetti; Julio Medina; Luciana Griffero; Lucía Spangenberg; Ma Noel Bentancor; Ma Pía Techera; Mailen Arleo; Martina Alonso; Matías Maidana; Mauricio Méndez; Melissa Duquía; Mercedes Paz; Natalia Rego; Natalia Reyes; Nicolas Nin; Odhille Chappos; Paula Perbolianachis; Pilar Moreno; Rodney Colina; Rodrigo Arce; Tamara Fernández-Calero; Tania Possi; Veronica Noya; Viviana Bortagaray
EPI_ISL_3671013	Hospital Fundacion San Vicente de Paul	Universidad Nacional de Colombia - Laboratorio Genómico One Health	Andres F. Cardona-Rios; Carlos Franco-Muñoz; Carolina Muñoz-Arango; Celeny Ortiz; Daniel O. Maldonado-Perez; Diego A. Álvarez-Díaz; Hector Alejandro Ruiz-Moreno; Idabely Betancur Ortiz; Jorge E. Osorio; Juan P. Hernandez-Ortiz; Karl A Ciuoderis; Katherine Laiton-Donato; Laura Silvana Perez; Lina M. Hurtado; Marcela Mercado-Reyes; Maria Angélica Maya; Maria Stella López; Rita Almanza Payares; Sandra Ines Cano; Simón Villegas Velásquez
EPI_ISL_2402511, EPI_ISL_3731081	Hospital General Universitario Gregorio Marañón	Hospital General Universitario Gregorio Marañón	Cristina Rodriguez-Grande; Darío García de Viedma; Julia Suárez; Laura Pérez-Lago; Marta Herranz Martin; Patricia Muñoz; Pedro Sola Campoy; Pilar Catalán; Sergio Buenestado Serrano; Victor Manuel de la Cueva
EPI_ISL_2567551	Hospital General Universitario de Alicante - Instituto de Investigación Sanitaria y Biomédica de Alicante	SeqCOVID-SPAIN consortium/IBV(CSIC)	Carmen Molina Pardines and SeqCOVID-SPAIN consortium; Maripaz Ventero Martín
EPI_ISL_471268	Hospital IESS Babahoyo	Institute of Microbiology, Universidad San Francisco de Quito	Belén Prado-Vivar; Bernardo Gutiérrez; Francisco Cordova; Gabriel Trueba; Juan José Guadalupe; Killen Briones-Claudette; Killen Briones-Zamora; Michelle Grunauer; Ninfa Henriquez; Patricio Rojas-Silva; Paúl Cárdenas; Sully Márquez; Verónica Barragán
EPI_ISL_3033082, EPI_ISL_3260092, EPI_ISL_3260095, EPI_ISL_3260109, EPI_ISL_3260111, EPI_ISL_3260112	Hospital Jaime Ferre - SAMCO Rafaela	Grupo de Genómica y Bioinformática del Instituto de Investigación de la Cadena Láctea CONICET-INTA on behalf of 'Proyecto Argentino Interinstitucional de genomica de SARS-CoV-2' (PAIS Consortium)	AF; Aliprandi D; Amadio; C; Eberhardt; Irazoqui; Isaia; JF; JM; MF; Pandolfi; Quaranta; Soratti R; V
EPI_ISL_2405164	Hospital Luis Vernaza	Omics Sciences Laboratory	Darlyn Amaya; Derly Andrade Molina; Gabriel Morey León; Juan Carlos Fernández Cadena; Rubén Armas González
EPI_ISL_2894559	Hospital Margarita Maza de Juárez	Microbial Genomics Laboratory	; Alejandra García-Gasca; Alejandra Hernández-Terán; Alejandro Sánchez-Flores; Alfredo Herrera-Estrella; Alicia Ocaña-Mondragón; Andreu Comas-García; Angel Gustavo Salas-Lais; Antonio Loza Román; Bernardo Martínez-Miguel; Blanca Taboada; Brenda Irasema Maldonado-Meza; Bruno Gómez-Gil; Carla Ivón Herrera-Najera; Carlos F. Arias; Celia Boukadida; Clara Esperanza Santacruz-Tinoco; Concepción Grajales-Muñiz; Consorcio Mexicano de Vigilancia Genómica (CoViGen-Mex). Authors (in alphabetical order): Julio Elias Alvarado-Yaah; Cristóbal Cháidez-Quiróz; Célida Duque Molina; Célida Martínez- Rodríguez; Daniel Fregoso-Rueda; Daniel Lira Morales; Eduardo Becerril-Vargas; Fernando Fontove-Herrera; Fidencio Mejía-Nepomuceno; Francisco Pulido; Gloria Elena Espinosa-Ayala; Gloria María Molina-Salinas; Gloria Vazquez; Hector Esteban Paz-Juárez; Hector Montoya-Fuentes; Helen Haydee Fernanda Ramírez-Plascencia; Irvin González-López; Jean Pierre González; Jesús Hernández; Joel Armando Vázquez-Pérez.; Jorge Salas-Hernández; José Antonio Enciso-Moreno; José Arturo Martínez-Orozco; José Esteban Muñoz-Medina; José de Jesús Nuñez-Contreras; Juan Bautista Chale-Dzul; Julissa Enciso-Ibarra; Luis Alberto Ochoa-Carrera; Margarita Matías-Florentino; Mario Mújica-Sánchez; Marissa Perez-Garcia; María Guadalupe Santiago-Mauricio; María Guadalupe de Jesús Mireles-Rivera; Nelly Sélem-Mojica; Pavel Isa; Ricardo Ciria Merce; Ricardo Grande; Rosa María Gutiérrez Rios; Santiago Ávila-Ríos; Selene Zárate; Susana Lopez; Verónica Mata-Haro; Victor Eduardo García-Arias; Víctor Hugo Borja-Aburto
EPI_ISL_491453	Hospital México	Inciensa, Instituto Costarricense de Investigación y Enseñanza en Nutrición y Salud	Adriana Godinez & Melany Calderon; Claudio Soto-Garita; Estela Cordero; Francisco Duarte; Hebleen Brenes
EPI_ISL_480327	Hospital Nacional de Niños	Charité Virology-University of Costa Rica	Andrei Montero Bonilla; Andres Moreira-Soto; Cristian Pérez Corrales; Eugenia Corrales-Aguilar; Ignacio Postigo-Hidalgo; Jan Felix Drexler
EPI_ISL_539496	Hospital Nostra Senyora de Meritxell	Instituto de Salud Carlos III	A. Monzón; F. Casas; F. Fernández; I; I. Jiménez; Iglesias-Caballero; M. Camarero; M. Cuesta; M. González-Esguevillas; M. Molinero Calamita; M. Zaballos; P. Jiménez; S. Juliá; S. Pozo; S. Varona
EPI_ISL_1391104	Hospital Universitari Vall d'Hebron - Vall d'Hebron Institut de Recerca	Hospital Universitari Vall d'Hebron - Vall d'Hebron Institut de Recerca	Andrés Antón; Ariadna Rando; Carla Castillo; Cristina Andrés; Damir Garcia-Cehic; Josep F Abril; Josep Quer; Juliana Esperalba; Maria Carmen Martin; Maria Gema Codina; Maria Piñana; Tomàs Pumarola
EPI_ISL_3008492	Hospital Universitari i Politècnic La Fe de València	SeqCOVID-SPAIN consortium/IBV(CSIC)	Ana Gil Brusola; Eva González Barberá; José Luis López Hontangas and SeqCOVID-SPAIN consortium; María Dolores Gómez Ruiz; Salvador Giner Almaraz
EPI_ISL_1120939	Hospital Universitario 12 de Octubre	SeqCOVID-SPAIN consortium/IBV(CSIC)	Esther Viedma; Irene Múñoz-Gallego; Jennifer Villa; Mª Dolores Folgueira and SeqCOVID-SPAIN consortiumconsortium; Rafael Delgado; Raúl Recio
EPI_ISL_510445	Hospital Universitario Virgen de las Nieves de Granada-SAS	SeqCOVID-SPAIN consortium/IBV(CSIC)	Irene Pedrosa Corral; José M. Navarro-Marí and SeqCOVID-SPAIN consortium; Mercedes Pérez Ruiz; Sara Sanbonmatsu Gámez
EPI_ISL_2135353	Hospital Universitario de La Ribera (Alzira, València)	SeqCOVID-SPAIN consortium/IBV(CSIC)	Julia González Cantó and SeqCOVID-SPAIN consortium; Olalla Martínez Macias
EPI_ISL_458028	Hospital for Tropical Diseases	COVID-19 Network Investigations (CONI) Alliance	Akanitt Jittmittraphap; Angkana Huang; Anthony R. Jones; Arporn Wangwiwatsin; Bhakbhoom Panthan; Chonticha Klungtong; Ekawat Pasomsub; Elizabeth Batty; Insee Sensorn; Janjira Thaipadungpanit; Khajohn Joonsalak; Kingkan Rakmanee; Krittikorn Kumpornsin; Namfon Kotanan; Nantarat Chantawat; Pornsawan Leaungwutiwong; Stefan Fernandez; Thanat Chookajorn; Theerarat Kochakarn; Treewat Watthanachockchai; Wasun Chantratita; Weena Janwitthayanan; Wudtichai Manasatienkij
EPI_ISL_2357830	Hospital of Southern Norway - Kristiansand, Department of Medical Microbiology	Norwegian Institute of Public Health, Department of Virology	Atiya R Ali; Debech Nadia; Engebretsen Serina Beate; Garcia Llorente Ignacio; Hilde Elshaug; Hilde Vollan; Jon Bråte; Kamilla Heddeland Instefjord; Karoline Bragstad; Kathrine Stene-Johansen; Marie Paulsen Madsen; Olav Hungnes; Pedersen Benedikte Nevjen; Rasmus Riis Kopperud
EPI_ISL_3023084	Hospital of the University of Pennsylvania Molecular Pathology Lab	Bushman Lab - University of Pennsylvania	Aoife M. Roche; Arupa Ganguly; Ayannah S. Fitzgerald; Brendan Kelly; Jevon Graham-Wooten; John K. Everett; Kyle Rodino; Layla A. Khatib; Mike Feldman; Ronald G. Collman and Frederic Bushan; Samantha A. Whiteside; Scott Sherrill-Mix; Shantan Reddy; Young Hwang
EPI_ISL_2221072	Houston Methodist Hospital	Houston Methodist Hospital	Ilya J. Finkelstein; James J. Davis; Jessica Cambric; Jimmy Gollihar; Kristina Reppond; Layne Pruitt; Madison N. Shyer; Marcus Nguyen; Matthew Ojeda Saavedra; Paul A. Christensen; Prasanti Yerramilli; Randall J. Olsen; Robert Olson; Ryan Gadd; S. Wesley Long; Sishir Subedi; and

			James M. Musser
EPI_ISL_2674799	Hrvatski zavod za javno zdravstvo	Hrvatski zavod za javno zdravstvo	Irena Tabain; Ivana Ferenčak
EPI_ISL_2466657	Human Genetic Research Center, Kawsar Biotech Company	Kawsar Biotech Company	Abbasalipour, M.; Azadmanesh, K.; Chamran Hospital, C.; Khosravi; M.A.; National Institute for Medical Research Development, N.; Zeinali, R.; Zeinali, S.
EPI_ISL_526224	Hungarian Defence Forces Military Medical Centre	National Laboratory of Virology, Szentágothai Research Centre	Balázs Somogyi; Bálint Eszenyi; Endre Gábor Tóth; Ferenc Jakab; Gábor Kemenesi
EPI_ISL_940539	Hôpital Bichat Claude Bernard, Laboratoire de Virologie	IAME UMR1137 Inserm, Université de Paris, Hôpital Bichat	Alexandre Storto; Amélie Recoing; Antoine Bridier-Nahmias; Benoit Visseaux; Charlotte Charpentier; Diane Descamps; Gilles Collin; Lena Daniel; Mélanie Bertine; Nadhira Houhou-Fidouh; Quentin Le Hingrat; Siham Hamri
EPI_ISL_2228879	Hôpital Cochin	Department of Virology, Henri Mondor University Hospital, Assistance Publique Hôpitaux de Paris, Université Paris-Est Créteil, INSERM U955	Alexandre Soulier; Christophe Rodriguez; Elisabeth Trawinski; Guillaume Gricourt; Jean-Michel Pawlotsky; Melissa N'Debi; Slim Fourati; Vanessa Demontant
EPI_ISL_961667	Hôpital Georges L. Dumont	National Microbiology Laboratory (NML)	Anna Majer; Anneliese Landgraff; CanCOGeN's metadata curation team; Darian Hole; Elsie Grudeski; Gary Van Domselaar; Grace Seo; Guillaume Desnoyers; Jennifer Tanner; Kirsten Biggar; Madison Chapel; Morag Graham; Natalie Knox; Nathalie Bastien; Philip Mabon; Public Health Agency of Canada CanCOGeN team; Rhiannon Huzarewich; Richard Garceau; Russell Mandes; Shari Tyson; Timothy Booth; Yan Li
EPI_ISL_535716	Hôpital de Verdun	Laboratoire de santé publique du Québec	Guillaume Bourque; Ioannis Ragoussis; Jesse Shapiro; Mark Lathrop and Michel Roger on behalf of the CoVSeQ research group; Sandrine Moreira
EPI_ISL_3127986	IA State Hygienic Laboratory	Centers for Disease Control and Prevention Division of Viral Diseases, Pathogen Discovery	Alex Burgin; Ben L. Rambo-Martin; Clinton R. Paden; Dakota Howard; Dave Wentworth; Dhwani Batra; Jasmine Padilla; Justin Lee; Krista Queen; Kristen Knipe; Kristine Lacek; Mark Burroughs; Matthew Schmerer; Meghan Bentz; Mili Sheth; Peter Cook; Sam Shepard; Sarah Nobles; Suxiang Tong; Vivien Dugan; Yvette Unoarumhi
EPI_ISL_1094329	IA State Hygienic Laboratory	Respiratory Viruses Branch, Division of Viral Diseases, Centers for Disease Control and Prevention	Anna Montmayeur; Anna Uehara; Ben L. Rambo-Martin; Clinton R. Paden; Dhwani Batra; Haibin Wang; Jasmine Padilla; Jing Zhang; Justin Lee; Krista Queen; Lori Rowe; Mark Burroughs; Mili Sheth; Peter W. Cook; Rachel Marine; Sarah Nobles; Suxiang Tong; Yan Li; Ying Tao
EPI_ISL_1704103, EPI_ISL_3533062	ICMR-National Institute of Virology - INSACOG	NIV Influenza	Dr. Varsha Potdar; Dr. Varsha Potdar and NIC Team
EPI_ISL_3355289	ID Bureau of Laboratories	Centers for Disease Control and Prevention Division of Viral Diseases, Pathogen Discovery	Alex Burgin; Ben Rambo-Martin; Clinton Paden; Dakota Howard; Dave Wentworth; Dhwani Batra; Jasmine Padilla; Justin Lee; Krista Queen; Kristen Knipe; Kristine Lacek; Mark Burroughs; Matthew Schmerer; Meghan Bentz; Mili Sheth; Peter Cook; Sam Shepard; Sarah Nobles; Suxiang Tong; Vivien Dugan; Yvette Unoarumhi
EPI_ISL_2811914	IDF medical corps	Central Virology Laboratory, Ministry of Health	Amos Adler; Efrat Bucris; Ella Mendelson; Michal Mandelboim; Moran Shwartswort-Cohen; Neta S. Zuckerman; Noam Protter; Oran Erster; Orna Mor; Saar Burstein
EPI_ISL_2444803, EPI_ISL_2444813, EPI_ISL_2444814, EPI_ISL_2444815, EPI_ISL_2444817, EPI_ISL_2444819, EPI_ISL_2444820, EPI_ISL_2444821, EPI_ISL_2444822, EPI_ISL_2444824			
see above	IICS-UNA	IICS-UNA	Adriana Valenzuela; Alejandra Rojas; Chyntia Diaz; Eva Nara; Fatima Cardozo; Florencia del Puerto; Joel Ortiz; Jonas Fernandez; Laura Franco; Laura Mendoza; Leticia Rojas; Magaly Martinez; Maria Eugenia Galeano.
EPI_ISL_1225990	IN State Department of Health Laboratory Services	Genomics and Discovery, Respiratory Viruses Branch, Division of Viral Diseases, Centers for Disease Control and Prevention	Anna Montmayeur; Anna Uehara; Ben L. Rambo-Martin; Clinton R. Paden; Dhwani Batra; Haibin Wang; Jasmine Padilla; Jing Zhang; Justin Lee; Katie Dillon; Krista Queen; Kristen Knipe; Kristine Lacek; Lori Rowe; Mark Burroughs; Matthew Schmerer; Mili Sheth; Peter W. Cook; Rachel Marine; Sam Shepard; Sarah Nobles; Shoshona Le; Suxiang Tong; Yan Li; Ying Tao
EPI_ISL_1577798, EPI_ISL_1577815, EPI_ISL_2628194, EPI_ISL_2628307	INHRR	Laboratorio de Virología Molecular	Aguilar M; Alarcon V; Carmen L Loureiro; CoViVen Group; D Angelo P; Delgado M; Domingo J Garzaro; Esmeralda Vizzi; Flor H Pujol; Garzaro D; Héctor R Rangel; Jaspe RC; José Luis Zambrano; Lieska Rodríguez; Loureiro CL; Mariana Hidalgo; Pierina D´Angelo; Pujol FH; Rangel HR; Rodriguez L; Rossana C Jaspe; Víctor Alarcón; Yoneira Sulbaran; Zambrano JL; Zoila Moros
EPI_ISL_410546	INMI Lazzaro Spallanzani IRCCS	Laboratory of Virology, INMI Lazzaro Spallanzani IRCCS	Barbara Bartolini; Cesare E. M. Gruber; Concetta Castilletti; Eleonora Lalle; Emanuela Giombini; Emanuele Nicastri; Fabrizio Carletti; Francesca Colavita; Francesco Messina; Giuseppe Ippolito.; Maria R. Capobianchi; Martina Rueca
EPI_ISL_3459414	INS	Instituto Nacional de Salud	Carlos Franco-Muñoz; Carmen Osorio; Diana Malo; Diego A. Álvarez-Díaz; Diego Andrés Prada; Gerardo Santamaría; Hector Alejandro Ruiz-Moreno; Jhonnatan Reales-González; Jorge Rivera; Juan Camilo Martinez; Julian Naizaque; Katherine Laiton-Donato; Lisseth Pardo; Magdalena Wiesner; Marcela Mercado-Reyes; Maria T. Herrera-Sepúlveda; Marta Lopez Blanco; Martha Lucia Ospina Martinez; Paola Rojas; Sergio Gomez; Sheryll Corchuelo; Ángela Alarcon Cruz
EPI_ISL_421499	INSA	Instituto Nacional de Saude (INSA)	Guiomar et al
EPI_ISL_2878662	INSACOG Surveillance	INSACOG at CSIR Institute of Genomics and Integrative Biology	INSACOG
EPI_ISL_3021944	INSACOG-MANIPUR	National Institute of Biomedical Genomics – INSACOG	Arindam Maitra; Kh. Ranjana Devi; L. Shivadutta Singh; Nidhan Kumar Biswas; R.K.Manojkumar Singh; Saumitra Das; Sreedhar Chinnaswamy
EPI_ISL_1419106	INSACOG-Mizoram	National Institute of Biomedical Genomics – INSACOG	Arindam Maitra; Gracy Laldinmawii; N Senthil Kumar; Nidhan Kumar Biswas; Saumitra Das; Sreedhar Chinnaswamy; Swagnik Roy
EPI_ISL_3019017	INSACOG-Sikkim	National Institute of Biomedical Genomics – INSACOG	Arindam Maitra; Kaden Zangmu Bhutia; Nidhan Kumar Biswas; Saumitra Das; Sreedhar Chinnaswamy Tshering Ongmu Bhutia; Tara D Sharma
EPI_ISL_1419175, EPI_ISL_1419280, EPI_ISL_1419495	INSACOG-WB	National Institute of Biomedical Genomics – INSACOG	Ajay Chakraborti; Arindam Maitra; Bhaswati Bandyopadhyay; Nidhan Kumar Biswas; Saumitra Das; Sreedhar Chinnaswamy; Tamal Ghosh
EPI_ISL_3246108, EPI_ISL_3246116	INSIDE DIAGNÓSTICOS	Instituto Butantan	Antonio Jorge Martins; Claudia Renata dos Santos Barros; David Schlesinger; Debora Botequio Moretti; Dimas Tadeu Covas; Elaine Cristina Marqueze; Elaine Vieira Santos; Evandra Strazza Rodrigues; Heidge Fukumasu; Jayme Augusto de Souza-Neto; José Salvatore Leister Patané; Luiz Alcantara; Luiz Lehmann Coutinho; Maria Carolina Elias; Maurício Lacerda Nogueira; Rafael dos Santos Bezerra; Raul Machado Neto; Rejane Maria Tommasini Grotto; Ricardo Haddad; Sandra Coccuzzo Sampaio Vessoni; Simone Kashima; Svetoslav Nanev Slavov; Vincent Louis Viala
EPI_ISL_2672993	INSP, Bamako	Malaria Research and Training Center-Parasito	Abdoulaye Djimde; Amadou Daou; Antoine Dara; Demba Koita; Ibrehima Guindo
EPI_ISL_826818	INSPI-CRN DE INFLUENZA Y OTROS VIRUS RESPIRATORIOS	Instituto de Salud Publica de Chile	Alfredo Bruno; Andres Castillo; Barbara Parra; Domenica de Mora; Gisselle Barra; Jaime Lagos; Javier Tognarelli; Jimmy Garcez; Jorge Fernandez; Loredana Arata; Manuel Gonzalez; Martiza Olmedo; Michelle Paez; Patricia Bustos; Rodrigo Fasce; Solon Narvaez
EPI_ISL_2488753, EPI_ISL_2757585, EPI_ISL_2757587, EPI_ISL_2757597, EPI_ISL_2757633, EPI_ISL_2895670, EPI_ISL_2988459, EPI_ISL_2988467, EPI_ISL_2988472, EPI_ISL_2988484, EPI_ISL_2988487, EPI_ISL_2988546, EPI_ISL_3010462, EPI_ISL_3274402, EPI_ISL_3274428			
see above	INSPI-CRN DE INFLUENZA Y OTROS VIRUS RESPIRATORIOS	NIC-INSPI	Alfredo Bruno; Domenica de Mora.; Jimmy Garcés; Johanna Laines; Lizbeth Patiño; Manuel Gonzalez; Maritza Olmedo; Michelle Páez
EPI_ISL_3845830	INTERLAB	Omics Sciences Laboratory	Darlyn Amaya; Derly Andrade Molina; Gabriel Morey León; Juan Carlos Fernández Cadena; Rubén Armas González
EPI_ISL_751445	IRCCS Sacro Cuore Don Calabria Hospital, Department of Infectious, Tropical Diseases & Microbiology	University of Verona, Department of Biotechnology	Antonio Mori; Chiara Degli Esposti; Chiara Piubelli; Cristina Beltrami; Elena Pomari; Emanuela Cosentino; Giulia Lopatriello; Luca Marcolungo; Massimo Delledonne; Michela Deiana
EPI_ISL_1630261	IRESSEF	Abbott	Adbou Padane; Ambroise Ahouidi; Aminata Dia; Aminata Mboup; Ana Olivo; Anna julienne selbe Ndiaye; Barbara Harris; Cyrille Diedhiou; Gavin Cloherty; Mary Rodgers; Moustapha Mbow; Nafissatou Leye; Ndeye Diabou Diagne; Papa Alassane Diaw; Souleymane Mboup; Todd Meyer
EPI_ISL_2015111	IU-Cerrahpasa, Cerrahpasa School of Medicine, COVID-19 Lab	IU-Cerrahpasa, Cerrahpasa School of Medicine, COVID-19 Lab	Kenan Midilli; Mert Kuskucu; Yesim Tuyji Tok
EPI_ISL_2628209, EPI_ISL_2628299, EPI_ISL_2628300, EPI_ISL_2628302, EPI_ISL_2628305, EPI_ISL_2628308, EPI_ISL_2628310, EPI_ISL_3298687			
see above	IVIC	Laboratorio de Virología Molecular	Carmen L Loureiro; CoViMol Group; CoViVen Group; Domingo J Garzaro; Esmeralda Vizzi; Flor H Pujol; Héctor R Rangel; José Luis Zambrano; Lieska Rodríguez; Mariana Hidalgo; Pierina D´Angelo; Rossana C Jaspe; Víctor Alarcón; Yoneira Sulbaran; Zoila Moros
EPI_ISL_3272471	Idaho Bureau of Laboratories	Boise VA Medical Center, PALMS	Aimee Ceniseros; Cheri Lamb McFarlane; Christian Loera; Dr. Christopher Ball; Emily Bartlett; James Razor; Matthew Burns; Ying Pei
EPI_ISL_632881, EPI_ISL_2095700	Idaho Bureau of Laboratories	Center for Global Health, University of New Mexico Health Sciences Center	Christopher Ball; Darrell Dinwiddie; Daryl Domman; Kurt Schwalm; Matthew Burns; Robert Voermans; Valerie Morley
EPI_ISL_2090634, EPI_ISL_3064181, EPI_ISL_3064205, EPI_ISL_3756692	Idaho Bureau of Laboratories	Idaho Bureau of Laboratories	"R. Beukelman; Aimee Ceniseros; Christian Loera; Christopher Ball; Christopher Ball"; Matthew Charles Burns; R. Beukelman; Robert L. Voermans
EPI_ISL_2801167	Illinois Department of Public Health	Gagnon Lab, Southern Illinois University	Keith Gagnon
EPI_ISL_2800760	Illinois Department of Public Health	Illinois Department of Public Health - Chicago Lab	Ira Heimler; Joel Price; Vineet K. Dhiman
EPI_ISL_1063798	Immunogenomics lab, Institute of Life Sciences, Bhubaneswar	Immunogenomics lab, Institute of Life Sciences, Bhubaneswar	Ajay Parida; Amol M. Kanampalliwar; Arup Ghosh; Atimukta Jha; INSACOG Consortium; Kirtal Hansdah; Punit Prasad; Rajeeb Swain; Rupesh Dash; Safal Walia; Shifu Aggarwal; Sunil K. Raghav
EPI_ISL_1517401, EPI_ISL_1517429	Inciensa, Instituto Costarricense de Investigación y Enseñanza en Nutrición y Salud	Inciensa, Instituto Costarricense de Investigación y Enseñanza en Nutrición y Salud	Barboza-Arguedas E & Blanco-Delgado C; Cristian Peréz-Corrales; Cristian Peréz-Corrales & Valeria Peralta-Barquero
EPI_ISL_476704	Incubadora Venezolana de Ciencia, Venezuela	Incubadora Venezolana de Ciencia, Venezuela / Instituto Nacional de Salud, Bogotá, Colombia / Grupo de Investigaciones Microbiológicas-UR (GIMUR), Departamento de Biología, Facultad de Ciencias Naturales, Universidad del Rosario, Bogotá, Colombia / Icahn School of Medicine at Mount Sinai, New York, USA	Alberto Paniz-Mondolfi; Ana S. Gonzalez-Reiche; Angelica Rico; Aníbal A. Teherán; Carolina Florez; Carolina Hernández; Emilia Mia Sordillo; Esther C. Barros; Harm van Bakel; Jesús E. Jaimes; Juan David Ramírez; Lisseth Pardo; Lourdes Delgado; Luis Perez-Garcia; Marina Muñoz; Matthew M. Hernandez; Sergio Gomez; Viviana Simon
EPI_ISL_647986	Indiana State Department of Health	Pathogen Discovery, Respiratory Viruses Branch, Division of Viral Diseases, Centers for Disease Control and Prevention	Anna Montmayeur; Anna Uehara; Brian Lynch; Clinton R. Paden; Haibin Wang; Jing Zhang; Krista Queen; Rachel Marine; Suxiang Tong; Yan Li; Ying Tao
EPI_ISL_3540049, EPI_ISL_3540059,	Indira Gandhi Memorial Hospital	Indiar Gandhi Memorial Hospital	D. Fathmath Nazla Rafeeq; Mr. Ibrahim Nishan Ahmed; Ms. Aishath Shuhudha; Ms. Aminath Nazfa; Ms. Fathimath Zimna

EPI_ISL_3540060, EPI_ISL_3540070, EPI_ISL_3540079, EPI_ISL_3540080			
EPI_ISL_3275174, EPI_ISL_3275384, EPI_ISL_3275385, EPI_ISL_3452722, EPI_ISL_3452723, EPI_ISL_3452727	Indira Gandhi Memorial Hospital	Indira Gandhi Memorial Hospital	Dr. Milza Abdul Muhsin; Mr. Ibrahim Nishan Ahmed; Ms. Aishath Shuhudha; Ms. Aminath Nazfa; Ms. Fathimath Zimna
EPI_ISL_3000158, EPI_ISL_3003644	Infection Prevention & Control Unit	Central Virology laboratory, Ministry of Health	Asaf Biber; Danis Sofer; Efrat Bucris; Ella Mendelson; Gili Regev-Yochay; Ilana Tal; Michal Mandelboim; Miki Goldenfeld; Neta Zuckerman; Orna Mor; Sabrina Hason; Sharon Amit; Shiraz Gefen-Halevi
EPI_ISL_596454	Infectious Disease and Tropical Medicine Research Center, Resistant Tuberculosis Institute, Zahedan University of Medical Sciences, Zahedan, Iran.	Genetics Research Center, University of Social Welfare and Rehabilitation Sciences	Ali Jafarpour; Azam Ghaziasadi; Ebrahim Kord; Hossein Najmabadi; Khadijeh Jalalvand; Kimia Kahrizi; Marzieh Mohseni; Seyed Mohammad Hashemi-Shahri; Seyed Mohammad Jazayeri; Seyedeh elham Mortazavi; Zohreh Fattahi
EPI_ISL_884351	Infectious Diseases, Quest Diagnostics	Infectious Diseases, Quest Diagnostics	Anderson, B.; Bernstein; D.F.; Gerasimova, A.; Hua, M.; K.E.; Kagan; L.E.; Lacbawan, F.; Liu, Y.; Livingston; Owen, R.; R.M.; Rosenthal; S.H.; Shalhout
EPI_ISL_2089321, EPI_ISL_2089449, EPI_ISL_2186734, EPI_ISL_2283374, EPI_ISL_3754621	Infinity Biologix	Centers for Disease Control and Prevention Division of Viral Diseases, Pathogen Discovery	Adrian Paskey; Benjamin Rambo-Martin; Chirayu Goswami; Christian Bixby; Christopher Gulvick; Clinton Paden; Clinton R. Paden; Dakota Howard; Darlene Wagner; Dhwani Batra; Duncan MacCannell; Jason Caravas; Jonathan Schultz; Kara Moser; Matthew Schmerer; Peter Cook; Peter W. Cook; Robin Grimwood; Russ Hager; Scott Sammons; Shatavia Morrison; Yihe Wang; Yvette Unoarumhi
EPI_ISL_2281233, EPI_ISL_2281238, EPI_ISL_2281248	Insitut Pasteur Bangui	Institut Pasteur de Dakar	Amadou Alpha Sall; Amadou Diallo; Benjamin Selekon; Cheikh Loucoubar; Christian Malaka; Mamadou Diop; Marie-Astrid Vernet; Moussa Moïse Diagne; Ndack Ndiaye; Ndongo Dia; Ousmane Faye; Rivalyn Nakoune Yandoko; Safietou Sankhe; Sandra Garba Ouangole
EPI_ISL_1913213	Institut National d'Hygiène	Laboratoire de Biotechnologie	Abdelomunim Essabbar; Fatima El Falaki; Hicham Oumzil; Lahcen Belyamani and Azeddine Ibrahimi; Mohamed Rhajaoui; Mouna Ouadghiri; Saaïd Amzazi; Tarik Aanniz
EPI_ISL_1439647	Institut National d'Hygiène	Unité Mixte Internationale TransVIHMI (UMI 233 IRD – U1175 INSERM - Université de Montpellier) IRD (Institut de recherche pour le développement)	Abla A. KONOU; Adodo SADJI; Ahidjo AYOUBA; Akoélé SILIADIN; Alassane OURO-MEDELI; Amivi EHLAN; Améyo DORKENOO; Anoumou DAGNRA; Christelle BUTEL; Déléma MABA; Eric DELAPORTE; Issaka Maman; Kokou TEGUENI; Laetitia SERRANO; Martine PEETERS; Messanh DOUFFAN; Mireille PRINCE-DAVID; Mounerou SALOU; Sidonie A.M.KAGNISSODE; Sika DOSSIM; Wembo A. HALATOKO
EPI_ISL_1415428	Institut National d'hygiène	"Unité Mixte Internationale TransVIHMI (UMI 233 IRD – U1175 INSERM - Université de Montpellier) IRD (Institut de recherche pour le développement)"	Abla A. KONOU; Adodo SADJI; Ahidjo AYOUBA; Akoélé SILIADIN; Alassane OURO-MEDELI; Amivi EHLAN; Améyo DORKENOO; Anoumou DAGNRA; Christelle BUTEL; Déléma MABA; Eric DELAPORTE; Issaka Maman; Kokou TEGUENI; Laetitia SERRANO; Martine PEETERS; Messanh DOUFFAN; Mireille PRINCE-DAVID; Mounerou SALOU; Sidonie A.M.KAGNISSODE; Sika DOSSIM; Wembo A. HALATOKO
EPI_ISL_1508953, EPI_ISL_1508958	Institut National d'hygiène	Unité Mixte Internationale TransVIHMI (UMI 233 IRD – U1175 INSERM - Université de Montpellier) IRD (Institut de recherche pour le développement)	Abla A. KONOU; Adodo SADJI; Ahidjo AYOUBA; Akoélé SILIADIN; Alassane OURO-MEDELI; Amivi EHLAN; Améyo DORKENOO; Anoumou DAGNRA; Christelle BUTEL; Déléma MABA; Eric DELAPORTE; Issaka Maman; Kokou TEGUENI; Koku AGBODEKA; Laetitia SERRANO; Martine PEETERS; Messanh DOUFFAN; Mireille PRINCE-DAVID; Mounerou SALOU; Sidonie A.M.KAGNISSODE; Sika DOSSIM; Syntyche DEVATCHAGNI; Wembo A. HALATOKO
EPI_ISL_418206, EPI_ISL_418208, EPI_ISL_481235	Institut Pasteur Dakar	Institut Pasteur de Dakar	Amadou Alpha Sall; Amadou Alpha Sall.; Mamadou Diop; Mamadou Malado Jallow; Marie Henriette Dior Ndione; Moussa Moise Diagne; Ndongo Dia; Ousmane Faye; Safietou Sanke
EPI_ISL_498238	Institut Pasteur de Dakar	Institut Pasteur de Dakar	Amadou Alpha Sall.; Mamadou Diop; Mamadou Malado Jallow; Marie Henriette Dior Ndione; Moussa Moise Diagne; Ndongo Dia; Ousmane Faye; Safietou Sankhe Mbengue
EPI_ISL_2245857, EPI_ISL_2245876, EPI_ISL_2245882, EPI_ISL_2245977	Institut Pasteur de Guinée	Institut Pateur de Dakar	Dia Ndongo; Diagne Moussa Moïse; Diallo Amadou; Diop Mamadou; Faye Ousmane; Grayo Solene; Loucoubar Cheikh; Maimouna Mbanne; Mbengue Safietou Sankhe; Mohamed Kane; Ndiaye Ndack; Sall Amadou Alpha; Tordo Noel
EPI_ISL_613421, EPI_ISL_613425, EPI_ISL_613446	Institut Pasteur de la Guadeloupe	Institut Pasteur de la Guadeloupe	Angela Brisebarre; Antoine Talarmin; Camille Capel; Etienne Simon-Lorière; Marion Barbet; Maud Vanpeene; Méline Bizard; Stéphanie Guyomard; Sylvie Behillil; Sylvie van der Werf; Sébastien Breurec; Vincent Enouf
EPI_ISL_955166, EPI_ISL_3163947	Institute for Biocides and Medical Ecology	Institute of microbiology and Immunology, Faculty of Medicine, University of Belgrade	Banko A.; Cupic M.; Jankovic, M.; Jovanovic, T.; Knezevic, A.; Lazarevic I.; Milicevic, O.; Miljanovic D.; Sekler, M.; Tesovic, B.; Vidanovic, D.
EPI_ISL_490092, EPI_ISL_718301, EPI_ISL_1696746, EPI_ISL_2091000, EPI_ISL_2684545, EPI_ISL_2685841, EPI_ISL_2815375			
see above	Institute for Medical Research, Infectious Disease Research Centre, National Institutes of Health, Ministry of Health Malaysia	Institute for Medical Research, Infectious Disease Research Centre, National Institutes of Health, Ministry of Health Malaysia	Azizan MA; Kalyanasundram J; Kamel K; Mohd Zawawi Z; Mohd-Zawawi Z; Ramly N; Robert F; Suppiah J; Thayan R
EPI_ISL_477210	Institute for Stem Cell Science and Regenerative Medicine	National Centre for Biological Sciences	Aswin Seshasayee; Awadhesh Pandit; Dasaradhi Palakodeti; Farhan Ali; Mohak Sharda; Shah-e-Jahan Gulzar; Srikar Krishna; Uma Ramakrishnan; Vanessa Molin Paynter; Varadha Sundarmurthy
EPI_ISL_882777, EPI_ISL_1491538	Institute for Urban Disease Control and Prevention	COVID-19 Network Investigations (CONI) Alliance	Amornmas Kongklieng; Anek Mungaomklang; Angkana Huang; Anthony R. Jones; Arporn Wangwiwatsin; Bhakbhoom Panthan; Chonticha Klungtong; Duangkamon Loesbanluechai; Ekawat Pasomsub; Elizabeth Batty; Insee Sensorn; Janjira Thaipadungpanit; Kamolthip Atsawawaranunt; Khajohn Joonlasak; Khajohn Joonsalak; Kingkan Rakmanee; Krittikorn Kumpornsin; Namfon Kotanan; Prayuth Kaewmalang; Pukkaporn Parnwijitkul; Stefan Fernandez; Thanat Chookajorn; Theerarat Kochakarn; Treewat Watthanachockchai; Vichan Pawun; Wasun Chantratita; Wudtichai Manasatienkij
EPI_ISL_1057035, EPI_ISL_1061425	Institute of Biocides and Medical Ecology, Belgarde, Serbia	Virology Department Institute of Microbiology and Immunology Faculty of Medicine University of Belgrade	Abazovic Dzihan; Banko Ana; Despot Dragana; Loncar Ana; Milicevic Ognjen; Miljanovic Danijela
EPI_ISL_2600368, EPI_ISL_3712556, EPI_ISL_3712588	Institute of Epidemiology, Disease Control and Research (IEDCR)	IEDCR-ideSHi-icddr,b	Firdausi Qadri; Hassan Afrad; Manjur Hossain Khan; Omar Hamza; Sadia Rahman; Tahmina Shirin
EPI_ISL_1510994, EPI_ISL_2322232, EPI_ISL_2322329, EPI_ISL_2322334, EPI_ISL_2492244, EPI_ISL_2644249, EPI_ISL_2644270, EPI_ISL_2887277, EPI_ISL_2966940, EPI_ISL_3030114, EPI_ISL_3503944			
see above	Institute of Microbiology and Immunology, Faculty of Medicine, University of Ljubljana	Institute of Microbiology and Immunology, Faculty of Medicine, University of Ljubljana	Alen Suljič; Andraž Celar; Dominika Šturm; Doroteja Vlaj; Mario Poljak; Matic Brvar; Miša Korva; Patricija Pozvek; Samo Zakotnik; Tatjana Avšič – Županc; Tomaž Mark Zorec; Špela Pleh
EPI_ISL_491937, EPI_ISL_527813, EPI_ISL_660532, EPI_ISL_697789, EPI_ISL_877554, EPI_ISL_2100425, EPI_ISL_2100429, EPI_ISL_2100431, EPI_ISL_2228102, EPI_ISL_2361460, EPI_ISL_3089166, EPI_ISL_3506201, EPI_ISL_3506202			
see above	Institute of Microbiology, Universidad San Francisco de Quito	Institute of Microbiology, Universidad San Francisco de Quito	Andrea Pinos; Belén Prado-Vivar; Bernardo Gutiérrez; Christian Zambrano; Edmundo Encalada; Erika B. Muñoz; Erika Muñoz; Fernada Zurita; Fernanda Zurita; Francisco Cordova; Gabriel Trueba; Guzmán Bernabéu Lorenzo; Hugo Hernández; Jonathan Araujo; Juan Carlos Zuñiga; Juan José Guadalupe; Juan Pablo Haro; Juan Pablo Román; Khurram Mahbbob; Kyllen Briones; Luis Morales; Michelle Grunauer; Miriam Ruiz; Monica Becerra-Wong; Nabih Dahik; Ninfa Hernandez; Patricio Rojas-Silva; Paúl Cárdenas; Rosario Erazo; Sully Márquez; Tania Lluma; Verónica Barragán
EPI_ISL_1443652, EPI_ISL_1443654	Institute of Microbiology, Universidad San Francisco de Quito	Omics Sciences Laboratory	; Andrea Cunguan; Belén Prado-Vivar; Bernardo Gutiérrez; Darlyn Amaya; Dayron Brossad; Derly Andrade Molina; Emily Sulay Saltos Montalvo; Fernanda Zurita; Gabriel Morey León; Gabriel Trueba; Juan Carlos Fernández Cadena; Juan José Guadalupe; Katheryn Sacheri Viteri; Michelle Grunauer; Monica Becerra-Wong; Nabih Dahik; Patricio Rojas-Silva; Paula Juliana Gavilanes Jarrín; Paúl Cárdenas; Rubén Armas González; Sully Márquez; Verónica Barragán
EPI_ISL_2709774	Institute of Molecular and Translational Medicine / Laboratory of Experimental Medicine, Faculty of Medicine and Dentistry, Palacky University and University Hospital Olomouc	Institute of Molecular and Translational Medicine / Laboratory of Experimental Medicine, Faculty of Medicine and Dentistry, Palacky University	Barbora Blumová; Hana Dřímalová; Marián Hajdúch; Rastislav Slavkovský; Vladimíra Koudeláková
EPI_ISL_403931	Institute of Pathogen Biology, Chinese Academy of Medical Sciences & Peking Union Medical College	Institute of Pathogen Biology, Chinese Academy of Medical Sciences & Peking Union Medical College	Chao Wu; Jianwei Wang; Lili Ren; Qi Jin; Yiwei Liu; Zhiqiang Wu; Zichun Xiang
EPI_ISL_2986987	Institute of Public Health Kraljevo	Veterinary Specialized Institute Kraljevo	Afonso, C.; Banovic Djeri, B.; Djukic V, .; Jankovic, M.; Jovanovic, T.; Knezevic, A.; Petrovic, T.; Sekler, M.; Tesovic, B.; Vidanovic, D.; Volkening, J.
EPI_ISL_959640, EPI_ISL_1797878, EPI_ISL_1818994, EPI_ISL_1994554, EPI_ISL_2351279, EPI_ISL_2894474	Institute of Virology, Biomedical Research Center of the Slovak Academy of Sciences, Bratislava	Faculty of Natural Sciences, Comenius University, Bratislava	Boris Klempa; Brona Brejova; Broňa Brejová; Jozef Nosek; Juraj Kopacek; Juraj Kopáček; Kristina Borsova; Kristína Boršová; Lubomira Lukacikova; Martina Lickova; Martina Ličková; Martina Nebohacova; Martina Neboháčová; Monika Slavikova; Monika Sláviková; Sabina Fumacova Havlikova; Sabina Fumačová Havlíková; Tomas Vinar; Tomáš Vinař; Viktoria Cabanova; Viktoria Hodorova; Viktória Hodorová; Viktória Čabanová; Ľubomíra Lukáčiková
EPI_ISL_1654837, EPI_ISL_3163954, EPI_ISL_3163956, EPI_ISL_3163960, EPI_ISL_3163961, EPI_ISL_3163962, EPI_ISL_3163965			
see above	Institute of Virology, Vaccines and Sera "Torlak"	Institute of microbiology and Immunology, Faculty of Medicine, University of Belgrade	Banko A.; Cupic M.; Jankovic, M.; Jovanovic, T.; Knezevic, A.; Lazarevic I.; Milicevic, O.; Miljanovic D.; Sekler, M.; Tesovic, B.; Vidanovic, D.
EPI_ISL_1017686	Institute of Virology, Vaccines and Sera ''Torlak''	Institute of microbiology and Immunology, Faculty of Medicine, University of Belgrade	Jankovic, M.; Jovanovic, T.; Knezevic, A.; Milicevic, O.; Sekler, M.; Tesovic, B.; Vidanovic, D.
EPI_ISL_792104	Instituto Adolfo Lutz - Central	Instituto Adolfo Lutz, Interdiciplinary Procedures Center, Strategic Laboratory	Claudia Regina Gonçalves; Claudio Tavares Sacchi; Erica Valessa Ramos Gomes; Karoline Rodrigues Campos

EPI_ISL_2691198	Instituto Adolfo Lutz - Reginal de Sao José do Rio Preto	Instituto Adolfo Lutz, Interdiciplinary Procedures Center, Strategic Laboratory	Caio Vinicius Dias Lopes; Claudia Regina Gonçalves; Claudio Tavares Sacchi; Erica Valessa Ramos Gomes; Karoline Rodrigues Campos; Leonardo Jose Tadeu de Araujo
EPI_ISL_2344240, EPI_ISL_2344450	Instituto Butantan	Instituto de Medicina Tropical de Sao Paulo	Brazil-UK Centre for Arbovirus Discovery Diagnosis Genomics and Epidemiology (CADDE) Genomic Network - Instituto de Medicina Tropical
EPI_ISL_3418681	Instituto Carlos Chagas - ICC, FIOCRUZ Parana	Instituto Carlos Chagas - ICC, FIOCRUZ Parana	A.A.; A.M.; A.R.; Aguiar; Albrecht, L.; Alves; Avila; Balsanelli, E.; Becker, G.; Blanes, L.; Dallagiovanna, B.; Debur; E.M.; F.K.; F.O.; Faoro, H.; Graef, T.; H.G.; I.N.; L.G.; L.R.; M.M.; M.O.; Marchini; Md.C.; Morello; Nardeli; Oliveira; P.C.; Passetti, F.; Pedrosa; Resende; Riediger; S.C.; Schemberger; Suzukawa; V.A.; Zanette, D.; de Baura; de Souza; dos Santos
EPI_ISL_491176, EPI_ISL_491190, EPI_ISL_491226	Instituto Gulbenkian de Ciência	Instituto Gulbenkian de Ciência	Cathy Paulino; Joao Sobral; João Costa; Ricardo Leite; Susana Ladeiro
EPI_ISL_2348761	Instituto Nacional De Investigación En Salud Pública-Crn De Influenza Y Otros Virus Respiratorios	NIC-Instituto Nacional de Investigación en Salud Pública	Alfredo Bruno; Domenica de Mora.; Jimmy Garcés; Johanna Laines; Lizbeth Patiño; Manuel Gonzalez; Maritza Olmedo; Michelle Páez
EPI_ISL_491953	Instituto Nacional de Investigación en Salud Pública - INSPI	INSPI - Charité	Alberto Orlando; Alexandra Usiña; Alfredo Bruno Caicedo; Andres Moreira-Soto; Anna-Lena Sander; Denisses Portugal; Domenica de Mora Coloma; Jan Felix Drexler; Juan Carlos Zeballos; Manuel Gonzalez; Maritza Olmedo; Nina Krause; Silvia Salgado
EPI_ISL_2492714, EPI_ISL_2492791, EPI_ISL_3730383, EPI_ISL_3730384, EPI_ISL_3730398, EPI_ISL_3730401, EPI_ISL_3730414, EPI_ISL_3730416, EPI_ISL_3730437, EPI_ISL_3730438, EPI_ISL_3730458, EPI_ISL_3859858, EPI_ISL_3859860, EPI_ISL_3859907, EPI_ISL_3860051, EPI_ISL_3860074, EPI_ISL_3860117			
see above	Instituto Nacional de Investigação em Saúde	CERI, Centre for Epidemic Response and Innvoation, Stellenbosch University and KRISP, KZN Research Innovation and Sequencing Platform, UKZN.	Afonso P; David K; Emmanuel SJ; Freitas RH; Giandhari J; Inglês L; Lutucuta S; Miranda J; Morais J; Mufinda M; Naidoo Y; Neto Z; Paulo A Carralero RR Paixão JP; Pereira A; Pillay S; Tegally H; Wilkinson E; de Oliveira T
EPI_ISL_1347945	Instituto Nacional de Investigação em Saúde	KRISP, KZN Research Innovation and Sequencing Platform	Afonso P; David K; Emmanuel SJ; Freitas RH; Giandhari J; Inglês L; Lutucuta S; Miranda J; Morais J; Mufinda M; Naidoo Y; Neto Z; Paulo A Carralero RR Paixão JP; Pereira A; Pillay S; Tegally H; Wilkinson E; de Oliveira T
EPI_ISL_1055023, EPI_ISL_2894237, EPI_ISL_3160783, EPI_ISL_3548071	Instituto Nacional de Medicina Genomica	Instituto Nacional de Medicina Genomica	Alcaraz N; Cedro-Tanda A; Cisneros- Villanueva M; Cisneros-Villanueva M; Escobar-Arrazola; Escobar-Arrazola MA; Gonzalez-Barrera D; Herrera-Montalvo LA; Herrera-Montalvo LA.; Hidalgo-Miranda A; M; Mendoza-Vargas A; Munguia-Garza P; Ramirez-Vega O; Rangel-DeLeon D; Reyes-Grajeda JP; Sifuentes-Rojas C
EPI_ISL_2500963, EPI_ISL_2500964, EPI_ISL_2500965, EPI_ISL_2827762	Instituto Nacional de Salud	Instituto Nacional de Salud- Dirección de Investigación en Salud Pública	Carlos Franco-Muñoz; Carmen Osorio; Diana Malo; Diego A. Álvarez-Díaz; Diego Andrés Prada; Gerardo Santamaría; Hector Alejandro Ruiz-Moreno; Jhonnatan Reales-González; Jorge Rivera; Juan Camilo Martinez; Julian Naizaque; Katherine Laiton-Donato; Lisseth Pardo; Magdalena Wiesner; Marcela Mercado-Reyes; Maria T. Herrera-Sepúlveda; Marta Lopez Blanco; Martha Lucia Ospina Martinez; Paola Rojas; Sergio Gomez; Sheryll Corchuelo; Ángela Alarcon Cruz
EPI_ISL_456130	Instituto Nacional de Salud - Unidad de Secuenciación y Análisis Genómico	Instituto Nacional de Salud, Universidad Cooperativa de Colombia, Instituto Alexander von Humboldt, Imperial College-London, London School of Hygiene & Tropical Medicine	Astrid C. Flórez; Carlos Franco-Muñoz; Christian Julian Villabona-Arenas; Diana Marcela Walteros-Acero; Diego A. Álvarez-Díaz; Erika Ospitia; Gloria Puerto; Jose A. Usme-Ciro; Juliana Barbosa; Katherine Laiton-Donato; Liz Villabona-Arenas; Luz Dary Rodriguez; Mailyn A.Gonzalez; Marcela Mercado-Reyes.; Martha Lucia Ospina Martinez; Nicolas D. Franco-Sierra; Sergio Gomez-Rangel; Sussy Echeverria; Zulma M. Cucunubá
EPI_ISL_526969, EPI_ISL_739673	Instituto Nacional de Salud, Bogotá, Colombia	Instituto Nacional de Salud, Bogotá, Colombia	Astrid C. Flórez; Carlos Andrés Durán; Carlos Franco-Muñoz; Carolina Ferro; Christian Julian Villabona-Arenas; Diana Marcela Walteros-Acero; Diego A. Álvarez-Díaz; Diego Andrés Prada; Franklin Prieto; Jonathan Reales; Jose A. Usme-Ciro; Katherine Laiton-Donato; Liz Villabona-Arenas; Magdalena Weisner; Marcela Mercado-Reyes; Martha Lucia Ospina Martinez; Mauricio Pacheco-Montealegre; Sheryl Corchuelo; Sussy Echeverria; Zulma M. Cucunubá
EPI_ISL_3663545, EPI_ISL_3663546, EPI_ISL_3663547, EPI_ISL_3663548, EPI_ISL_3663550, EPI_ISL_3663552, EPI_ISL_3663554, EPI_ISL_3663569, EPI_ISL_3663582, EPI_ISL_3663600			
see above	Instituto Nacional de Saude (INS), Mozambique	CERI, Centre for Epidemic Response and Innvoation, Stellenbosch University and KRISP, KZN Research Innovation and Sequencing Platform, UKZN.	Emmanuel S; Giandhari J; Nadia Sitoe; Naidoo Yeshnee; Nalia Ismael; Nedio Mabunda; Paulo Arnaldo; Pillay S; Tegally H; Tshabuila Derek; Wilkinson E; Yajna Ramphal; de Oliveira T
EPI_ISL_2396951	Instituto Nacional de Saude (INS), Mozambique	KRISP, KZN Research Innovation and Sequencing Platform	Emmanuel S; Giandhari J; Nadia Sitoe; Nalia Ismael; Nedio Mabunda; Paulo Arnaldo; Pillay S; Tegally H; Wilkinson E; de Oliveira T
EPI_ISL_3507367, EPI_ISL_3507369, EPI_ISL_3507373, EPI_ISL_3507374, EPI_ISL_3507375	Instituto Venezolano de Investigaciones Científicas	Laboratorio de Virología Molecular	Carmen L Loureiro; CoViVen Group; Domingo J Garzaro; Flor H Pujol; Héctor R Rangel; José Luis Zambrano; Lieska Rodríguez; Mariana Hidalgo; Pierina D´Angelo; Rossana C Jaspe; Víctor Alarcón; Yoneira Sulbaran; Zoila Moros
EPI_ISL_455432, EPI_ISL_455434, EPI_ISL_493338, EPI_ISL_493342, EPI_ISL_576278	Instituto de Diagnostico y Referencia Epidemiologicos (INDRE)	Instituto de Diagnostico y Referencia Epidemiologicos (INDRE)	Abril Rodriguez-Maldonado; Adnan Araiza-Rodriguez; Araiza-Rodriguez Adnan; Barrera-Badillo Gisela; Claudia Wong-Arambula; Dayanira Arellano-Suarez; Edgar Mendieta-Condado; Ernesto Ramirez-Gonzalez; Ernesto Ramirez-Gonzalez.; Fabiola Garces-Ayala; Garces-Ayala Fabiola; Gisela Barrera-Badillo; Gisela Barrera-Badillo.; Hernandez-Rivas Lucia; Irma Lopez-Martinez; Lopez-Martinez Irma.; Lucia Hernandez-Rivas; Mendieta-Condado Edgar; Natividad Cruz-Ortiz; Rodriguez-Maldonado Abril; Taboada Ramírez Blanca. Ramirez-Gonzalez Ernesto; Tatiana Nunez-Garcia; Wong-Arambula Claudia
EPI_ISL_1060761	Instituto de Diagnostico y Referencia Epidemiologicos (INDRE)_RNLSP	Instituto de Diagnostico y Referencia Epidemiologicos (INDRE)	Abril Rodriguez-Maldonado; Adnan Araiza-Rodriguez; Claudia Wong-Arambula; David Fragoso-Fonseca; Ernesto Ramirez-Gonzalez.; Fabiola Garces-Ayala; Gisela Barrera-Badillo; Irma Lopez-Martinez; Lucia Hernandez-Rivas; Mayra Jimenez-Morales; Nancy Munoz-Hernandez; Natividad Cruz-Ortiz; Sergio Rangel-Guerrero; Tatiana Nunez-Garcia
EPI_ISL_913929, EPI_ISL_913936, EPI_ISL_913971, EPI_ISL_1054949, EPI_ISL_1054969, EPI_ISL_1054986, EPI_ISL_1168551			
see above	Instituto de Diagnostico y Referencia Epidemiologicos INDRE_RNLSP	Instituto de Diagnostico y Referencia Epidemiologicos (INDRE)	Abril Rodriguez-Maldonado; Adnan Araiza-Rodriguez; Ariadna Medina-Benitez; Claudia Wong-Arambula; David Fragoso-Fonseca; Ernesto Ramirez-Gonzalez.; Fabiola Garces-Ayala; Gisela Barrera-Badillo; Irma Lopez-Martinez; Joaquin Quiroz-Mercado; Lucia Hernandez-Rivas; Mayra Jimenez-Morales; Nancy Munoz-Hernandez; Natividad Cruz-Ortiz; Sergio Rangel-Guerrero; Tatiana Nunez-Garcia; Vanessa Rivero-Arredondo
EPI_ISL_1301592, EPI_ISL_1301702, EPI_ISL_3463581, EPI_ISL_3463595	Instituto de Diagnostico y Referencia Epidemiologicos InDRE_RNLSP	Instituto de Biotecnología de la UNAM	Alejandra Hernández-Terán; Alejandro Sanchez-Flores; Alma Rincón-Rubio; Andrea Santos Coy-Arechavaleta; Authors from IBT; Blanca Taboada; Celia Boukadida; Clara Esperanza Santacruz-Tinoco; Edgar Mendieta-Condado; Eduardo Becerril-Vargas; Fidencio Mejía-Nepomuceno; Francisco Pulido; Gisela Barrera-Badillo; Gloria Vazquez; Hector Esteban Paz-Juárez; IMSS; InDRE and INER (in alphabetical order): Carlos F. Arias; Irma Lopez-Martinez; Jerome Jean Verleyen; Joel Armando Vázquez-Pérez; Jorge Salas-Hernández; José Arturo Martínez-Orozco; José Ernesto Ramírez-González; José Esteban Muñoz-Medina; Larissa Fernandes-Matano; Lucia Hernandez-Rivas; Luis Alberto Ochoa-Carrera; Margarita Matías-Florentino; Mario Mújica-Sánchez; Natividad Cruz-Ortiz; Pavel Isa; Ricardo Grande; Santiago Ávila-Ríos; Tatiana Nunez-Garcia; Teresita Rojas-Mendoza
EPI_ISL_1395782	Instituto de Investigaciones Biomédicas en Retrovirus y SIDA (INBIRS)	Área de Secuenciación del Laboratorio de Virología del Hospital de Niños Dr. Ricardo Gutierrez on behalf of 'Proyecto Argentino Interinstitucional de genomica de SARS-CoV-2' (PAIS Consortium)	Acuña; Alexay; D; Federico Remes Lenicov; Goya; Horacio Salomón; LE; Lusso; M; MI; Nabaes Jodar; Natale; S; Valinotto; Vanesa Seery; Viegas, M.
EPI_ISL_1378844, EPI_ISL_3236440, EPI_ISL_3236451	Instituto de Medicina Tropical & Salud Global Universidad Iberoamericana	Grubaugh Lab - Yale School of Public Health	Alejandaro Vallejo Degaudenzi; Anderson Brito; Annie Watkins; Chaney Kalinich; Chantal Vogels; Elisa Contreras; Esperanza Mendoza; Isabel Ott; Isabell Ott; Jessica Rothman; Joseph Fauver; Kendall Billig; Mallery Breban; Mary Petrone; Nathan Grubaugh; Robert Paulino-Ramirez; Tara Alpert; Tobias Koch; Victor Virgilio Calderon
EPI_ISL_2601035	Instituto de Medicina Tropical & Salud Global, Universidad Iberoamericana (UNIBE)	Instituto de Medicina Tropical & Salud Global, Universidad Iberoamericana (UNIBE)	Alejandro Vallejo Degaudenzi & Robert Paulino-Ramirez; Victor Virgilio Calderón
EPI_ISL_3020261	Instituto de Medicina Tropical Alexander Von Humboldt, Sede Cusco	Laboratorio de Genómica Microbiana, Universidad Peruana Cayetano Heredia	Alejandra Dávila-Barclay; Diego Cuicapuza; Guillermo Salvatierra; Janet Huancachoque; Luis González; Pablo Tsukayama; Pedro E. Romero; Pool Marcos
EPI_ISL_1697390	Iressef / LBV	Iressef Genomics lab	Abdou PADANE; Adjiratou Aissatou BA; Alioune TINE; Ambroise AHOUIDI; Aminata DIA; Aminata MBOUP; Anna Julienne Selbe NDIAYE; Assane DIENG; Astou Gaye GAYE; Barada CISSE; Birahim Piere NDIAYE; Gora LO; Khadim GUEYE; Makhtar Camara; Mengue FALL; Moustapha MBOW; Nafisatou LEYE; Ndeye Coumba Toure KANE; OUMY DIOP; Papa Alassane DIAW; Pauline SENE; SADA DIALLO; Serigne Mbaye Lo NDIAYE; Souleymane MBOUP; Yacine DIA
EPI_ISL_2620888, EPI_ISL_2620889, EPI_ISL_2873833, EPI_ISL_2887851, EPI_ISL_2887852, EPI_ISL_3154879, EPI_ISL_3154891, EPI_ISL_3154898			
see above	Iressef Genomics lab	IRESSEF	Abdou PADANE; Ambroise AHOUIDI; Aminata DIA; Aminata MBOUP; Astou Gaye GAYE; Barada CISSE; Birahim Piere NDIAYE; Diabou Diagne; Gora LO; Khadim GUEYE; Moustapha MBOW; Nafisatou LEYE; Ndeye Coumba Toure KANE; Papa Alassane DIAW; Samba Ndiour; Seni Ndiaye; Souleymane MBOUP; Yacine DIA
EPI_ISL_1167164	Iressef Genomics lab	L'institut de Recherche en Santé, de Surveillance Épidémiologique et de Formation (IRESSEF)	Abdou PADANE; Abdoulie KANTEH; Abdul Karim SESAY; Ambroise AHOUIDI; Aminata DIA; Aminata MBOUP; Astou Gaye GAYE; Barada CISSE; Birahim Piere NDIAYE; Gora LO; Khadim GUEYE; Moustapha MBOW; Nafisatou LEYE; Ndeye Coumba Toure KANE; Papa Alassane DIAW; Souleymane MBOUP; Yacine DIA
EPI_ISL_2363855	Islab, Pohjois-Savon aluelaboratorio	Expert Microbiology, National Institute for Health and Welfare	Carita Savolainen-Kopra; Erika Lindh; Haider al-Hello; Jani Halkilahti; Kirsi Liitsola; Niina Ikonen; Pekka Ellonen; Päivi Laurila; Sari Hannula; Soile Blomqvist; Teemu Smura
EPI_ISL_1240647	Israel Central Virology Laboratory	Israel National Consortium for SARS-CoV-2 sequencing	Assaf Rokney; Dana Bar-Ilan; David A. Zeevi; Efrat Dahan Bucris; Efrat Glick-Saar; Efrat Rorman; Ella Mendelson; Ephraim Fass; Eva Nachum; Gal Zizelski Valenci; Gideon Rechavi; Israel Nissan; Joseph Jaffe; Maya Davidovich Cohen; Michal Mandelboim; Mor Rubinstein; Neta Zuckerman; Omer Murik; Omri Nayshool; Oran Erster; Orna Mor; Tzvia Mann
EPI_ISL_514301, EPI_ISL_516885, EPI_ISL_516903, EPI_ISL_575332, EPI_ISL_649094	Israel Central Virology laboratory	Israel Central Virology laboratory	Efrat Dahan Bucris; Ella Mendelson; Michal Mandelboim; Neta Zuckerman; Oran Erster
EPI_ISL_2085212, EPI_ISL_2636201, EPI_ISL_2636253, EPI_ISL_3278414, EPI_ISL_3278749	Israel Central Virology laboratory	Israel National Consortium for SARS-CoV-2 sequencing	Dana Bar-Ilan; Danit Sofer; Efrat Dahan Bucris; Efrat Glick-Saar; Ella Mendelson; Gideon Rechavi; Michal Mandelboim; Miranda Geva; Neta Zuckerman; Netanel Abu; Omer Asraf; Omri Nayshool; Oran Erster; Orna Mor
EPI_ISL_778745, EPI_ISL_1424301, EPI_ISL_2226866, EPI_ISL_2356926,	Istituto Zooprofilattico Sperimentale del Mezzogiorno	TIGEM	Andrea Ballabio; Anna Manfredi; Antonio Grimaldi; Antonio Grimaldi Patrizia Annunziata Francesco Panariello Biancamaria Pierri Claudia Tiberio Teresa Giuliano Valentina Bouche Chiara Colantuono Maria Concetta Cuomo Denise Di Concilio Lucio Di Filippo Anna Manfredi Marcello Salvi Antonio Limone Luigi Atripaldi Pellegrino Cerino Andrea Ballabio Davide Cacchiarelli; Antonio Limone; Antonio Limone Luigi Atripaldi Pellegrino Cerino; Biancamaria Pierri; Biancamaria Pierri Claudia Tiberio Valentina Bouche; Chiara Colantuono; Davide Cacchiarelli; Davide Cacchiarelli.; Denise Di Concilio; Francesco Panariello; Lucio Di Filippo; Marcello Salvi; Maria Concetta Cuomo; Patrizia Annunziata; Pellegrino Cerino; Valentina Bouche

EPI_ISL_2550987			
EPI_ISL_1254720	Istituto Zooprofilattico Sperimentale del Mezzogiorno	Telethon Institute of Genetics and Medicine (TIGEM)	Antonio Grimaldi Patrizia Annunziata Francesco Panariello Biancamaria Pierri Claudia Tiberio Valentina Bouche Chiara Colantuono Maria Concetta Cuomo Denise Di Concilio Lucio Di Filippo Anna Manfredi Marcello Salvi Antonio Limone Luigi Atripaldi Pellegrino Cerino Andrea Ballabio Davide Cacchiarelli
EPI_ISL_3769356, EPI_ISL_3769372	JKN PAHANG	iPROMISE, UiTM	Ariza Adnan; Fadzilah Mohd Nor; Lim Wai Feng; Mohd Asif Mohd Sukri; Mohd Nur Fakhruzzaman Noorizhab; Mohd Zaki Salleh; Sazzli Shahlan Kassim; Siti Farah Alwani Mohd Nawi; Siti Hamimah Sheikh Abdul Kadir; Teh Lay Kek; Wang Seok Mui
EPI_ISL_2958697	Jaber Al Ahmad Hospital	Virology Lab, Jaber Al Ahmad Hospital	Dr. Ebaa Al-Awadhi; Dr. Zahrah Buhamad; Haroon Masih; Khubaid-ur-Rehman
EPI_ISL_3087589	Jaber Quarantine Station	Virology Lab, Jaber Al Ahmad Hospital	Adil Afridi; Dr. Ebaa Al-Awadhi; Dr. Zahrah Buhamad; Haroon Masih; Khubaid-ur-Rehman
EPI_ISL_2984234	Jahra Hospital	Kuwait Cancer Control Center	Mona Alateeqi; Shakir Bahzad
EPI_ISL_779257, EPI_ISL_779272, EPI_ISL_779288, EPI_ISL_779290, EPI_ISL_2151364, EPI_ISL_3052635	Jamil-ur-Rahman Center for Genome Research, Dr. Panjwani Center for Molecular Medicine and Drug Research	Jamil-ur-Rahman Center for Genome Research, Dr. Panjwani Center for Molecular Medicine and Drug Research	A.H.; Ain, N.; Ansari, S.; Hashmi; I.A.; Imran, S.; Irfan, M.; Khan; Khan, I.; Khan, S.; M.K.; Nisa; Nisa, Z.; Parveen, N.; Rashid, M.; Shafi; Shakeel, M.; Siddiqui, S.; Z.U.; Zehra, M.
EPI_ISL_416626	Japanese Quarantine Stations	Pathogen Genomics Center, National Institute of Infectious Diseases	Hajime Kamiya; Hideki Hasegawa; Ikuyo Takayama; Kentaro Itokawa; Makoto Kuroda; Masanori Hashino; Motoi Suzuki; Rina Tanaka; Shinji Saito; Takaji Wakita; Takuri Takahashi; Takuya Yamagishi; Tsutomu Kageyama; Tsuyoshi Sekizuka
EPI_ISL_3105867	Jecsons Medical Center	Research Institute for Tropical Medicine	Clyde Dapat; Deana Mae Ocampo; Emmanuel Kagning Tsinda; Francisco Gerardo Polotan; Hitoshi Oshitani; Inez Andrea Medado; Jefferson Earl Halog; Joana Ina Manalo; Lei Lanna Dancel; Ma Angelica Tujan; Mariko Saito-Obata; Mayuko Saito; Michiko Okamoto; Samantha Louise Bado
EPI_ISL_2140207	Jessa	Jessa	Berden et al. on behalf of the Jessa_cmdLab
EPI_ISL_2932611, EPI_ISL_2932618	Jordan University of Science Technology/ Irbid filed Hospital/ MOH	Princess Haya Biotechnology Center/ Jordan University of Science Technology	Areej Alquran; Emad Abu Yaqeen; Hazem Haddad; Maha Karam; Saied Jaradat; Shereen Issa; Suha Hasan
EPI_ISL_3275172	K. Maafushi	Indira Gandhi Memorial Hospital	Dr. Milza Abdul Muhsin; Mr. Ibrahim Nishan Ahmed; Ms. Aishath Shuhudha; Ms. Aminath Nazfa; Ms. Fathimath Zimna
EPI_ISL_2602527, EPI_ISL_2602631, EPI_ISL_2602659, EPI_ISL_2602663, EPI_ISL_2602665, EPI_ISL_2602710, EPI_ISL_2602724, EPI_ISL_2602763, EPI_ISL_2603095, EPI_ISL_2603104, EPI_ISL_2603108, EPI_ISL_3049430, EPI_ISL_3049483, EPI_ISL_3049509, EPI_ISL_3049510, EPI_ISL_3049570, EPI_ISL_3049694, EPI_ISL_3049762, EPI_ISL_3049775, EPI_ISL_3049815, EPI_ISL_3049838			
see above	KEMRI-Wellcome Trust Research Programme,Kilifi	KEMRI-Wellcome Trust Research Programme,Kilifi	; Githinji G.; Matoke D.; Mburu M.W.; Mohamed K.S.; Onyango C.; Thiongo K.; de Laurent Z.
EPI_ISL_568703, EPI_ISL_968883, EPI_ISL_1039229	KEMRI-Wellcome Trust Research Programme/KEMRI-CGMR-C Kilifi	KEMRI-Wellcome Trust Research Programme/KEMRI-CGMR-C Kilifi	Githinji et al; Githinji et al 2020
EPI_ISL_3643972	KIMBERLEY LABORATORY	National Institute for Communicable Diseases of the National Health Laboratory Service	Amoako DG; Bhiman JN; Everatt J; Ismail A; Mahlangu B; Mnguni A; Mohale T; Ntuli N; Scheepers C
EPI_ISL_1048849, EPI_ISL_1049006	KU Leuven, Rega Institute, Clinical and Epidemiological Virology	KU Leuven, Rega Institute, Clinical and Epidemiological Virology	Bert Vanmechelen; Joan Marti-Carerras; Piet Maes; Tony Wawina-Bokalanga
EPI_ISL_3430318	Kaduna	National Reference Laboratory, Nigeria Centre for Disease Control	Anthony Ahumibe; Catherine Okoi; Celestina Obiekea; Chimaobi Chukwu; Dr Chikwe Ihekweazu; Dr Ndodo Nnaemeka; Dr Omoare Adesuyi; Nwando Mba; Olusola Anuoluwapo Akanbi
EPI_ISL_495443	Kafkas University, Faculty of Medicine, Department of Medical Microbiology	Kafkas University, Faculty of Medicine, Department of Medical Microbiology	Didem Ozgur; E. Ediz Tutuncu; Murat Karamese
EPI_ISL_3806899	Kansas Health and Environmental Lab	Kansas Health and Environmental Lab	Amanda Bradley; Carrie Welch; Gary Buruss; Jonathan Barnell; Meg Wise; Mike Grose; and Phil Adam
EPI_ISL_2233101	Kantor Kesehatan Pelabuhan Kelas II Cilacap	National Institute of Health Research and Development	Arie Ardiansyah Nugraha; Hana Apsari Pawestri; Hartanti Dian Ikawati; Kartika Dewi Puspa; Krisna Pangesti; Nelly Puspandari; Subangkit; Triyani Soekarso; Vivi Setiawaty
EPI_ISL_2898899, EPI_ISL_3461891	Karolinska University Hospital Solna	Karolinska University Hospital	Annelie Bjerkner; Isak Sylvin; Jan Albert; Karolina Ininbergs; Lina Guerra Blomqvist; Lynda Eneh; Martin Ekman; Martina Wahlund; Robert Dyrdak; Sandra Broddesson; Tanja Normark; Tobias Allander; Valtteri Wirta; Zhibing Yun
EPI_ISL_2820297, EPI_ISL_2820304	Kasane Primary Hospital Laboratory	Botswana Harvard HIV Reference Laboratory	Agnes Karutwaeng; Boitumelo Zuze; Botshelo Radibe; Dorcas Maruapula; Joseph Makhema; Keoratile Ntshambiwa; Legodile Kooepile; Madisa Mine; Mosepele Mosepele; Ontlametse T. Bareng; Roger Shapiro; Rose Munyere; Shahin Lockman; Sikhulile Moyo; Simani Gaseitsiwe; Thongbotho Mphoyakgosi; Wonderful T. Choga
EPI_ISL_754063, EPI_ISL_754064, EPI_ISL_754065	Kathmandu University	Nepal Health Research Council	Meghnath Dhimal; Pradip Gyanwali
EPI_ISL_512812, EPI_ISL_512817	Kenema Government Hospital, Ministry of Health and Sanitation	Kenema Government Hospital, Ministry of Health and Sanitation	Andersen, K.; Garry, R.; Goba, A.; Grant, D.; Happi, C.; Jalloh, S.; Mehta, S.; Momoh, M.; Olawoye, I.; Oluniyi, P.; Park, D.; Sandi, J.; Siddle, K.; Tomkins-Tinch, C.
EPI_ISL_966331	Kentucky State Public Health Lab	Kentucky State Public Health Lab	Joshua Tobias; Karim George; Matthew Johnson; Rachel Zinner; Rhonda Lucas; Stephanie Lunn; Vaneet Arora; William Grooms
EPI_ISL_489997, EPI_ISL_490004	King Fahad Medical City	King Fahad Medical City	Alghoraibi, M.; Alosaimi, B.; Enani, M.; Naeem, A.
EPI_ISL_3237048	King Faisal Specialist Hospital & Research Centre	King Faisal Specialist Hospital & Research Centre	Abeer N Alshukairi; Ashraf Dada; Mohamad K Al Hroub; Rayan Bawayan; Waseem A Al Mousa
EPI_ISL_483547	Kingdom of Bahrein Ministry of Health	Erasmus Medical Center	Amjad Ghanem Mohamed; Anne van der Linden; Bas Oude Munnink; Claudia Schapendonk; David Nieuwenhuijse; Ebrahim Shehad; Fatema; Hashmeya Al Wasti; Irina Chestakova; Marion Koopmans; Mark Pronk; Pascal Lexmond; Reina Sikkema; Richard Molenkamp; Stefan van Nieuwkoop; Theo Bestebroer; on behalf of the Dutch national COVID-19 response team.
EPI_ISL_2674157, EPI_ISL_2693564, EPI_ISL_3386593, EPI_ISL_3652165	Klinika za infektivne bolesti "Dr. Fran Mihaljević"	Hrvatski zavod za javno zdravstvo	Irena Tabain; Ivana Ferenčak
EPI_ISL_2400376	Klinisch Laboratorium ZNA	Klinisch Laboratorium ZNA	Verstrepen et al.
EPI_ISL_1008418	Klinisk mikrobiologi	The Public Health Agency of Sweden	Anna Risberg; Anna-Malin Linde; Carlo Berg; Karin Tegmark-Wisell; Maria Lind Karlberg; Mattias Haukland; Mia Brytting; Noura Walai; Oskar Karlsson Lindsjo; Petra Edquist; Petra Holmstrom; Reza Advani; Sofia Stamouli
EPI_ISL_3333236, EPI_ISL_3333581	Kota Bharu Public Health Laboratory	Institute for Medical Research, Infectious Disease Research Centre, National Institutes of Health, Ministry of Health Malaysia	Anasir MI; Azizan MA; Kamel K; Mohd Zawawi Z; Ramly N; Robert F; Suppiah J; Thayan R
EPI_ISL_515182	Kumasi Centre for Collaborative Research in Tropical Medicine, Kumasi.	Institute of Virology, Charité – Universitätsmedizin Berlin	Augustina Sylverken; Christian Drosten; Eric Adu; Jesse Addo Asamoah; Julia Schneider; Jörn Beheim-Schwarzbach; Michael Owusu; Philip El-Duah; Richard Phillips.; Richmond Gorman; Richmond Yeboah; Sherihane Aryeetey; Victor Max Corman
EPI_ISL_2289468	LABORATOIRE DE BIOLOGIE MEDICALE	CNR Virus des Infections Respiratoires - France SUD	Antonin Bal; Bruno Lina; Gregory Destras; Gwendolyne Burfin; Hadrien Regue; Laurence Josset; Martine Valette; Quentin Semanas
EPI_ISL_3206996	LABORATOIRE d'ANALYSES de BIOLOGIE MEDICALES	CNR Virus des Infections Respiratoires - France SUD	Antonin Bal; Bruno Lina; Gregory Destras; Gwendolyne Burfin; Hadrien Regue; Laurence Josset; Martine Valette; Quentin Semanas
EPI_ISL_918534	LACEN - Laboratório Central de Saúde Pública do Amazonas	Evandro Chagas Institute	A.M.; Barbagelata; E.C.; E.M.A.; Ferreira; J.A.; Junior; K.C.; L.C.; L.S.; M.C.; P.S.; Pinheiro; Santos; Silva; Sousa; Sousa Junior; W.D.C.; da Silva
EPI_ISL_717899	LACEN RJ - Noel Nutels	Bioinformatics Laboratory / LNCC	Alexandra L Gerber; Amilcar Tanuri; Ana Paula de C Guimarães; Ana Tereza R de Vasconcelos; Andréa Cony Cavalcanti; Carolina M Voloch; Claudia dos Santos Rodrigues; Cynthia C Cardoso; Diana Mariani; Luiz G P de Almeida; Otavio Bustrolini; Ronaldo da Silva F Jr; Terezinha M P P Castiñeira
EPI_ISL_3134695, EPI_ISL_3134856, EPI_ISL_3705132	LACEN/PE	WallauLab on behalf of Fiocruz COVID-19 Genomic Surveillance Network	Alexandre Freitas da Silva; Cassia Docena; Constância Flávia Junqueira Ayres; Filipe Zimmer Dezordi; Gabriel Luz Wallau; Gustavo Barbosa de Lima; Lais Ceschini Machado; Lilian Caroliny Amorim Silva; Marcelo Henrique dos Santos Paiva; Matheus Filgueira Bezerra; Sinval Pinto Brandão Filho
EPI_ISL_3149320	LANIIA	LANIIA-Nayarit	; Alejandra García-Gasca; Alejandra Hernández-Terán; Alejandro Sánchez-Flores; Alfredo Herrera-Estrella; Alicia Ocaña-Mondragón; Andreu Comas-García; Angel Gustavo Salas-Lais; Antonio Loza Román; Bernardo Martínez-Miguel; Blanca Taboada; Brenda Irasema Maldonado-Meza; Bruno Gómez-Gil; Carla Ivón Herrera-Najera; Carlos Eduardo Covantes-Rosales; Carlos F. Arias; Celia Boukadida; Clara Esperanza Santacruz-Tinoco; Concepción Grajales-Muñiz; Consorcio Mexicano de Vigilancia Genómica (CoViGen-Mex). Authors (in alphabetical order): Julio Elias Alvarado-Yaah; Cristóbal Cháidez-Quiróz; Célida Duque Molina; Célida Martínez- Rodríguez; Daniel Alberto Girón Pérez; Daniel Fregoso-Rueda; Daniel Lira Morales; Eduardo Becerril-Vargas; Fernando Fontove-Herrera; Fidencio Mejía-Nepomuceno; Francisco Pulido; Gloria Elena Espinosa-Ayala; Gloria María Molina-Salinas; Gloria Vazquez; Hector Esteban Paz-Juárez; Hector Montoya-Fuentes; Helen Haydee Fernanda Ramírez-Plascencia; Irvin González-López; Jean Pierre González; Jesús Hernández; Joel Armando Vázquez-Pérez. LANNIA Nayarit: Manuel Ivan Girón Pérez; Jorge Salas-Hernández; José Antonio Enciso-Moreno; José Arturo Martínez-Orozco; José Esteban Muñoz-Medina; José de Jesús Nuñez-Contreras; Juan Bautista Chale-Dzul; Julissa Enciso-Ibarra; Luis Alberto Ochoa-Carrera; Margarita Matías-Florentino; Mario Mújica-Sánchez; Marissa Perez-Garcia; María Guadalupe Santiago-Mauricio; María Guadalupe de Jesús Mireles-Rivera; Nelly Sélem-Mojica; Pavel Isa; Ricardo Ciria Merce; Ricardo Grande; Rosa María Gutiérrez Rios; Santiago Ávila-Ríos; Selene Zárate; Susana Lopez; Ulises Mercado.; Verónica Mata-Haro; Victor Eduardo García-Arias; Víctor Hugo Borja-Aburto
EPI_ISL_861870, EPI_ISL_3010881, EPI_ISL_3010906, EPI_ISL_3267996	LATE - Laboratório de Técnicas Especiais - Hospital Israelita Albert Einstein	LATE - Laboratório de Técnicas Especiais - Hospital Israelita Albert Einstein	Alexandre Hideaki Takara; Ana Paula Moreira Salles; Anelisie da Silva Santos; Deyvid Amgarten; Erick Gustavo Dorlass; Fernanda de Mello Malta; João Renato Rebello Pinho; Marcio Anunciacao Menezes; Pedro Henrique Sebe Rodrigues; Raquel Riyuzo
EPI_ISL_3132167, EPI_ISL_3132169, EPI_ISL_3132170, EPI_ISL_3132171	LCSP	IICS-UNA	Adriana Valenzuela; Alejandra Rojas; Andrea Gomez de la Fuente; Andrea Ojeda; Chyntia Diaz; Cynthia Vazquez; Eva Nara; Fatima Cardozo; Florencia del Puerto; Guillermo Sequera.; Joel Ortiz; Jonas Fernandez; Juan Torales; Laura Franco; Leticia Rojas; Magaly Martinez; Maria Eugenia Galeano; Maria Liz Gamarra; Shirley Villalba
EPI_ISL_1091785, EPI_ISL_1091786	LDSP	Universidad Nacional de Colombia - Laboratorio Genómico One Health	Andres F. Cardona-Rios; Carlos Franco-Muñoz; Daniel O. Maldonado-Perez; Diego A. Álvarez-Díaz; Idabely Betancur Ortiz; Jorge E. Osorio; Juan P. Hernandez-Ortiz; Karl A Ciuoderis; Laura Silvana Perez; Marcela Mercado-Reyes; Maria Angélica Maya; Sandra Ines Cano

EPI_ISL_2827783	LDSP - CAQUETA HOSPITAL MALVINAS	Instituto Nacional de Salud- Dirección de Investigación en Salud Pública	Carlos Franco-Muñoz; Carmen Osorio; Diana Malo; Diego A. Álvarez-Díaz; Diego Andrés Prada; Gerardo Santamaría; Hector Alejandro Ruiz-Moreno; Jhonnatan Reales-González; Jorge Rivera; Juan Camilo Martinez; Julian Naizaque; Katherine Laiton-Donato; Lisseth Pardo; Magdalena Wiesner; Marcela Mercado-Reyes; Maria T. Herrera-Sepúlveda; Marta Lopez Blanco; Martha Lucia Ospina Martinez; Paola Rojas; Sergio Gomez; Sheryll Corchuelo; Ángela Alarcon Cruz
EPI_ISL_3459400	LDSP CESAR	Instituto Nacional de Salud	Carlos Franco-Muñoz; Carmen Osorio; Diana Malo; Diego A. Álvarez-Díaz; Diego Andrés Prada; Gerardo Santamaría; Hector Alejandro Ruiz-Moreno; Jhonnatan Reales-González; Jorge Rivera; Juan Camilo Martinez; Julian Naizaque; Katherine Laiton-Donato; Lisseth Pardo; Magdalena Wiesner; Marcela Mercado-Reyes; Maria T. Herrera-Sepúlveda; Marta Lopez Blanco; Martha Lucia Ospina Martinez; Paola Rojas; Sergio Gomez; Sheryll Corchuelo; Ángela Alarcon Cruz
EPI_ISL_3320741	LDSP Casanare	Instituto Nacional de Salud	Carlos Franco-Muñoz; Carmen Osorio; Diana Malo; Diego A. Álvarez-Díaz; Diego Andrés Prada; Gerardo Santamaría; Hector Alejandro Ruiz-Moreno; Jhonnatan Reales-González; Jorge Rivera; Juan Camilo Martinez; Julian Naizaque; Katherine Laiton-Donato; Lisseth Pardo; Magdalena Wiesner; Marcela Mercado-Reyes; Maria T. Herrera-Sepúlveda; Marta Lopez Blanco; Martha Lucia Ospina Martinez; Paola Rojas; Sergio Gomez; Sheryll Corchuelo; Ángela Alarcon Cruz
EPI_ISL_2674294, EPI_ISL_2674295	LDSP LETICIA	Instituto Nacional de Salud- Dirección de Investigación en Salud Pública	Carlos Franco-Muñoz; Carmen Osorio; Diana Malo; Diego A. Álvarez-Díaz; Diego Andrés Prada; Gerardo Santamaría; Hector Alejandro Ruiz-Moreno; Jhonnatan Reales-González; Jorge Rivera; Juan Camilo Martinez; Julian Naizaque; Katherine Laiton-Donato; Lisseth Pardo; Magdalena Wiesner; Marcela Mercado-Reyes; Maria T. Herrera-Sepúlveda; Marta Lopez Blanco; Martha Lucia Ospina Martinez; Paola Rojas; Sergio Gomez; Sheryll Corchuelo; Ángela Alarcon Cruz
EPI_ISL_1582987	LDSP VICHADA	Instituto Nacional de Salud- Dirección de Investigación en Salud Pública	Carlos Franco-Muñoz; Carmen Osorio; Diana Malo; Diego A. Álvarez-Díaz; Diego Andrés Prada; Gerardo Santamaría; Hector Alejandro Ruiz-Moreno; Jhonnatan Reales-González; Juan Camilo Martinez; Julian Naizaque; Katherine Laiton-Donato; Lisseth Pardo; Magdalena Wiesner; Marcela Mercado-Reyes; Maria T. Herrera-Sepúlveda; Marta Lopez Blanco; Martha Lucia Ospina Martinez; Paola Rojas; Sergio Gomez; Sheryll Corchuelo; Ángela Alarcon Cruz
EPI_ISL_3074007	LEBOWAKGOMO LABORATORY	National Institute for Communicable Diseases of the National Health Laboratory Service	Amoako DG; Bhiman JN; Everatt J; Ismail A; Mahlangu B; Mnguni A; Mohale T; Ntuli N; Scheepers C
EPI_ISL_2455934, EPI_ISL_2861186, EPI_ISL_3265406	LESP Aguascalientes	Instituto de Diagnostico y Referencia Epidemiologicos (INDRE)	Abril Rodriguez-Maldonado; Ariadna Medina-Benitez; Claudia Wong-Arambula; Ernesto Ramirez-Gonzalez.; Gisela Barrera-Badillo; Irma Lopez-Martinez; Joaquin Quiroz-Mercado; Lucia Hernandez-Rivas; Maribel Gonzalez-Villa; Natividad Cruz-Ortiz; Sergio Rangel-Guerrero; Tatiana Nunez-Garcia; Vanessa Rivero-Arredondo
EPI_ISL_2443047, EPI_ISL_2545807, EPI_ISL_2920685	LESP Baja California	Instituto de Diagnostico y Referencia Epidemiologicos (INDRE)	Abril Rodriguez-Maldonado; Ariadna Medina-Benitez; Claudia Wong-Arambula; Ernesto Ramirez-Gonzalez.; Gisela Barrera-Badillo; Irma Lopez-Martinez; Joaquin Quiroz-Mercado; Lucia Hernandez-Rivas; Natividad Cruz-Ortiz; Sergio Rangel-Guerrero; Tatiana Nunez-Garcia; Vanessa Rivero-Arredondo
EPI_ISL_1651891, EPI_ISL_2937848	LESP Baja California Sur	Instituto de Diagnostico y Referencia Epidemiologicos (INDRE)	Abril Rodriguez-Maldonado; Ariadna Medina-Benitez; Claudia Wong-Arambula; Ernesto Ramirez-Gonzalez.; Gisela Barrera-Badillo; Irma Lopez-Martinez; Joaquin Quiroz-Mercado; Lucia Hernandez-Rivas; Maribel Gonzalez-Villa; Natividad Cruz-Ortiz; Sergio Rangel-Guerrero; Tatiana Nunez-Garcia; Vanessa Rivero-Arredondo
EPI_ISL_1400299, EPI_ISL_2545806	LESP Campeche	Instituto de Diagnostico y Referencia Epidemiologicos (INDRE)	Abril Rodriguez-Maldonado; Ariadna Medina-Benitez; Claudia Wong-Arambula; Ernesto Ramirez-Gonzalez.; Gisela Barrera-Badillo; Irma Lopez-Martinez; Joaquin Quiroz-Mercado; Lucia Hernandez-Rivas; Natividad Cruz-Ortiz; Sergio Rangel-Guerrero; Tatiana Nunez-Garcia; Vanessa Rivero-Arredondo
EPI_ISL_2492489	LESP Chiapas	Instituto de Diagnostico y Referencia Epidemiologicos (INDRE)	Abril Rodriguez-Maldonado; Ariadna Medina-Benitez; Claudia Wong-Arambula; Ernesto Ramirez-Gonzalez.; Gisela Barrera-Badillo; Irma Lopez-Martinez; Joaquin Quiroz-Mercado; Lucia Hernandez-Rivas; Natividad Cruz-Ortiz; Sergio Rangel-Guerrero; Tatiana Nunez-Garcia; Vanessa Rivero-Arredondo
EPI_ISL_2674699	LESP Chihuahua	Instituto de Diagnostico y Referencia Epidemiologicos (INDRE)	Abril Rodriguez-Maldonado; Ariadna Medina-Benitez; Claudia Wong-Arambula; Ernesto Ramirez-Gonzalez.; Gisela Barrera-Badillo; Irma Lopez-Martinez; Joaquin Quiroz-Mercado; Lucia Hernandez-Rivas; Natividad Cruz-Ortiz; Sergio Rangel-Guerrero; Tatiana Nunez-Garcia; Vanessa Rivero-Arredondo
EPI_ISL_2283707	LESP Ciudad de Mexico	Instituto de Diagnostico y Referencia Epidemiologicos (INDRE)	Abril Rodriguez-Maldonado; Ariadna Medina-Benitez; Claudia Wong-Arambula; Ernesto Ramirez-Gonzalez.; Gisela Barrera-Badillo; Irma Lopez-Martinez; Joaquin Quiroz-Mercado; Lucia Hernandez-Rivas; Natividad Cruz-Ortiz; Sergio Rangel-Guerrero; Tatiana Nunez-Garcia; Vanessa Rivero-Arredondo
EPI_ISL_2663345, EPI_ISL_3459926	LESP Colima	Instituto de Diagnostico y Referencia Epidemiologicos (INDRE)	Abril Rodriguez-Maldonado; Ariadna Medina-Benitez; Claudia Wong-Arambula; Ernesto Ramirez-Gonzalez.; Gisela Barrera-Badillo; Irma Lopez-Martinez; Joaquin Quiroz-Mercado; Lucia Hernandez-Rivas; Maribel Gonzalez-Villa; Natividad Cruz-Ortiz; Sergio Rangel-Guerrero; Tatiana Nunez-Garcia; Vanessa Rivero-Arredondo
EPI_ISL_3265625	LESP Estado de Mexico	Instituto de Diagnostico y Referencia Epidemiologicos (INDRE)	Abril Rodriguez-Maldonado; Ariadna Medina-Benitez; Claudia Wong-Arambula; Ernesto Ramirez-Gonzalez.; Gisela Barrera-Badillo; Irma Lopez-Martinez; Joaquin Quiroz-Mercado; Lucia Hernandez-Rivas; Maribel Gonzalez-Villa; Natividad Cruz-Ortiz; Sergio Rangel-Guerrero; Tatiana Nunez-Garcia; Vanessa Rivero-Arredondo
EPI_ISL_2341009, EPI_ISL_2674694, EPI_ISL_3460199	LESP Guanajuato	Instituto de Diagnostico y Referencia Epidemiologicos (INDRE)	Abril Rodriguez-Maldonado; Ariadna Medina-Benitez; Claudia Wong-Arambula; Ernesto Ramirez-Gonzalez.; Gisela Barrera-Badillo; Irma Lopez-Martinez; Joaquin Quiroz-Mercado; Lucia Hernandez-Rivas; Maribel Gonzalez-Villa; Natividad Cruz-Ortiz; Sergio Rangel-Guerrero; Tatiana Nunez-Garcia; Vanessa Rivero-Arredondo
EPI_ISL_3768986	LESP Guerrero	Instituto de Diagnostico y Referencia Epidemiologicos (INDRE)	Abril Rodriguez-Maldonado; Ariadna Medina-Benitez; Claudia Wong-Arambula; Ernesto Ramirez-Gonzalez.; Gisela Barrera-Badillo; Irma Lopez-Martinez; Joaquin Quiroz-Mercado; Lucia Hernandez-Rivas; Maribel Gonzalez-Villa; Natividad Cruz-Ortiz; Sergio Rangel-Guerrero; Tatiana Nunez-Garcia; Vanessa Rivero-Arredondo
EPI_ISL_2859012, EPI_ISL_3265336	LESP Hidalgo	Instituto de Diagnostico y Referencia Epidemiologicos (INDRE)	Abril Rodriguez-Maldonado; Ariadna Medina-Benitez; Claudia Wong-Arambula; Ernesto Ramirez-Gonzalez.; Gisela Barrera-Badillo; Irma Lopez-Martinez; Joaquin Quiroz-Mercado; Lucia Hernandez-Rivas; Maribel Gonzalez-Villa; Natividad Cruz-Ortiz; Sergio Rangel-Guerrero; Tatiana Nunez-Garcia; Vanessa Rivero-Arredondo
EPI_ISL_2496023, EPI_ISL_3460026	LESP Jalisco	Instituto de Diagnostico y Referencia Epidemiologicos (INDRE)	Abril Rodriguez-Maldonado; Ariadna Medina-Benitez; Claudia Wong-Arambula; Ernesto Ramirez-Gonzalez.; Gisela Barrera-Badillo; Irma Lopez-Martinez; Joaquin Quiroz-Mercado; Lucia Hernandez-Rivas; Maribel Gonzalez-Villa; Natividad Cruz-Ortiz; Sergio Rangel-Guerrero; Tatiana Nunez-Garcia; Vanessa Rivero-Arredondo
EPI_ISL_2246832	LESP Michoacan	Instituto de Diagnostico y Referencia Epidemiologicos (INDRE)	Abril Rodriguez-Maldonado; Ariadna Medina-Benitez; Claudia Wong-Arambula; Ernesto Ramirez-Gonzalez.; Gisela Barrera-Badillo; Irma Lopez-Martinez; Joaquin Quiroz-Mercado; Lucia Hernandez-Rivas; Natividad Cruz-Ortiz; Sergio Rangel-Guerrero; Tatiana Nunez-Garcia; Vanessa Rivero-Arredondo
EPI_ISL_2341014	LESP Morelos	Instituto de Diagnostico y Referencia Epidemiologicos (INDRE)	Abril Rodriguez-Maldonado; Ariadna Medina-Benitez; Claudia Wong-Arambula; Ernesto Ramirez-Gonzalez.; Gisela Barrera-Badillo; Irma Lopez-Martinez; Joaquin Quiroz-Mercado; Lucia Hernandez-Rivas; Natividad Cruz-Ortiz; Sergio Rangel-Guerrero; Tatiana Nunez-Garcia; Vanessa Rivero-Arredondo
EPI_ISL_2495934, EPI_ISL_3033452	LESP Nuevo Leon	Instituto de Diagnostico y Referencia Epidemiologicos (INDRE)	Abril Rodriguez-Maldonado; Ariadna Medina-Benitez; Claudia Wong-Arambula; Ernesto Ramirez-Gonzalez.; Gisela Barrera-Badillo; Irma Lopez-Martinez; Joaquin Quiroz-Mercado; Lucia Hernandez-Rivas; Maribel Gonzalez-Villa; Natividad Cruz-Ortiz; Sergio Rangel-Guerrero; Tatiana Nunez-Garcia; Vanessa Rivero-Arredondo
EPI_ISL_2492457, EPI_ISL_2559314, EPI_ISL_3459974	LESP Queretaro	Instituto de Diagnostico y Referencia Epidemiologicos (INDRE)	Abril Rodriguez-Maldonado; Ariadna Medina-Benitez; Claudia Wong-Arambula; Ernesto Ramirez-Gonzalez.; Gisela Barrera-Badillo; Irma Lopez-Martinez; Joaquin Quiroz-Mercado; Lucia Hernandez-Rivas; Maribel Gonzalez-Villa; Natividad Cruz-Ortiz; Sergio Rangel-Guerrero; Tatiana Nunez-Garcia; Vanessa Rivero-Arredondo
EPI_ISL_2246836, EPI_ISL_2340875, EPI_ISL_3265419	LESP Sinaloa	Instituto de Diagnostico y Referencia Epidemiologicos (INDRE)	Abril Rodriguez-Maldonado; Ariadna Medina-Benitez; Claudia Wong-Arambula; Ernesto Ramirez-Gonzalez.; Gisela Barrera-Badillo; Irma Lopez-Martinez; Joaquin Quiroz-Mercado; Lucia Hernandez-Rivas; Maribel Gonzalez-Villa; Natividad Cruz-Ortiz; Sergio Rangel-Guerrero; Tatiana Nunez-Garcia; Vanessa Rivero-Arredondo
EPI_ISL_2545812	LESP Sonora	Instituto de Diagnostico y Referencia Epidemiologicos (INDRE)	Abril Rodriguez-Maldonado; Ariadna Medina-Benitez; Claudia Wong-Arambula; Ernesto Ramirez-Gonzalez.; Gisela Barrera-Badillo; Irma Lopez-Martinez; Joaquin Quiroz-Mercado; Lucia Hernandez-Rivas; Natividad Cruz-Ortiz; Sergio Rangel-Guerrero; Tatiana Nunez-Garcia; Vanessa Rivero-Arredondo
EPI_ISL_2859048	LESP Tabasco	Instituto de Diagnostico y Referencia Epidemiologicos (INDRE)	Abril Rodriguez-Maldonado; Ariadna Medina-Benitez; Claudia Wong-Arambula; Ernesto Ramirez-Gonzalez.; Gisela Barrera-Badillo; Irma Lopez-Martinez; Joaquin Quiroz-Mercado; Lucia Hernandez-Rivas; Maribel Gonzalez-Villa; Natividad Cruz-Ortiz; Sergio Rangel-Guerrero; Tatiana Nunez-Garcia; Vanessa Rivero-Arredondo
EPI_ISL_2545691, EPI_ISL_2559281	LESP Tamaulipas	Instituto de Diagnostico y Referencia Epidemiologicos (INDRE)	Abril Rodriguez-Maldonado; Ariadna Medina-Benitez; Claudia Wong-Arambula; Ernesto Ramirez-Gonzalez.; Gisela Barrera-Badillo; Irma Lopez-Martinez; Joaquin Quiroz-Mercado; Lucia Hernandez-Rivas; Natividad Cruz-Ortiz; Sergio Rangel-Guerrero; Tatiana Nunez-Garcia; Vanessa Rivero-Arredondo
EPI_ISL_2937845	LESP Tlaxcala	Instituto de Diagnostico y Referencia Epidemiologicos (INDRE)	Abril Rodriguez-Maldonado; Ariadna Medina-Benitez; Claudia Wong-Arambula; Ernesto Ramirez-Gonzalez.; Gisela Barrera-Badillo; Irma Lopez-Martinez; Joaquin Quiroz-Mercado; Lucia Hernandez-Rivas; Maribel Gonzalez-Villa; Natividad Cruz-Ortiz; Sergio Rangel-Guerrero; Tatiana Nunez-Garcia; Vanessa Rivero-Arredondo
EPI_ISL_2545756, EPI_ISL_2779133	LESP Yucatan	Instituto de Diagnostico y Referencia Epidemiologicos (INDRE)	Abril Rodriguez-Maldonado; Ariadna Medina-Benitez; Claudia Wong-Arambula; Ernesto Ramirez-Gonzalez.; Gisela Barrera-Badillo; Irma Lopez-Martinez; Joaquin Quiroz-Mercado; Lucia Hernandez-Rivas; Maribel Gonzalez-Villa; Natividad Cruz-Ortiz; Sergio Rangel-Guerrero; Tatiana Nunez-Garcia; Vanessa Rivero-Arredondo
EPI_ISL_2229365	LSUHS Emerging Viral Threat Laboratory	LSUHS Emerging Viral Threat Laboratory	Alexander Mijalis; Andrew D. Yurochko; Christopher G. Kevil; Gregory L. Ware; Jennifer L. Carroll; Jeremy P. Kamil; John A. Vanchiere; Maarten Van Diest; Rona S. Scott
EPI_ISL_2249090	Lab La Salete Robles - VN Famalicao	Instituto Nacional de Saude (INSA) and Institute of Biomedicine (iBiMed), Universidade de Aveiro	Borges et al
EPI_ISL_982296, EPI_ISL_1532923, EPI_ISL_2192355, EPI_ISL_2861474, EPI_ISL_3868444, EPI_ISL_3868984	Lab voor klinische biologie	Lab voor klinische biologie	Bruno Verhasselt; Hannelore Hamerlinck; Marija Janevska
EPI_ISL_2275868	Lab. Microbiologia e Virologia Cotugno A.O. dei Colli - Istituto Zooprofilattico Sperimentale del Mezzogiorno	TIGEM	Antonio Grimaldi Patrizia Annunziata Francesco Panariello Biancamaria Pierri Claudia Tiberio Teresa Giuliano Valentina Bouche Chiara Colantuono Maria Concetta Cuomo Denise Di Concilio Lucio Di Filippo Anna Manfredi Marcello Salvi Antonio Limone Luigi Atripaldi Pellegrino Cerino Andrea Ballabio Davide Cacchiarelli
EPI_ISL_579097, EPI_ISL_626352, EPI_ISL_661263, EPI_ISL_755631, EPI_ISL_1016867, EPI_ISL_1315309, EPI_ISL_1621295, EPI_ISL_1967897, EPI_ISL_2103201, EPI_ISL_2103202, EPI_ISL_2406489, EPI_ISL_2650016, EPI_ISL_2811955, EPI_ISL_2811957, EPI_ISL_2964925, EPI_ISL_2964932, EPI_ISL_2964935, EPI_ISL_3164081, EPI_ISL_3164087, EPI_ISL_3164124, EPI_ISL_3477096, EPI_ISL_3477098, EPI_ISL_3709079			
see above	LabPLUS	Institute of Environmental Science and Research (ESR)	Anja Werno; Antje van der Linden; Arlo Upton; Chris Mansell; David Hammer; Dragana Drinkovic; Erasmus Smit; Gary McAuliffe; Hana Sofia Andersson; Hermes Perez; James Ussher; Jill Sherwood; Jing Wang; Joep de Ligt; Josh Freeman; Julia Howard; Juliet Elvy; Lauren Jelly; Mary DeAlmeida; Matt Blakiston; Matt Storey; Matthew Rogers; Max Bloomfield; Michael Addidle; Michelle Balm; Muhammad Faisal; Nikki Freed; Olin Silander; Olivia Stroeven; Rachel Boyle; Sally Roberts; SallyAnn Harbison; Sarah Jefferies; Sharmini Muttaiyah; Susan Morpeth; Susan Taylor; Timothy Blackmore; Vani Sathyendran; Veronica Playle; Virginia Hope; Xiaoyun Ren
EPI_ISL_548105	LabTests	Institute of Environmental Science and Research (ESR)	Anja Werno; Antje van der Linden; Arlo Upton; Chris Mansell; David Hammer; Dragana Drinkovic; Erasmus Smit; Gary McAuliffe; Hana Sofia Andersson; Hermes Perez; James Ussher; Jill Sherwood; Jing Wang; Joep de Ligt; Josh Freeman; Julia Howard; Juliet Elvy; Lauren Jelly; Mary DeAlmeida; Matt Blakiston; Matt Storey; Matthew Rogers; Max Bloomfield; Michael Addidle; Michelle Balm; Muhammad Faisal; Nikki Freed; Olin Silander; Sally Roberts; Sarah Jefferies; Sharmini Muttaiyah; Susan Morpeth; Susan Taylor; Timothy Blackmore; Vani Sathyendran; Veronica Playle; Virginia Hope; Xiaoyun Ren
EPI_ISL_2445802	Labeto - CAB - Leiria	Instituto Nacional de Saude (INSA)	Borges et al
EPI_ISL_1219962, EPI_ISL_2188367, EPI_ISL_2279647, EPI_ISL_2709261, EPI_ISL_2923153, EPI_ISL_3153499, EPI_ISL_3690330, EPI_ISL_3690543, EPI_ISL_3804397			
see above	Labo Analyses Med	National Reference Center for Viruses of Respiratory Infections, Institut Pasteur, Paris	Angela Brisebarre; Anne-Laure Garand; Claire Capel; Christophe Malabat; Claude Jorion; Corinne Maufrais; DURIVAULT Jérôme; Durivault JéRôMe; Etienne Simon-Lorière; Frédéric Lemoine; HEYM Beate; Hub Bioinformatique Biostatistiques; Hub de Bioinformatique et Biostatistique; Louise Lefrançois; Marie-HéLèNe Glaudon Louveau De La Guigneraye; Marion Barbet; Maud Vanpeene; Méline Bizard; Patricia Tamby; S. Cocco; Sylvie Behillil; Sylvie Van der Werf; Sylvie van der Werf; TAMBY Patricia; Victoire Baillet; Vincent Enouf
EPI_ISL_2657251	Labolatorium Biologi Molekularnej USK	1. Tricity SARS-CoV-2 sequencing consortium: University of Gdansk, Medical University of Gdansk, Vaxican Ltd., Invicta Ltd. 2. National Institute of Public Health - National Institute of Hygiene, Warsaw, Poland	Celina Cybulska; Karolina Gackowska; Katarzyna Groth; Katarzyna Zacharczuk; Krystyna Bienkowska Szewczyk; Lukasz Rabalski; Maciej Grzybek; Maciej Kosinski; Magdalena Nowakowska; Marcin Lubocki; Małgorzata Sadkowska-Todys; Tomasz Wołkowicz

EPI_ISL_3113147	Labor Berlin Charité Vivantes GmbH / Institut für Virologie	Charité Universitätsmedizin Berlin, Institut für Virologie/Labor Berlin	Barbara Mühlemann; Christian Drosten; Christine Stephan; Peter Menzel; Rolf Schwarzer; Terry Jones; Victor M Corman
EPI_ISL_2978187, EPI_ISL_2978191, EPI_ISL_2978200, EPI_ISL_2978220, EPI_ISL_2978221, EPI_ISL_2978224, EPI_ISL_2978236			
see above	Laboratoire National de Santé Publique, Brazzaville, République du Congo	Pathogen Genomics Lab, National Institute for Biomedical Research (INRB)	Amuri Aziza; Andrew Rambaut; Catherine Pratt; Eddy Kinganda-Lusamaki; Edith Nkwembe; Emmanuel Lokilo Lofiko; Fabien Roch Niama; Francisca Muyembe Mawete; Gabriel Kabamba; Ian Goodfellow; Jean Claude Makangara; Jean-Jacques Muyembe Tamfum; Josh Quick; Matthias Pauthner; Michael Wiley; Nick Loman; Placide Mbala-Kingebeni; Raphael Lumembe; Steve Ahuka-Mundeke; Trevor Bedford
EPI_ISL_2157570, EPI_ISL_2492531	Laboratoire National de Santé Publique – LNSP (HAITI - LNSP)	Laboratory of Respiratory Viruses and Measles, Oswaldo Cruz Institute, FIOCRUZ	Alice Sampaio Rocha; Ana Carolina Mendonca; Anna Carolina Paixao; Elisa Cavalcante Pereira; Fernando Motta; Ito Journel; Jaques Boncy; Luciana Appolinario; Marilda Siqueira on behalf of the Fiocruz COVID-19 Genomic Surveillance Network; Paola Resende; Patrick Delly; Renata Serrano Lopes; Taina Venas
EPI_ISL_419562	Laboratoire National de Santé, Microbiology, Virology	Laboratoire National de Santé, Microbiology, Epidemiology and Microbial Genomics	Anke Wienecke-Baldacchino; Ardashel Latsuzbaia; Catherine Ragimbeau; Guillaume Fournier; Jessica Tapp; Joel Mossong; Tamir Abdelrahman; Trung Nguyen Nguyen
EPI_ISL_3365845	Laboratoire Professeur Daniel GAHOUMA (LPDG)	Centre de Recherches Médicales de Lambaréné (CERMEL)	Ayong More; Bertrand Lell; Davy Leger Mouangala; Elvyre Mbongo-Nkama; Emilio Skarwan; Georgelin Nguema Ondo; Guy Stéphane Padzys; Gédéon Prince Manouana; Jean Bernard Lekana-Douki; Joël-Fleury Djoba Siawaya and Ayola Akim Adegnika; Kevine Zang Ella; Ludovic Mewono; Moustapha Nzamba Maloum; Noé Patrick Mbondoukwe; Rodrigue Bikangui; Rodrigue Mintsa Nguema; Sam O'neilla Oye Bingono; Samira Zoa Assoumou; Sandrine Zeh Nfor; Srinivas reddy Pallerla; Steffen Borrmann; Thirumalaisamy P. Velavan
EPI_ISL_1159699	Laboratoire central de Virologie	Laboratoire de Biotechnologie	Abdelomunim Essabbar; Amal Zouaki; Ghizlane EL Amin; Hakima Kabbaj; Lahcen Belyamani and Azeddine Ibrahimi; Mouna Ouadghiri; Myriam Seffar; Saaïd Amzazi; Tarik Aanniz
EPI_ISL_2820514	Laboratoire de Diagnostic, Centre de Soins 1, Caisse Nationale de Sécurité Sociale (CNSS) de Djibouti	IHU Méditerranée Infection	Anthony Levasseur; Christian A. Devaux.; Didier Raoult; Idil Salah abdillahi; Ikram Omar Osman; Iman Abdillahi Hassan; Jeremy Delerce; Linda Houhamdi; Ludivine Brechar; Mohamed Houmed Aboubaker; Philippe Colson; Pierre-Edouard Fournier; Zeinab Ali Waberi
EPI_ISL_660446, EPI_ISL_660448, EPI_ISL_660487, EPI_ISL_660492, EPI_ISL_660497, EPI_ISL_660519, EPI_ISL_2142738			
see above	Laboratoire de Microbiologie CHU Souro Sanou	Centre Muraz	Abdoul-Salam Ouedraogo; Amariane Koné; Armel Poda; Arsène Zongo; Essia Belarbi; Fabian Leendertz; Grit Schubert; Halidou Tinto; Lassana Sangaré; Soumeya Ouangraoua; Thérèse Kagoné; Yacouba Sawadogo; Zekiba Tarnagda
EPI_ISL_2289129	Laboratoire de Microbiologie CHU Souro Sanou	Laboratoire bacteriologie virologie CHUSS	Abdoul-Salam Ouedraogo; Abdoulie Kanteh; Abdul Sesay; Annette Erhart; Armel Poda; François Kiemdé; Halidou Tinto; Mariama Kujabi; Yacouba Sawadogo
EPI_ISL_933715	Laboratoire de Recherche et d'Analyses Medicales de la Gendarmerie Royale	Laboratoire de Recherche et d'Analyses Medicales de la Gendarmerie Royale	Amal Souiri; Momammed Labioui; Nabil Lemzaoui; Sanaâ Lemriss; Saâd El kabbaj; elmostafa El Fahime
EPI_ISL_3239896, EPI_ISL_3253426	Laboratoire de Recherche et d'Analyses Médicales de la Gendarmerie Royale	Laboratoire de Recherche et d'Analyses Médicales de la Gendarmerie Royale	Amal SOUIRI; Hajar LEMRISS; Sanaâ LEMRISS; Saâd EL KABBAJ
EPI_ISL_1790074	Laboratoire de Référence de la TB Douala	Institut Pasteur de Dakar	Carniel Elisabeth; Dia Ndongo; Diagne Moussa Moïse; Diallo Amadou; Diop Mamadou; Faye Ousmane; Loucoubar Cheikh; Ndiaye Ndack; Njouom Richard; Sall Amadou Alpha; Sankhe Safietou
EPI_ISL_1379134, EPI_ISL_2293924, EPI_ISL_2388533, EPI_ISL_2975574, EPI_ISL_3458701, EPI_ISL_3458873, EPI_ISL_3459077			
see above	Laboratoire de santé publique du Québec	Laboratoire de santé publique du Québec	Guillaume Bourque; Ioannis Ragoussis; Jesse Shapiro; Mark Lathrop and Michel Roger on behalf of the CoVSeQ research group; Mark Lathrop and Michel Roger on behalf of the CoVSeQ research group (http://covseq.ca/researchgroup); Sandrine Moreira
EPI_ISL_476830	Laboratoire des Fièvres Hémorragiques Virales du Benin	Charité-Universitätsmedizin Berlin	Anges; Drexler; Jan Felix; Moreira-Soto Andres; Sander Anna-Lena; Yadouleton
EPI_ISL_2932536, EPI_ISL_2932541, EPI_ISL_2932544, EPI_ISL_2932555	Laboratoire des Fièvres Hémorragiques Virales du Benin	Institut für Virologie - Institute of Virology - Charite	Andres Moreira-Soto; Anges Yadouleton; Anna-Lena Sander; Benjamin Hounkpatin and Jan Felix Drexler; Carine Tchibozo; Christian Drosten; Dossou Ange; Eclou Sedjro; Edmilson F de Oliveira Filho; Gildas Hounkanrin; Hinson Fidelia; Keke K. René; Mamoudou Harouna Djingarey; Melchior A. Joël Aïssi; Michael Nagel; Olfert Landt; Praise Adewumi; Salifou Sourakatou; Victor Max Corman; Wendy Karen Jo; Yvette Badou
EPI_ISL_3334656, EPI_ISL_3334657	Laboratoire national de sante, 1 rue Louis Rech, L-3555 Dudelange, Luxembourg	Laboratoire national de sante, Microbiology, Microbial Genomics Platform	Anke Wienecke-Baldacchino; Catherine Ragimbeau; Elodie Solarino; Fatu Djabi; Jessica Tapp; Lise Pignon; Raoul Salmon; Tamir Abdelrahman; Trung Nguyen Nguyen; Virginie Jover
EPI_ISL_2401040	Laboratoire national de sante, Microbiology, Virology	Laboratoire national de sante, Microbiology, Microbial Genomics Platform	Anke Wienecke-Baldacchino; Catherine Ragimbeau; Fatu Djabi; Jessica Tapp; Lise Pignon; Raoul Salmon; Tamir Abdelrahman; Trung Nguyen Nguyen
EPI_ISL_739866, EPI_ISL_744724	Laboratoire national de santé, Microbiology, Virology	Laboratoire national de santé, Microbiology, Microbial Genomics Platform	Anke Wienecke-Baldacchino; Catherine Ragimbeau; Fatu Djabi; Jessica Tapp; Lise Pignon; Raoul Salmon; Tamir Abdelrahman
EPI_ISL_2400871, EPI_ISL_2400920, EPI_ISL_3147199, EPI_ISL_3147660	Laboratoires Reunis	Laboratoire national de sante, Microbiology, Microbial Genomics Platform	Anke Wienecke-Baldacchino; Bernard Weber; Catherine Ragimbeau; Elodie Solarino; Fatu Djabi; Jessica Tapp; Lise Pignon; Raoul Salmon; Tamir Abdelrahman; Virginie Jover
EPI_ISL_3334797, EPI_ISL_3334817, EPI_ISL_3334841	Laboratoires Reunis, 38 Rue Hiel, 6131 Junglinster, Luxembourg	Laboratoire national de sante, Microbiology, Microbial Genomics Platform	Anke Wienecke-Baldacchino; Bernard Weber; Catherine Ragimbeau; Elodie Solarino; Fatu Djabi; Jessica Tapp; Lise Pignon; Raoul Salmon; Tamir Abdelrahman; Virginie Jover
EPI_ISL_2400690, EPI_ISL_3148041, EPI_ISL_3148163	Laboratoires d'analyses medicales - Ketterthill	Laboratoire national de sante, Microbiology, Microbial Genomics Platform	Anke Wienecke-Baldacchino; Caroline Scheiber; Catherine Ragimbeau; Elodie Solarino; Fatu Djabi; Jessica Tapp; Lise Pignon; Raoul Salmon; Serge Vedy; Tamir Abdelrahman; Virginie Jover
EPI_ISL_3432487	Laboratori d'anàlisis clíniques, Hospital Nostra Senyora de Meritxell	LABORATORI NACIONAL EPIDEMIOLOGIA	C; Lobaco et Al
EPI_ISL_3384885, EPI_ISL_3384887, EPI_ISL_3384893	Laboratori d'anàlisis clíniques, Hospital Nostra Senyora de Meritxell	LBM de CHU de Toulouse, Hôpitaux de Toulouse	M; Rendon et Al
EPI_ISL_2645901	Laboratorio Central de Saude Publica do Estado do Para (LACEN/PA)	Laboratory of Respiratory Viruses and Measles, Oswaldo Cruz Institute, FIOCRUZ	Alice Sampaio Rocha; Ana Carolina Mendonca; Anna Carolina Paixao; Elisa Cavalcante Pereira; Fernando Motta; Luciana Appolinario; Marilda Siqueira on behalf of the Fiocruz COVID-19 Genomic Surveillance Network; Paola Resende; Renata Serrano Lopes; Taina Venas; Valnete Andrade
EPI_ISL_964895	Laboratorio Central Mg. Luis Alfredo Pianciola on behalf of 'Proyecto Argentino Interinstitucional de genomica de SARS-CoV-2' (PAIS Consortium)	Laboratorio Central Mg. Luis Alfredo Pianciola on behalf of 'Proyecto Argentino Interinstitucional de genomica de SARS-CoV-2' (PAIS Consortium)	C Pintos; C Ziehm; J Ousset; L Pianciola.; M Fernandez; M Mazzeo; M Nabaes
EPI_ISL_2385445, EPI_ISL_2837027	Laboratorio Central Noel Nutels	Bioinformatics Laboratory / LNCC	Alessandra P Lamarca; Alexandra L Gerber; Amilcar Tanuri; Ana Paula de C Guimaraes; Ana Tereza R Vasconcelos; Andrea Cony Cavalcanti; Caio Luiz Pereira Ribeiro; Cassia Alves; Cintia Policarpo; Claudia Maria Braga de Mello; Cristiane Gomes da Silva; Diana Mariani; Douglas Terra Machado; Flavio Dias da Silva; Gleidson da Silva de Oliveira; Leandro Magalhaes de Souza; Liliane Cavalcante; Luiz G P de Almeida; Marcio Henrique de Oliveira Garcia; Mario Sergio Ribeiro; Ronaldo da Silva F Jr; Silvia Carvalho
EPI_ISL_1662201, EPI_ISL_3568775, EPI_ISL_3568811	Laboratorio Central de Epidemiologia (LCE)	Unidad de Genomica Avanzada	; Alejandra Garcia-Gasca; Alejandra Hernandez-Teran; Alejandro Sanchez-Flores; Alfredo Herrera-Estrella; Alicia Ocana-Mondragon; Alicia OcaÃ±a-Mondragon; Andreu Comas-Garcia; Angel Gustavo Salas-Lais; Antonio Loza Roman; Bernardo Martinez-Miguel; Blanca Taboada; Brenda Irasema Maldonado-Meza; Bruno Gomez-Gil; Carla Ivon Herrera-Najera; Carlos F. Arias; Celia Boukadida; Celida Duque Molina; Celida Martinez- Rodriguez; Clara Esperanza Santacruz-Tinoco; Concepcion Grajales-Muniz; Concepcion Grajales-MuÃ±iz; Consorcio Mexicano de Vigilancia Genomica (CoViGen-Mex). Authors (in alphabetical order): Julio Elias Alvarado-Yaah; Cristobal Chaidez-Quiroz; Daniel Fregoso-Rueda; Daniel Lira Morales; Eduardo Becerril-Vargas; Fernando Fontove-Herrera; Fidencio Mejia-Nepomuceno; Francisco Pulido; Gloria Elena Espinosa-Ayala; Gloria Maria Molina-Salinas; Gloria Vazquez; Hector Esteban Paz-Juarez; Hector Montoya-Fuentes; Helen Haydee Fernanda Ramirez-Plascencia; Irvin Gonzalez-Lopez; Jean Pierre Gonzalez; Jesus Hernandez; Joel Armando Vazquez-Perez.; Jorge Salas-Hernandez; Jose Antonio Enciso-Moreno; Jose Arturo Martinez-Orozco; Jose Esteban Munoz-Medina; Jose Esteban MuÃ±oz-Medina; Jose de Jesus Nunez-Contreras; Jose de Jesus NuÃ±ez-Contreras; Juan Bautista Chale-Dzul; Julissa Enciso-Ibarra; Luis Alberto Ochoa-Carrera; Margarita Matias-Florentino; Maria Guadalupe Santiago-Mauricio; Maria Guadalupe de Jesus Mireles-Rivera; Mario Mujica-Sanchez; Marissa Perez-Garcia; Nelly Selem-Mojica; Pavel Isa; Ricardo Ciria Merce; Ricardo Grande; Rosa Maria Gutierrez Rios; Santiago avila-Rios; Santiago Ávila-Rios; Selene Zarate; Susana Lopez; Veronica Mata-Haro; Victor Eduardo Garcia-Arias; Victor Hugo Borja-Aburto
EPI_ISL_2671638, EPI_ISL_2671736, EPI_ISL_3805485, EPI_ISL_3805507, EPI_ISL_3805519, EPI_ISL_3805521, EPI_ISL_3805527, EPI_ISL_3805538, EPI_ISL_3805571, EPI_ISL_3805641, EPI_ISL_3805689, EPI_ISL_3805698, EPI_ISL_3805732			
see above	Laboratorio Central de Epidemiologia (LCE)	Unidad de Genomica Avanzada	; Alejandra Garcia-Gasca; Alejandra Hernandez-Teran; Alejandro Sanchez-Flores; Alfredo Herrera-Estrella; Alicia Ocaña-Mondragon; Andreu Comas-Garcia; Angel Gustavo Salas-Lais; Antonio Loza Roman; Bernardo Martinez-Miguel; Blanca Taboada; Brenda Irasema Maldonado-Meza; Bruno Gomez-Gil; Carla Ivon Herrera-Najera; Carlos F. Arias; Celia Boukadida; Celida Duque Molina; Celida Martinez- Rodriguez; Clara Esperanza Santacruz-Tinoco; Concepcion Grajales-Muñiz; Consorcio Mexicano de Vigilancia Genomica (CoViGen-Mex). Authors (in alphabetical order): Julio Elias Alvarado-Yaah; Cristobal Chaidez-Quiroz; Daniel Fregoso-Rueda; Daniel Lira Morales; Eduardo Becerril-Vargas; Fernando Fontove-Herrera; Fidencio Mejia-Nepomuceno; Francisco Pulido; Gloria Elena Espinosa-Ayala; Gloria Maria Molina-Salinas; Gloria Vazquez; Hector Esteban Paz-Juarez; Hector Montoya-Fuentes; Helen Haydee Fernanda Ramirez-Plascencia; Irvin Gonzalez-Lopez; Jean Pierre Gonzalez; Jesus Hernandez; Joel Armando Vazquez-Perez.; Jorge Salas-Hernandez; Jose Antonio Enciso-Moreno; Jose Arturo Martinez-Orozco; Jose Esteban Muñoz-Medina; Jose de Jesus Nuñez-Contreras; Juan Bautista Chale-Dzul; Julissa Enciso-Ibarra; Luis Alberto Ochoa-Carrera; Margarita Matias-Florentino; Maria Guadalupe Santiago-Mauricio; Maria Guadalupe de Jesus Mireles-Rivera; Mario Mujica-Sanchez; Marissa Perez-Garcia; Nelly Selem-Mojica; Pavel Isa; Ricardo Ciria Merce; Ricardo Grande; Rosa Maria Gutierrez Rios; Santiago avila-Rios; Selene Zarate; Susana Lopez; Veronica Mata-Haro; Victor Eduardo Garcia-Arias; Victor Hugo Borja-Aburto
EPI_ISL_1351878	Laboratorio Central de EpidemiologÃa (LCE)	Unidad de Genomica Avanzada	Alejandro Sanchez-Flores; Alfredo Herrera-Estrella; Alicia Ocana-Mondragon; Angel Gustavo Salas-Lais; Bernardo Martinez-Miguel; Blanca Taboada; Brenda Irasema Maldonado-Meza; Carla Ivon Herrera-Najera; Carlos F. Arias; Celia Boukadida; Clara Esperanza Santacruz-Tinoco; Concepcion Grajales-Muniz; Consorcio Mexicano de Vigilancia Genomica (CoViGen-Mex). Authors (in alphabetical order): Julio Elias Alvarado-Yaah; Fernando Fontove-Herrera; Francisco Pulido; Gloria Elena Espinoza-Ayala; Gloria Maria Molina-Salinas; Gloria Vazquez; Hector Esteban Paz-Juarez; Hector Montoya-Fuentes; Helen Haydee Fernanda Ramirez-Plascencia; Jorge Ivan Salinal-Nevarez; Jose Antonio Enciso-Moreno; Jose Esteban Munoz-Medina; Jose de Jesus Nunez-Contreras; Juan Bautista Chale-Dzul; Luis Alberto Ochoa-Carrera; Margarita Matias-Florentino; Maria Guadalupe Santiago-Mauricio; Maria Guadalupe de Jesus Mireles-Rivera; Nelly Selem-Mojica; Pavel Isa; Ricardo Grande; Santiago Avila-Rios; Victor Hugo Borja-Aburto
EPI_ISL_2490557	Laboratorio Central de Epidemiología (LCE)	Centro de Investigación en Enfermedades Infecciosas (CIENI), Instituto Nacional de Enfermedades Respiratorias (INER)	; Alejandra Garcia-Gasca; Alejandra Hernández-Terán; Alejandro Sanchez-Flores; Alfredo Herrera-Estrella; Alicia Ocaña-Mondragón; Andreu Comas-García; Angel Gustavo Salas-Lais; Antonio Loza Román; Bernardo Martínez-Miguel; Blanca Taboada; Brenda Irasema Maldonado-Meza; Bruno Gomez-Gil; Carla Ivón Herrera-Najera; Carlos F. Arias; Celia Boukadida; Clara Esperanza Santacruz-Tinoco; Concepción Grajales-Muñiz; Consorcio Mexicano de Vigilancia Genómica (CoViGen-Mex). Authors (in alphabetical order): Julio Elias Alvarado-Yaah; Cristóbal Cháidez-Quiróz; Célida Duque Molina; Célida Martínez- Rodríguez; Daniel Fregoso-Rueda; Daniel Lira Morales; Eduardo Becerril-Vargas; Fernando Fontove-Herrera; Fidencio Mejía-Nepomuceno; Francisco Pulido; Gloria Elena Espinosa-Ayala; Gloria María Molina-Salinas; Gloria Vazquez; Hector Esteban Paz-Juárez; Hector Montoya-Fuentes; Helen Haydee Fernanda Ramírez-Plascencia; Irvin González-López; Jean Pierre González; Joel Armando Vázquez-Pérez.; Jorge Salas-Hernández; José Antonio Enciso-Moreno; José Arturo Martínez-Orozco; José Esteban Muñoz-Medina; José de Jesús Nuñez-Contreras; Juan Bautista Chale-Dzul; Julissa Enciso-Ibarra; Luis Alberto Ochoa-Carrera; Margarita Matías-Florentino; Mario Mújica-Sánchez; Marissa Perez-Garcia; María Guadalupe Santiago-Mauricio; María Guadalupe de Jesús Mireles-Rivera; Nelly Sélem-Mojica; Pavel Isa; Ricardo Ciria Merce; Ricardo Grande; Rosa María Gutierrez Rios; Santiago Ávila-Ríos; Selene Zárate; Susana Lopez; Victor Eduardo Garcia-Arias; Víctor Hugo Borja-Aburto
EPI_ISL_1279539, EPI_ISL_1585621	Laboratorio Central de Epidemiología (LCE)	Instituto Nacional de Enfermedades Respiratorias (INER): Centro de Investigación en Enfermedades Infecciosas (CIENI)	Alejandro Sanchez-Flores; Alfredo Herrera-Estrella; Alicia Ocaña-Mondragón; Angel Gustavo Salas-Lais; Bernardo Martínez-Miguel; Blanca Taboada; Brenda Irasema Maldonado-Meza; Carla Ivón Herrera-Najera; Carlos F. Arias; Celia Boukadida; Clara Esperanza Santacruz-Tinoco; Concepción Grajales-Muñiz; Consorcio Mexicano de Vigilancia Genómica (CoViGen-Mex). Authors (in alphabetical order): Julio Elias Alvarado-Yaah; Consorcio Mexicano de Vigilancia Genómica (CoViGen-Mex). Authors (in alphabetical order): Julio Elias Alvarado-Yaah; Célida Duque Molina; Fernando Fontove-Herrera; Francisco Pulido; Gloria Elena Espinoza-Ayala; Gloria Elena Espinoza-Ayala; Gloria María Molina-Salinas; Gloria Vazquez; Hector Esteban Paz-Juárez; Hector Montoya-Fuentes; Helen Haydee Fernanda Ramírez-Plascencia; Jorge Ivan Salinal-Nevarez; José Antonio Enciso-Moreno; José Esteban Muñoz-Medina; José de Jesús Nuñez-Contreras; Juan Bautista Chale-Dzul; Luis Alberto Ochoa-Carrera; Margarita Matías-Florentino; María Guadalupe Santiago-Mauricio; María Guadalupe de Jesús Mireles-Rivera; Nelly Sélem-Mojica; Pavel Isa; Ricardo Grande; Santiago Ávila-Ríos; Victor Eduardo Garcia-Arias; Víctor Hugo Borja-Aburto
EPI_ISL_1416441, EPI_ISL_1416469, EPI_ISL_1416637, EPI_ISL_1811482, EPI_ISL_2391606, EPI_ISL_2391618, EPI_ISL_2391631, EPI_ISL_2391645, EPI_ISL_2391711, EPI_ISL_2681135, EPI_ISL_2681155, EPI_ISL_2801799, EPI_ISL_2801837, EPI_ISL_2801845, EPI_ISL_2801852, EPI_ISL_3091097, EPI_ISL_3347563, EPI_ISL_3347698, EPI_ISL_3347747, EPI_ISL_3347773, EPI_ISL_3347804			
see above	Laboratorio Central de Epidemiología (LCE)	Instituto de Biotecnología de la UNAM	; Alejandra Garcia-Gasca; Alejandra García-Gasca; Alejandra Hernández-Terán; Alejandro Sanchez-Flores; Alejandro Sánchez-Flores; Alfredo Herrera-Estrella; Alicia Ocaña-Mondragón; Andreu Comas-García; Angel Gustavo Salas-Lais; Antonio Loza Román; Bernardo Martínez-Miguel; Blanca Taboada; Brenda Irasema Maldonado-Meza; Bruno Gomez-Gil; Bruno Gómez-Gil; Carla Ivón Herrera-Najera; Carlos F. Arias; Celia Boukadida; Clara Esperanza Santacruz-Tinoco; Concepción Grajales-Muñiz; Consorcio Mexicano de Vigilancia Genómica (CoViGen-Mex). Authors (in

			alphabetical order): Julio Elias Alvarado-Yaah; Cristóbal Cháidez-Quiróz; Célida Duque Molina; Célida Duque-Molina; Célida Martínez- Rodríguez; Daniel Fregoso-Rueda; Daniel Lira Morales; Eduardo Becerril-Vargas; Fernando Fontove-Herrera; Fidencio Mejía-Nepomuceno; Francisco Pulido; Gloria Elena Espinosa-Ayala; Gloria Elena Espinoza-Ayala; Gloria María Molina-Salinas; Gloria Vazquez; Hector Esteban Paz-Juárez; Hector Montoya-Fuentes; Helen Haydee Fernanda Ramírez-Plascencia; Irvin González-López; Jean Pierre González; Jesús Hernández; Joel Armando Vázquez-Pérez).; Joel Armando Vázquez-Pérez.; Jorge Ivan Salinal-Nevarez; Jorge Salas-Hernández; José Antonio Enciso-Moreno; José Arturo Martínez-Orozco; José Esteban Muñoz-Medina; José de Jesús Nuñez-Contreras; Juan Bautista Chale-Dzul; Julio Elias Alvarado-Yaah; Julissa Enciso-Ibarra; Kathia Elizabeth Tapia-Diaz; Luis Alberto Ochoa-Carrera; Margarita Matías-Florentino; Mario Mújica-Sánchez; Marissa Perez-Garcia; María Guadalupe Santiago-Mauricio; María Guadalupe de Jesús Mireles-Rivera; Nelly Sélem-Mojica; Pavel Isa; Ricardo Ciria Merce; Ricardo Grande; Rosa María Gutierrez Rios; Rosa María Gutiérrez Rios; Santiago Ávila-Ríos; Selene Zárate; Susana Lopez; Verónica Mata-Haro; Victor Eduardo Garcia-Arias; Victor Eduardo García-Arias; Víctor Hugo Borja-Aburto
EPI_ISL_1301468, EPI_ISL_1301636, EPI_ISL_1301672	Laboratorio Central de Epidemiología IMSS	Instituto de Biotecnología de la UNAM	Alejandra Hernández-Terán; Alejandro Sanchez-Flores; Alma Rincón-Rubio; Andrea Santos Coy-Arechavaleta; Authors from IBT; Blanca Taboada; Celia Boukadida; Clara Esperanza Santacruz-Tinoco; Edgar Mendieta-Condado; Eduardo Becerril-Vargas; Fidencio Mejía-Nepomuceno; Francisco Pulido; Gisela Barrera-Badillo; Gloria Vazquez; Hector Esteban Paz-Juárez; IMSS; InDRE and INER (in alphabetical order): Carlos F. Arias; Irma Lopez-Martinez; Jerome Jean Verleyen; Joel Armando Vázquez-Pérez; Jorge Salas-Hernández; José Arturo Martínez-Orozco; José Ernesto Ramírez-González; José Esteban Muñoz-Medina; Larissa Fernandes-Matano; Lucia Hernandez-Rivas; Luis Alberto Ochoa-Carrera; Margarita Matías-Florentino; Mario Mújica-Sánchez; Natividad Cruz-Ortiz; Pavel Isa; Ricardo Grande; Santiago Ávila-Ríos; Tatiana Nunez-Garcia; Teresita Rojas-Mendoza
EPI_ISL_3825482, EPI_ISL_3825483, EPI_ISL_3825486, EPI_ISL_3825487	Laboratorio Central de Salud Publica	Laboratorio de Biología Molecular, Instituto de Medicina Regional on behalf of 'Proyecto Argentino Interinstitucional de genomica de SARS-CoV-2' (PAIS Consortium)	Andrea Ayala; Bettina Brusés; Erica Struss; Esteban Paredes; Griselda Oria; Horacio Lucero.; Javier Mussin; Laura Formichelli; Melina Lorenzini Campos; Raúl Maximiliano Acevedo; Verónica Gómez; Victoria Femenías
EPI_ISL_3586959, EPI_ISL_3586976, EPI_ISL_3586987	Laboratorio Central de Salud Publica de Paraguay	Fundação Ezequiel Dias	Andre Leal; Andrea Gómez de la Fuente; Cynthia Vazquez; Elaine Cristina; Felipe Iani; Flavia Aburjaile; Gislene Garcia de Castro Lichs; Glauco Carvalho; Hegger Fritsch; Joilson Xavier; Juan Torales; Luiz Alcantara.; Luiz Henrique Ferraz Demarchi; Luiz Takao Watanabe; Marina Castilhos Souza Umaki Zardin; Marta Giovanetti; María José Ortega; María Liz Gamarra; Natalia Guimaraes; Raquel da Silva Ferreira; Shirley Villalba; Talita Adelino; Vagner Fonseca; de Oliveira
EPI_ISL_1492648	Laboratorio Central de Salud Publica de Paraguay	Laboratorio Central de Salud Publica de Paraguay	Andrea Gómez de la Fuente; Cynthia Vázquez; Flavia Aburjaile; Juan Torales; Luiz Carlos Junior Alcantara; Marta Giovanetti; María José Ortega; María Liz Gamarra; Shirley Villalba; Talita Adelino; Vagner Fonseca
EPI_ISL_2940230	Laboratorio Central de Salud Pública de la Provincia de Jujuy	Instituto de Patologia Vegetal (CIAP-INTA) on behalf of 'Proyecto Argentino Interinstitucional de genomica de SARS-CoV-2' (PAIS Consortium)	Ariel David Fridman; Claudia Mamani; Debat, HJ.; FD; Fabiana Vaca; Fernández; Marquez, N.; Miguel Alejandro Charre
EPI_ISL_3832480	Laboratorio Central de Saude Publica do Estado da Paraiba (LACEN/PB)	Laboratory of Respiratory Viruses and Measles, Oswaldo Cruz Institute, FIOCRUZ	Alice Sampaio Rocha; Ana Carolina Mendonca; Anna Carolina Paixao; Dalane Loudal Florentino Teixeira; Elisa Cavalcante Pereira; Fernando Motta; Joao Felipe Bezerra; Luciana Appolinario; Marilda Siqueira on behalf of the Fiocruz COVID-19 Genomic Surveillance Network; Paola Resende; Renata Serrano Lopes; Taina Venas
EPI_ISL_2466233	Laboratorio Central de Saude Publica do Estado de Alagoas (LACEN/AL)	Laboratory of Respiratory Viruses and Measles, Oswaldo Cruz Institute, FIOCRUZ	Alice Sampaio Rocha; Ana Carolina Mendonca; Anderson Brandao Leite; Anna Carolina Paixao; Elisa Cavalcante Pereira; Fernando Motta; Luciana Appolinario; Marilda Siqueira on behalf of the Fiocruz COVID-19 Genomic Surveillance Network; Paola Resende; Renata Serrano Lopes; Taina Venas
EPI_ISL_3801904	Laboratorio Central de Saude Publica do Estado de Santa Catarina (LACEN/SC)	Laboratory of Respiratory Viruses and Measles, Oswaldo Cruz Institute, FIOCRUZ	Alice Sampaio Rocha; Ana Carolina Mendonca; Anna Carolina Paixao; Darcita Buerger Rovaris; Elisa Cavalcante Pereira; Fernando Motta; Luciana Appolinario; Marilda Siqueira on behalf of the Fiocruz COVID-19 Genomic Surveillance Network; Paola Resende; Renata Serrano Lopes; Sandra Bianchini Fernandes; Taina Venas
EPI_ISL_3045439	Laboratorio Central de Saude Publica do Estado do Espirito Santo (LACEN/ES)	Laboratory of Respiratory Viruses and Measles, Oswaldo Cruz Institute, FIOCRUZ	Alice Sampaio Rocha; Ana Carolina Mendonca; Anna Carolina Paixao; Elisa Cavalcante Pereira; Fernando Motta; Luciana Appolinario; Marilda Siqueira on behalf of the Fiocruz COVID-19 Genomic Surveillance Network; Paola Resende; Renata Serrano Lopes; Rodrigo Ribeiro Rodrigues; Taina Venas
EPI_ISL_2775494	Laboratorio Central de Saude Publica do Estado do Parana (Instituto de Biologia Molecular do Paraná (LAC)EN-PR)	Instituto Carlos Chagas - Fiocruz	Alessandra De Melo Aguiar; Andreia Akemi Suzukawa; Andréa Rodrigues Ávila; Bruno Dallagiovanna; Dalila Zanette; Eduardo Balsanelli; Emanuel Maltempi de Souza; Fabio Passetti; Fabricio Klerynton Marchini; Fábio de Oliveira Pedrosa; Guilherme Becker; Helisson Faoro; Hellen Geremias dos Santos; Irina Nastassja Riediger; Letusa Albrecht; Lucas Blanes; Luis Gustavo Morello; Lysangela Ronalte Alves; Maria do Carmo Debur; Mauro de Medeiros Oliveira; Michelle Orane Schemberger; Paola Cristina Resende; Sheila Cristina Nardeli; Tiago Gräf; Valter Antônio de Baura
EPI_ISL_3832366	Laboratorio Central de Saude Publica do Estado do Parana (LACEN/PR)	Laboratory of Respiratory Viruses and Measles, Oswaldo Cruz Institute, FIOCRUZ	Agatha Soares; Alice Sampaio Rocha; Ana Carolina Mendonca; Anna Carolina Paixao; Elisa Cavalcante Pereira; Fernando Motta; Ighor Leonardo Arantes; Irina Riediger; Luciana Appolinario; Marilda Siqueira on behalf of the Fiocruz COVID-19 Genomic Surveillance Network; Paola Resende; Renata Serrano Lopes; Taina Venas
EPI_ISL_3832401	Laboratorio Central de Saude Publica do Estado do Rio Grande do Norte (LACEN/RN)	Laboratory of Respiratory Viruses and Measles, Oswaldo Cruz Institute, FIOCRUZ	Agatha Soares; Alice Sampaio Rocha; Ana Carolina Mendonca; Ana Paula Ferreira Costa; Anna Carolina Paixao; Antonnyo Palmielly Diogenes Lima; Aurélio de Oliveira Bento; Elisa Cavalcante Pereira; Fernando Motta; Gessika Brenna Costa Alves; Heglayne Pereira Vital da Silva; Iago de Souza Gomes; Ighor Arantes; Isabelle Cristina Clemente dos Santos; Janaina Sonale Cavalcante Nogueira de Oliveira; Jayra Juliana Paiva Alves Abrantes; Jonas José da Silva; Luciana Appolinario; Marilda Siqueira on behalf of the Fiocruz COVID-19 Genomic Surveillance Network; Paola Resende; Renata Serrano Lopes; Taina Venas; Themis Rocha de Souza; Vítor Gabriel Saldanha Fernandes
EPI_ISL_3539870	Laboratorio Central de Saude Publica do Estado do Rio Grande do Sul (LACEN-RS)	Laboratory of Respiratory Viruses and Measles, Oswaldo Cruz Institute, FIOCRUZ	Alice Sampaio Rocha; Ana Carolina Mendonca; Anna Carolina Paixao; Elisa Cavalcante Pereira; Fernando Motta; Luciana Appolinario; Marilda Siqueira on behalf of the Fiocruz COVID-19 Genomic Surveillance Network; Paola Resende; Renata Serrano Lopes; Richard Salvato; Taina Venas; Tatiana Schaffer Gregianini
EPI_ISL_3033044, EPI_ISL_3033045, EPI_ISL_3033061, EPI_ISL_3033063, EPI_ISL_3033064, EPI_ISL_3033066, EPI_ISL_3033076, EPI_ISL_3260098, EPI_ISL_3260103, EPI_ISL_3260104			
see above	Laboratorio Central de la Ciudad de Santa Fe	Grupo de Genómica y Bioinformática del Instituto de Investigación de la Cadena Láctea CONICET-INTA on behalf of 'Proyecto Argentino Interinstitucional de genomica de SARS-CoV-2' (PAIS Consortium)	AF; Amadio; C; Eberhardt; G; Irazoqui; JM; MF; Mugna; Ojeda; Pastor; Rompato; V
EPI_ISL_2271693, EPI_ISL_2271695	Laboratorio Central, Ministerio de Salud Cordoba	Instituto de Patologia Vegetal (CIAP-INTA) on behalf of 'Proyecto Argentino Interinstitucional de genomica de SARS-CoV-2' (PAIS Consortium)	Barbas, G.; Castro, G.; Debat, HJ.; FD; Fernandez; M; M.B.; Marquez, N.; Pisano; Re, V.
EPI_ISL_792527, EPI_ISL_3230016, EPI_ISL_3230018, EPI_ISL_3860389, EPI_ISL_3860391, EPI_ISL_3860395	Laboratorio Central, Ministerio de Salud Córdoba	Instituto de Patologia Vegetal (CIAP-INTA) on behalf of 'Proyecto Argentino Interinstitucional de genomica de SARS-CoV-2' (PAIS Consortium)	Barbas, G.; Castro, G.; Debat, HJ.; FD; Fernández; M; M.B.; MB; Marquez, N.; Pisano; Re; Re, V.; V
EPI_ISL_2102626	Laboratorio Estatal de Salud Publica de Nuevo Leon	Laboratorio Estatal de Salud Publica de Nuevo Leon	(in alphabetical order) Consuelo Treviño-Garza; Eduardo Isaac de la Rosa-Moreno; Else del Carmen Garcia-Garcia; Gloria Alejandra Jasso-de la Peña; Manuel Enrique de la O-Cavazos; Olín Medina-Chávez; Yulianna Mayre Cordero-Cruz
EPI_ISL_3527791, EPI_ISL_3527799, EPI_ISL_3527801, EPI_ISL_3527803, EPI_ISL_3527812, EPI_ISL_3527816, EPI_ISL_3527818, EPI_ISL_3527827, EPI_ISL_3801286, EPI_ISL_3801289, EPI_ISL_3835365, EPI_ISL_3835373, EPI_ISL_3835378, EPI_ISL_3835385, EPI_ISL_3835387, EPI_ISL_3835388, EPI_ISL_3835392, EPI_ISL_3835397, EPI_ISL_3835415			
see above	Laboratorio Nacional de Salud	Asociación de Salud Integral / Clínica Familiar "Luis Ángel García"	Ana S. Gonzalez-Reiche; Claudia Rangel; Danicela Mercado; Eduardo Arathon; Hilda Ruiz; Luis Aguirre; Luis Rivas; Narda Medina; Oscar Bonilla; Osmar Gamboa.
EPI_ISL_2825113, EPI_ISL_2827791, EPI_ISL_2827830	Laboratorio Nacional de Salud	Laboratorio Nacional de Salud	Gabriela García; Linda Mendoza
EPI_ISL_837595	Laboratorio Nacional de Salud	Laboratory of Respiratory Viruses and Measles, Oswaldo Cruz Institute, FIOCRUZ	Ana Carolina Mendonca; Anna Carolina Paixao; Cesar Roberto Conde Pereira; Claudia Estrada; Fernando Motta; Luciana Appolinario; Marilda Siqueira on behalf of the Fiocruz COVID-19 Genomic Surveillance Network; Paola Resende
EPI_ISL_3045395	Laboratorio Nacional de Salud Pública Dr. Defilló - LNSPDD	Laboratory of Respiratory Viruses and Measles, Oswaldo Cruz Institute, FIOCRUZ	Alice Sampaio Rocha; Ana Carolina Mendonca; Anna Carolina Paixao; Elisa Cavalcante Pereira; Fernando Motta; Grey Benoit Vasquez; Isaac Miguel Sanchez; Ivonne Imbert; Lucia de la Cruz; Luciana Appolinario; Marilda Siqueira on behalf of the Fiocruz COVID-19 Genomic Surveillance Network; Nurys de Castro; Paola Resende; Renata Serrano Lopes; Ronald Skewes; Taina Venas
EPI_ISL_3275191, EPI_ISL_3275231	Laboratorio Nacional de Salud, Ministerio de Salud Publica y Asistencia Social	Genomics and Proteomics Departament, Gorgas Memorial Institute For Health Studies	Alexander A Martinez; Ambar Moreno; Claudia Estrada; Claudia Gonzalez V; César Roberto Conde Pereira; Jessica Gondola; Leyda Abrego; Marlene Castillo; Oris Chavarria
EPI_ISL_2650526, EPI_ISL_2650528, EPI_ISL_2650534	Laboratorio Nacional de Vigilancia de la Salud – Sección de Virologia	Genomics and Proteomics Departament, Gorgas Memorial Institute For Health Studies	Alexander Martinez; Ambar Moreno; Claudia Díaz; Claudia Gonzalez; Jessica Gondola; Leyda Abrego; Marlene Castillo; Mitzi Castro; Oris Chavarria; Sandra Paola Paz; Sofia Carolina Alvarado
EPI_ISL_3320740	Laboratorio Previs IPS	Instituto Nacional de Salud	Carlos Franco-Muñoz; Carmen Osorio; Diana Malo; Diego A. Álvarez-Díaz; Diego Andrés Prada; Gerardo Santamaría; Hector Alejandro Ruiz-Moreno; Jhonnatan Reales-González; Jorge Rivera; Juan Camilo Martinez; Julian Naizaque; Katherine Laiton-Donato; Lisseth Pardo; Magdalena Wiesner; Marcela Mercado-Reyes; Maria T. Herrera-Sepúlveda; Marta Lopez Blanco; Martha Lucia Ospina Martinez; Paola Rojas; Sergio Gomez; Sheryll Corchuelo; Ángela Alarcon Cruz
EPI_ISL_1091261	Laboratorio de Apoyo a la Vigilancia e Investigación Epidemiológica - CIBO	Laboratorio de Infectología Molecular, Departamento de Bioquímica y Medicina Molecular,Facultad de Medicina - Universidad Autónoma de Nuevo León	Alma M. Perez-Rios; Ana M. Rivas-Estilla; Blanca M. Torres; Claudia L. Charles-Niño; Daniel Arellanos-Soto; Diego A. Gonzalez-Altamirano; Hector Montoya-Fuentes; Javier Ramos-Jimenez; Jose J. Figueroa-Delgadillo; Kame A. Galán-Huerta; María F. Herrera-Saldivar; Natalia Martínez-Acuña; Rafael Soto-Alvarez; Sonia A. Lozano-Sepúlveda
EPI_ISL_2802862	Laboratorio de Biologia Molecular, Hospital General de Guayaramerin, Beni, Bolivia	Microbiologia Molecular, Instituto SELADIS, Universidad Mayor de San Andrés	Aneth Vasquez Michel; Carmen Delgado Barrera; Oscar M. Rollano-Peñaloza; Sandra Miranda Sardon
EPI_ISL_2462063	Laboratorio de Biologia Molecular, Hospital San Pedro Claver	Microbiologia Molecular, Instituto SELADIS, Universidad Mayor de San Andrés	Aneth Vasquez Michel; Carmen Delgado Barrera; Oscar M. Rollano-Peñaloza; Sandra Miranda Sardon
EPI_ISL_1278277, EPI_ISL_2600378	Laboratorio de Biologia Molecular, Hospital San Pedro Claver	Molecular Genetics Laboratory, Instituto de Investigaciones Químicas, Universidad Mayor de San Andrés	Aneth Vasquez Michel; Carmen Delgado Barrera; Oscar M. Rollano-Peñaloza; Sandra Miranda Sardon
EPI_ISL_457953, EPI_ISL_457967	Laboratorio de Biología Molecular Asociación Española Primera en Salud	Departments of Pathology and Medicine, New York University School of Medicine	Adriana Heguy; Christian Marier; Gael Westby; Gonzalo Manrique; Maria Noel Zubillaga; Maria Victoria Elizondo; Matthew T Maurano; Paul Zappile
EPI_ISL_2427584, EPI_ISL_2427666	Laboratorio de Biología Molecular Médica Uruguaya	Departments of Pathology and Medicine, New York University School of Medicine	Adriana Heguy; Cecilia Sorhouet; Christian Marier; Dacia Dimartino; Gonzalo Manrique; Maria Cristina Mogdasy; Maria Noel Zubillaga; Maria Victoria Elizondo; Paul Zappile
EPI_ISL_3090033	Laboratorio de Diagnóstico Molecular, Centro de Innovación en Vigilancia Epidemiológica (CiVE), Institut Pasteur Montevideo, Uruguay	Centro de Innovación en Vigilancia Epidemiológica (CiVE), Institut Pasteur Montevideo, Uruguay	Alicia Costábile; Alvaro Fajardo; Ana Moller; Andrés Lizasoain; Belén González; Bernardina Rivera; Cecilia Alonso; Cecilia Salazar; Gonzalo Bello; Gonzalo Moratorio; Gregorio Iraola; Henry Albornoz; Ignacio Ferrés; Javier Hurtado; Juan Zanetti; Julio Medina; Luciana Griffero; Lucía Spangenberg; Ma Noel Bentancor; Ma Pía Techera; Mailen Arleo; Martina Alonso; Matías Maidana; Mauricio Méndez; Melissa Duquía; Mercedes Paz; Natalia Rego; Natalia Reyes; Nicolas Nin; Odhille Chappos; Paula Perbolianachis; Pilar Moreno; Rodney Colina; Rodrigo Arce; Tamara Fernández-Calero; Tania Possi; Veronica Noya; Viviana Bortagaray
EPI_ISL_3707356	Laboratorio de Genómica Microbiana, Universidad Peruana Cayetano Heredia	cov0953	Alejandra Dávila-Barclay; Diego Cuicapuza; Guillermo Salvatierra; Janet Huancachoque; Luis González; Pablo Tsukayama; Pedro E. Romero; Pool Marcos
EPI_ISL_3758939, EPI_ISL_3758941, EPI_ISL_3758949	Laboratorio de Infectologia y Virologia Molecular	Laboratory of Molecular Virology, School of Medicine, Pontificia Universidad Catolica de Chile	Ana Maria Contreras; Andres E. Munoz-Marcos; Carlos Palma; Catalina Pardo-Roa; Constanza Maldonado; Constanza Martinez-Valdevenito; Eileen Serrano; Erick Salinas; Estefany Poblete; Francisco Melo; Jennifer Angulo; Jorge Levican; Leonardo I. Almonacid; M. Belen Leyton; Marcela Ferres; Maria Jose Avendano; Rafael A. Medina; Tamara Garcia-Salum

EPI_ISL_779175	Laboratorio de Infectología, Servicio de Infectología, Hospital Universitario Dr. José Eleuterio González - Universidad Autónoma de Nuevo León	Laboratorio de Infectología Molecular, Departamento de Bioquímica y Medicina Molecular,Facultad de Medicina - Universidad Autónoma de Nuevo León	Adrian Camacho-Ortiz; Ana M. Rivas-Estilla; Daniel Arellanos-Soto; Eduardo Perez-Alba; Elvira Garza-González; Kame A. Galán-Huerta; Laura Nuzzolo-Shihadeh; María F. Herrera-Saldivar; Natalia Martínez-Acuña; Paola Bocanegra-Ibarias; Samantha M. Flores-Treviño; Sonia A. Lozano-Sepúlveda
EPI_ISL_792507	Laboratorio de Inmunología del Hospital Perrando e Instituto de Medicina Regional de la UNNE	Grupo de Genómica y Bioinformática del Instituto de Investigación de la Cadena Láctea CONICET-INTA on behalf of 'Proyecto Argentino Interinstitucional de genomica de SARS-CoV-2' (PAIS Consortium)	A; AF; Amadio; Ayala; Cayré; Deluca; Eberhardt; Foussal; G; Giusiano; Gómez; H; Irazoqui; JM; L; Lescano; Lucero; M; MD; MF; MV; Marin; NA
EPI_ISL_2674333	Laboratorio de Inmunología y Biología Molecular	Instituto Nacional de Salud- Dirección de Investigación en Salud Pública	Carlos Franco-Muñoz; Carmen Osorio; Diana Malo; Diego A. Álvarez-Díaz; Diego Andrés Prada; Gerardo Santamaría; Hector Alejandro Ruiz-Moreno; Jhonnatan Reales-González; Jorge Rivera; Juan Camilo Martinez; Julian Naizaque; Katherine Laiton-Donato; Lisseth Pardo; Magdalena Wiesner; Marcela Mercado-Reyes; Maria T. Herrera-Sepúlveda; Marta Lopez Blanco; Martha Lucia Ospina Martinez; Paola Rojas; Sergio Gomez; Sheryll Corchuelo; Ángela Alarcon Cruz
EPI_ISL_1672551, EPI_ISL_1700675, EPI_ISL_1700687	Laboratorio de Investigaciones de Baney	Swiss Tropical and Public Health Institute	Bonifacio Manguire Nlavo; Carlos Cortes; Claudia Daubenberger; Diosdado Odjama Nseng Ada; Elizabeth Nyakarungu; Guillermo Garcia; Maximilian Mpina; Mitoha Ondo O Ayekaba; Philip Wonder Phiri; Philipp Wagner; Salome Hosch; Tobias Schindler
EPI_ISL_648306, EPI_ISL_648318, EPI_ISL_649164	Laboratorio de Investigaciones de Baney	University Hospital Basel, Clinical Bacteriology	Adrian Egli; Alfredo Mari; Bonifacio Manguire Nlavo; Carlos Cortes; Claudia Daubenberger; Diosdado Odjama Nseng Ada; Elizabeth Nyakarungu; Guillermo Garcia; Helena Seth-Smith; Madlen Stange; Maximilian Mpina; Mitoha Ondo O Ayekaba; Philip Wonder Phiri; Salome Hosch; Tim Roloff; Tobias Schindler
EPI_ISL_2854124, EPI_ISL_2907505	Laboratorio de Medicina Molecular, Universidad de Magallanes	Centro Asistencial Docente y de Investigacion, Universidad de Magallanes	Constanza Ceroni; Diego Alvarez; Inés Cid; Jacqueline Aldridge; Jorge González; Marcelo Navarrete; Roberto Uribe-Paredes
EPI_ISL_3761645, EPI_ISL_3761649, EPI_ISL_3761680	Laboratorio de Pesquisa em Virologia, FAMERP, SJRP	Laboratorio de Pesquisa em Virologia, FAMERP, SJRP	Beatriz de Carvalho Marques; Cecília Artico Banho; Cíntia Bittar; Fábio Sossai Possebon; Guilherme Campos; Helena Lage Ferreira; Jorge A. Petroli Marchesi; João Pessoa Araújo Jr.; Leila Sabrina Ullmann; Lívia Sacchetto; Maisa C. Pereira Parra; Marília Moraes; Maurício L. Nogueira.; Paula Rahal; Paulo Inacio da Costa
EPI_ISL_2462066	Laboratorio de Referencia Departamental en Inmunología, Sedes-Pando	Molecular Genetics Laboratory, Instituto de Investigaciones Químicas, Universidad Mayor de San Andrés	Aneth Vasquez Michel; Carmen Delgado Barrera; Oscar M. Rollano-Peñaloza; Sandra Miranda Sardon
EPI_ISL_1111440	Laboratorio de Referencia Nacional de Enteropatógenos. Instituto Nacional de Salud del Perú	Laboratorio de Referencia Nacional de Enteropatógenos. Instituto Nacional de Salud del Perú	Fiorella Orellana Peralta; Iris Silva Molina; Junior Caro Castro; Ronnie Gavilan Chavez; Veronica Hurtado Vela; Willi Quino Sifuentes
EPI_ISL_489987, EPI_ISL_1093181, EPI_ISL_1532159, EPI_ISL_1532161, EPI_ISL_1532189, EPI_ISL_1534644	Laboratorio de Referencia Nacional de Virus Respiratorio. Instituto Nacional de Salud Perú	Laboratorio de Referencia Nacional de Biotecnología y Biología Molecular. Instituto Nacional de Salud Perú	Carlos Padilla Rojas; Henri Bailon Calderon; Johanna Balbuena Torres; Johanna Balbuena Torrez; Karolyn Chozo Vega; Karolyn Vega Chozo; Luis Barcena; Marco Galarza Perez; Maribel Huaringa Nuñez; Nancy Rojas Serrano; Nancy Rojas Serrano.; Omar Caceres Rey; Priscila Lope Pari
EPI_ISL_1111296	Laboratorio de Referencia Nacional de Virus Respiratorio. Instituto Nacional de Salud Perú	Laboratorio de Referencia Nacional de Enteropatógenos. Instituto Nacional de Salud del Perú	Fiorella Orellana Peralta; Iris Silva Molina; Junior Caro Castro; Ronnie Gavilan Chavez; Veronica Hurtado Vela; Willi Quino Sifuentes
EPI_ISL_540940, EPI_ISL_812459	Laboratorio de Referencia Nacional de Virus Respiratorios, Instituto Nacional de Salud Peru	Laboratorio de Genómica Microbiana, Universidad Peruana Cayetano Heredia	Alejandra Dávila-Barclay; Brenda Ayzanoa; Camila Castillo-Vilcahuaman; Camila Castillo-Vilcahuamán; Guillermo Salvatierra; Janet Huancachoque; Luis González; Maribel Huaringa; Nancy Rojas; Oscar Escalante; Pablo Tsukayama; Pedro E. Romero; Pool Marcos; Priscila Lope
EPI_ISL_3401450, EPI_ISL_3401473, EPI_ISL_3401542, EPI_ISL_3537667, EPI_ISL_3546953, EPI_ISL_3670590, EPI_ISL_3672420, EPI_ISL_3672431, EPI_ISL_3826431, EPI_ISL_3826856, EPI_ISL_3826902, EPI_ISL_3826933, EPI_ISL_3826947, EPI_ISL_3826980			
see above	Laboratorio de Referencia Nacional de Virus Respiratorios. Centro Nacional de Salud Publica. Instituto Nacional de Salud Peru.	Laboratorio de Referencia Nacional de Virus Respiratorios. Centro Nacional de Salud Publica. Instituto Nacional de Salud Peru.	Carlos Padilla Rojas; Henri Bailon Calderon; Iris Silva Molina; Joseph Huayra Niquen; Lely Solari Zerpa; Luis Barcena Flores; Marco Galarza Perez; Nancy Rojas Serrano; Nieves Sevilla Castañeda; Omar Caceres Rey; Orson Mestanza Millones; Princesa Medrano Alhuay; Priscila Lope Pari; Sandra Morales Ruiz; Sara Gordillo Vilchez; Steve Acedo Lazo; Veronica Hurtado Vela; Victor Jimenez Vasquez; Wendy Lizarraga Olivares
EPI_ISL_2536768, EPI_ISL_2536769, EPI_ISL_2536771, EPI_ISL_2844707, EPI_ISL_3023630, EPI_ISL_3376240, EPI_ISL_3376267, EPI_ISL_3376273, EPI_ISL_3376281, EPI_ISL_3376346, EPI_ISL_3376403			
see above	Laboratorio de Referencial Nacional de Virus Respiratorios	Laboratorio de Referencial Nacional de Virus Respiratorios	Carlos Padilla Rojas; Henri Bailon Calderon; Iris Silva Molina; Joseph Huayra Niquen; Lely Solari Zerpa; Luis Barcena Flores; Marco Galarza Perez; Nancy Rojas Serrano; Omar Caceres Rey; Orson Mestanza Millones; Priscila Lope Pari; Sandra Morales Ruiz; Steve Acedo Lazo; Veronica Hurtado Vela
EPI_ISL_3275283	Laboratorio de Vigilancia en Salud Pública el Salvador	Genomics and Proteomics Departament, Gorgas Memorial Institute For Health Studies	Alexander Martinez; Ambar Moreno; Claudia Díaz; Claudia Gonzalez; Denis G Jovel A; Gustavo M Ramirez; Jessica Gondola; Leyda Abrego; Marlene Castillo; Oris Chavarria; Ruth C Vasquez C; Sandra Paola Paz
EPI_ISL_2965572, EPI_ISL_3090040	Laboratorio de Virología Molecular, Centro Universitario Regional del Litoral Norte. Universidad de la República, Salto, Uruguay	Centro de Innovación en Vigilancia Epidemiológica (CiVE), Institut Pasteur Montevideo, Uruguay	Alicia Costábile; Alvaro Fajardo; Ana Moller; Andrés Lizasoain; Belén González; Bernardina Rivera; Cecilia Alonso; Cecilia Salazar; Gonzalo Bello; Gonzalo Moratorio; Gregorio Iraola; Henry Albornoz; Ignacio Ferrés; Javier Hurtado; Juan Zanetti; Julio Medina; Luciana Griffero; Lucía Spangenberg; Ma Noel Bentancor; Ma Pía Techera; Mailen Arleo; Martina Alonso; Matías Maidana; Mauricio Méndez; Melissa Duquía; Mercedes Paz; Natalia Rego; Natalia Reyes; Nicolas Nin; Odhille Chappos; Paula Perbolianachis; Pilar Moreno; Rodney Colina; Rodrigo Arce; Tamara Fernández-Calero; Tania Possi; Veronica Noya; Viviana Bortagaray
EPI_ISL_792177, EPI_ISL_1396215, EPI_ISL_2007500	Laboratorio de Virología del Hospital de Niños Dr. Ricardo Gutierrez	Área de Secuenciación del Laboratorio de Virología del Hospital de Niños Dr. Ricardo Gutierrez on behalf of 'Proyecto Argentino Interinstitucional de genomica de SARS-CoV-2' (PAIS Consortium)	A; AS; Acevedo; Acuña; Alexay; Alvarez Lopez; Barreda Frank; C; D; E; G; Goya; Grandis; Gravis; Jacques; LE; Labarta; Lusso; M; ME; MI; MS; Medina; Mistchenko; N; Nabaes Jodar; Natale; O; S; Streitenberger; Thomas; Valinotto; Viegas, M.; Villegas
EPI_ISL_1396128	Laboratorio de la Dirección de Epidemiología	Área de Secuenciación del Laboratorio de Virología del Hospital de Niños Dr. Ricardo Gutierrez on behalf of 'Proyecto Argentino Interinstitucional de genomica de SARS-CoV-2' (PAIS Consortium)	AG; Acuña; Alexay; Bertone; C; D; Goya; JC; LE; Lusso; M; MI; Nabaes Jodar; Natale; Rechimont; S; Usero; Valinotto; Viegas, M.; Villasana
EPI_ISL_3044157, EPI_ISL_3055566	Laboratorio de salud publica Puebla	LABOPAT	Cynthia Penaloza; Luis Mendoza; Silvia Montilla
EPI_ISL_1396354	Laboratorio del Hospital Regional Ushuaia Gdor. Ernesto Campos	Nodo de Secuenciación Tierra del Fuego - Hospital Regional Ushuaia - Centro Austral De Investigaciones Científicas - Universidad Nacional De Tierra Del Fuego on behalf of 'Proyecto Argentino Interinstitucional de genomica de SARS-CoV-2' (PAIS Consortium)	Alejandro Ezequiel Rojas; Carina Andrea De Roccis; Carolina Beatriz Yulan; Cristina Fernanda Nardi; Fernando Gallego; Gabriel Alejandro Castro; Ivan Dario Gramundi; Manuel Fabian Boutureira; Santiago Guillermo Ceballos; Silvana Beatriz Cáceres
EPI_ISL_791977	Laboratorium Mikrobiologi Samarinda	National Institute of Health Research and Development	AA; HA; HD; Ikawati; KD; KNA; N; Nugraha; Pangesti; Pawestri; Puspa; Puspandari; Setiawaty; Soekarso; Subangkit; T; Tandirogang; V; Yadi
EPI_ISL_2897143	Laboratorium Mikrobiologiczne z Pracownią Cytologiczną Szpital Specjalistyczny w Mielcu	1. Tricity SARS-CoV-2 sequencing consortium: University of Gdansk, Medical University of Gdansk, Vaxican Ltd., Invicta Ltd. 2. National Institute of Public Health - National Institute of Hygiene, Warsaw, Poland	Celina Cybulska; Karolina Gackowska; Katarzyna Groth; Katarzyna Zacharczuk; Krystyna Bienkowska Szewczyk; Lukasz Rabalski; Maciej Grzybek; Maciej Kosinski; Magdalena Nowakowska; Marcin Lubocki; Małgorzata Sadkowska-Todys; Tomasz Wołkowicz
EPI_ISL_3086894	Laboratorium Riset Unsoed	Indonesian Institute of Sciences (LIPI) and Laboratorium Riset Universitas Jenderal Soedirman	Dody Novrial; Endang S. Kusmintarsih; Idris; Indriawati; Isa Nuryana; M. M. Rudi Prihatno; M. Z. Syamsul Hidayat; Puspita Lisdiyanti; Sugiyono Saputra; Syaiful Rizal; Syam B. Iryanto
EPI_ISL_3281521	Laboratory CREAMS (from Russian) in Kindia, Guinea	Department of Microbiology, Russian Anti-Plague Research Institute	A. Yu. Popova; A.A. Kritsky; A.D. Katyshev; A.P. Shevtsova; A.V. Fedorov; E.A. Naryshkina; E.A. Sosedova; E.V. Kazorina; E.V. Kolomoets; M. Keita; N.P. Guseva; S. Keita; S.A. Shcherbakova; V.V. Kutyrev; Ya.M. Krasnov
EPI_ISL_2045671, EPI_ISL_2045805, EPI_ISL_2045813, EPI_ISL_2045841, EPI_ISL_2046560, EPI_ISL_2046599, EPI_ISL_2046602, EPI_ISL_2183731, EPI_ISL_2184150, EPI_ISL_2184941, EPI_ISL_2185426, EPI_ISL_2305099, EPI_ISL_2306587, EPI_ISL_2398034, EPI_ISL_2398337, EPI_ISL_2398582, EPI_ISL_2482157, EPI_ISL_2873174, EPI_ISL_2927206, EPI_ISL_2927727, EPI_ISL_3017067, EPI_ISL_3327656, EPI_ISL_3328522, EPI_ISL_3329005, EPI_ISL_3329878, EPI_ISL_3512464, EPI_ISL_3514152, EPI_ISL_3514843, EPI_ISL_3514969, EPI_ISL_3515927, EPI_ISL_3517402, EPI_ISL_3604638, EPI_ISL_3676373, EPI_ISL_3679391, EPI_ISL_3680020, EPI_ISL_3681592, EPI_ISL_3682422, EPI_ISL_3683477, EPI_ISL_3685927, EPI_ISL_3686439, EPI_ISL_3740712, EPI_ISL_3741037, EPI_ISL_3741930, EPI_ISL_3742316, EPI_ISL_3743123, EPI_ISL_3747750, EPI_ISL_3749939, EPI_ISL_3750159, EPI_ISL_3750307, EPI_ISL_3751995, EPI_ISL_3755692			
see above	Laboratory Corporation of America	Centers for Disease Control and Prevention Division of Viral Diseases, Pathogen Discovery	Adrian Paskey; Amanda Douglas; Amanda Suchanek; Andrea Throop; Ayla Burns; Benjamin Rambo-Martin; Bobbi Croy; Brian Krueger; Brian Norvell; Christopher Gulvick; Christos Petropoulos; Clinton Paden; Clinton R. Paden; Craig Lukasik; Dakota Howard; Darlene Wagner; Debbie Boles; Dhwani Batra; Duncan MacCannell; Eyad Almasri; Goran Stevovic; Howard Engler; Hrushikesh Deshmukh; Jake Humphrey; Jana Schroth; Jason Caravas; Joe Voshell; John Pruitt; Jonathan Meltzer; Jonathan Williams; Kara Moser; Kimberly Wagner; Lax Iyer; Lisa Pfefferle; Lyndon Tilson; Manoj Jain; Marcia Eisenberg; Mary Ann Cristobal; Mary Cristobal; Mary Williamson; Matthew Robinson; Matthew Schmerer; Michael Levandoski; Mike Sapeta; Mindy Nye; Minoo Agarwal; Mohan Kolli; Nuthawin Charoensri; Oren Cohen; Peter Cook; Peter W. Cook; Prashant Gupta; Qian Zeng; Rama Ghatti; Scott Parker; Scott Ryan; Scott Sammons; Shatavia Morrison; Stanley Letovsky; Steven Ragan; Suresh Babu Selvaraju; Suresh Selvaraju; Susan Countryman; Susan Hicks; Suzanne Dale; Thomas Urban; Tim Kuphal; Tricia Zwiefelhofer; Vincent Drouillon; Yvette Unoarumhi
EPI_ISL_541651, EPI_ISL_3102050	Laboratory Diagnostic, Veterinary Specialized Institute Kraljevo	Laboratory Diagnostic, Veterinary Specialized Institute Kraljevo	Afonso, C.; Banovic Djeri, B.; Djukic, V.; Jankovic, M.; Jovanovic, T.; Knezevic, A.; Petrovic, T.; Sekler, M.; Tesovic, B.; Vidanovic, D.; Volkening, J.
EPI_ISL_754906	Laboratory Diagnostics and Clinical Immunology of Developmental Age, Medical University of Warsaw	genXone SA, Research & Development Laboratory; The Faculty of Mathematics, Informatics and Mechanics of the University of Warsaw	Anna Gambin; Grzegorz Nowicki; Jakub Grabowski; Maciej Sykulski; Michał Kaszuba; Monika Mańkowska-Woźniak; Natalia Drwęska-Matelska; Urszula Demkow; Łukasz Krych
EPI_ISL_411915	Laboratory Medicine	Department of Laboratory Medicine, Lin-Kou Chang Gung Memorial Hospital, Taoyuan, Taiwan.	Chung-Guei Huang; Kuo-Chien Tsao; Shin-Ru Shih; Shu-Li Yang; Yhu-Chering Huang; Yi-Chun Li; Yu-Nong Gong
EPI_ISL_1138969, EPI_ISL_1653933	Laboratory for HIV and opportunistic infections diagnosis The Republican Research and Practical Center for Epidemiology and Microbiology (RRPCEM)	Laboratory for HIV and opportunistic infections diagnosis The Republican Research and Practical Center for Epidemiology and Microbiology (RRPCEM)	Alena Mikhalenka; Alexander Kilchevcky; Alina Drozd; Analoly Krasko; Anatoly Krasko; Artur Akhremchuk; Elena Gasich; Katsiaryna Belyakova; Kirill Bulda; Leonid Valentovich; Nastassia Kabankova; Vladimir Gorbunov; Yauhen Sysaliatsin

EPI_ISL_3550693, EPI_ISL_3556740, EPI_ISL_3558549, EPI_ISL_3559403, EPI_ISL_3564524	Laboratory for Molecular Diagnostics, IPHMN	Eurofins Genomics Europe Sequencing GmbH	Danijela Vujošević; Marija Govedarica
EPI_ISL_3631535, EPI_ISL_3665934, EPI_ISL_3696514, EPI_ISL_3707592	Laboratory for Molecular Diagnostics,IPHMN	Eurofins Genomics Europe Sequencing GmbH	Danijela Vujošević
EPI_ISL_435045, EPI_ISL_435047	Laboratory of Applied Genetics	RSE "National Center for Biotechnology"	Alexandr Shevtsov; Asylulan Amirgazin; Ilyas Akhmetollayev; Ruslan Kalendar; Viktoriya Lutsay; Yerlan Ramanculov
EPI_ISL_3769274	Laboratory of Clinical Microbiology, Virology and Bioemergencies, ASST Fatebenefratelli Sacco - Sacco Hospital	Laboratory of Clinical Microbiology, Virology and Bioemergencies, ASST Fatebenefratelli Sacco - Sacco Hospital	Valeria Micheli
EPI_ISL_2376813	Laboratory of Clinical Virology	Greek Genome Center, Biomedical Research Foundation of the Academy of Athens (BRFAA)	Dimitrios Thanos; Emmanouil Athanasiadis; George Sourvinos; Giannis Vatsellas; Katerina Zoi; Theodoros Loupis
EPI_ISL_2365282	Laboratory of Clinical Virology	Greek Genome Center, Biomedical Research Foundation of the Academy of Athens (BRFAA)	Dimitrios Thanos; Emmanouil Athanasiadis; George Sourvinos; Giannis Vatsellas; Katerina Zoi; Theodoros Loupis
EPI_ISL_3453172	Laboratory of Microbiology and Virology, Ospedale Amedeo di Savoia, ASL "Città di Torino"	Laboratory of Microbiology and Virology, Ospedale Amedeo di Savoia, ASL "Città di Torino"	Antonella Bottoni; Elisa Burdino; Francesco Cerutti; Gabriella Gregori; Maria Grazia Milia; Paola Tremante; Tiziano Allice; Valeria Ghisetti
EPI_ISL_654017, EPI_ISL_654019, EPI_ISL_2896981	Laboratory of Microbiology, National Reference Lab, Charles Nicolle Hospital; 2- University of Tunis ElManar, Faculty of Medicine of Tunis, LR99ES09, Tunis, Tunisia	1-Clinical and Experimental Pharmacology Lab, LR16SP02, National Center of Pharmacovigilance, University of Tunis El Manar, Tunis, Tunisia. 2- Neurodegenerative diseases and psychiatric troubles, LR18SP03, Razi Hospital, University of Tunis El Manar, Tunis, Tunisia. 3- Ministry of Health, National Observatory of New and Emerging Diseases, 1006, Tunis, Tunisia	Alia Ben Kahla; Alia Benkahla; Emna Gaies; Gaies Emna; Ilhem Boutiba-Ben Boubaker; Ilhem Boutiba-Ben Boubaker.; Imen Kacem; Imen Mkada; Ines Mdini; Jalila Ben Khelil; Maher Kharrat; Mouna Ben Sassi; Mouna Safer; Nissaf Ben Alaya; Riadh Daghfous; Riadh Gouider; Riadh Gouider.; Roua Ben Othman; Salma Abid; Sameh Trabelsi; Sana Ferjani; Sarra Chammam; Soumaya Rammeh
EPI_ISL_3343233, EPI_ISL_3343988, EPI_ISL_3344064, EPI_ISL_3344067	Laboratory of Molecular Biology and Cancer Immunology	Quadram Institute Bioscience	Abdul K. Sessay; Alexander J. Trotter; Andrew J. Page; Bassam Badran; Georgi Merhi; Hala Abou Naja; Hamad Hasan; Jad Koweyes; Janine M. Wilkinson; Kate A. Makin; Leonardo de Oliveira Martins; Mark Pallen; Mark Webber; Martin Lott; Matthew W. Felgate; Mona Al Buaini; Nabil-Fareed Alikhan; Nada Ghosn; Orla J. Jupp; Rachael Stanley; Rose K. Davidson; Sarah Gardener; Sima Tokajian; Sophie J. Prosolek; Tatiana Tohmeh; Thanh Le-Viet
EPI_ISL_2893220, EPI_ISL_2893222, EPI_ISL_3062345, EPI_ISL_3233197	Laboratory of Molecular Biology and Cancer Immunology, Faculty of Sciences, Lebanese University	Microbial Pathogenomics Lab - LAU	Bassam Badran; Fadi Abdel Sater; Georgi Merhi; Hamad Hassan; Jad Koweyes; Nada Ghosn; Rawan Makki; Sima tokajian; Tatiana Tohmeh
EPI_ISL_2301775	Laboratory of Molecular Biology, Mamatsio General Hospital of Kozani	Greek Genome Center, Biomedical Research Foundation of the Academy of Athens (BRFAA)	Dimitrios Thanos; Emmanouil Athanasiadis; Giannis Vatsellas; Katerina Zoi; Konstantina Gartzonika; Theodoros Loupis
EPI_ISL_801640, EPI_ISL_801705, EPI_ISL_801841	Laboratory of Molecular Virology, Pontificia Universidad Católica de Chile	MSHS Pathogen Surveillance Program	Adolfo Garcia-Sastre; Adriana van De Guchte; Ajay Obla; Ana Maria Contreras; Ana S. Gonzalez-Reiche; Bremy Alburquerque; Carlos Palma; Constanza Maldonado; Edward C. Holmes; Eileen Serrano; Erick Salinas; Hala Alshammary; Harm van Bakel; Jayeeta Dutta; Jorge Levican; Juan Soto; Leonardo I. Almonacid; M. Belen Leyton; Marcela Ferres; Matthew M. Hernandez; Melissa Smith; Rafael A. Medina.; Robert Sebra; Shwetha Hara Sridhar; Tamara García-Salum; Viviana Simon; Ying-Chih Wang; Zenab Khan
EPI_ISL_541353, EPI_ISL_3045508	Laboratory of Respiratory Viruses and Measles, Oswaldo Cruz Institute, FIOCRUZ	Laboratory of Respiratory Viruses and Measles, Oswaldo Cruz Institute, FIOCRUZ	Agatha Cristinne Prudencio; Alice Sampaio Rocha; Ana Carolina Mendonca; Ana Carolina Mendonça; Anna Carolina Paixao; Anna Carolina Paixão; Elisa Cavalcante Pereira; Fernando Motta; Ighor Leonardo Arantes Gomes; Jonathan Lopes; Luciana Appolinario; Marilda Siqueira on behalf of the Fiocruz COVID-19 Genomic Surveillance Network; Paola Resende; Renata Serrano Lopes; Taina Venas
EPI_ISL_941163	Laboratory of Virology and Molecular Diagnostics	Institute of Public Health of Republic of North Macedonia Laboratory of Virology and Molecular Diagnostics	Maja Kuzmanovska
EPI_ISL_451302	Laboratory of Virology, INMI Lazzaro Spallanzani IRCCS	Laboratory of Virology, INMI Lazzaro Spallanzani IRCCS	Antonino Di Caro; Barbara Bartolini; Cesare E.M. Gruber; Francesco Messina; Giuseppe Ippolito; Maria R. Capobianchi; Martina Rueca
EPI_ISL_1341379, EPI_ISL_1341641, EPI_ISL_1365697	Laboratory of Virology, National center of expertise	RSE "National Center of Expertise" and RSE "National center for Biotechnology"	Abdaliyev Askar; Amirgazin Asylulan; Balykbaev Kanat; Kamalova Dinara; Ramankulov Erlan; Sharipova Saule; Shevtsov Alexandr; Tungushbayev Talgat
EPI_ISL_2987561, EPI_ISL_2987564, EPI_ISL_3020337, EPI_ISL_3545681, EPI_ISL_3545687, EPI_ISL_3545707, EPI_ISL_3545717			
see above	Laboratory of virology and molecular diagnostics, Institute of Public Health	Laboratory of virology and molecular diagnostics, Institute of Public Health	Boshevska G; Buzharova T; Janchevska E.; Kuzmanovska M
EPI_ISL_454585	Laboratory of virology, National Center of Expertise	Laboratory of molecular-genetic research, National Center for Expertise, Kazakhstan National Center for Biotechnology, Kazakhstan	; Abdaliyev Askar; Akhmetollayev Ilyas; Amirgazin Asylulan; Aushakhmetova Zabira; Kalendar Ruslan; Lutsay Viktoriya; Rakhmetova Akbota; Ramankulov Yerlan; Shevtsov Alexandr
EPI_ISL_1334517, EPI_ISL_1334570, EPI_ISL_1365036	Laboratory of virology, National center of expertise	RSE "National Center for Biotechnology" and RSE "National Center of Expertise"	Abdaliyev Askar; Amirgazin Asylulan; Balykbaev Kanat; Kamalova Dinara; Ramankulov Yerlan; Sharipova Saule; Shevtsov Alexandr; Tungushbayev Talgat
EPI_ISL_2696312, EPI_ISL_2696321, EPI_ISL_2696354, EPI_ISL_2696629, EPI_ISL_2696713, EPI_ISL_3461287	Laboratory, Gravity Diagnostics	Laboratory, Gravity Diagnostics	Diagnostics, G.
EPI_ISL_576122	Laboratory, The Bio Arte Limited	Laboratory, The Bio Arte Limited	Biazzo, M.; Madeddu, S.; Pinzauti, D.; Santoro, F.
EPI_ISL_2777981, EPI_ISL_3050309	Laboratório Central de Saúde Pública do Amazonas - LACEN-AM	Laboratorio de Ecologia de Doencas Transmissiveis na Amazonia, Instituto Leonidas e Maria Deane - Fiocruz Amazonia	André Corado; Debora Duarte; Felipe Naveca; Fernanda Nascimento; George Silva; Karina Pessoa; Luciana Gonçalves; Maria Júlia Brandão; Matilde Mejía; Michele Jesus; Valdinete Nascimento; Victor Souza; Ágatha Costa
EPI_ISL_2150982, EPI_ISL_2158599	Laboratório de Biologia Molecular Jean Piaget	MRCG at LSHTM, Genomics lab	Abdoulie Kanteh; Abdul Karim Sesay; Adul Candé; Aicha Balde; Aladje Balde; Bakary Sanyang; Bubacar Delgado Pinto Embaló; Dabiri Damilari; Edmira Maria da Costa; Erica Luis Maria Magalhães; Faatu Cassama; Mariama Kujabi; Milanca Agostinho Cá; Paulina Joãozinho da Costa Jarra Manneh; Rei José Pereira; Rui Inndi; Sainabou Laye Ndure; Simão Tchuda Bióté
EPI_ISL_2466172	Laboratório de Biologia Molecular de Doenças Infecciosas e do Câncer (LADIC - UFRN)	Laboratory of Respiratory Viruses and Measles, Oswaldo Cruz Institute, FIOCRUZ	Alice Sampaio Rocha; Ana Carolina Mendonca; Anna Carolina Paixao; Elisa Cavalcante Pereira; Fernando Motta; Josélio Araújo; Luciana Appolinario; Marilda Siqueira on behalf of the Fiocruz COVID-19 Genomic Surveillance Network; Paola Resende; Renata Serrano Lopes; Taina Venas
EPI_ISL_2835167	Laboratório de Biotecnologia Aplicada (LBA) - Laboratório de Biologia Molecular - Hospital das Clínicas, Faculdade de Medicina de Botucatu, Departamento de Bioprocessos e Biotecnologia - Faculdade de Ciências Agronômicas, UNESP – Botucatu/SP	Laboratory of Respiratory Viruses and Measles, Oswaldo Cruz Institute, FIOCRUZ	Alice Sampaio Rocha; Ana Carolina Mendonca; Anna Carolina Paixao; Elisa Cavalcante Pereira; Felipe Allan da Silva Costa; Fernando Motta; Jayme Augusto de Souza Neto; Leonardo Nazario de Moraes; Luciana Appolinario; Marilda Siqueira on behalf of the Fiocruz COVID-19 Genomic Surveillance Network; Paola Resende; Patricia Akemi Assato; Rejane Maria Tommasini; Renata Serrano Lopes; Taina Venas
EPI_ISL_2768036, EPI_ISL_2768045	Labormedizinisches Zentrum Dr Risch	Clinical Bacteriology	Adrian Egli; Alfredo Mari; Fanny Wegner; Hans Hirsch; Helena MB Seth-Smith; Julia Bielicki; Karoline Leuzinger; Lorenz Risch; Manuel Battegay; Martin Risch; Nadia Wohlwend; Tim Roloff
EPI_ISL_2812898, EPI_ISL_3184992, EPI_ISL_3281189, EPI_ISL_3398485	Laverty Pathology	NSW Health Pathology - Institute of Clinical Pathology and Medical Research; Westmead Hospital; University of Sydney	Arnott A.; CIDM-PH et al.; Draper J.; Gall M.; Martinez E.; Rockett R.; Sintchenko V.; on behalf of ICPMR
EPI_ISL_1987063, EPI_ISL_2392238, EPI_ISL_2392466, EPI_ISL_2538426, EPI_ISL_2569427, EPI_ISL_2639670, EPI_ISL_3337826, EPI_ISL_3706406			
see above	Lighthouse Lab in Alderley Park	Wellcome Sanger Institute for the COVID-19 Genomics UK (COG-UK) Consortium	Cordelia Langford; David K. Jackson; Dominic Kwiatkowski; Ewan Harrison; Ian Johnston; Jacquelyn Wynn; Jeffrey Barrett; John Sillitoe on behalf of the Wellcome Sanger Institute COVID-19 Surveillance Team; Mairead Hyland; Roberto Amato; Sonia Goncalves; The Lighthouse Lab in Alderley Park and Alex Alderton
EPI_ISL_557721	Lighthouse Lab in Alderley Park	Wellcome Sanger Institute for the COVID-19 Genomics UK (COG-UK) consortium	Cordelia Langford; David K. Jackson; Dominic Kwiatkowski; Ewan Harrison; Ian Johnston; John Sillitoe on behalf of the Wellcome Sanger Institute COVID-19 Surveillance Team; Roberto Amato; Sonia Goncalves; The Lighthouse Lab in Alderley Park and Alex Alderton
EPI_ISL_3203057, EPI_ISL_3837631	Lighthouse Lab in Glasgow	Wellcome Sanger Institute for the COVID-19 Genomics UK (COG-UK) Consortium	Anna Dominiczak and Alex Alderton; Carol Clugston; Cordelia Langford; David Gray; David K. Jackson; Dominic Kwiatkowski; Ewan Harrison; Harper VanSteenhouse; Ian Johnston; Jeffrey Barrett; John Sillitoe on behalf of the Wellcome Sanger Institute COVID-19 Surveillance Team; Roberto Amato; Sonia Goncalves; Yumi Kasai
EPI_ISL_2317229,	Lighthouse Lab in Milton Keynes	Wellcome Sanger Institute for the COVID-19	Cordelia Langford; David K. Jackson; Dominic Kwiatkowski; Ewan Harrison; Ian Johnston; Jeffrey Barrett; John Sillitoe on behalf of the Wellcome Sanger Institute COVID-19 Surveillance Team; Roberto Amato; Sonia Goncalves; The Lighthouse Lab in Milton Keynes and Alex Alderton

EPI_ISL_3095199		Genomics UK (COG-UK) Consortium	
EPI_ISL_770505	Lithuanian University of Health Sciences Hospital, Department of Laboratory Medicine	Lithuanian University of Health Sciences, Molecular cardiology lab.	Arnoldas Pautienius; Astra Vitkauskiene; Dovydas Gecys; Ingrida Olendraitė; Kamile Tamusauskaite; Laura Pareckaite; Lukas Zemaitis; Vaiva Lesauskaite
EPI_ISL_2156514	Lung Center of the Philippines (LCP)	Philippine Genome Center	Alethea R. de Guzman; Anna Ong-Lim; Arianne A. Zamora; Asia Louisa U. Chong; Benedict A. Maralit; Candice Francheska B. Tambaoan; Carlo M. Lapid; Celia Carlos; Devon Ray Pacial; Edsel Maurice Salvaña; El King D. Morado; Elcid Aaron R. Pangilinan; Eva Maria Cutiongco-de la Paz; Francis A. Tablizo; Irish Coleen A. Asin; Jaime C. Montoya; Jan Michael C. Yap; Jo-Hannah S. Llames; John Q. Wong; Joshua Gregor A. Dizon; Juan Antonio R. Magalang; Karol Sophia Agape R. Padilla; Kenneth M. Kim; Kris P. Punayan; Marc Edsel C. Ayes; Marc Jerrone R. Castro; Maria Rosario Singh-Vergeire and Cynthia P. Saloma; Maria Sofia L. Yangzon; Marissa Alejandria; Razel Nikka M. Hao; Rianna Patricia S. Cruz; Sheila Mae M. Araiza
EPI_ISL_1501827	Lurie Children's Hospital of Chicago	Northwestern University - Ozer Lab	Egon A. Ozer; Judd F. Hultquist; Lacy M. Simons; Larry K. Kociolek; Michael G. Ison; Ramon Lorenzo-Redondo; Taylor J. Dean; William J. Muller; Xiaotian; Zheng
EPI_ISL_3419352	Luzerner Kantonsspital	Institute for Infectious Diseases	Alban Ramette; Christian Baumann; Cora SÃ¤gesser; Franziska Suter-Riniker; Miguel A Terrazos Miani; Pascal Bittel; Peter Keller; Stefan Neuenschwander; Stephen L Leib
EPI_ISL_2443819	M Health Fairview	Minnesota Department of Health, Public Health Laboratory	Alexandra Lorentz; Jacob Garfin; Matt Plumb; and Xiong Wang
EPI_ISL_2495435, EPI_ISL_2495579, EPI_ISL_3356845	MB-Cadham Provincial laboratory	National Microbiology Laboratory (NML)	Anna Majer; Anneliese Landgraff; CanCOGeN's metadata curation team; Darian Hole; David Alexander; Elsie Grudeski; Gary Van Domselaar; Grace Seo; Jared Bullard; Jennifer Tanner; Kerry Dust; Kirsten Biggar; Madison Chapel; Morag Graham; Natalie Knox; Nathalie Bastien; Paul Van Caeseele; Philip Mabon; Public Health Agency of Canada CanCOGeN team; Rhiannon Huzarewich; Russell Mandes; Shari Tyson; Timothy Booth; Yan Li
EPI_ISL_3152348	MD PHL	Maryland Department of Health Laboratories Administration	Ami Patel; Eric N. Keller; Jillian Loomis; Kwang Low; Terence L. Moore; and Robert Myers
EPI_ISL_903380, EPI_ISL_2557251, EPI_ISL_2557459	MOH - Jaber Al-Ahmad Hospital (Innovation Research Laboratory)	MOH - Jaber Al-Ahmad Hospital (Innovation Research Laboratory)	Mohammad Alghounaim; Salman Al-Sabah
EPI_ISL_2346386, EPI_ISL_2346389	MRC/UVRI & LSHTM Uganda Research Unit, Central Public Health Laboratories	MRC/UVRI & LSHTM Uganda Research Unit, Central Public Health Laboratories	Dan Lule Bugembe; Isaac Sseeewanyana; Matthew Cotten; My V.T. Phan; Patrick Semanda; Pontiano Kaleebu; Susan Nabadda
EPI_ISL_3149407, EPI_ISL_3149427	MRC/UVRI & LSHTM Uganda Research Unit, Uganda Virus Research Institute	MRC/UVRI & LSHTM Uganda Research Unit, Uganda Virus Research Institute	; Dan Lule Bugembe; John Kayiwa; Matthew Cotten; My V.T Phan; Pontiano Kaleebu
EPI_ISL_954229, EPI_ISL_1469355, EPI_ISL_1469387	MRC/UVRI & LSHTM Uganda Research Unit	Where sequence data have been generated and submitted to GISAID	Dan Lule Bugembe; Isaac Sseeewanyana; Matthew Cotten; My V.T. Phan; Patrick Semanda; Pontiano Kaleebu; Susan Nabadda
EPI_ISL_3149373, EPI_ISL_3149392, EPI_ISL_3149398, EPI_ISL_3149402, EPI_ISL_3546312	MRC/UVRI & LSHTM Uganda Research Unit , Rakai Health Sciences Program	MRC/UVRI & LSHTM Uganda Research Unit , Rakai Health Sciences Program	; Charles Ssuuna; Matthew Cotten Dan Lule Bugembe; My V.T. Phan; Pontiano Kaleebu; Ronald Moses Galiwango; Steven J Reynolds
EPI_ISL_3546304, EPI_ISL_3546305, EPI_ISL_3546308, EPI_ISL_3546311	MRC/UVRI & LSHTM Uganda Research Unit, Central Public Health Laboratories	MRC/UVRI & LSHTM Uganda Research Unit, Central Public Health Laboratories	Dan Lule Bugembe; Hellen Nansumba; Isaac Sseeewanyana; Matthew Cotten; My V.T. Phan; Patrick Semanda; Pontiano Kaleebu; Susan Nabadda
EPI_ISL_2690464, EPI_ISL_2690470, EPI_ISL_2690472, EPI_ISL_2690477	MRC/UVRI & LSHTM Uganda Research Unit, Central Public Health Laboratories, Rakai Health Sciences Program	MRC/UVRI & LSHTM Uganda Research Unit, Rakai Health Sciences Program	Charles Ssuuna; Dan Lule Bugembe; Matthew Cotten; My V.T. Phan; Pontiano Kaleebu; Ronald Moses Galiwango; Steven J Reynolds
EPI_ISL_3149362	MRC/UVRI & LSHTM Uganda Research Unit, Central Public Health Laboratories	MRC/UVRI & LSHTM Uganda Research Unit, Central Public Health Laboratories	Dan Lule Bugembe; Hellen Nansumba; Isaac Sseeewanyana; Matthew Cotten; My V.T. Phan; Patrick Semanda; Pontiano Kaleebu; Susan Nabadda
EPI_ISL_471161, EPI_ISL_471168, EPI_ISL_561038, EPI_ISL_810982, EPI_ISL_811037, EPI_ISL_1731553, EPI_ISL_2820697, EPI_ISL_2958626, EPI_ISL_2958636, EPI_ISL_2958645, EPI_ISL_2958646, EPI_ISL_2958647, EPI_ISL_3132295, EPI_ISL_3132296, EPI_ISL_3132305, EPI_ISL_3132309, EPI_ISL_3132315, EPI_ISL_3132319, EPI_ISL_3132334, EPI_ISL_3132340, EPI_ISL_3150935, EPI_ISL_3150949			
see above	MRCG at LSHTM Genomics lab	MRCG at LSHTM Genomics lab	Abdoulie Kanteh; Abdul Karim sesay; Bakary Sanyang; Dabiri Damilari; Jarra Manneh; Mariama Kujabi; Sesay et al
EPI_ISL_2920862	MS Public Health Laboratory	University of Mississippi Medical Center, Molecular and Genomics Core Facility	Ashley C. Johnson; D. Ashley Robinson; Ithiel J. Frame; Krishna K. Ayyalasomayajula; Michael R. Garrett; Wenjie Wu
EPI_ISL_2845238	MVZ Dr. Eberhard & Partner Dortmund	Robert Koch Institute	
EPI_ISL_2869802	MVZ Labor Krone GbR	Center of Medical Microbiology, Virology, and Hospital Hygiene, University of Duesseldorf	Alexander Dilthey; Andreas Walker; André Heimbach; Bärbel Lippke; Carsten Tiemann; Dennis Deschka; Janine Silvery; Julia Fazaal; Jörg Timm; Kerstin Ludwig; Klaus Pfeffer; Malte Kohns Vasconcelos; Per Hoffmann; Tobias Wienemann; Torsten Houwaart
EPI_ISL_1936102, EPI_ISL_1936126, EPI_ISL_1936143, EPI_ISL_1936290	Main Chemical Laboratories Egypt Army	Main Chemical Laboratories Egypt Army	Abdullah Salama; AbedElrahman Zekri; Ahmed Gad; Mervat Hassan; Mohamed Abdel-Monem; Mohamed El-Esawi; Mohamed Seadawy; Mohamed Shamel; Sabah Ahmed
EPI_ISL_2802787, EPI_ISL_3298801, EPI_ISL_3425484, EPI_ISL_3544425	Maine Health and Environmental Testing Laboratory	Tewhey Lab, The Jackson Laboratory	Barter, M.; Dewey, H.; H. and Tewhey, R.; Iosue, F.; Lynch, R.; Matluk, N.; Munger
EPI_ISL_3251511	MakLab Laboratorium Diagnostyczne	Wojewodzka Stacja Sanitarno-Epidemiologiczna w Rzeszowie, Laboratorium Diagnostyki Medycznej	Anna Nowakowska; Karolina Ostrowska; Katarzyna Wilk; Marzena Baranowska
EPI_ISL_2875418, EPI_ISL_3065067, EPI_ISL_3065179, EPI_ISL_3603785	Mako Medical	Centers for Disease Control and Prevention Division of Viral Diseases, Pathogen Discovery	Adrian Paskey; Benjamin Rambo-Martin; Christopher Gulvick; Clinton Paden; Clinton R. Paden; Dakota Howard; Darlene Wagner; Dhwani Batra; Duncan MacCannell; Jason Caravas; Kara Moser; Lauren Moon; Matthew Schmerer; Matthew Tugwell; Peter Cook; Peter W. Cook; Scott Sammons; Shatavia Morrison; Yvette Unoarumhi
EPI_ISL_3770668, EPI_ISL_3770672, EPI_ISL_3770673, EPI_ISL_3770679, EPI_ISL_3770687	Malawi Liverpool Wellcome Trust Clinical Research Program	Malawi Liverpool Wellcome Trust CLinical Research Program	Ben Morton; Catherine Anscombe; Kondwani Jambo; Philip Ashton; Sam Lissauer
EPI_ISL_1435808	Malaysia Genome Institute	Malaysia Genome Institute	Azrin Ahmad; Enizza Kasim; Irni Suhayu Sapian; Mohd Faizal Abu Bakar; Mohd Noor Mat Isa; Nor Azfa Johari.; Nurhezreen Md Iqbal; Shamsidar Sopie; Siti Noraini Othman; Yusuf Muhammad Noor
EPI_ISL_2464573	Maryland Genomics, Institute for Genome Sciences, University of Maryland School of Medicine	Maryland Genomics, Institute for Genome Sciences, University of Maryland School of Medicine	Aditya; Claire M; Fraser; Holly; Humphrys; Jacques; Kranthi; Lisa D; Luke J; Mehta; Mike; Ott; Ravel; Roussey; Sadzewicz; Sandra; Tallon; Vavikolanu
EPI_ISL_3453891	Maun Reference Hospital laboratory	Botswana Harvard HIV Reference Laboratory	Boitumelo J.L Zuze; Botshelo Radibe; Dorcas Maruapula; Joseph Makhema; Keoratile Ntshambiwa; Legodile Kooepile; Madisa Mine; Modisa Motswaledi; Mosepele Mosepele; Ontlametse T. Bareng; Roger Shapiro; Sandra Maripe; Shahin Lockman; Sikhulile Moyo; Simani Gaseitsiwe; Thongbotho Mphoyakgosi; Timothy Odirile Matsuokwane; Wonderful T. Choga
EPI_ISL_456377	MedLab Central Ltd	Institute of Environmental Science and Research (ESR)	Anja Werno; Antje van der Linden; Arlo Upton; Chris Mansell; David Hammer; Dragana Drinkovic; Erasmus Smit; Gary McAuliffe; Hana Sofia Andersson; James Ussher; Jill Sherwood; Joep de Ligt; Josh Freeman; Julia Howard; Juliet Elvy; Lauren Jelly; Mary DeAlmeida; Matt Blakiston; Matt Storey; Matthew Rogers; Max Bloomfield; Michael Addidle; Michelle Balm; Sally Roberts; Sarah Jefferies; Sharmini Muttaiyah; Susan Morpeth; Susan Taylor; Timothy Blackmore; Vani Sathyendran; Veronica Playle; Virginia Hope; Xiaoyun Ren
EPI_ISL_482759, EPI_ISL_482769, EPI_ISL_1109624	Medical Ain Shams Research Institute (MASRI), Ain Shams University	Medical Ain Shams Research Institute (MASRI), Ain Shams University	Ahmad Moustafa; Ashraf Omar; Aya Mohamed; Fatma Ebied; Ghada Ismael; Hagar Elshora; Hala Hafez; Hesham Elghazaly; Hoda Ezz Elarab; Iman Foda; Mahmoud Elmeitini; Manal Hamdy Elsaid; Mohamed Elhadidi; Osama Mansour; Osama Mansour.; Reham Kassab; Reham Mamdouh; Samia Abdou Girgis; Sara Elnakeep; Sara Hassan Agwa; Shaimaa Moustafa; Shimaa Moustafa
EPI_ISL_2107525	Medical Laboratory Sciences, Arab American University	Medical Laboratory Sciences, Arab American University	Al-Jawabreh, A.; Al-Jawabreh, H.; Dumaidi, K.; Ereqat, S.; Nasereddin, A.
EPI_ISL_2333359, EPI_ISL_2491386	Medical Microbiology Unit, Department for Laboratory Medicine, Drammen Hospital, Vestre Viken Health Trust,	Norwegian Institute of Public Health, Department of Virology	'Kathrine Stene-Johansen; Atiya R Ali; Debech Nadia; Engebretsen Serina Beate; Garcia Llorente Ignacio; Hilde Elshaug; Hilde Vollan; Jon Bråte; Kamilla Heddeland Instefjord; Karoline Bragstad; Kathrine Stene-Johansen; Line Victoria Moen; Marie Paulsen Madsen; Olav Hungnes; Pedersen Benedikte Nevjen; Rasmus Riis Kopperud
EPI_ISL_2124340	Medizinisches Labor Ostsachsen MVZ GbR Nebenbetriebsstatte Dresden	Robert Koch Institute	
EPI_ISL_3186721, EPI_ISL_3281329, EPI_ISL_3833949	Medlab Pathology	NSW Health Pathology - Institute of Clinical Pathology and Medical Research; Westmead Hospital; University of Sydney	Arnott A.; Draper J.; Gall M.; Martinez E.; Rockett R.; Sintchenko V.; on behalf of ICPMR
EPI_ISL_1158089, EPI_ISL_3129820, EPI_ISL_3446578	Michigan Department of Health and Human Services, Bureau of Laboratories	Michigan Department of Health and Human Services, Bureau of Laboratories	Blankenship HM; Riner D; Soehnlen MK
EPI_ISL_2650294, EPI_ISL_3771104	Microbiologia CATLAB	Can Ruti SARS-CoV-2 Sequencing Hub (HUGTiP/IrsiCaixa/IGTP)	Alba Sánchez; Alexia París; Anna Not; Antoni E Bordoy; Bonaventura Clotet; Cristina Casañ; Cristina Esteban; David Panisello; Francesc Catala-Moll; Gemma Clara; Ignacio Blanco; Laia Soler; Lauro Sumoy; Marc Noguera-Julian; Maria Casadellà; Mariona Parera; Mercedes Guerrero; Montserrat Giménez; Pere-Joan Cardona; Pilar Armengol; Roger Paredes; Verónica Saludes; and Elisa Martró on behalf of the Can Ruti SARS-CoV-2 Sequencing Hub
EPI_ISL_519882, EPI_ISL_521906, EPI_ISL_562942, EPI_ISL_592743, EPI_ISL_962822, EPI_ISL_1033147, EPI_ISL_1250002, EPI_ISL_2379271, EPI_ISL_2379279, EPI_ISL_2379280, EPI_ISL_2379288, EPI_ISL_2405345, EPI_ISL_2405348, EPI_ISL_2405351, EPI_ISL_2482435, EPI_ISL_2482447, EPI_ISL_2482454, EPI_ISL_2839591, EPI_ISL_3050770, EPI_ISL_3050778, EPI_ISL_3050786, EPI_ISL_3050789, EPI_ISL_3050796, EPI_ISL_3050806			

see above	Microbiological Diagnostic Unit - Public Health Laboratory (MDU-PHL)	MDU-PHL	M. B.; M.L.; N.L.; Sait; Sait, M.; Schultz; Schultz M.; Schultz M. B.; Seemann T.; Seemann, T.; Sherry; Sherry, N.
EPI_ISL_2621569	Microbiological Diagnostic Unit - Public Health Laboratory (MDU-PHL)	Microbiological Diagnostic Unit - Public Health Laboratory (MDU-PHL)	M.L.; N.L.; Sait; Seemann T.; Sherry
EPI_ISL_427075	Microbiological Diagnostic Unit Public Health Laboratory	Microbiological Diagnostic Unit Public Health Laboratory	Sait, M.; Schultz M.; Seemann T.; Sherry, N.
EPI_ISL_1372049	Microbiology Department , Late Shri Lakhiram Agrawal Memory Hospital & College, Raigarh, Chhattisgarh	Institute of Life Sciences - INSACOG	Ajay Parida; Amol M. Kanampalliwar; Arup Ghosh; Atimukta Jha; INSACOG Consortium; Punit Prasad; Rajeeb Swain; Rupesh Dash; Safal Walia; Shifu Aggarwal; Sunil K. Raghav
EPI_ISL_3156121, EPI_ISL_3817454	Microbiology Department, Laboratori Clínic Metropolitana Nord. Hospital Universitari Germans Trias i Pujol	Can Ruti SARS-CoV-2 Sequencing Hub (HUGTiP/IrsiCaixa/IGTP)	Alba Sánchez; Alexia París; Anna Not; Antoni E Bordoy; Bonaventura Clotet; Cristina Casañ; David Panisello; Francesc Catala-Moll; Gemma Clara; Ignacio Blanco; Laia Soler; Lauro Sumoy; Marc Noguera-Julian; Maria Casadellà; Mariona Parera; Mercedes Guerrero; Montserrat Giménez; Pere-Joan Cardona; Pilar Armengol; Roger Paredes; Verónica Saludes; and Elisa Martró on behalf of the Can Ruti SARS-CoV-2 Sequencing Hub
EPI_ISL_2312984, EPI_ISL_2508503	Microbiology Division, SC DHEC	Microbiology Division, SC DHEC	Flores, H.; Freeman, J.
EPI_ISL_447303	Microbiology Division, Barzilai University Medical Center	Stern Lab	Stern Lab
EPI_ISL_3545554	Microbiology Laboratory, Attikon University Hospital, Athens	Central National Laboratory ,Public Health Organization	N.Siafakas; S.Pournaras et al
EPI_ISL_3525476	Microbiology, Russian Research Anti-plague Institute 'Microbe'	Microbiology, Russian Research Anti-plague Institute 'Microbe'	A.A.; A.D.; A.V.; E.A.; E.V.; Fedorov; Katyshev; Kazorina; Keita, M.; Keita, S.; Krasnov; Kritsky; Kutyrev; Naryshkina; S.A.; Shcherbakova; Sosedova; V.V.; Y.M.
EPI_ISL_456256, EPI_ISL_548003, EPI_ISL_548145, EPI_ISL_1016844, EPI_ISL_1016876, EPI_ISL_1967899, EPI_ISL_2406492, EPI_ISL_2406493, EPI_ISL_2811944, EPI_ISL_2811945, EPI_ISL_2964922, EPI_ISL_2964933, EPI_ISL_2964937, EPI_ISL_3164077, EPI_ISL_3477082, EPI_ISL_3664441, EPI_ISL_3804595, EPI_ISL_3832963, EPI_ISL_3832975, EPI_ISL_3832996			
see above	Middlemore Hospital	Institute of Environmental Science and Research (ESR)	Anja Werno; Antje van der Linden; Arlo Upton; Chris Mansell; David Hammer; Dragana Drinkovic; Erasmus Smit; Gary McAuliffe; Hana Sofia Andersson; Hermes Perez; James Ussher; Jill Sherwood; Jing Wang; Joep de Ligt; Josh Freeman; Julia Howard; Juliet Elvy; Lauren Jelly; Mary DeAlmeida; Matt Blakiston; Matt Storey; Matthew Rogers; Max Bloomfield; Michael Addidle; Michelle Balm; Muhammad Faisal; Nikki Freed; Olin Silander; Olivia Stroeven; Rachel Boyle; Sally Roberts; SallyAnn Harbison; Sarah Jefferies; Sharmini Muttaiyah; Susan Morpeth; Susan Taylor; Timothy Blackmore; Vani Sathyendran; Veronica Playle; Virginia Hope; Xiaoyun Ren
EPI_ISL_3652187	Ministry Of Health - Central Labs	Ministry Of Health - Central Labs	Eyad Atwa; Mahmoud Al Gazo; Mustafa Abed Rabo; Suhaila Elyan
EPI_ISL_3650535	Ministry Of Health- labs	Ministry Of Health- labs	Eyad Atwa; Mahmoud Al Gazo; Suhaila Elyan
EPI_ISL_718215	Ministry of Health Hospitals	Institute of Health and Community Medicine	Chan Chia Jui; Chua Hock Hin; David Perera; Ooi Mong How; Tonnii Sia Loong Loong; Wong Jyn Shan; Wong Kiing Aik
EPI_ISL_2232666, EPI_ISL_2403271, EPI_ISL_2403272, EPI_ISL_3063334, EPI_ISL_3417037, EPI_ISL_3533176, EPI_ISL_3538349, EPI_ISL_3549200, EPI_ISL_3565595, EPI_ISL_3566323, EPI_ISL_3654710			
see above	Ministry of Health Turkey	Ministry of Health Turkey	Fatma Bayrakdar; Gulay Korukluoglu; Gülay Korukluoğlu; Suleyman Yalcin; Süleyman Yalcin; Yasemin Cosgun; Yasemin Coşgun
EPI_ISL_2604216	Ministry of Health, Jaber Al-Ahmad Hospital	Virology Unit, Department of Microbiology, Faculty of Medicine, Kuwait University	Anfal Al-Adwani; Ebaa Al-Awadhi; Hussain Safar; Nada Madi
EPI_ISL_1712727, EPI_ISL_1713189, EPI_ISL_1713277, EPI_ISL_1713377, EPI_ISL_1713448, EPI_ISL_1713503, EPI_ISL_1713576, EPI_ISL_1713879, EPI_ISL_1713938, EPI_ISL_2408343, EPI_ISL_2842881, EPI_ISL_2843037, EPI_ISL_2843101, EPI_ISL_2843238, EPI_ISL_2843243, EPI_ISL_2843303			
see above	Ministry of Public Health / Hamad Medical Corporation	Biomedical Research Center (BRC), Qatar University / Qatar Genome Project (QGP)	Asmaa A. Al-Thani. MOPH and HMC: Abdullatif Al-Khal; Asmaa A. Al-Thani. MOPH and HMC: Abdullatif Al-Khal; BRC: Fatiha M. Benslimane; Chadi Saad; Dana Al-Batesh; Dina Elgakhlab QGP: Fatima H. Al-Kuwari; Dina Elgakhlab QGP: Fatima H. Al-Kuwari; Einas A. E. Al-Kuwari; Hadi M. Yassine; Hamad E. Al-Romaihi; Hamda Alromaihi; Heba A. Al-Khatib; Mashael A. Al-Bader; Mohammed Al-Thani; Muna A. S. Al-Maslamani; Oal Al-Jamal; Peter V. Coyle; Reham A. El-Kahlout. QBB: Tasneem Al-Hamad; Reham A. El-Kahlout. QBB: Tasneem Al-Hamad; Roberto Bertollini; Salih Al-Marri
EPI_ISL_1714088, EPI_ISL_1714410, EPI_ISL_1714509, EPI_ISL_1714737	Ministry of Public Health / Hamad Medical Corporation	Weill Cornell Medical College - Qatar (WCM-Q), Genomics Core Laboratory / Qatar Genome Project (QGP)	Chadi Saad MOPH and HMC: Abdullatif Al-Khal; Dina Elgakhlab; Einas A. E. Al-Kuwari; Hamad E. Al-Romaihi; Hamda Alromaihi; Joel A Malek. QGP: Fatima H. Al-Kuwari; Laith Abu-Raddad; Mashael A. Al-Bader; Meryem Bensaad; Mohammed Al-Thani; Muna A. S. Al-Maslamani; Peter V. Coyle; Reham A. El-Kahlout. QBB: Tasneem Al-Hamad; Roberto Bertollini; Salih Al-Marri; Shameem Younuskunju; WCMQ: Ayeda A. Ahmed; Yasmin Mohamoud
EPI_ISL_450768	Minnesota Department of Health, Public Health Laboratory	Minnesota Department of Health, Public Health Laboratory	Jacob Garfin; Matt Plumb; and Xiong Wang
EPI_ISL_1706286, EPI_ISL_2304205, EPI_ISL_3161244, EPI_ISL_3503750	Missouri State Public Health Laboratory	Missouri State Public Health Laboratory	Ashley New; Joshua Barry; Matthew Sinn
EPI_ISL_2958895	Mobile County Health Department	Synergy Laboratories	Megan Cornwell
EPI_ISL_520735, EPI_ISL_520738	Mohammed Bin Rashid University of Medicine and Health Sciences	Al Jalila Genomics Center	Abdulmajeed Alkhaja; Abiola Catherine Senok; Ahmad Abou Tayoun; Alawi Alsheikh-Ali; Divinlal Harilal; Hamda Khansaheb; Hanan Al Suwaidi; Mohammed Uddin; Norbert Nowotny; Qutayba Hamid; Rabih Halwani; Rifat Hamoudi; Rupa Murthy Varghese; Sathishkumar Ramaswamy; Tom Loney; Zulfa Omar Deesi
EPI_ISL_2102066, EPI_ISL_2105957, EPI_ISL_2107816, EPI_ISL_2399545, EPI_ISL_2716474, EPI_ISL_2718537, EPI_ISL_2854661, EPI_ISL_2970561, EPI_ISL_2970827, EPI_ISL_3052681			
see above	Molecular Diagnostics Pathology Department Mater Dei Hospital Malta	Molecular Diagnostics Pathology Department Mater Dei Hospital Malta	C Cilia; G Zahra; L Grech; M Briffa; R Borg
EPI_ISL_3255988, EPI_ISL_3256005, EPI_ISL_3256006, EPI_ISL_3314957	Molecular Diagnostics Laboratory	National Reference Laboratory, Nigeria Centre for Disease Control	Anthony Ahumibe; Catherine Okoi; Celestina Obiekea; Chimaobi Chukwu; Dr Chikwe Ihekweazu; Dr Ndodo Nnaemeka; Dr Omoare Adesuyi; Nwando Mba; Olusola Anuoluwapo Akanbi
EPI_ISL_467446	Molecular Diagnostics Services (MDS)	KRISP, KZN Research Innovation and Sequencing Platform	Chimukangara B; Giandhari J; Khan S; Lessells R; Mdlalose K; Pillay S; Tegally H; Wilkinson E; York D; de Oliveira T
EPI_ISL_2626398, EPI_ISL_2626399, EPI_ISL_2626429, EPI_ISL_3101330	Molecular diagnostic laboratory of Federal Budget Institution of Science "Central Research Institute of Epidemiology" of The Federal Service on Customers' Rights Protection and Human Well-being Surveillance	Group of Genomics and Postgenomic Technologies of Central Research Institute of Epidemiology	Akimkin V.G.; Berlina Y.Y.; Bulanenko V.P.; Cherkashina A.S.; Dohoyan A.Y.; Golubeva A.G.; Kaptelova V.V.; Kondrasheva L.Y.; Korneenko E.V.; Nadtoka M.I.; Saenko S.S.; Samoilov A.E.; Shipulina O.Y.; Sinitsyn S.O.; Smirnova Y.S.; Solovieva E.D.; Speranskaya A.S.; Tivanova E.V.; Valdohina A.V.; Zotova M.I.; Zuev S.N.
EPI_ISL_1367677, EPI_ISL_1367692, EPI_ISL_1663669, EPI_ISL_3536601, EPI_ISL_3536603, EPI_ISL_3536604	Molecular diagnostic unit for viral haemorrhagic fevers and emerging viruses, Bouaké CHU Laboratory	Molecular diagnostic unit for viral haemorrhagic fevers and emerging viruses, Bouaké CHU Laboratory	Adjaratou Traoré; Bamba Fatoumata Touré; Chantal Akoua-Koffi; Coulibaly Mbegnan; Diané Bamourou; Essia Belarbi; Etilé Anoh; Fabian Leendertz; Grit Schubert; Kra Ouffoué; Monemo Pacome; Oby Wayoro; Safiatou Karidioula; Soundélé Maïté
EPI_ISL_614349, EPI_ISL_614353, EPI_ISL_614354	Molecular diagnostic unit for viral haemorrhagic fevers and emerging viruses, Bouaké CHU Laboratory	Project group Epidemology of Highly Pathogenic Microorganisms, Robert Koch-Institute	Adjaratou Traoré; Bamba Fatoumata Touré; Chantal Akoua-Koffi; Coulibaly Mbegnan; Diané Bamourou; Essia Belarbi; Etilé Anoh; Fabian Leendertz; Grit Schubert; Kra Ouffoué; Monemo Pacome; Safiatou Karidioula; Soundélé Maïté
EPI_ISL_2533902, EPI_ISL_2710343, EPI_ISL_3133157	Montana Public Health Laboratory	Montana Public Health Laboratory	Carrie Biskupiak; Deborah Gibson; Joy Ritter; Michael Dills; Michelle Mozer
EPI_ISL_2688379, EPI_ISL_3493027, EPI_ISL_3493068	Multiplex DX	Multiplex DX	Diana Drobna; Jakub Kovac; Pavol Cekan; Silvia Rybecka; Veronika Mancikova
EPI_ISL_3007818	N. A. Semashko Republican Clinical Hospital	WHO National Influenza Centre Russian Federation	Andrey Komissarov; Artem Fadeev; Daria Danilenko; Dmitry Lioznov; Elena Nabieva; Georgii Bazykin; Kirill Varchenko; Ksenia Safina; Kseniya Komissarova; Maria Pisareva; Mikhail Bakaev; Nikita Yolshin; Oula Mansour; Tamila Musaeva; Veronika Eder
EPI_ISL_2816309, EPI_ISL_3086695	N.F. Gamaleya Research Center for Epidemiology and Microbiology	WHO National Influenza Centre Russian Federation	Alexander Gintsburg; Alexey Shchetinin; Alina Odintsova; Andrei Botikov; Andrei Pochtovyi; Andrei Siniavin; Andrey Komissarov; Anna Kovyrshina; Artem Fadeev; Artem Tkachuk; Daria Danilenko; Denis Kleymenov; Denis Logunov; Dmitry Lioznov; Dmitry Shcheblyakov; Elena Mazunina; Elena Nabieva; Elena Shidlovskaya; Elizaveta Divisenko; Evgeniia Bykonia; Georgii Bazykin; Inna Dolzhikova; Kirill Varchenko; Ksenia Safina; Kseniya Komissarova; Liubov Popova; Ludmila Vasilchenko; Maria Nikiforova; Maria Pisareva; Mikhail Bakaev; Nadezhda Kuznetsova; Nikita Yolshin; Oula Mansour; Tamila Musaeva; Veronika Eder; Vladimir Gushchin
EPI_ISL_3334211	NB-Hôpital Georges L. Dumont	New Brunswick – Vitalité Health Network	Allain E.; Chacko S.; Crapoulet N.; Desnoyers G.; Garceau R.; Lacroix J.; Lyons P.; Shaw W.
EPI_ISL_605918, EPI_ISL_605922	NGS Lab, DNA SOLUTION LTD.	NGS Lab, DNA SOLUTION LTD.	Chowdhury, M.; H.U.; Haider; Hasan; Hosen; K.N.; Khaleque, A.; Khan; Khan, M.; M.B.; M.F.A.; M.H.; M.I.; Rabbi; Rahman, M.; Razu; Sufian, A.
EPI_ISL_3132538	NHLS Charlotte Maxeke Johannesburg Academic Hospital and the University of the Witwatersrand	KRISP, KZn Research Innovation and Sequencing Platform	Bulelani Manene; Florette Treurnicht; Giandhari Jennifer; Kathleen Subramoney; Naidoo Yeshnee; Pillay Sureshnee; San James; Tegally Houriiyah; Tshabuila Derek; Wilkinson Eduan; Yajna Ramphal; de Oliveira Tulio
EPI_ISL_2162339, EPI_ISL_3746809	NHLS Universitas Academic	UFS Virology	D Goedhals; Emmanuel Ogunbayo; MM Nyaga; MT Mogotsi; P Nthiga; PA Bester; T de Oliveira
EPI_ISL_467509	NHLS-IALCH	KRISP, KZN Research Innovation and Sequencing Platform	Chimukangara B; Giandhari J; Khan S; Lessells R; Mdlalose K; Pillay S; Tegally H; Wilkinson E; York D; de Oliveira T

EPI_ISL_2693055	NHLS_VIRO	KRISP, KZN Research Innovation and Sequencing Platform	Giandhari Jennifer; Naidoo Yeshnee; Pillay Sureshnee; San James; Tegally Houriiyah; Tshabuila Derek; Wilkinson Eduan; Yajna Ramphal; de Oliveira Tulio
EPI_ISL_2693891, EPI_ISL_3275369	NHLS_VIRO Eastern Cape	KRISP, KZN Research Innovation and Sequencing Platform	Giandhari Jennifer; Laguda-Akingba O; Naidoo Yeshnee; Pillay Sureshnee; San James; Tegally Houriiyah; Tshabuila Derek; Wilkinson Eduan; Yajna Ramphal; de Oliveira Tulio
EPI_ISL_420037	NIC Viral Respiratory Unit - Institut Pasteur of Algeria	National Reference Center for Viruses of Respiratory Infections, Institut Pasteur, Paris	Angela Brisebarre; Etienne Simon-Lorière; Fawzi Derrar; Flora Donati; Marion Barbet; Maud Vanpeene; Mélanie Albert; Méline Bizard; Sylvie Behillil; Sylvie van der Werf; Vincent Enouf
EPI_ISL_3161806, EPI_ISL_3161807, EPI_ISL_3161808, EPI_ISL_3161809, EPI_ISL_3375624, EPI_ISL_3375625, EPI_ISL_3375628, EPI_ISL_3375630, EPI_ISL_3375633, EPI_ISL_3375634			
see above	NIC, Viral Respiratory Unit	Virology Departement	Aicha Bensalem; Aissam Hachid; Alia Grad; Amel Benyahia; Fawzi Derrar; Fayez Ahmed Khardine; Fayez Khardine; Fetouma Doudou; Mohamed Amine Beloufa; Nardjes Hihi
EPI_ISL_2968090	NILMRC	Genomic Research Laboratory, Bangabandhu Sheikh Mujib Medical University	Arifa Akram; Laila Anjuman Banu; Md.Saydur Rahman; Md.Sharfuddin Ahmed; Sharadindu Kanti Sinha
EPI_ISL_2484594, EPI_ISL_2484598, EPI_ISL_3150970	NL-Dr. Leonard A. Miller Centre for Health Services	National Microbiology Laboratory (NML)	Adel Malek; Anna Majer; Anneliese Landgraff; CanCOGeN's metadata curation team; Darian Hole; Elsie Grudeski; Gary Van Domselaar; George Zahariadis; Grace Seo; Jennifer Tanner; Kerri Smith; Kirsten Biggar; Laura Gilbert; Madison Chapel; Morag Graham; Natalie Knox; Nathalie Bastien; Philip Mabon; Public Health Agency of Canada CanCOGeN team; Rhiannon Huzarewich; Robert Needle; Russell Mandes; Shari Tyson; Timothy Booth; Yan Li; Yang Yu
EPI_ISL_422390, EPI_ISL_422402	NMIMR, Department of Virology	WACCBIP, University of Ghana	Abraham Kwabena Anang; Augustina Arjarquah; Bright Adu; Collins M. Morang'a; Dominic S. Y. Amuzu; Erasmus Kotey; Evelyn Bonney; Fred Tei-Maya; George B. Kyei; Gordon A. Awandare; Ivy Asante; Joyce M. Ngoi; Kofi Bonney; Linda Boatemaa; Miriam Eshun; Peter Quashie; Selassie Kumordjie; Vanessa Magnussen; William Ampofo
EPI_ISL_2835584, EPI_ISL_3399714, EPI_ISL_3399759	NS-QEII Health Sciences Centre	National Microbiology Laboratory (NML)	Anna Majer; Anneliese Landgraff; CanCOGeN's metadata curation team; Dan Gaston; Darian Hole; Elsie Grudeski; Gary Van Domselaar; Grace Seo; Janice Pettipas; Jason LeBlanc; Jennifer Tanner; Kirsten Biggar; Madison Chapel; Morag Graham; Natalie Knox; Nathal; Nathalie Bastien; Philip Mabon; Public Health Agency of Canada CanCOGeN team; Rhiannon Huzarewich; Russell Mandes; Shari Tyson; Timothy Booth; Todd Hatchette; Yan Li
EPI_ISL_1626499	NSTU COVID-19 Diagnostic Center	NSU Genome Research Institute (NGRI)	Abdul Khaleque; Abdus Sadique; Aura Rahman; Firoz Ahmed; Hasan Mahmud Reza; Kazi Nadim Hasan; Maqsud Hossain; Md. Aminul Islam; Mohammad Salim Hossain; Newaz Mohammed Bahadur; Syeda Naushin Tabassum; Tahrima Saiha Huq
EPI_ISL_632312	NU-sjukvården	Clinical microbiology, Sahlgrenska University Hospital	Hedvig Engström Jakobsson; Johan Ringlander; Josefin Olausson; Magnus Lindh
EPI_ISL_2274679	NYC Pandemic Response Lab	Wadsworth Center, New York State Department of Health	Alexis Russell; Catharine Prussing; Daryl M. Lamson; Erasmus Schneider; Erica Lasek-Nesselquist; John Kelly; Jonathan Plitnick; Kirsten St. George; Matthew Shudt; Melissa A Leisner; Navjot Singh
EPI_ISL_2509496, EPI_ISL_2614811	Nacionalinis maisto ir veterinarijos rizikos vertinimo institutas	National Public Health Surveillance Laboratory	Ana Steponkiene; Danas Baksa; Jelena Razmuk; Lukas Vasionis; Lukas Zemaitis; Migle Gabrielaite; Svajune Muralyte
EPI_ISL_2798962	Nacionālais medicīnas serviss - laboratorija, SIA	Riga East University Hospital, National Microbiology Reference Laboratory; Eurofins Genomics Europe Sequencing GmbH	Arzu Algulieva; Diāna Dušacka; Dārta Pūpola; Ilva Pole; Inita Balta; Jevgenijs Bodrenko; Jūlija Čevere; Natalja Mikena; Reinis Vangravs; Reinis Zeltmatis; Sergejs Ņikišins; Ģirts Šķenders
EPI_ISL_3061301, EPI_ISL_3401987	Nastavni zavod za javno zdravstvo Primorsko- Goranske Županije	Hrvatski zavod za javno zdravstvo	Irena Tabain; Ivana Ferenčak
EPI_ISL_2532603	Nationa Laboratory of Health, Environment and Food	Department of Public Health Microbiology Ljubljana, National Laboratory for Health, Environment and Food	José Gonçalves; Katarina Prosenc; Marija Trkov; Martin Bosilj; Metka Paragi; Natasa Berginc; Tom Koritnik; Verica Mioč
EPI_ISL_3064702, EPI_ISL_3064707, EPI_ISL_3064708, EPI_ISL_3064709	National Agency for Public Health, Republic of Moldov	Charité Universitätsmedizin Berlin, Institut für Virologie	Ala Halacu; Barbara Mühlemann; Christian Drosten; Julia Schneider; Jörn Beheim-Schwarzbach; Mariana Apostol; Talitha Veith; Terry Jones; Victor M Corman
EPI_ISL_2894995, EPI_ISL_2894996	National Agency for Public Health, Republic of Moldova	Charité Universitätsmedizin Berlin, Institut für Virologie	Ala Halacu; Barbara Mühlemann; Christian Drosten; Julia Schneider; Julia Tesch; Jörn Beheim-Schwarzbach; Mariana Apostol; Talitha Veith; Terry Jones; Tobias Bleicker; Victor M Corman
EPI_ISL_1805651, EPI_ISL_1805740, EPI_ISL_1805957	National Center for Communicable Diseases (NCCD) National Influenza Center	National Center for Communicable Diseases (NCCD) National Influenza Center	Ankhbayar S; Battur L; Bayasgalan N; Darmaa B; Hideka M; Khishigmunkh Ch; Mina N; Naranzul Ts; Nymadawa P; Seiichiro F; Shinji W; Tsogzolmaa G
EPI_ISL_1805697	National Center for Communicable Diseases (NCCD) National Influenza Center	National Centre for Disease Control (NCDC) National Influenza Center	Ankhbayar S; Battur L; Bayasgalan N; Darmaa B; Hideka M; Khishigmunkh Ch; Mina N; Naranzul Ts; Nymadawa P; Seiichiro F; Shinji W; Tsogzolmaa G
EPI_ISL_1231756	National Center for Infectious and Parasitic Diseases (NCIPD)	National Center for Infectious and Parasitic Diseases (NCIPD)	Alexiev et al
EPI_ISL_1718284, EPI_ISL_1718304	National Center of Disease Control and Prevention of the Republic of Armenia	Institute of Molecular Biology NAS RA, Republic of Armenia, Department of Bioengineering, BioinformaticsInstitute and Molecular Biology IBMPh RAU, Republic of Armenia	Andranik Chavushyan; Arsen Arakelyan; Diana Avetyan; Gayane Melik-Pashayan; Gisane Khachatyan; Hovsep Ghazaryan; Maria Nikoghosyan; Nelli Muradyan; Roksana Zakharyan; Shushan Sargrsyan; Siras Hakobyan; Tamara Sirunyan
EPI_ISL_1854612, EPI_ISL_1854640	National Center of Disease Control and Prevention of the Republic of Armenia	UW Virology Lab	Alexander Greninger; Anahit Hovhannisyan; Andranik Chavushyan; Anna Khazaryan; Arsen Arakelyan; Diana Avetyan; Gisane Khachatyan; Hong Xie; Hovsep Ghazaryan; Keith R. Jerome; Lasata Shrestha; Lyudmila Niazyan; Maria Nikoghosyan; Meei-Li Huang; Michelle Lin; Nelli Muradyan; Pavitra Roychoudhury; Roksana Zakharyan; Shah Mohamed Bakhash; Siras Hakobyan; Tamara Sirunyan
EPI_ISL_3543605, EPI_ISL_3543614, EPI_ISL_3543616, EPI_ISL_3543629, EPI_ISL_3543635, EPI_ISL_3543638	National Center of Disease Control and Prevention of the Republic of Armenia, Davidyants Laboratories, Yerevan, Armenia	Institute of Molecular Biology NAS RA, Republic of Armenia, Department of Bioengineering, BioinformaticsInstitute and Molecular Biology IBMPh RAU, Republic of Armenia	Andranik Chavushyan; Ani Melkonyan; Arsen Arakelyan; Diana Avetyan; Gayane Melik-Pashayan; Gisane Khachatyan; Hovsep Ghazaryan; Lilit Ghukasyan; Maria Nikoghosyan; Nelli Muradyan; Roksana Zakharyan; Shushan Sargrsyan; Siras Hakobyan; Tamara Sirunyan
EPI_ISL_1301994, EPI_ISL_2081910, EPI_ISL_2180157, EPI_ISL_2621388, EPI_ISL_2621411, EPI_ISL_2621413, EPI_ISL_2648093, EPI_ISL_2772530, EPI_ISL_2841895, EPI_ISL_2841915, EPI_ISL_2841970, EPI_ISL_3076736, EPI_ISL_3076750, EPI_ISL_3543052, EPI_ISL_3796095, EPI_ISL_3796647, EPI_ISL_3796723, EPI_ISL_3796741			
see above	National Center of Infectious and Parasitic Diseases	National Center of Infectious and Parasitic Diseases	Alexiev; Alexiev et al; Dimitrova; Dobrinov; Donchev; Grigorova I.; Grigorova L.; Hristova; Ivanov; Kantardjiev; Korsun; Philipova; Stoitsova; Stoykov; Trifonova
EPI_ISL_2897683	National Centre for Cell Science	CDFD	Ashwin Dalal; Asmita Gupta; Divya Vashisht; Murali Bashyam; Reelina Basu; Vinay Donipadi
EPI_ISL_2106789, EPI_ISL_2106934	National Centre for Disease Control	CDFD-INSACOG	Ashwin Dalal; Asmita Gupta; Divya Vashisht; Murali Bashyam; Pratyusha Bala; Vinay Donipadi
EPI_ISL_466850, EPI_ISL_528616	National Genomics Core-Center for DNA Fingerprinting and Diagnostics	National Genomics Core- Center for DNA Fingerprinting and Diagnostics (NGC-CDFD)- DBT's PAN-INDIA-1000 Genome consortium	ASHWIN DALAL; Amrita Bhattacherjee; Arjita Jaiswal; Arpita Singh; Ashwin Dalal; Bala Pratyusha; DEBASHIS MITRA; DIVYA VASHISHT; Debashish Mitra; Devanshi Gupta; Divya Vashisht; G Shashikanth; Heena Shah; J. Mallikarjun; K. Viswakalyan; K.Manohar; Kaisar Ahmad Lone; Kausika Kumar Malik; MURALI DHARAN BASHYAM; Madhumohan Rao; Murali Dharan Bashyam; N. Sudheer; Neeraj Kumar; R HARINARAYANAN; R Harinarayanan; RASHNA BHANDARI; Rashna Bhandari; Romila Moirangthem; SPR Prasad; Sanjana Sarkar; Shivani Yadav; Vinay Donipadi; Yogesh Patidar
EPI_ISL_3663497, EPI_ISL_3730332, EPI_ISL_3730344, EPI_ISL_3730377, EPI_ISL_3730379	National HIV Reference Laboratory, Ministry of Health, Public Health Institute of Malawi	CERI, Centre for Epidemic Response and Innvoation, Stellenbosch University and KRISP, KZN Research Innovation and Sequencing Platform, UKZN.	Auld A; Chilima B; Chiwaula M; Emmanuel SJ; Giandhari J; Kaba M; Kampira E; Kasambara W; Kim L; Lessells R; Maida A; Mvula B; Mwangomba W; Naidoo Y; Panja L; Pillay S; Tegally H; Tshiabuila Derek; Wadonda N; Wilkinson E; de Oliveira T
EPI_ISL_1407115, EPI_ISL_2494782, EPI_ISL_2494830, EPI_ISL_2494924, EPI_ISL_2494966, EPI_ISL_2494972, EPI_ISL_2609595			
see above	National HIV Reference Laboratory, Ministry of Health, Public Health Institute of Malawi	KRISP, KZN Research Innovation and Sequencing Platform	Auld A; Chilima B; Chiwaula M; Emmanuel SJ; Giandhari J; Kaba M; Kampira E; Kasambara W; Kim L; Lessells R; Maida A; Mvula B; Mwangomba W; Naidoo Y; Panja L; Pillay S; Tegally H; Wadonda N; Wilkinson E; de Oliveira T
EPI_ISL_3663621, EPI_ISL_3663623, EPI_ISL_3663631, EPI_ISL_3663632, EPI_ISL_3663642, EPI_ISL_3663661, EPI_ISL_3663671			
see above	National HIV Reference Laboratory, Ministry of Health, Public Health Institute of Malawi	KRISP, KZn Research Innovation and Sequencing Platform	Auld A; Chilima B; Chiwaula M; Emmanuel SJ; Giandhari J; Kaba M; Kampira E; Kasambara W; Kim L; Lessells R; Maida A; Mvula B; Mwangomba W; Naidoo Y; Panja L; Pillay S; Tegally H; Tshiabuila Derek; Wadonda N; Wilkinson E; de Oliveira T
EPI_ISL_1677732	National Health Laboratory	Botswana Institute for Technology Research and Innovation	Dineo Emang Tshiamo. Tefelo Thela; Gape Nyepetsi; Kefentse Arnold Tumedi; Madisa Mine; Maitshwarelo Ignatius Matsheka; Malebogo Kebabonye; Thongbotho Mphoyakgosi
EPI_ISL_3799050	National Health Laboratory Service, South Africa	KRISP, KZn Research Innovation and Sequencing Platform	Emmanuel S; Giandhari J; Naidoo Yeshnee; Pillay S; Tegally H; Tshabuila Derek; Wilkinson E; Yajna Ramphal; de Oliveira T
EPI_ISL_456600	National Health Laboratory, Timor-Leste	Microbiological Diagnostic Unit Public Health Laboratory, The Peter Doherty Institute for Infection and Immunity	Antonia da Costa, E.; Baird, R.; Barreto, I.; Caly, L.; Canisia, D.; Dakh, F.; Dolores de Jesus da Costa, M.; Douglas, N.; Francis, J.; Freeman, K.; Jayanti Pereira Tilman, A.; Marr, I.; Sait, M.; Salles de Sousa, A.; Schultz, M.; Seemann, T.; Sherry, N.; Soares da Silva, E.; Wapling, J.; Ximenes, J.
EPI_ISL_2161032	National Hematology and Transfusiology Center	National Hematology and Transfusiology Center	Aghayev AR
EPI_ISL_2455484, EPI_ISL_2455498, EPI_ISL_2455504, EPI_ISL_2455506, EPI_ISL_2458057	National Hospital for Tropical Diseases	Oxford University Clinical Research Unit, Hanoi, Vietnam	H.Rogier van Doorn on behalf of the OUCRU COVID-19 research group; Le Van Duyet; Nguyen Thi Hong Thuong; Nguyen Thi Kim Chi; Nguyen Thi Tam; Nguyen Thu Trang; Pham Ngoc Thach; Phan Manh Cuong; Thomas Kesteman; Van Dinh Trang
EPI_ISL_959282, EPI_ISL_959283	National Influenza Center, Virology Department	National Influenza Center	A Nejati; F Ajaminejad; J Yavarian; K Sadeghi; N Ghavvami and T Mokhtari Azad; NZ Shafiei Jandaghi; V Salimi

EPI_ISL_2285860, EPI_ISL_2535792, EPI_ISL_2873850	National Influenza Centre	National Influenza Centre	; Benjamiin B. Lindsey; Benjamin H. Foulkes; Dennis Laryea; Ernest Asiedu; Franklin Asiedu-Bekoe; Gordon Awandare; Ivy A. Asante; Joseph Oliver-Commey; Joyce Ngoi; Linda Boatemaa; Lorreta Kwasah; Mathew D. Parker; Michael Marks; Mildred Adusei-Poku; Sharon Hsu; Thushan I de Silva; William K. Ampofo
EPI_ISL_402125	National Institute for Communicable Disease Control and Prevention (ICDC) Chinese Center for Disease Control and Prevention (China CDC)	National Institute for Communicable Disease Control and Prevention (ICDC) Chinese Center for Disease Control and Prevention (China CDC)	Chen; Dai; F.-H.; Hu, Y.; J.-H.; J.-J.; J.-L. and Zhu; Liu, Y.; Pei; Q.-M.; She; Song; T.-Y.; Tao; Tian; Wang; Wang, W.; Wu, F.; Xu, L.; Y.-L.; Y.-M.; Y.-Y.; Y.-Z.; Yu, B.; Z.-G.; Z.-W.; Zhang; Zhao, S.; Zheng
EPI_ISL_3644034, EPI_ISL_3644065, EPI_ISL_3718064	National Institute for Communicable Diseases of the National Health Laboratory Service	National Institute for Communicable Diseases of the National Health Laboratory Service	Amoako DG; Bhiman JN; Everatt J; Ismail A; Mahlangu B; Mnguni A; Mohale T; Ntuli N; Scheepers C
EPI_ISL_469254	National Institute for Viral Disease Control and Prevention, China CDC	Institute of Viral Disease Control and Prevention, China CDC	Chun Huang; Dayan Wang; George Fu Gao; Guizhen Wu; Li Zhao; Lijuan Chen; Peihua Niu , Baoying Huang; Roujian Lu; Wenbo Xu; Wenjie Tan; Wenling Wang; Yubai Bi
EPI_ISL_591275, EPI_ISL_2170893	National Institute for Viral Disease Control and Prevention, China CDC	National Institute for Viral Disease Control and Prevention, China CDC	; 10; 4&; 7; Baoying Huang3; Cao Chen; Cao Chen3&; Dayan Wang; Dayan Wang3; Dongyan Wang3; Fengqin Li6; George F.Gao; George Fu Gao1; Haibo Sun5; Hong Wang; Hong Wang3; Huilai Ma; Huilai Ma1&; Ji Wang; Ji Wang3&; Jian Cai1; Jianqun Zhang2&; Jianxing Yu1; Jingdong Song; Jun Han; Jun Meng2; Li Bai6; Li Zhao3; Liang Wang1; Lingling Mao5; Mingchun Luan2; Naiying Mao3; Ning Li6; Peihua Niu3; Qian Yang3; Ruqin Gao; Shaofeng Jiang9; Shihong Yang2; Shuangli Zhu3; Tao Ma1; Tianjiao Ji3; Wei Yao2*; Wenbo Xu; Wenbo Xu3*; Wenjie Tan3; Wenqing Yao5*; Xiang Ren1; Xiang Zhao; Xiang Zhao3; Yan Zhang3; Yang Song3&; Yanhai Wang3; Yao Meng; Yecheng Yao11; Ying Qin1&; Yingwei Sun5; Yong Zhang; Yong Zhang3; Yuchao Wu; Yunting Xia8; Zhaoguo Wang; Zhen Zhu3; Zhijian Bo2; Zhixiao Chen; Zhongjie Li1; Zijian Feng1*
EPI_ISL_1447484	National Institute of Infectious Diseases (NIID)	National Institute of Infectious Diseases (NIID)	Hideka Miura; Kentaro Itokawa; Kiyoko Okamoto; Kumiko Araki; Makoto Kuroda; Masanori Hashino; Rina Tanaka; Seiichiro Fujisaki; Shinichiro Hirai; Tsuyoshi Sekizuka
EPI_ISL_979803, EPI_ISL_1279947, EPI_ISL_2308250, EPI_ISL_3259564	National Institute of Infectious Diseases-Prof. Dr. Matei Bals Molecular Diagnostics Laboratory	National Institute of Infectious Diseases-Prof. Dr. Matei Bals Molecular Diagnostics Laboratory	Andreea Tudor; Corina Casangiu; Dan Otelea; Leontina Banica; Marius Surleac; Ovidiu Vlaicu; Petre Milu; Robert Hohan; Simona Paraschiv
EPI_ISL_466636, EPI_ISL_466638, EPI_ISL_475083, EPI_ISL_1531554, EPI_ISL_2803761	National Institute of Laboratory Medicine and Referral Center	Genomic Research Lab, BCSIR	A. K. M. Shamsuzzaman; Abu Sayeed Mohammad Mahmud; Arifa Akram; Asish Kumar Ghosh; Barna Goswami; Bayzid Bin Monir; Eshrar Osman; Iffat Jahan; Mahmuda Yeasmin; Md. Ahasan Habib; Md. Kamrul Islam; Md. Maruf Ahmed Molla; Md. Murshed Hasan Sarkar; Md. Murshed Hasan Sarker; Md. Saddam Hossain; Md. Salim Khan; Mohammad Mohi Uddin; Mohammad Samir Uzzaman; Salek Ahmed Sajib; Shahina Akter; Sheikh Md. Selim Al Din; Tanjina Akhter Banu; Tanjina Akhter Banu; Tasnim Nafisa; Utpal Chandra Ray
EPI_ISL_2323219	National Institute of Public Health	Charles University, Faculty of Science, BIOCEV, OMICS Genomics	Blanka Hamplová; Ingrid Poláková; Jana Šmahelová; Jiří Novák; Magdalena Jančářová; Ruth Tachezy; Sebastian Cristian Treitli; Vladimír Hampl; Zoltán Füssy; Štěpánka Hrdá
EPI_ISL_2687993	National Institute of Public Health	National Institute of Infectious Diseases-Prof. Dr. Matei Bals Molecular Diagnostics Laboratory	Corina Casangiu; Dan Otelea; Leontina Banica; Marius Surleac; Petre Milu; Robert Hohan; Simona Paraschiv
EPI_ISL_2727050, EPI_ISL_3063179, EPI_ISL_3319026, EPI_ISL_3716755	National Institute of Public Health	National Institute of Public Health	Alexander Nagy; Dusan Trnka; Helena Jirincova; Jaromira Vecerova; Timotej Suri
EPI_ISL_1510302, EPI_ISL_2466503, EPI_ISL_2562083, EPI_ISL_2674248, EPI_ISL_3588364	National Institute of Public Health	State Veterinary Institute Prague	A; Alexander Nagy; Cernikov; D; H; Helena Jirincova; J; Jaromira Vecerova; Jirincova; L; Lenka Cernikova; M; Martina Stara; Nagy; Stara; Suri; T; Timotej Suri; Trnka; Vecerova
EPI_ISL_3032389	National Institute of Public Health NIH - National Research Institute	National Institute of Public Health NIH - National Research Institute	Gierczyńki Rafał; Sadkowska-Todys Małgorzata; Wołkowicz Tomasz; Zacharczuk Katarzyna
EPI_ISL_2928004, EPI_ISL_2928005, EPI_ISL_2928006, EPI_ISL_2928011, EPI_ISL_2928012	National Institute of Public Health, Bujumbura,Burundi, MRC/UVRI & LSHTM Uganda Research Unit	MRC/UVRI & LSHTM Uganda Research Unit, National Institute of Public Health	Alexis Niyomwungere; Anatole Nkeshimana; Dan Lule Bugembe; Deogratius Ssemwanga; Jerome Nkurunziza; Léopold Ouedraogo; Matthew Cotten; My V.T. Phan; Pontiano Kaleebu
EPI_ISL_1056025	National Laboratory for Health, Environment and Food	CISLD (Clinical Institute of Special Laboratory Diagnostics), University Children's Hospital, University Medical Center Ljubljana	Ana Grom; Barbara Jenko Bizjan; Jernej Kovač; Katarina Kozmos; Marko Pokorn; Maruša Debeljak; Robert Šket; Tadej Battelino; Tine Tesovnik
EPI_ISL_3829648	National Laboratory for Health, Environment and Food, OMM, Celje	NLZOH (National Laboratory for Health, Environment and Food) / CISLD (Clinical Institute of Special Laboratory Diagnostics), University Children's Hospital, University Medical Center Ljubljana	Aleksander Kocuvan; Aleksander Mahnic; Alenka Štorman; Ana Grom; Barbara Jenko Bizjan; Daša Kavka / Jernej Kovač; Kaja Tominc; Katarina Kozmos; Maja Rupnik; Marko Pokorn; Maruša Debeljak; Mateja Borinc; Maša Jarčič; Nika Gobec; Robert Šket; Sandra Janezic; Tadej Battelino; Tine Tesovnik; Tjaša Žohar Čretnik
EPI_ISL_3316907	National Laboratory for Health, Environment and Food, OMM, Kranj	NLZOH (National Laboratory for Health, Environment and Food) / CISLD (Clinical Institute of Special Laboratory Diagnostics), University Children's Hospital, University Medical Center Ljubljana	Aleksander Kocuvan; Aleksander Mahnic; Alenka Štorman; Ana Grom; Barbara Jenko Bizjan; Kaja Tominc; Katarina Kozmos; Maja Rupnik; Marjana Petrevčič / Jernej Kovač; Marko Pokorn; Maruša Debeljak; Mateja Ravnik; Maša Jarčič; Monika Korošec; Nika Gobec; Robert Šket; Sandra Janezic; Tadej Battelino; Tine Tesovnik; Tjaša Žohar Čretnik
EPI_ISL_3316557, EPI_ISL_3829151, EPI_ISL_3829415	National Laboratory for Health, Environment and Food, OMM, Maribor	NLZOH (National Laboratory for Health, Environment and Food) / CISLD (Clinical Institute of Special Laboratory Diagnostics), University Children's Hospital, University Medical Center Ljubljana	Aleksander Kocuvan; Aleksander Mahnic; Alenka Štorman; Ana Grom; Andrej Golle / Jernej Kovač; Barbara Jenko Bizjan; Kaja Tominc; Katarina Kozmos; Maja Rupnik; Marko Pokorn; Maruša Debeljak; Maša Jarčič; Mojca Cimerman; Nika Gobec; Nika Volmajer; Robert Šket; Sandra Janezic; Tadej Battelino; Tine Tesovnik; Tjaša Žohar Čretnik
EPI_ISL_1240562	National Laboratory for Health, Environment and Food, OMM, Novo mesto	CISLD (Clinical Institute of Special Laboratory Diagnostics), University Children's Hospital, University Medical Center Ljubljana	Ana Grom; Barbara Jenko Bizjan; Jernej Kovač; Katarina Kozmos; Marko Pokorn; Maruša Debeljak; Robert Šket; Tadej Battelino; Tine Tesovnik
EPI_ISL_512639	National Laboratory for Influenza/Virology reference laboratory, Public Health Center of the Ministry of Health of Ukraine	Respiratory Virus Unit, Microbiology Services Colindale, Public Health England	Dr. Iryna Demchyshyna; PHE Covid Sequencing Team
EPI_ISL_647980, EPI_ISL_1191805, EPI_ISL_1191818, EPI_ISL_1191819, EPI_ISL_1191822, EPI_ISL_1191895	National Microbiology Reference Laboratory	Quadram Institute Bioscience	Agnes Juru; Alexander Goredema; Ana-Victoria Gutierrez; Andrew J. Page; Andrew Tarupiwa; Barbra Murwira; Beuty Makamure; Charles Nyagupe; David Baker; Faustinos T Takawira; Gaetan Thilliez; Gemma Kay; Gibson Mhlanga; Hlanai Gumbo; Isaac Phiri; Justin O'Grady; Kenneth K Maeka; Leonardo de Oliveira Martins; Muchaneta Mugabe; Portia Manangazira; Raiva Simbi; Robert Kingsley; Sekesai Zinyowera; Tapfumanei Mashe; Tatenda Takawira; Thanh Le Viet
EPI_ISL_3722283, EPI_ISL_3722286, EPI_ISL_3722287, EPI_ISL_3722310, EPI_ISL_3730395, EPI_ISL_3730400, EPI_ISL_3730429, EPI_ISL_3730431, EPI_ISL_3730481, EPI_ISL_3730482			
see above	National Microbiology Reference Laboratory, Ministry of Health, Harare, Zimbabwe	CERI, Centre for Epidemic Response and Innvoation, Stellenbosch University and KRISP, KZN Research Innovation and Sequencing Platform, UKZN.	Agnes Juru; Air Comodor Dr J. Chimedza; Charles Nyagupe; Dr Raiva Simbi; Emmanuel SJ; Giandhari J; Hlanai Gumbo; Kenneth Maeka; Naidoo Y; Pillay S; Tapfumanei Mashe; Tatenda Takawira; Tegally H; Wilkinson E; de Oliveira T
EPI_ISL_2483712, EPI_ISL_2756206	National Platform bis UMONS/Jolimont	National Platform bis UMONS/Jolimont	Eric Tarantino; Florian Juszczak; François Dufrasne; Gautier Detry; Guillaume Bayon-Vicente; Ruddy Wattiez
EPI_ISL_3547669, EPI_ISL_3547673, EPI_ISL_3547676, EPI_ISL_3547686, EPI_ISL_3547687, EPI_ISL_3547689, EPI_ISL_3547690, EPI_ISL_3547691, EPI_ISL_3547694, EPI_ISL_3547703			
see above	National Public Health Institute of Liberia Reference Lab	Center for Infection and Immunity, Columbia University	Bode Shobayo; Jane MaCauley; Komal Jain; Mitali Mishra; Nischay Mishra; Thomas Briese; W. Ian Lipkin
EPI_ISL_2479957	National Public Health Laboartory	CSIR-Institute of Genomics and Integrative Biology	Jyoti Acharya; Lokbandhu Chaudhary; Priya Jha; Runa Jha; Suni Dangol
EPI_ISL_3235144	National Public Health Laboratory	Laboratory of Respiratory Viruses and Measles, Oswaldo Cruz Institute, FIOCRUZ	Alice Sampaio Rocha; Ana Carolina Mendonca; Anna Carolina Paixao; Elisa Cavalcante Pereira; Fernando Motta; Luciana Appolinario; Marilda Siqueira on behalf of the Fiocruz COVID-19 Genomic Surveillance Network; Michelle Hamilton; Paola Resende; Renata Serrano Lopes; Taina Venas
EPI_ISL_2674094	National Public Health Laboratory	National Public Health Laboratory/CSIR-Institute of Genomics and Integrative Biology	National Public Health Laboratory Team
EPI_ISL_754073	National Public Health Laboratory	Nepal Health Research Council	Meghnath Dhimal; Pradip Gyanwali
EPI_ISL_845546, EPI_ISL_845551	National Public Health Laboratory, Cameroon	African Centre of Excellence for Genomics of Infectious Diseases (ACEGID), Redeemer's University	Oluniyi P.E. et al
EPI_ISL_2450774, EPI_ISL_2450805	National Public Health Laboratory, Ministry of Health, Ministry of Health, Republic of South Sudan	South Sudan Ministry of Health, WHO South Sudan, MRC/UVRI & LSHTM Uganda Research Unit	Abe G. Abias; Dan Lule Bugembe; Dennis Kenyi Lodiongo; James Ayei; John Rumunu; Joseph Francis Wamala; Juma John HM; Lul Lojok Deng; Matthew Cotten; My V.T. Phan; Pontiano Kaleebu; Richard Lino Loro Lako; Sudhir Bunga
EPI_ISL_410713, EPI_ISL_418994, EPI_ISL_422431, EPI_ISL_493421, EPI_ISL_498602, EPI_ISL_498611, EPI_ISL_536445, EPI_ISL_596473, EPI_ISL_596490, EPI_ISL_645128, EPI_ISL_693331, EPI_ISL_728201, EPI_ISL_804004, EPI_ISL_995295, EPI_ISL_1081928, EPI_ISL_1489726, EPI_ISL_1719873, EPI_ISL_2349711, EPI_ISL_2349737, EPI_ISL_2349858, EPI_ISL_2508865, EPI_ISL_2509054, EPI_ISL_2621931, EPI_ISL_3008996, EPI_ISL_3262082, EPI_ISL_3573015, EPI_ISL_3737383			

see above	National Public Health Laboratory, National Centre for Infectious Diseases	National Public Health Laboratory, National Centre for Infectious Diseases	Chavatte JM; Cui L; Grace Jie Yin Ngan; Grace Ngan; Katherine Ching; Lin Cui; Lin RTP; Mak TM; Octavia S; Raymond Tzer Pin Lin; Royce Ang; Sophie Octavia; Tze Minn Mak; Zhenyang Zhou; Zhou Z
EPI_ISL_3184538	National Public Health Laboratory/COVID-19 Testing Lab Dailekh	National Public Health Laboratory/CSIR-Institute of Genomics and Integrative Biology	National Public Health Laboratory Team /COVID-19 Testing Lab Dailekh Team
EPI_ISL_3184527	National Public Health Laboratory/Dadeldhura, COVID-19 PCR Lab	National Public Health Laboratory/CSIR-Institute of Genomics and Integrative Biology	COVID-19 PCR Lab Team; National Public Health Laboratory Team /Dadeldhura
EPI_ISL_3184524	National Public Health Laboratory/Mahakali, COVID-19 PCR Lab	National Public Health Laboratory/CSIR-Institute of Genomics and Integrative Biology	COVID-19 PCR Lab Team; National Public Health Laboratory Team /Mahakali
EPI_ISL_3184551	National Public Health Laboratory/PPHL-Karnali	National Public Health Laboratory/CSIR-Institute of Genomics and Integrative Biology	National Public Health Laboratory Team /PPHL-Karnali Team
EPI_ISL_2674083	National Public Health Laboratory/Trishuli Hospital COVID 19 PCR Lab	National Public Health Laboratory/CSIR-Institute of Genomics and Integrative Biology	National Public Health Laboratory Team /Trishuli Hospital COVID 19 PCR Lab Team
EPI_ISL_480299	National Reference Laboratory "Influenza and acute respiratory diseases"	NRL-HIV	Ivailo Alexiev; Ivan Ivanov; Ivva Philipova
EPI_ISL_1273392	National Reference Laboratory - Ministry of Health Maseru Lesotho	National Institute for Communicable Diseases of the National Health Laboratory Service	Amoako DG; Banda R; Bhiman JN; Gorova V; Ismail A; Mahlangu B; Mathabo M; Mohale T; Mooko M; Ntuli N; Scheepers C
EPI_ISL_962877, EPI_ISL_3602088, EPI_ISL_3602112, EPI_ISL_3681514, EPI_ISL_3681525	National Virology Reference Laboratory	National Public Health Laboratory, National Centre for Infectious Diseases	Katherine Ching; Lin Cui; Raymond Tzer Pin Lin; Royce Ang; Taib Surita; Tze Minn Mak; Zaini Zainun; Zhenyang Zhou
EPI_ISL_848095	National Virus Reference Laboratory	Irish Coronavirus Sequencing Consortium - National University of Ireland Galway	Grainne Mc Andrew; Kate Reddington; Simone Coughlan
EPI_ISL_501261, EPI_ISL_525385, EPI_ISL_752571, EPI_ISL_1132602, EPI_ISL_1180578, EPI_ISL_1732235, EPI_ISL_2131948, EPI_ISL_2132094, EPI_ISL_2132109, EPI_ISL_2363459, EPI_ISL_2490969, EPI_ISL_2710694, EPI_ISL_2862248, EPI_ISL_2933927, EPI_ISL_3011570, EPI_ISL_3136294, EPI_ISL_3468162, EPI_ISL_3502991, EPI_ISL_3503013, EPI_ISL_3672049, EPI_ISL_3739299			
see above	National Virus Reference Laboratory	National Virus Reference Laboratory	Aditi Chaturvedi; Calum Walsh; Charlene Bennet; Charlene Bennett; Cillian F De Gascun; Daniel Hare; Fiona Crispie; Gabriel Gonzalez; Guerrino Macori; Jonathan Dean; Matthew McCabe; Michael Carr; Paul Cotter; Seamus Fanning; Suzie Coughlan; Zoe Yandle
EPI_ISL_2533834, EPI_ISL_2533850, EPI_ISL_2533870, EPI_ISL_2533882, EPI_ISL_2858619, EPI_ISL_2860830, EPI_ISL_3845304, EPI_ISL_3845322, EPI_ISL_3845338			
see above	Naval Medical Research Unit No. 3	Naval Medical Research Center Biological Defense Research Directorate	Andrea E. Luquette; Andrew J. Bennett; Bishwo N. Adhikari; Catherine E. Arnold; Chaselynn M. Watters; Emily K. Stefanov; Francisco Malagon; Kyle A. Long; Lindsay A. Glang; Logan J. Voegtly; Luis A. Estrella; Michael V. Deschenes; Regina Z. Cer; Robin H. Miller; Stephen M. Eggan; and Kimberly A. Bishop-Lilly
EPI_ISL_3791308	Navavej Hospital	COVID-19 Network Investigations (CONI) Alliance	Angkana Huang; Anthony R. Jones; Arporn Wangwiwatsin; Bhakbhoom Panthan; Chonticha Klungtong; Duangkamon Loesbanluechai; Ekawat Pasomsub; Elizabeth Batty; Insee Sensorn; Janjira Thaipadungpanit; Khajohn Joonlasak; Kingkan Rakmanee; Krittikorn Kumpornsin; Namfon Kotanan; Stefan Fernandez; Thanat Chookajorn; Theerarat Kochakarn; Treewat Watthanachockchai; Wasun Chantratita; Wudtichai Manasatienkij
EPI_ISL_2091003, EPI_ISL_2651135, EPI_ISL_3346532, EPI_ISL_3696628	Nebraska Public Health Laboratory	NPHL COVID-19 Response Team	NPHL COVID-19 Response Team
EPI_ISL_475190	Nebraska Public Health Laboratory	UNMC COVID-19 Response Team	UNMC COVID-19 Response Team
EPI_ISL_3101469	Nemocnice Trebic	University Hospital Brno, CMBG	Jan Svaton; Kristyna Dufkova; Martina Lengerova; Matej Bezdicek
EPI_ISL_754068, EPI_ISL_754070	Nepal Korea Friendship Municipality Hospital	Nepal Health Research Council	Meghnath Dhimal; Pradip Gyanwali
EPI_ISL_3717578	Neuberg Supratech Reference Laboratory, Ahmedabad	Gujarat Biotechnology Research Centre	Arpit Shukla; Bhadreshsinh Gohil; Chaitanya Joshi; Dinesh Kumar; Janvi Raval; Madhvi Joshi; Nimesh Patel; Nitin Savaliya; Nitin Shukla; Ramesh Pandit; Shiva; Sonal Sharma; Twinkle Soni; Zarna Patel
EPI_ISL_1219874, EPI_ISL_1219879, EPI_ISL_1219886, EPI_ISL_1263190, EPI_ISL_3761182	Nevada State Public Health Laboratory	Nevada State Public Health Laboratory	Andrew Gorzalski; Mark Pandori
EPI_ISL_1791135	New Mexico Department of Health Scientific Laboratory	Center for Global Health, University of New Mexico Health Sciences Center	Anastacia Griego; Darrell Dinwiddie; Daryl Domman; Joseph Hicks; Kurt Schwalm; Michael Edwards; Twila Kunde; Valerie Morley
EPI_ISL_831916, EPI_ISL_1186050, EPI_ISL_2858925, EPI_ISL_3812697	New Mexico Department of Health Scientific Laboratory	New Mexico Department of Health Scientific Laboratory	Anastacia Griego-Fisher; D'eldra Malone; Ellie Johnson; Jennifer Benoit; Keila Gutierrez; Linda Salazar; Ratheesh Rajan
EPI_ISL_1061035, EPI_ISL_2107450, EPI_ISL_2107529, EPI_ISL_2107530, EPI_ISL_2107534, EPI_ISL_3072006	New South Wales Health Pathology Royal Prince Alfred Hospital	Microbiology RPAH	Au, J.; Bull, R.; Deveson, I.; Foster, C.; Rawlinson, W.; Ruiz Silva, M.; Van Hal, S.
EPI_ISL_1196009	Nhlangano Health Centre	National Institute for Communicable Diseases of the National Health Laboratory Service	Amoako DG; Bhiman JN; Ismail A; Mahlangu B; Maphalala GP; Mohale T; Ntuli N; Scheepers C
EPI_ISL_516934	Nicolae Testemitanu State University of Medicine and Pharmacy	International Centre for Genetic Engineering and Biotechnology (ICGEB) and ARGO Open Lab Platform for Genome Sequencing	Dal Monego S; Licastro D; Marcello A; Rajasekharan S; Ulinici M
EPI_ISL_1242024	Nigeria Centre for Disease Control (NCDC)	African Centre of Excellence for Genomics of Infectious Diseases (ACEGID), Redeemer's University	Oluniyi P.E. et al
EPI_ISL_455412, EPI_ISL_527884, EPI_ISL_729967, EPI_ISL_729981, EPI_ISL_729989, EPI_ISL_730019	Nigeria Centre for Disease Control (NCDC)	African Centre of Excellence for Genomics of Infectious Diseases (ACEGID), Redeemer's University, Ede, Osun State, Nigeria	Ajogbasile F.V.; Folarin O.A.; Happi C.T.; Ihekweazu C.; Kayode A.; Oguzie J.; Olawoye I.; Olumade T.; Oluniyi P.E.; Oluniyi P.E. et al; Uwanibe J.
EPI_ISL_1093450, EPI_ISL_1235657	Nigerian Centre for Disease Control (NCDC)	African Centre of Excellence for Genomics of Infectious Diseases (ACEGID), Redeemer's University	I.B.; Olawoye; et al; et.al
EPI_ISL_3407826	Nonthaburi Public Health Office	National Institute of Health, Department of Medical Sciences, Ministry of Public Health, Thailand	; Natchaya Khiadsang; Nuttida Thongpramul; Pakorn Piromtong; Pilailuk Okada; Ratana Tacharoenmuang; Siripaporn Phuygun; Sittiporn Parnmen; Sunthareeya Waicharoen; Thanutsapa Thanadachakul; Warawan Wongboot; sirikanda wimol
EPI_ISL_2363972	NordLab Oulu	Expert Microbiology, National Institute for Health and Welfare	Carita Savolainen-Kopra; Erika Lindh; Haider al-Hello; Jani Halkilahti; Kirsi Liitsola; Niina Ikonen; Pekka Ellonen; Päivi Laurila; Sari Hannula; Soile Blomqvist; Teemu Smura
EPI_ISL_848639, EPI_ISL_2339915, EPI_ISL_2661712, EPI_ISL_2858839, EPI_ISL_3062318, EPI_ISL_3370369, EPI_ISL_3370377, EPI_ISL_3758651			
see above	North Dakota Department of Health, Public Health Laboratory	North Dakota Department of Health, Public Health Laboratory	Lisa Wingerter
EPI_ISL_456395, EPI_ISL_579406, EPI_ISL_579407, EPI_ISL_3760067	North Shore Hospital	Institute of Environmental Science and Research (ESR)	Anja Werno; Antje van der Linden; Arlo Upton; Chris Mansell; David Hammer; Dragana Drinkovic; Erasmus Smit; Gary McAuliffe; Hana Sofia Andersson; Hermes Perez; James Ussher; Jill Sherwood; Jing Wang; Joep de Ligt; Josh Freeman; Julia Howard; Juliet Elvy; Lauren Jelly; Mary DeAlmeida; Matt Blakiston; Matt Storey; Matthew Rogers; Max Bloomfield; Michael Addidle; Michelle Balm; Muhammad Faisal; Nikki Freed; Olin Silander; Olivia Stroeven; Rachel Boyle; Sally Roberts; SallyAnn Harbison; Sarah Jefferies; Sharmini Muttaiyah; Susan Morpeth; Susan Taylor; Timothy Blackmore; Vani Sathyendran; Veronica Playle; Virginia Hope; Xiaoyun Ren
EPI_ISL_2126773	Northumbria University / South Tees Hospitals NHS Foundation Trust / North Cumbria Integrated Care NHS Foundation Trust / North Tees and Hartlepool NHS Foundation Trust / Newcastle Hospitals NHS Foundation Trust	COVID-19 Genomics UK (COG-UK) Consortium	Andrew Nelson; Brendan Payne; Clive Graham; Darren L Smith; Debra Padgett; Edward Barton; Emma Swindells; Garren Scott; Gary Black; Gary Eltringham; Giles S Holt; Greg R Young; Jane Greenaway; Jennifer Collins; John Allan; Joshua Loh; Lynn Dover; Matthew Bashton; Mohammad A Tariq; Paul Baker; Sarah Essex; Steve Liggett; Wen C Yew; Yusri Taha
EPI_ISL_2842603	Northwestern Memorial Hospital	Northwestern University - Ozer Lab	Chad J. Achenbach; Chao Qi; Egon A. Ozer; Judd F. Hultquist; Lacy M. Simons; Lawrence J. Jennings; Michael G. Ison; Ramon Lorenzo-Redondo; Taylor J. Dean
EPI_ISL_925853, EPI_ISL_925872, EPI_ISL_925900, EPI_ISL_925903, EPI_ISL_960257, EPI_ISL_960302, EPI_ISL_1301745, EPI_ISL_1301754, EPI_ISL_2362521, EPI_ISL_2521987, EPI_ISL_2521989, EPI_ISL_2521992, EPI_ISL_2521993, EPI_ISL_2521997, EPI_ISL_2811723, EPI_ISL_2828478, EPI_ISL_3012181, EPI_ISL_3012190, EPI_ISL_3012193, EPI_ISL_3012196, EPI_ISL_3012201, EPI_ISL_3012218, EPI_ISL_3012224, EPI_ISL_3012233			
see above	Nucleic Acid Testing, National Reference	GIGA Medical Genomics	Bouchra Boujemla; Corinne Fasquelle; Esperence Umumararungu; Jacob Souopgui; Keith Durkin; Léon Mutesa; Maria Artesi; Marie-Pierre Hayette; Nathalie Renotte; Patrick Tuyisenge; Reuben Sindayiheba; Robert Rutayisire; Sabin Nsanzimana; Swaibu Gatare; Sébastien Bontems;

	Laboratory		Vincent Bours; Yvan Butera
EPI_ISL_525802, EPI_ISL_1122242	OHSU Lab Services Molecular Microbiology Lab	Oregon SARS-CoV-2 Genome Sequencing Center	Alec J. Hirsch; Andrew C. Adey; Benjamin N. Bimber; Brendan L. O'Connell; Brian J. O'Roak; Daniel N. Streblow; Donna Hansel; Guang Fan; Kayla Carter; Qin; Ruth V. Nichols; Sally Grindstaff; William B. Messer; Xuan
EPI_ISL_3395844	OLVZ Aalst	OLVZ Aalst	Astrid Holderbeke
EPI_ISL_1366744	OUCRU	OUCRU	Guy Thwaites; Huynh Trung Trieu; Lam Minh Yen; Le Manh Hung; Le Nguyen Truc Nhu; Le Thi Thu Huong; Le Van Tan; Nghiem My Ngoc; Ngo Ngoc Quang Minh; Nguyen Thanh Dung; Nguyen Thanh Phong; Nguyen Thanh Truong; Nguyen Thi Thu Hong; Nguyen To Anh; Nguyen Tri Dung; Nguyen Van Vinh Chau; Tran Nguyen Hoang Tu; Tran Tan Thanh
EPI_ISL_3214708	Ochsner Health	Bioinfoexperts	Amy Feehan; Ben Lain; Chris Huston; David J. Nolan; Judy Crabtree; Julia-Garcia-Diaz; Lucio Miele; Rebecca Rose; Samual Moot; Susanna L. Lamers; Tessa LaFleur
EPI_ISL_1138855	Office of Diseases Prevention and Control Region 4 Saraburi	COVID-19 Network Investigations (CONI) Alliance	Angkana Huang; Anthony R. Jones; Arporn Wangwiwatsin; Bhakbhoom Panthan; Chonticha Klungtong; Duangkamon Loesbanluechai; Ekawat Pasomsub; Elizabeth Batty; Insee Sensorn; Janjira Thaipadungpanit; Jutikul Kaewmalakul; Khajohn Joonlasak; Kingkan Rakmanee; Krittikorn Kumpornsin; Namfon Kotanan; Nathamon Runnachot; Pakjira Rimdusit; Payon Pengyo; Praima Moonmuang; Sataporn Hatsadichart; Sirinapa Singthong; Siriporn Lakesukthom; Siriwan Yaemnimnual; Stefan Fernandez; Suttiruk Changchawai; Thanat Chookajorn; Theerarat Kochakarn; Treewat Watthanachockchai; Wasun Chantratita; Wonvimol Lemprasert; Wudtichai Manasatienkij
EPI_ISL_2544511	Ohio Department of Health Laboratory	Ohio Department of Health Laboratory	Allison Black; Brent Lee; Caitlin McDonnell; Eric Brandt; Erica Leasure; Glen McGillivary; Heather Blankenship; Holmes; Jade Mowery; Jennifer; Kelsey Florek; Keoni Omura; Kirtana Ramadugu; Quanta Brown; Stephanie Mccracken; Tyler Payne; and Tammy Bannerman
EPI_ISL_492017	Oman-NIC	Department of Microbiology and Immunology-SQUH	Abdulla Balkhair; Ahlam Al-Amri; Aisha Al-Amri; Aisha Al-Busaidi; Amina Al Jardani; Fahad Zadjali; Fatma BaAlawi; Hamida AL Barwani; Hanan Al-kindi; Intisar Al-Shukri; Khulood Al-Mammary; Mohammed Al-Tobi; Samiha Al Kharusi; Samira Al-Maruqi; Zeyana AL-Dahmani
EPI_ISL_457701, EPI_ISL_457706, EPI_ISL_457977, EPI_ISL_457998	Oman-NIC	Oman-NIC	Abdulla Balkhair; Ahlam Al-Amri; Aisha Al-Amri; Aisha Al-Busaidi; Amina Al Jardani; Fahad Zadjali; Fatma BaAlawi; Hamida AL Barwani; Hanan Al-kindi; Intisar Al-Shukri; Khulood Al-Mammary; Mohammed Al-Tobi; Samiha Al Kharusi; Samira Al-Maruqi; Zeyana AL-Dahmani
EPI_ISL_2921173, EPI_ISL_2921187, EPI_ISL_2921200, EPI_ISL_2921201, EPI_ISL_2925607, EPI_ISL_2925617, EPI_ISL_2925619			
see above	Oman-NIC	Oman-National Influenza Center	Aisha Al-Busaidi; Amina Al Jardani; Bilal Hussain.; Fahad Al Balushi; Hanan Al Kindi; Intisar Al-Shukri; Intisar Al-Shukri.; Laila Al Balushi; Samiha Al Kharusi
EPI_ISL_1532300	Oman-National Influenza Center	Biotechnology & OMICs Laboratory	Abdul Latif Khan; Ahmed Al Harrasi.; Ahmed Al-Rawahi; Ahmed N Al-Rawahi; Aisha Al-Amri; Amal Al-Maani; Amina Al-Jardani; Bilal Hussain; Hanan Al-Kindi; Intisar Al-Shukri; Sajjad Asaf; Samira Al-Mahruqi; Samiya Al-Zadjali; Saqib Bilal
EPI_ISL_766569	Oman-National Influenza Center	Oman-National Influenza Center	Aisha Al-Busaidi; Amina Al-Jardani; Hamida Al-Barwani; Hanan Al-Kindi; Intisar Al-Shukri; Laila Al-Balushi; Samiha Al-Kharusi; Samira Al-Mahruqi
EPI_ISL_1443674, EPI_ISL_3088307	Omics Sciences Laboratory	Omics Sciences Laboratory	Darlyn Amaya; Derly Andrade Molina; Emily Sulay Saltos Montalvo; Gabriel Morey León; Juan Carlos Fernández Cadena; Katheryn Sacheri Viteri; Paula Juliana Gavilanes Jarrín; Rubén Armas González
EPI_ISL_2713063, EPI_ISL_2933947, EPI_ISL_3370931, EPI_ISL_3806959	Oregon State Public Health Laboratory	Oregon State Public Health Laboratory	Eugene Yeboah; John Fontana and Shane Sevey; Laura Tsaknaridis; Rafia Razzaque; Vanda Makris
EPI_ISL_1040028	Original detection - Virology Unit, Institut Pasteur du Cambodge; Sequencing - US National Institute of Allergy and Infectious Diseases Cambodia	Virology Unit, Institut Pasteur du Cambodge	Chau Darapheak; Chin Savuth; Erik A Karlsson; Jennifer Bohl; Jessica Manning; Kraing Sidonn; Ly Sovann; Sophana Chea; Sreyngim Lay; Veasna Duong; Yi Sengdoeurn
EPI_ISL_1979567	Originating lab: Wales Specialist Virology Centre Sequencing lab: Pathogen Genomics Unit	Public Health Wales Microbiology Cardiff Wales Specialist Virology Centre	Alec Birchley; Alexander Adams; Amy Gaskin; Angela Marchbank; Bree Gatica-Wilcox; Catherine Moore; Jason Coombes; Joanne Watkins; Joel Southgate; Johnathan Evans; Laura Gifford; Lauren Gilbert; Lee Graham; Malorie Perry; Matthew Bull; Nicole Pacchiarini; Sally Corden; Sara Kumziene-Summerhayes; Sara Rey; Sarah Taylor; Simon Cottrell; Sophie Jones; Tom Connor
EPI_ISL_417488, EPI_ISL_417489, EPI_ISL_2333106	Oslo University Hospital, Department of Medical Microbiology	Norwegian Institute of Public Health, Department of Virology	Atiya R Ali; Debech Nadia; Engebretsen Serina Beate; Garcia Llorente Ignacio; Hilde Elshaug; Hilde Vollan; Jon Bråte; Kamilla Heddeland Instefjord; Karoline Bragstad; Kathrine Stene-Johansen; Line Victoria Moen; Marie Paulsen Madsen; Olav Hungnes; Pedersen Benedikte Nevjen; Rasmus Riis Kopperud
EPI_ISL_3014036	Osp. S.Pertini	National Institute for Infectious Diseases (INMI) L. Spallanzani I.R.C.C.S	A Di Caro; B Bartolini; CEM Gruber; E Giombini; F Messina; F Santini; G Bonfiglio; M Rueca; MR Capobianchi; O Butera
EPI_ISL_3846616	Ospedale Santa Caterina Novella	Istituto Zooprofilattico Sperimentale della Puglia e della Basilicata	Bianco A.; Bruno A. R.; Capozzi L.; Del Sambro L.; Difato L.; Parisi A.; Simone D.
EPI_ISL_590992	Ostfold Hospital Trust - Kalnes, Centre for Laboratory Medicine, Section for gene technology and infection serology	Norwegian Institute of Public Health, Department of Virology	Hilde Elshaug; Hilde Vollan; Kamilla Heddeland Instefjord; Karoline Bragstad; Kathrine Stene-Johansen; Olav Hungnes; Rasmus Riis Kopperud
EPI_ISL_3355747	Outre mer	National Reference Center for Viruses of Respiratory Infections, Institut Pasteur, Paris	Angela Brisebarre; Antoine Talarmin; Camille Capel; Christophe Malabat; Corinne Maufrais; Etienne Simon-Lorière; Frédéric Lemoine; Hub de Bioinformatique et Biostatistique; Louise Lefrançois; Marion Barbet; Maud Vanpeene; Méline Bizard; Sylvie Behillil; Sylvie Van der Werf; Vincent Enouf
EPI_ISL_1293350	Oxford University Clinical Research Unit (OUCRU)	Oxford University Clinical Research Unit (OUCRU)	Guy Thwaites; Huynh Trung Trieu; Lam Minh Yen; Le Manh Hung; Le Nguyen Truc Nhu; Le Thi Thu Huong; Nghiem My Ngoc; Ngo Ngoc Quang Minh; Nguyen Thanh Dung; Nguyen Thanh Phong; Nguyen Thanh Truong; Nguyen Thi Thu Hong; Nguyen To Anh; Nguyen Tri Dung; Nguyen Van Vinh Chau; Tran Nguyen Hoang Tu; Tran Tan Thanh; le Van Tan
EPI_ISL_2695569	POLOKWANE MANKWENG HOSPITAL	National Institute for Communicable Diseases of the National Health Laboratory Service	Amoako DG; Bhiman JN; Everatt J; Ismail A; Mahlangu B; Mnguni A; Mohale T; Ntuli N; Scheepers C
EPI_ISL_2229161, EPI_ISL_2384022, EPI_ISL_3127918	PR Public Health Lab	Centers for Disease Control and Prevention Division of Viral Diseases, Pathogen Discovery	Alex Burgin; Alison Laufer Halpin; Ben L. Rambo-Martin; Clinton R. Paden; Dakota Howard; Darlene Wagner; Dave Wentworth; Dhwani Batra; Jasmine Padilla; Justin Lee; Katie Dillon; Krista Queen; Kristen Knipe; Kristine Lacek; Mark Burroughs; Matthew Schmerer; Meghan Bentz; Mili Sheth; Peter Cook; Sam Shepard; Sarah Nobles; Shoshona Le; Suxiang Tong; Vivien Dugan; Yvette Unoarumhi
EPI_ISL_3459410, EPI_ISL_3459411	PREVIS - IPS	Instituto Nacional de Salud	Carlos Franco-Muñoz; Carmen Osorio; Diana Malo; Diego A. Álvarez-Díaz; Diego Andrés Prada; Gerardo Santamaría; Hector Alejandro Ruiz-Moreno; Jhonnatan Reales-González; Jorge Rivera; Juan Camilo Martinez; Julian Naizaque; Katherine Laiton-Donato; Lisseth Pardo; Magdalena Wiesner; Marcela Mercado-Reyes; Maria T. Herrera-Sepúlveda; Marta Lopez Blanco; Martha Lucia Ospina Martinez; Paola Rojas; Sergio Gomez; Sheryll Corchuelo; Ángela Alarcon Cruz
EPI_ISL_2621177, EPI_ISL_2621178, EPI_ISL_2621180	PRIME DIAGNOSTICS	Universidad Nacional de Colombia - Laboratorio Genómico One Health	Andres F. Cardona-Rios; Carlos Franco-Muñoz; Carolina Muñoz-Arango; Celeny Ortiz; Daniel O. Maldonado-Perez; Diego A. Álvarez-Díaz; Hector Alejandro Ruiz-Moreno; Idabely Betancur Ortiz; Jorge E. Osorio; Juan P. Hernandez-Ortiz; Karl A Ciuoderis; Katherine Laiton-Donato; Laura Silvana Perez; Lina M. Hurtado; Marcela Mercado-Reyes; Maria Angélica Maya; Maria Stella López; Rita Almanza Payares; Sandra Ines Cano; Simón Villegas Velásquez
EPI_ISL_2345796	PSF MARIA JOSE SALTO DE PIRAPORA	Instituto Butantan / Mendelics	Antonio Jorge Martins; Claudia Renata dos Santos Barros; David Schlesinger; Debora Botequio Moretti; Dimas Tadeu Covas; Elaine Cristina Marqueze; Elaine Vieira Santos; Evandra Strazza Rodrigues; Heidge Fukumasu; Jayme Augusto de Souza-Neto; José Salvatore Leister Patané; Luiz Alcantara; Luiz Lehmann Coutinho; Maria Carolina Elias; Maurício Lacerda Nogueira; Rafael dos Santos Bezerra; Raul Machado Neto; Rejane Maria Tommasini Grotto; Ricardo Haddad; Sandra Coccuzzo Sampaio Vessoni; Simone Kashima; Svetoslav Nanev Slavov; Vincent Louis Viala
EPI_ISL_3076899	PZU "Eurofarm - Centar Poliklinika"	University of Sarajevo, Veterinary Faculty, Laboratory for Molecular Diagnostic and Research Laboratory	Goletic S; Goletic T; Hodzic A.; Huseinbegovic E; Jazic A.; Nicevic M.; Prguda-Mujic J; Sabic E.; Softic A; Terzic I.
EPI_ISL_2241616, EPI_ISL_3453879	Palapye Primary Hospital Laboratory	Botswana Harvard HIV Reference Laboratory	Boitumelo J.L Zuze; Boitumelo Zuze; Botshelo Radibe; David Lawrence; Dorcas Maruapula; Joseph Makhema; Keoratile Ntshambiwa; Kwana Lechiile; Legodile Kooepile; Letsibogo Gaoraelwe; Madisa Mine; Modisa Motswaledi; Mosepele Mosepele; Ontlametse T. Bareng; Roger Shapiro; Shahin Lockman; Sikhulile Moyo; Sikhulile Wonderful T. Choga; Simani Gaseitsiwe; Thela Tefelo; Thongbotho Mphoyakgosi; Wonderful T. Choga
EPI_ISL_596505, EPI_ISL_596509, EPI_ISL_596529	Palestinian Ministry of Health	Molecular Genetics Lab	Damien Richard; Dana Najjar; Francois Balloux; Hisham Darwish; Husam Sallam; Issa Shtayeh; Lucy van Dorp; Mahmoud Ruzayqat; Nouar Qutob; Osama Najjar; Zaidoun Salah
EPI_ISL_708185, EPI_ISL_708188	Pamukkale University Hospital	Pamukkale University Department of Medical Genetics	Onur TOKGUN et al.
EPI_ISL_2634931	Pandemic Response Lab - NYC	Pandemic Response Lab, R&D	Cybill del Castillo; Dylan Law; Haiping Hao; Henry Lee; Jon Laurent; Katharine Nelson; Melissa Hopkins; Michael Hammerling; Pradeep Bugga; Shinyoung Clair Kang; Sol Rey; William Ward
EPI_ISL_2802115	Parow	NHLS/UCT	Arash Iranzadeh; Bruna Galvao; Carolyn Williamson; Deelan Doolabh; Diana Hardie; Gert Marais; Innocent Mudau; Lynn Tyers; Marvin Hsiao; Stephen Korsman
EPI_ISL_430845	Pasig City General Hospital	Research Institute for Tropical Medicine	Bautista; Brunker, K.; C.S.; C.T.; D.L.; Demetria; E.S.; F.G.M.; I.A.P.; Manalo; Medado; Mercado; O.J.T.; Onza; Polotan
EPI_ISL_2035941	Pasteur Institute - Laboratory of Clinical Virology	Pasteur Institute - Laboratory of Clinical Virology	Anissa Chouikha; Henda Triki; Kais Ghedira; Mariem Gdoura; Sondos Haddad; Wasfi Fares
EPI_ISL_456163, EPI_ISL_456194, EPI_ISL_456301, EPI_ISL_582019, EPI_ISL_622806, EPI_ISL_755626, EPI_ISL_3477099, EPI_ISL_3477100, EPI_ISL_3477101, EPI_ISL_3477102, EPI_ISL_3477103, EPI_ISL_3477104, EPI_ISL_3477105			
see above	PathLab Bay of Plenty	Institute of Environmental Science and Research (ESR)	Anja Werno; Antje van der Linden; Arlo Upton; Chris Mansell; David Hammer; Dragana Drinkovic; Erasmus Smit; Gary McAuliffe; Hana Sofia Andersson; Hermes Perez; James Ussher; Jill Sherwood; Jing Wang; Joep de Ligt; Josh Freeman; Julia Howard; Juliet Elvy; Lauren Jelly; Mary DeAlmeida; Matt Blakiston; Matt Storey; Matthew Rogers; Max Bloomfield; Michael Addidle; Michelle Balm; Muhammad Faisal; Nikki Freed; Olin Silander; Olivia Stroeven; Rachel Boyle; Sally Roberts; SallyAnn Harbison; Sarah Jefferies; Sharmini Muttaiyah; Susan Morpeth; Susan Taylor; Timothy Blackmore; Vani Sathyendran; Veronica Playle; Virginia Hope; Xiaoyun Ren
EPI_ISL_470851, EPI_ISL_2662451, EPI_ISL_2713075, EPI_ISL_2932263	PathWest Laboratory Medicine WA	PathWest Laboratory Medicine WA	Avram Levy; Chisha Sikazwe; David Smith and David Speers; Jurissa Lang; PathWest Laboratory Medicine WA; PathWest Laboratory Medicine WA Microbial Surveillance Unit
EPI_ISL_512720, EPI_ISL_512756, EPI_ISL_596717, EPI_ISL_596770, EPI_ISL_605852, EPI_ISL_672626, EPI_ISL_672637, EPI_ISL_708789, EPI_ISL_708796, EPI_ISL_794685, EPI_ISL_933799, EPI_ISL_1069392, EPI_ISL_1416321, EPI_ISL_1816920, EPI_ISL_1828699, EPI_ISL_1828700, EPI_ISL_1914666, EPI_ISL_1914668, EPI_ISL_1972908, EPI_ISL_1972909, EPI_ISL_1972912, EPI_ISL_2081529, EPI_ISL_2098723, EPI_ISL_2598297, EPI_ISL_2757655, EPI_ISL_2757656, EPI_ISL_2757657, EPI_ISL_2784344, EPI_ISL_2861270, EPI_ISL_2982303, EPI_ISL_3038898, EPI_ISL_3038899, EPI_ISL_3150198, EPI_ISL_3150199, EPI_ISL_3245746, EPI_ISL_3245750, EPI_ISL_3245752, EPI_ISL_3693292, EPI_ISL_3693297, EPI_ISL_3693300, EPI_ISL_3693302, EPI_ISL_3693306, EPI_ISL_3693308, EPI_ISL_3841993, EPI_ISL_3841994			
see above	PathWest Laboratory Medicine WA	PathWest Laboratory Medicine WA Microbial Surveillance Unit	PathWest Laboratory Medicine WA Microbial Surveillance Unit
EPI_ISL_2691707	PathWest Laboratory Medicine WA Microbial Surveillance Unit	PathWest Laboratory Medicine WA Microbial Surveillance Unit	Hospital Ave; Nedlands WA 6009; QEII Medical Centre
EPI_ISL_591469, EPI_ISL_685295, EPI_ISL_686002, EPI_ISL_687301, EPI_ISL_691796, EPI_ISL_897331, EPI_ISL_898967, EPI_ISL_1131201, EPI_ISL_1426234, EPI_ISL_1429814, EPI_ISL_1430699, EPI_ISL_2337437, EPI_ISL_3192933, EPI_ISL_3193677, EPI_ISL_3194200			
see above	Pathogen Genomics Center, National Institute	Pathogen Genomics Center, National Institute of	Kentaro Itokawa; Makoto Kuroda; Masanori Hashino; Rina Tanaka; Tsuyoshi Sekizuka

	of Infectious Diseases	Infectious Diseases	
EPI_ISL_512920, EPI_ISL_512922, EPI_ISL_513055	Pathogen Genomics Lab King Abdullah University of Science and Technology(KAUST)	Pathogen Genomics Lab King Abdullah University of Science and Technology(KAUST)	Abdulaziz Alahmadi; Afrah Alsomali; Ahmad Bakur Mahmoud; Amanda; Amanda Ooi; Amit Kumar Subudhi; Anwar Hashem; Arnab Pain; Asim Khogeer; Fadwa Alofi; Fathia Ben Rached; Jumana Taha; Kahled Algithami; Luke; Luke Esau; Naif Almontashiri; Raeece Naeem; Rahul P Salunke; Sara Mfarrej; Sharif Hala
EPI_ISL_407896, EPI_ISL_410717	Pathology Queensland	Public Health Virology Laboratory	Alyssa Pyke; Amanda De Jong; Andrew Van Den Hurk; Ben Huang; Carmel Taylor; David Warrilow; Doris Genge; Elisabeth Gamez; Glen Hewitson; Ian Maxwell Mackay; Inga Sultana; Jamie McMahon; Jean Barcelon; Judy Northill; Mitchell Finger; Natalie Simpson; Neelima Nair; Peter Burtonclay; Peter Moore; Sarah Wheatley; Sean Moody; Sonja Hall-Mendelin; Timothy Gardam; and Frederick Moore.
EPI_ISL_455061, EPI_ISL_593673	Pathology West - NSW Health Pathology	NSW Health Pathology - Institute of Clinical Pathology and Medical Research; Westmead Hospital; University of Sydney	CIDM-PH et al.
EPI_ISL_2433646	Pattani Hoapital	Division of Genomic Medicine and Innovation support,Department of Medical Sciences, Ministry of Public Health, Thailand	Archawin Rojanawiwat; Jirapha Pakdee; Natthakul Bunneang; Nuanjun Wichukchinda; Penpitcha Thawong; Pilailuk Akkapaiboon Okada; Pundharika Piboonsiri; Surakameth Mahasirimongkol; Waritta Sawaengdee
EPI_ISL_3454057	Perm Krai Blood Transfusion Station	WHO National Influenza Centre Russian Federation	Andrey Komissarov; Artem Fadeev; Daria Danilenko; Dmitry Lioznov; Elena Nabieva; Georgii Bazykin; Kirill Varchenko; Ksenia Safina; Kseniya Komissarova; Maria Pisareva; Mikhail Bakaev; Nikita Yolshin; Oula Mansour; Tamila Musaeva; Veronika Eder
EPI_ISL_853876	Pharmgenetix GmbH	Bergthaler laboratory, CeMM Research Center for Molecular Medicine of the Austrian Academy of Sciences	Alexander Lercher; Alexandra Popa; Andreas Bergthaler; Anna Schedl; Benedikt Agerer; Christoph Bock; Jakob-Wendelin Genger; Jan Laine; Lukas Endler; Martin Senekowitsch; Michael Schuster; Thomas Penz
EPI_ISL_2156159	Philippine Airport Diagnostic Laboratory	Philippine Genome Center	Alethea R. de Guzman; Anna Ong-Lim; Arianne A. Zamora; Asia Louisa U. Chong; Benedict A. Maralit; Candice Francheska B. Tambaoan; Carlo M. Lapid; Celia Carlos; Devon Ray Pacial; Edsel Maurice Salvaña; El King D. Morado; Eva Maria Cutiongco-de la Paz; Francis A. Tablizo; Irish Coleen A. Asin; Jaime C. Montoya; Jan Michael C. Yap; Jo-Hannah S. Llames; John Q. Wong; Joshua Gregor A. Dizon; Juan Antonio R. Magalang; Karol Sophia Agape R. Padilla; Kenneth M. Kim; Kris P. Punayan; Marc Edsel C. Ayes; Marc Jerrone R. Castro; Maria Rosario Singh-Vergeire and Cynthia P. Saloma; Maria Sofia L. Yangzon; Marissa Alejandria; Razel Nikka M. Hao; Rianna Patricia S. Cruz; Sheila Mae M. Araiza
EPI_ISL_2859170	Philippine General Hospital (PGH)	Philippine Genome Center	Alethea R. de Guzman; Anna Ong-Lim; Arianne A. Zamora; Benedict A. Maralit; Carlo M. Lapid; Celia Carlos; Devon Ray Pacial; Diomedes A. Carino; Edsel Maurice Salvaña; El King D. Morado; Elcid Aaron R. Pangilinan; Eva Maria Cutiongco-de la Paz; Francis A. Tablizo; Henrietta Marie Rodriguez; Jaime C. Montoya; Jan Michael C. Yap; Jarvin E. Nipales; Jo-Hannah S. Llames; John Q. Wong; Joshua Gregor A. Dizon; Juan Antonio R. Magalang; Karol Sophia Agape R. Padilla; Kenneth M. Kim; Kris P. Punayan; Krisitna Patriz Dela Cruz; Lindsay Claire D.L. Carandang; Ma. Exanil Plantig; Marc Edsel C. Ayes; Maria Rosario Singh-Vergeire and Cynthia P. Saloma; Maria Sofia L. Yangzon; Marielle M Gamboa; Marissa Alejandria; Nina Francesca Bustamante; Razel Nikka M. Hao; Renato Jacinto Q. Mantaring; Rianna Patricia S. Cruz; Sheila Mae M. Araiza; Yvonne Valerie Austria; Zipporah Mariebelle R. Enriquez; Zyrel V. Mollejon
EPI_ISL_792022	Plateforme COVID IDF	National Reference Center for Viruses of Respiratory Infections, Institut Pasteur, Paris	Angela Brisebarre; Camille Capel; Etienne Simon-Lorière; Jacques Fourgeaud; Marion Barbet; Maud Vanpeene; Méline Bizard; Sylvie Behillil; Sylvie van der Werf; Vincent Enouf
EPI_ISL_2709213	Poliklinika Muminovic	Alea Genetic Centre	Dino Pecar; Ivana Ceko; Lana Salihefendic; Naida Mulahusejinovic; Rijad Konjhodzic; Selma Durgut
EPI_ISL_2178953	Praava Health	Child Health Research Foundation	CHRF Bangladesh Genomics Team; Shafiul Azam; Zaheed Husain
EPI_ISL_2265206	Praxisgemeinschaft für Laboratoriumsmedizin Labor Blumenstraße; Praxis Dr. E. Wietschel & Dr. F. Wietschel	Robert Koch Institute	
EPI_ISL_2790397	Primary Health Care Banja Luka	Public Health Institute of Republic of Srpska	Branka Culibrk; Dijana Vukajlovic; Milica Celic; Pava Dimitrijevic; Stanka Tomic; Tatjana Markovic; Zeljka Sumic
EPI_ISL_1477046, EPI_ISL_3668635, EPI_ISL_3673671	Private clinic of Biogen Med, Tashkent, Uzbekistan	Center of Genomics and bioinformatics, Bioinformatics laboratory	Abdurakhmon N Yusupov; Dilshod E Usmanov; Ibrokhim Y Abdurakhmonov; Ibrokhim Y Abdurakhmonov.; Khurshida A Ubaydullaeva; Mirzakamol S Ayubov; Mukhammadjon H Mirzakhmedov; Shukhrat E Shermatov; Zabardast T Buriev
EPI_ISL_1469602	Pronto Atendimento Campo Bom	Epiclin	Ana Paula Muterle; Carolina Comerlato; Eliana Márcia Da Ros Wendland; Fernando Hayashi Sant'Anna; Janira Prichula; Juliana Comerlato
EPI_ISL_1017197	Providence Alaska Medical Center	Providence St. Joseph Health Molecular Genomics Laboratory	Alexa K Dowdell; Brian D Piening; Carlo B Bifulco; Fred L Robinson; Mary Campbell
EPI_ISL_1968628	Provincial Public Health Reference Laboratory	Provincial Public Health Reference Laboratory	ANDLEEB HANIF
EPI_ISL_1180691	Public Health Authority of the Slovak Republic	Bergthaler laboratory, CeMM Research Center for Molecular Medicine of the Austrian Academy of Sciences	Andreas Bergthaler; Anna Schedl; Bekir Erguner; Benedikt Agerer; Christoph Bock; Fabian Amman; Jan Laine; Lukas Endler; Maelle Le Moing; Martin Senekowitsch; Michael Schuster; Thomas Penz
EPI_ISL_2497478, EPI_ISL_2657892, EPI_ISL_2688092, EPI_ISL_2844572, EPI_ISL_3117967, EPI_ISL_3229349, EPI_ISL_3229374, EPI_ISL_3452619			
see above	Public Health Authority of the Slovak Republic	Laboratory of Genomics and Bioinformatics, Comenius University Science Park	Anna Gičová; Diana Rusňáková; Jakub Styk; Jaroslav Budiš; Miroslav Böhmer; Tatiana Sedláčková; Tomáš Szemes
EPI_ISL_2965748	Public Health Authority of the Slovak Republic	Public Health Authority of the Slovak Republic	Anna Gičová; Barbora Kotvasová; Elena Tichá; Lucia Ševčíková; Miroslav Böhmer; Pavol Mišenko; Terézia Vrabľová; Tomáš Szemes
EPI_ISL_1312062	Public Health Institute of Varaždin County	Croatian Institute of Public Health	Irena Tabain; Ivana Ferenčak
EPI_ISL_2697794	Public Health Laboratory, Minnesota Department of Health	University of Minnesota Genomics Center	Corbin Dirkx; Daryl M. Gohl; Jaquelyn Kuriger-Laber; John Garbe; and Sean Wang
EPI_ISL_2860640, EPI_ISL_2860642, EPI_ISL_2860643, EPI_ISL_2860644, EPI_ISL_2860645, EPI_ISL_2860647	Public Health Reference Laboratory	Erasmus Medical Center	Anne van der Linden; Annemiek van der Eijk; Bas Oude Munnink; Corine GeurtsvanKessel; David Nieuwenhuijse; Emmanuelle Munger; Irina Chestakova; Marion Koopmans; Marjan Boter; Omar Elahmer-Abdulla Bashein- Rahma Algheriani-Ahlam Alarif; Reina Sikkema; Richard Molenkamp; on behalf of the Dutch national COVID-19 response team.
EPI_ISL_3826755	Public Health Service Center 13 Maitree Vanich	Division of Genomic Medicine and Innovation support,Department of Medical Sciences, Ministry of Public Health, Thailand	Archawin Rojanawiwat; Jirapha Pakdee; Natthakul Bunneang; Nuanjun Wichukchinda; Penpitcha Thawong; Pilailuk Akkapaiboon Okada; Pundharika Piboonsiri; Surakameth Mahasirimongkol; Waritta Sawaengdee
EPI_ISL_1098645, EPI_ISL_1910858	Public Health Virology-Forensic and Scientific Services	Public Health Virology-Forensic and Scientific Services	Son Nguyen
EPI_ISL_1495246	Public Health Authority of the Slovak Republic	Bergthaler laboratory, CeMM Research Center for Molecular Medicine of the Austrian Academy of Sciences	Andreas Bergthaler; Anna Schedl; Bekir Erguner; Benedikt Agerer; Christoph Bock; Fabian Amman; Jan Laine; Lukas Endler; Maelle Le Moing; Martin Senekowitsch; Michael Schuster; Petr Triska; Thomas Penz
EPI_ISL_434547	Puerto Rico Department of Health	Centers for Disease Control and Prevention, Dengue Branch	Betzabel Flores; Chaney Kalinich; Fabiola Cruz; Gilberto A. Santiago; Glenda Gonzalez; Jessica I. Falcon; Jorge L. Munoz-Jordan; Joseph Fauver; Keyla Charriez; Nathan Grubaugh
EPI_ISL_3401884	Puskesmas Kecamatan Tanjung Priok	Genomik Solidaritas Indonesia Laboratorium	Ahrahayati Wildany; Annisa Muthiah Sukirman; Anuraj Shankar; Ariel Pradipta; Carissa Sintca Wijaya; Dhahlia Agustina Cahyono; Dwi Oktavia; Gracia Felias Enos Korompis; Himawan Masyhuri; Louisa Markus; Meutia Ayuputeri Kumaheri; Ngabila Salama; Tiranti Vindha; Vania Gavrila Wikasa
EPI_ISL_2918642	Puskesmas Rancamanyar	Eijkman Institute for Molecular Biology, National Research and Innovation Agency; West Java Health Laboratory	Amin Soebandrio; Azzania Fibriani; Cut Nur Cinthia Alamanda; Edison Johar; Ema Rahmawati; Frilasita A Yudhaputri; Hidayat Trimarsanto; Iskandar Adnan; Khin Saw Myint; Lidwina Priliani; Lydia V. Panggalo; Muhammad Rezki Rasyak; Rifky Waluyajati Rachman; Ryan Bayusantika Ristandi; Safarina G Malik; Sukma Oktavianthi; Willy Agustine
EPI_ISL_1055513	QEII Health Sciences Centre	National Microbiology Laboratory (NML)	Anna Majer; Anneliese Landgraff; CanCOGeN's metadata curation team; Dan Gaston; Darian Hole; Elsie Grudeski; Gary Van Domselaar; Grace Seo; Janice Pettipas; Jason LeBlanc; Jennifer Tanner; Kirsten Biggar; Madison Chapel; Morag Graham; Natalie Knox; Nathalie Bastien; Philip Mabon; Public Health Agency of Canada CanCOGeN team; Rhiannon Huzarewich; Russell Mandes; Shari Tyson; Timothy Booth; Todd Hatchette; Yan Li
EPI_ISL_2615848	QLabs	WVU and Marshall University Combined Genomics Core Facilities	James Denvir; Peter Perrotta; Peter Stoilov; Ryan Percifield; Wesley Kimble
EPI_ISL_576132, EPI_ISL_593645, EPI_ISL_693269, EPI_ISL_693282, EPI_ISL_849671, EPI_ISL_849692, EPI_ISL_849694, EPI_ISL_849754, EPI_ISL_1300531, EPI_ISL_1424003, EPI_ISL_1910856, EPI_ISL_2001059, EPI_ISL_2137032, EPI_ISL_2137035, EPI_ISL_2137036, EPI_ISL_2274247, EPI_ISL_2274248, EPI_ISL_2274249, EPI_ISL_2507084, EPI_ISL_2507095, EPI_ISL_2603822, EPI_ISL_2661521, EPI_ISL_2661524, EPI_ISL_2675252, EPI_ISL_2811889, EPI_ISL_2811890, EPI_ISL_2811893, EPI_ISL_2993397, EPI_ISL_3011178, EPI_ISL_3049194, EPI_ISL_3161826, EPI_ISL_3161830, EPI_ISL_3161839, EPI_ISL_3161841, EPI_ISL_3161842, EPI_ISL_3247188, EPI_ISL_3247209, EPI_ISL_3247215, EPI_ISL_3247228, EPI_ISL_3333212, EPI_ISL_3333214, EPI_ISL_3333216, EPI_ISL_3333231			
see above	Queensland Health Forensic and Scientific Services	Queensland Health Forensic and Scientific Services	Chenwei Wang on behalf of Q-PHIRE Genomics; Q-PHIRE Genomics; Son Nguyen; Son Nguyen et al; Son Nguyen et al.
EPI_ISL_530232, EPI_ISL_530252	Queensland Health Forensic and Scientific Services, Public Health Virology	Public Health Virology Laboratory, Forensic and Scientific Services, Queensland Health	Son Nguyen et al
EPI_ISL_1424816	Queensland Medical Laboratories	Melbourne Diagnostic Unit Public Health Laboratory (MDU-PHL)	N.L.; Palou, T.; Seemann, T.; Sherry; Vaccher, S.
EPI_ISL_1424519	Queensland Medical Laboratories	Victorian Infectious Diseases Reference Laboratory (VIDRL) and the Melbourne Diagnostic Unit Public Health Laboratory (MDU-PHL)	N.L.; Palou, T.; Seemann, T.; Sherry; Vaccher, S.
EPI_ISL_571527, EPI_ISL_571551, EPI_ISL_604231, EPI_ISL_937067	Quest Diagnostics	Quest Diagnostics	Anderson, B.; D.F.; Gerasimova, A.; Grover, D.; Hua, M.; K.E.; Kagan; Lacbawan, F.; Liu Y.; Livingston; Owen, R.; R.M.; Rosenthal; S.H.; Shalhout
EPI_ISL_1193694, EPI_ISL_1267222, EPI_ISL_1552330, EPI_ISL_2133768, EPI_ISL_2143015, EPI_ISL_2203892, EPI_ISL_2247317, EPI_ISL_2268458, EPI_ISL_2367831, EPI_ISL_2371770, EPI_ISL_2599691, EPI_ISL_2652279, EPI_ISL_2869008, EPI_ISL_2869532, EPI_ISL_2871151, EPI_ISL_2874485, EPI_ISL_2874557, EPI_ISL_2876883, EPI_ISL_2930265, EPI_ISL_3018772, EPI_ISL_3114334, EPI_ISL_3114731, EPI_ISL_3353026, EPI_ISL_3395610, EPI_ISL_3395993, EPI_ISL_3396787, EPI_ISL_3396958			
see above	Quest Diagnostics Incorporated	Centers for Disease Control and Prevention Division of Viral Diseases, Pathogen Discovery	A. Gerasimova; A. Perez; Adrian Paskey; B. Anderson; Ben L. Rambo-Martin; Benjamin Rambo-Martin; Christopher Gulvick; Clinton R. Paden; Dakota Howard; Darlene Wagner; Dhwani Batra; Duncan MacCannell; F. Lacbawan; I. A. Shlyakhter; Jason Caravas; K.E. Livingston; Kara Moser; L.E. Bernstein; M. Hua; Matthew Schmerer; P. Tanpaiboon; Peter W. Cook; R. M. Kagan; R. Owen; R. V. Rolando; S. H. Rosenthal; Scott Sammons; Shatavia Morrison; Suxiang Tong; Y. Liu; Yvette Unoarumhi

EPI_ISL_415641, EPI_ISL_415644	R. G. Lugar Center for Public Health Research, National Center for Disease Control and Public Health (NCDC) of Georgia.	R. G. Lugar Center for Public Health Research, National Center for Disease Control and Public Health (NCDC) of Georgia.	Adam Kotorashvili; Amiran Gamkrelidze.; Ana Papkiauri; Ann Machablishvili; Anna Kasradze; Davit Tsaguria; Ekaterine Khmaladze; Ekaterine Zangaladze; Ekaterine Zhghenti; Giorgi Tomashvili; Gvantsa Brachveli; Gvantsa Chanturia; Irma Burjanadze; Ketevan Sidamonidze; Khatuna Zakhashvili; Lela Sabadze; Lela Urushadze; Magda Dgebuadze; Maia Alkhazashvili; Mari Gavashelidze; Mariam Zakalashvili; Marine Murtskhvaladze; Meri Pantsulaia; Nato Kotaria; Nino Berishvili; Paata Imnadze; Roena Sukhiashvili; Tamar Jashiashvili; Tata Imnadze; Tea Tevdoradze
EPI_ISL_455783	REGIONAL VRDL,ICMR-RMRC BBSR	Immunogenomics lab, Institute of Life Sciences, Bhubaneswar	Ajay Parida; Arup Ghosh; Atimukta Jha; COVID-19 team of ILS & RMRC; DBT's PAN-INDIA 1000 SARS-CoV2 RNA genome sequencing consortium; Debdutta Bhattacharya; Ghulam Hussain Syed; Jaya Singh Khastri; Jyotirmayee Turuk; Manasi Priyadarshini; Orissa COVID-19 study group; Punit Prasad; Rajeeb Swain; Rupesh Dash; Sanghamitra Pati; Shanti Senapati; Shuchi Smita; Soma Chattopadhyay; Sunil Raghav; Swati Madhulika; Tushar K. Beuria; Viplov K. Biswas
EPI_ISL_2373835	REUNILAB	CNR Virus des Infections Respiratoires - France SUD	Antonin Bal; Bruno Lina; Gregory Destras; Gwendolyne Burfin; Hadrien Regue; Laurence Josset; Martine Valette; Quentin Semanas
EPI_ISL_3130604	REUNILAB ST PAUL	CNR Virus des Infections Respiratoires - France SUD	Antonin Bal; Bruno Lina; Gregory Destras; Gwendolyne Burfin; Hadrien Regue; Laurence Josset; Martine Valette; Quentin Semanas
EPI_ISL_419553	RI State Health Laboratories	Pathogen Discovery, Respiratory Viruses Branch, Division of Viral Diseases, Centers for Disease Control and Prevention	Anna Uehara; Clinton R. Paden; Haibin Wang; Jasmine Padilla; Jing Zhang; Justin Lee; Krista Queen; Suxiang Tong; Yan Li; Ying Tao
EPI_ISL_2273060, EPI_ISL_2454664, EPI_ISL_3451454	ROB FERREIRA LABORATORY	National Institute for Communicable Diseases of the National Health Laboratory Service	Amoako DG; Bhiman JN; Everatt J; Ismail A; Mahlangu B; Mnguni A; Mohale T; Ntuli N; Scheepers C
EPI_ISL_2844827	RS PMI, Bogor, West Java	Biosafety Level-3 Laboratory, Indonesian Institute of Sciences (LIPI)	Anggia Prasetyoputri; Erwin F. Hasrianda; Fahrurrozi; Herjuno A. Nugroho; Nova D. Yanthi; Wien Kusharyoto
EPI_ISL_888986	RSU Bhakti Kartini	Eijkman Institute for Molecular Biology, Ministry of Research and Technology/National Agency for Research and Innovation	Amin Soebandrio; Edison Johar; Frilasita A Yudhaputri; Hidayat Trimarsanto; Iskandar Adnan; Khin Saw Myint; Lydia V. Panggalo; Safarina G Malik; Sukma Oktavianthi; Willy Agustine
EPI_ISL_2258213	RSUD Arifin Ahmad Riau	National Institute of Health Research and Development	Arie Ardiansyah Nugraha; Fajri Marinda; Hana Apsari Pawestri; Hartanti Dian Ikawati; Kartika Dewi Puspa; Krisna Pangesti; Nelly Puspandari; Subangkit; Triyani Soekarso; Vivi Setiawaty
EPI_ISL_791986	RSUD Morotai Maluku Utara	National Institute of Health Research and Development	AA; Denggo; HA; HD; Ikawati; KD; KNA; N; Nugraha; Pangesti; Pawestri; Puspa; Puspandari; SD; Setiawaty; Soekarso; Subangkit; T; V
EPI_ISL_3230241	RSUP FATMAWATI / DINAS KESEHATAN DKI JAKARTA	Genomik Solidaritas Indonesia Laboratorium	Annisa Muthiah Sukirman; Anuraj Shankar; Ariel Pradipta; Carissa Sintca Wijaya; Gracia Felias Enos Korompis; Jerry; Lidya Utami; Loli Simajuntak; Meutia Ayuputeri Kumaheri; Reinhart Gabriel; Vania Gavrila Wikasa
EPI_ISL_768616	Rajavithi Hospital	National Institute of Health, Department of Medical Sciences, Ministry of Public Health, Thailand	; Natchaya Khiadsang; Pakorn Piromtong; Pilailuk Okada; Ratana Tacharoenmuang; Siripaporn Phuygun; Sittiporn Parnmen; Sunthareeya Waicharoen; Thanutsapa Thanadachakul; Warawan Wongboot; sirikanda wimol
EPI_ISL_2348644	Rakai Health Sciences Program	MRC/UVRI & LSHTM Uganda Research Unit	Charles Ssuuna; Dan Lule Bugembe; Matthew Cotten; My V.T. Phan; Pontiano Kaleebu; Ronald Moses Galiwango; Steven J Reynolds
EPI_ISL_447029, EPI_ISL_3152924	Ramathibodi Hospital	COVID-19 Network Investigations (CONI) Alliance	Angkana Huang; Anthony R. Jones; Arporn Wangwiwatsin; Bhakbhoom Panthan; Chonticha Klungtong; Duangkamon Loesbanluechai; Ekawat Pasomsub; Elizabeth Batty; Insee Sensorn; Janjira Thaipadungpanit; Khajohn Joonlasak; Khajohn Joonsalak; Kingkan Rakmanee; Krittikorn Kumpornsin; Namfon Kotanan; Stefan Fernandez; Thanat Chookajorn; Theerarat Kochakarn; Treewat Watthanachockchai; Wasun Chantratita; Wudtichai Manasatienkij
EPI_ISL_2000576	Rami Kantor lab	Rami Kantor lab	Ewa King; Josephine Darpolor; Mark Howison; Rami Kantor; Richard Huard; Vlad Novitsky
EPI_ISL_2157585	Red - Regional de Vigilancia Genómica del COVID-19	Laboratory of Respiratory Viruses and Measles, Oswaldo Cruz Institute, FIOCRUZ	Alice Sampaio Rocha; Ana Carolina Mendonca; Anna Carolina Paixao; Claudia Díaz; Eilisa Cavalcante Pereira; Fernando Motta; Luciana Appolinario; Marilda Siqueira on behalf of the Fiocruz COVID-19 Genomic Surveillance Network; Mitzi Castro; Paola Resende; Renata Serrano Lopes; Sandra Paola Paz; Taina Venas
EPI_ISL_2433442	Regional Medical Science Center 2 Phitsanulok	Division of Genomic Medicine and Innovation support,Department of Medical Sciences, Ministry of Public Health, Thailand	Archawin Rojanawiwat; Jirapha Pakdee; Natthakul Bunneang; Nuanjun Wichukchinda; Penpitcha Thawong; Pilailuk Akkapaiboon Okada; Pundharika Piboonsiri; Surakameth Mahasirimongkol; Waritta Sawaengdee
EPI_ISL_3797055	Regional Medical Sciences Center 12 Songkhla	National Institute of Health, Department of Medical Sciences, Ministry of Public Health, Thailand	Archawin Rojanawiwat; Natchaya Khiadsang; Nuttida Thongpramul; Pakorn Piromtong; Pilailuk Okada; Ratana Tacharoenmuang; Siripaporn Phuygun; Sittiporn Parnmen; Sunthareeya Waicharoen; Thanutsapa Thanadachakul; Warawan Wongboot; sirikanda wimol
EPI_ISL_3342069	Regional Medical Sciences Center 8 Udonthani	National Institute of Health, Department of Medical Sciences, Ministry of Public Health, Thailand	; Natchaya Khiadsang; Nuttida Thongpramul; Pakorn Piromtong; Pilailuk Okada; Ratana Tacharoenmuang; Siripaporn Phuygun; Sittiporn Parnmen; Sunthareeya Waicharoen; Thanutsapa Thanadachakul; Warawan Wongboot; sirikanda wimol
EPI_ISL_708804, EPI_ISL_708806, EPI_ISL_708807	Regional medical sciences center 6 chonburi	National Institute of Health, Department of Medical Sciences, Ministry of Public Health, Thailand	Malinee Chittaganpitch; Pakorn Piromtong; Pilailuk Okada; Siripaporn Phuygun; Sittiporn Parnmen; Sunthareeya Waicharoen; Thanutsapa Thanadachakul; Warawan Wongboot
EPI_ISL_3122992, EPI_ISL_3122996, EPI_ISL_3122998	Republican Children's Clinical Infectious Diseases Hospital	WHO National Influenza Centre Russian Federation	Alexey Masharsky; Andrey Komissarov; Artem Fadeev; Daria Danilenko; Dmitry Lioznov; Elena Nabieva; Georgii Bazykin; Kirill Varchenko; Ksenia Safina; Kseniya Komissarova; Maria Baturova; Maria Pisareva; Mikhail Bakaev; Nikita Yolshin; Oula Mansour; Tamila Musaeva; Veronika Eder
EPI_ISL_491474	Research Institute for Tropical Medicine	Research Institute for Tropical Medicine	Catalino Demetria; Criselda Bautista; Daria Manalo; Edelwisa Mercado; Francisco Gerardo Polotan; Inez Andrea Medado; Kirstyn Brunker; Ma. Angelica Tujan; Othoniel Jan Onza
EPI_ISL_2154909	Research Institute for Tropical Medicine, Inc. (RITM)	Philippine Genome Center	Alethea R. de Guzman; Anna Ong-Lim; Arianne A. Zamora; Asia Louisa U. Chong; Benedict A. Maralit; Candice Francheska B. Tambaoan; Carlo M. Lapid; Celia Carlos; Devon Ray Pacial; Edsel Maurice Salvaña; El King D. Morado; Eva Maria Cutiongco-de la Paz; Francis A. Tablizo; Irish Coleen A. Asin; Jaime C. Montoya; Jan Michael C. Yap; Jo-Hannah S. Llames; John Q. Wong; Joshua Gregor A. Dizon; Juan Antonio R. Magalang; Karol Sophia Agape R. Padilla; Kenneth M. Kim; Kris P. Punayan; Marc Edsel C. Ayes; Marc Jerrone R. Castro; Maria Rosario Singh-Vergeire and Cynthia P. Saloma; Maria Sofia L. Yangzon; Marissa Alejandria; Razel Nikka M. Hao; Rianna Patricia S. Cruz; Sheila Mae M. Araiza
EPI_ISL_2544708, EPI_ISL_2544712	Research Center for Emerging Viral Infections, Chang Gung University, Taiwan	Research Center for Emerging Viral Infections, Chang Gung University, Taiwan	Carol Wang; Chung-Guei Huang; Hsiao-Chen Tu; Hui-Ying Weng; Hung-Yu Shu; Jason Su; Jora Lin; Kuo-Ming Lee; Po-Wei Huang; Pocky Lai; Shih-Feng Tsai; Shin-Ru Shih; Shu-Li Yang; Tsu-Lan Wu; Yu-Nong Gong; Yung-Feng Lin
EPI_ISL_2737733, EPI_ISL_2737737	Respiratory Virus Unit, Microbiology Services Colindale, Public Health England	COVID-19 Genomics UK (COG-UK) Consortium	PHE Covid Sequencing Team
EPI_ISL_747099, EPI_ISL_747132	Respiratory Viruses Branch, Centers for Disease Control and Prevention	Respiratory Viruses Branch, Centers for Disease Control and Prevention	C.R.; Cook; Lee, J.; Li, Y.; Marine, R.; Montmayeur, A.; P.W.; Paden; Queen, K.; Sheth, M.; Tao, Y.; Tong, S.; Uehara, A.; Wang, H.
EPI_ISL_977077, EPI_ISL_3525915	Rhode Island Department of Health	Infectious Disease Program, Broad Institute of Harvard and MIT	Adams, G.; Azevedo, K.; B.L.; B.W.; Bauer, M.; Birren; Carter, A.; Chaluvadi, S.; D.J.; DeRuff, K.; Gallagher, G.; Gladden-Young, A.; Huard, R.; J.E.; K.J.; King, E.; Lagerborg, K.; Lemieux; Loreth, C.; MacInnis; Miller, A.; Normandin, E.; P.C.; Park; Pearlman, L.; Reilly, S.; Rudy, M.; Sabeti; Siddle; Smole, S.; Tomkins-Tinch, C.; and MacInnis; and Sabeti
EPI_ISL_2603333, EPI_ISL_3024110	Rhode Island State Health Laboratory	Rhode Island State Health Laboratory	Ewa King; Kristin Carpenter-Azevedo; Richard C. Huard
EPI_ISL_2798289, EPI_ISL_2798298, EPI_ISL_2798406	Riga East University Hospital, National Microbiology Reference Laboratory	Riga East University Hospital, National Microbiology Reference Laboratory; Eurofins Genomics Europe Sequencing GmbH	Arzu Algulieva; Diāna Dušacka; Dārta Pūpola; Ilva Pole; Jevgenijs Bodrenko; Jūlija Čevere; Reinis Vangravs; Reinis Zeltmatis; Sergejs Ņikišins; Ģirts Šķenders
EPI_ISL_419831	Royal Darwin Hospital	Victorian Infectious Diseases Reference Laboratory and Microbiological Diagnostic Unit Public Health Laboratory, Doherty Institute	Caly L.; Druce J.; Meumann, E.; Sait, M.; Schultz M.; Seemann T.
EPI_ISL_521860, EPI_ISL_521862, EPI_ISL_779406, EPI_ISL_779408, EPI_ISL_854745, EPI_ISL_2321157, EPI_ISL_2379269, EPI_ISL_2839562, EPI_ISL_2839563, EPI_ISL_2839567, EPI_ISL_2839568, EPI_ISL_2907544, EPI_ISL_2907545, EPI_ISL_2907546, EPI_ISL_3030412			
see above	Royal Darwin Hospital Pathology	MDU-PHL	Caly L.; Druce J.; M.L.; Meumann, E.; N.L.; Sait; Sait, M.; Schultz M.; Seemann T.; Sherry; Sherry, N.
EPI_ISL_2250195	Royal Darwin Hospital Pathology	Microbiological Diagnostic Unit Public Health Laboratory (MDU-PHL)	Caly L.; Druce J.; M.L.; Meumann, E.; N.L.; Sait; Seemann T.; Sherry
EPI_ISL_426902	Royal Darwin Hospital Pathology	Microbiological Diagnostic Unit Public Health Laboratory and Victorian Infectious Diseases Reference Laboratory, Doherty Institute	Caly L.; Druce J.; Meumann, E.; Sait, M.; Schultz M.; Seemann T.; Sherry, N.
EPI_ISL_430631	Royal Darwin Hospital Pathology	Microbiological Diagnostic Unit Public Health Laboratory and Victorian Infectious Diseases Reference Laboratory, The Peter Doherty Institute for Infection and Immunity	Caly L.; Druce J.; Meumann, E.; Sait, M.; Schultz M.; Seemann T.; Sherry, N.
EPI_ISL_456497	Royal Darwin Hospital Pathology	Microbiological Diagnostic Unit Public Health Laboratory, The Peter Doherty Institute for Infection and Immunity	Caly L.; Druce J.; Meumann, E.; Sait, M.; Schultz M.; Seemann T.; Sherry, N.
EPI_ISL_577607, EPI_ISL_3247060	Royal Hobart Hospital	Royal Hobart Hospital	Cooley L.; Dr L. Cooley; Mr R Vanhaeften; van Haeften R.
EPI_ISL_522621, EPI_ISL_522636, EPI_ISL_522689, EPI_ISL_522705	Royal Hobart Hospital Microbiology Department	MDU-PHL	Cooley L.; M.B.; Sait M.; Schultz; Seemann T.; Sherry N.; van Haeften R.
EPI_ISL_451145, EPI_ISL_467989, EPI_ISL_468039, EPI_ISL_483126, EPI_ISL_508606, EPI_ISL_516550, EPI_ISL_622762, EPI_ISL_654808, EPI_ISL_752598, EPI_ISL_771368, EPI_ISL_1029956, EPI_ISL_1704790, EPI_ISL_2462350, EPI_ISL_2462351, EPI_ISL_2462352, EPI_ISL_2462353, EPI_ISL_2462355, EPI_ISL_2462356, EPI_ISL_2462358, EPI_ISL_2462361, EPI_ISL_2462364, EPI_ISL_2462365, EPI_ISL_2839981, EPI_ISL_2839983, EPI_ISL_2839984, EPI_ISL_2839987, EPI_ISL_2839989, EPI_ISL_2839993, EPI_ISL_2839994, EPI_ISL_2839996, EPI_ISL_2978690, EPI_ISL_2978692, EPI_ISL_3071861, EPI_ISL_3071863, EPI_ISL_3391989, EPI_ISL_3391992, EPI_ISL_3710500, EPI_ISL_3710501, EPI_ISL_3710502, EPI_ISL_3710503, EPI_ISL_3710505, EPI_ISL_3710510, EPI_ISL_3710512, EPI_ISL_3710513			
see above	SA Pathology	SA Pathology	Caitlin Selway; Chuan Kok Lim; Geoff Higgins; Ivan Bastian; Julien Soubrier; Karin Kassahn; Lex Leong; Luke Walters; Mark Turra; Song Gao
EPI_ISL_455603	SA Pathology	VPRL	Beard, MR.; C.K.; Coldbeck-Shackley, R.; Kirby, E.; L.E.X.; Leong; Lim; Llamas, B.; Merrett, J.; Shue, B.; Van Der Hoek, K.
EPI_ISL_2209888	SANTA CASA DE MISERICORDIA SAO JOSE	Instituto Butantan / Mendelics	Antonio Jorge Martins; Claudia Renata dos Santos Barros; David Schlesinger; Debora Botequio Moretti; Dimas Tadeu Covas; Elaine Cristina Marqueze; Elaine Vieira Santos; Evandra Strazza Rodrigues; Heidge Fukumasu; Jayme Augusto de Souza-Neto; José Salvatore Leister Patané; Luiz Alcantara; Luiz Lehmann Coutinho; Maria Carolina Elias; Maurício Lacerda Nogueira; Rafael dos Santos Bezerra; Raul Machado Neto; Rejane Maria Tommasini Grotto; Ricardo Haddad; Sandra Coccuzzo Sampaio Vessoni; Simone Kashima; Svetoslav Nanev Slavov; Vincent Louis Viala

EPI_ISL_2791863, EPI_ISL_2792359, EPI_ISL_2958166, EPI_ISL_2986100, EPI_ISL_3646356	SARS-CoV-2 Sequencing Castilla y Leon-Spain Consortium	SARS-CoV-2 Sequencing Castilla y Leon-Spain Consortium	Antonio Orduña-Domingo; Carlos Fuster Foz; Carmen Aldea-Mansilla; Carmen Gimeno Crespo; David Abad; Gabriel March Rosello; Gabriel March Rossello; Gregoria Megías Lobón; Jose María Eiros Bouza; Laura Sánchez de Prada; M. Isabel Fernandez-Natal; Marta Dominguez-Gil; Marta Hernandez; María Antonia García Castro; Mª Fe Brezmes-Valdivieso; Noelia Arenal Andrés; Silvia Rojo; Sonsoles Garcinuño Pérez
EPI_ISL_2930583, EPI_ISL_3195597, EPI_ISL_3199067, EPI_ISL_3543127	SARS-CoV-2 testing team, National Institute of Infectious Diseases	Pathogen Genomics Center, National Institute of Infectious Diseases	Hazuka Y Furihata; Hiromizu Takahashi; Kentaro Itokawa; Makoto Kuroda; Masanori Hashino; Masumichi Saito; Naomi Nojiri; Nozomu Hanaoka; Rina Tanaka; Tsuguto Fujimoto; Tsuyoshi Sekizuka
EPI_ISL_2828403	SC (UCO) Igiene e Sanità Pubblica, ASUGI, Trieste	ARGO Laboratorio Genomica ed Epigenomica	Barbone F; Breda C; Busetti M; D'Agaro P; Dal Monego S; Degasperi M; Fontana F; Licastro D; Marcello A; Piscianz E; Segat L
EPI_ISL_3355211, EPI_ISL_3813516	SD Public Health Laboratory	Centers for Disease Control and Prevention Division of Viral Diseases, Pathogen Discovery	Alex Burgin; Ben Rambo-Martin; Clinton Paden; Dakota Howard; Dave Wentworth; Dhwani Batra; Jasmine Padilla; Justin Lee; Krista Queen; Kristen Knipe; Kristine Lacek; Mark Burroughs; Matthew Schmerer; Meghan Bentz; Mili Sheth; Peter Cook; Sam Shepard; Sarah Nobles; Suxiang Tong; Vivien Dugan; Yvette Unoarumhi
EPI_ISL_2156799	SD Public Health Laboratory	Pathogen Discovery, Respiratory Viruses Branch, Division of Viral Diseases, Centers for Disease Control and Prevention	Adam Retchless; Anna Kelleher; Anna Uehara; Brian Lynch; Clinton R. Paden; Dhwani Batra; Haibin Wang; Han Jia Justin Ng; Jasmine Padilla; Jing Zhang; Justin Lee; Krista Queen; Mark Burroughs; Mili Sheth; Morgan Davis; Peter Cook; Rachel Marine; Sarah Nobles; Suxiang Tong; Tara Coalter; Yan Li; Ying Tao
EPI_ISL_2966511	SE Kyiv CDC MHU	The Institute of Molecular Biology and Genetics of NASU	M.Tukalo et al.
EPI_ISL_2934570, EPI_ISL_2934573, EPI_ISL_2966512	SECP MHU	The Institute of Molecular Biology and Genetics of NASU	M.Tukalo et al.
EPI_ISL_3636644	SEEBMO	Instituto Nacional de Saude (INSA)	Borges et al
EPI_ISL_2983897	SELAS MEDILYS	Department of Virology, Henri Mondor University Hospital, Assistance Publique Hôpitaux de Paris, Université Paris-Est Créteil, INSERM U955	Alexandre Soulier; Christophe Rodriguez; Elisabeth Trawinski; Guillaume Gricourt; Jean-Michel Pawlotsky; Melissa N'Debi; Slim Fourati; Vanessa Demontant
EPI_ISL_3385835	SERVICIOS MEDICOS OLIMPUS	Instituto Nacional de Salud- Dirección de Investigación en Salud Pública	Carlos Franco-Muñoz; Carmen Osorio; Diana Malo; Diego A. Álvarez-Díaz; Diego Andrés Prada; Gerardo Santamaría; Hector Alejandro Ruiz-Moreno; Jhonnatan Reales-González; Jorge Rivera; Juan Camilo Martinez; Julian Naizaque; Katherine Laiton-Donato; Lisseth Pardo; Magdalena Wiesner; Marcela Mercado-Reyes; Maria T. Herrera-Sepúlveda; Marta Lopez Blanco; Martha Lucia Ospina Martinez; Paola Rojas; Sergio Gomez; Sheryll Corchuelo; Ángela Alarcon Cruz
EPI_ISL_3262126	SI «Public Health Center of MHU»	The Institute of Molecular Biology and Genetics of NASU	M.Tukalo et al.
EPI_ISL_2600819	SIESP CHIETI - DRIVE IN VASTO	Istituto Zooprofilattico Sperimentale dell'Abruzzo e Molise "G. Caporale"	Ancora M; Calistri P; Cammà C; Caporale M; Curini V; Delli Compagni E; Di Domenico M; Di Lollo Valeria; Di Pasquale A; Lorusso A; Mangone I; Marcacci M; Puglia I; Rinaldi A; Savini G; Scialabba S
EPI_ISL_2131385	SIESP DIPARTIMENTO DI PREVENZIONE TERAMO	Istituto Zooprofilattico Sperimentale dell'Abruzzo e Molise "G. Caporale"	Ancora M; Calistri P; Cammà C; Caporale M; Curini V; Delli Compagni E; Di Domenico M; Di Lollo Valeria; Di Pasquale A; Lorusso A; Mangone I; Marcacci M; Puglia I; Rinaldi A; Savini G; Scialabba S
EPI_ISL_2565131, EPI_ISL_2586843, EPI_ISL_2587835, EPI_ISL_2883604, EPI_ISL_3255597, EPI_ISL_3534795	SK-Roy Romanow Provincial Laboratory	National Microbiology Laboratory (NML)	Alanna Senecal; Amanda Lang; Anna Majer; Anneliese Landgraff; CanCOGeN's metadata curation team; Darian Hole; Elsie Grudeski; Gary Van Domselaar; Grace Seo; Jennifer Tanner; Jessica Minion; Kara Loos; Keith MacKenzie; Kirsten Biggar; Madison Chapel; Meredith Faires; Morag Graham; Natalie Knox; Nathalie Bastien; Philip Mabon; Public Health Agency of Canada CanCOGeN team; Rachel; Rachel DePaulo; Rhiannon Huzarewich; Russell Mandes; Ryan McDonald; Shari Tyson; Timothy Booth; Yan Li
EPI_ISL_3251603	SP ZOZ Centralny Szpital Kliniczny UM w Lodzi Pracownia Diagnostyki Wirusow Oddechowych	Wojewodzka Stacja Sanitarno-Epidemiologiczna w Rzeszowie, Laboratorium Diagnostyki Medycznej	Anna Nowakowska; Karolina Ostrowska; Katarzyna Wilk; Marzena Baranowska
EPI_ISL_2230114	SP ZOZ Centralny Szpital Kliniczny UM w Łodzi Zakład Diadnostyli Labolatoryjnej Pracownia Wirusów Oddechowych	1. National Institute of Public Health - National Institute of Hygiene; 2. Eurofins Genomics Europe Sequencing GmbH	ECDC COVID-19 WGS support team; Eurofins Genomics Europe Sequencing Team; Gierczyńki Rafał; Sadkowska-Todys Małgorzata; Wołkowicz Tomasz; Zacharczuk Katarzyna
EPI_ISL_2139699, EPI_ISL_2139705	SYNLAB	GIGA Medical Genomics	Bouchra Boujemla; Cécile Meex; Keith Durkin; Maria Artesi; Marie-Pierre Hayette; Nathalie Renotte; Pierrette Melin; Raphaël Boreux; Sébastien Bontems; Vincent Bours
EPI_ISL_2642840	SYNLAB Eesti OÜ	Department of Microbiology, Institute of Biomedicine and Translational Medicine, University of Tartu	Aare Abroi; Andrio Lahesaare; Arina Shablinskaja; Dagmar Hoidmets; Ene-Ly Jõgeda; Eveli Kallas; Heiki Niglas; Irja Lutsar; Kai Truusalu; Kaisa Truus; Katrin Kaarna; Kristi Huik; Liidia Dotsenko; Lili Azin Milani; Mari-Anne Härma; Mats Hansen; Meri Pauskar; Olga Sadikova; Paul Naaber; Radko Avi; Taavi Päll; Tuuli Reisberg; Ulvi Gerst Talas
EPI_ISL_1645330, EPI_ISL_2128387	SYNLAB Jena Oncoscreen	Robert Koch Institute	
EPI_ISL_3496538	SYNLAB Labor München Zentrum LMZ	Robert Koch Institute	
EPI_ISL_2114081, EPI_ISL_2844954	SYNLAB MVZ Leverkusen	Robert Koch Institute	
EPI_ISL_2116799	SYNLAB MVZ Trier	Robert Koch Institute	
EPI_ISL_1157473	SYNLAB MVZ Weiden	Robert Koch Institute	
EPI_ISL_2230856, EPI_ISL_2230904, EPI_ISL_2444430, EPI_ISL_2444524, EPI_ISL_2978498, EPI_ISL_3160813, EPI_ISL_3277674, EPI_ISL_3503605, EPI_ISL_3503622, EPI_ISL_3548281, EPI_ISL_3721688, EPI_ISL_3721752			
see above	Salud Digna	Instituto Nacional de Medicina Genomica	Abraham Campos-Romero; Cedro-Tanda A; Escobar-Arrazola; Escobar-Arrazola MA; Gonzalez-Barrera D; Herrera-Montalvo LA.; Hidalgo-Miranda A; Luna-Ruiz Marco; M; Mendoza-Vargas A; Moreno-Camacho José Luis; Munguia-Garza P; Ramirez-Vega O; Rangel-DeLeon D; Reyes-Grajeda JP; Rodriguez-Gallegos Jorge; Yair Alfaro-Mora
EPI_ISL_3373994, EPI_ISL_3374008	Salud Digna, A.C	Andersen lab at Scripps Research	Abraham Garcia Gil; Jose Luis Moreno Camacho; Marco Antonio Luna Ruiz-Esparza; Miguel A. Fernandez Rojas; SEARCH Alliance with Abraham Campos Romero
EPI_ISL_2199992	Samodzielny Publiczny Zespół Opieki Zdrowotnej w Brzesku	1. Tricity SARS-CoV-2 sequencing consortium: University of Gdansk, Medical University of Gdansk, Vaxican Ltd., Invicta Ltd. 2. National Institute of Public Health - National Institute of Hygiene, Warsaw, Poland	Celina Cybulska; Karolina Gackowska; Katarzyna Groth; Katarzyna Zacharczuk; Krystyna Bienkowska Szewczyk; Lukasz Rabalski; Maciej Grzybek; Maciej Kosinski; Magdalena Nowakowska; Małgorzata Sadkowska-Todys; Tomasz Wołkowicz
EPI_ISL_755292	San Diego County Public Health Laboratory	Andersen lab at Scripps Research	Brett Austin; Jovan Shephard; SEARCH Alliance San Diego with Tracy Basler
EPI_ISL_2156226	San Lazaro Hospital (SLH)	Philippine Genome Center	Alethea R. de Guzman; Anna Ong-Lim; Arianne A. Zamora; Asia Louisa U. Chong; Benedict A. Maralit; Candice Francheska B. Tambaoan; Carlo M. Lapid; Celia Carlos; Devon Ray Pacial; Edsel Maurice Salvaña; El King D. Morado; Eva Maria Cutiongco-de la Paz; Francis A. Tablizo; Irish Coleen A. Asin; Jaime C. Montoya; Jan Michael C. Yap; Jo-Hannah S. Llames; John Q. Wong; Joshua Gregor A. Dizon; Juan Antonio R. Magalang; Karol Sophia Agape R. Padilla; Kenneth M. Kim; Kris P. Punayan; Marc Edsel C. Ayes; Marc Jerrone R. Castro; Maria Rosario Singh-Vergeire and Cynthia P. Saloma; Maria Sofia L. Yangzon; Marissa Alejandria; Razel Nikka M. Hao; Rianna Patricia S. Cruz; Sheila Mae M. Araiza
EPI_ISL_542367	San Matteo Hospital Pavia	Dep. Of Oncology and Hemato-Oncology University of Milan	Antonio Piralla; Carlo Federico Perno; Chiara Vismara; Claudia Alteri; Elisa Matarazzo; Fausto Baldanti; Federica Giardina; Federica Novazzi; Luna Colagrossi; Maria Antonello; Massimo Puoti; Monica Tallarita; Oscar Massimiliano Epis; Roberto Fumagalli; Silvia Renica; Stefano Gaiarsa; Valentino Costabile; Valeria Cento
EPI_ISL_748667, EPI_ISL_750171, EPI_ISL_750177	Sanatorio Americano	Institut Pasteur de Montevideo	Ana Carolina Mendonça; Andrés Lizasoain; Camila Simoes; Cecilia Alonso; Cecilia Salazar; Daiana Mir; Fernando López-Tort; Fernando Motta; Gonzalo Bello; Ighor Arantes; Ignacio Ferrés; Jose Sotelo; Leticia Maya; Letícia Garay Martins; Luciana Appolinario; Lucía Spangenberg; Mailen Arleo; Mariana Brandes; Marilda Mendonça Siqueira; Marilda Tereza Mar da Rosa; María José Benitez-Galeano; Martín Graña; Matías Castells; Matías Victoria; Matías Salvo; Natalia Rego; Natalia Reyes; Pablo Smircich; Paola Cristina Resende; Rodney Colina; Tamara Fernandez-Calero; Tania Possi; Tatiana Schäffer Gregianini; Verónica Noya; Yasser Vega
EPI_ISL_1439597, EPI_ISL_1477049	Sanitary-Epidemiological And Public Health Department Of Tashkent Region, Uzbekistan	Center of Genomics and bioinformatics, Bioinformatics laboratory	Abdurakhmon N Yusupov; Ibrokhim Y Abdurakhmonov.; Mirzakamol S Ayubov; Mukhammadjon H Mirzakhmedov; Shukhrat E Shermatov; Zabardast T Buriev
EPI_ISL_479800	Sapporo City Institute of Public Health	Pathogen Genomics Center, National Institute of Infectious Diseases	Asami Ohnishi; Hajime Kamiya; Kentaro Itokawa; Makoto Kuroda; Masanori Hashino; Motoi Suzuki; Rina Tanaka; Tsuyoshi Sekizuka
EPI_ISL_3611031	Satkhira Medical College, Satkhira	Virology Laboratory, International Centre for Diarrhoeal Disease Research, Bangladesh (ICDDR,B)	ASM Alamgir; Abhijit Guho; Ahmed Nawsher Alam; Dinesh Mondal; Firdausi Qadri; Hassan Afrad; Mahbubur Rahman; Manjur Hossain Khan; Md. Mahfuzur Rahman; Mohammad Enayet Hossain; Mohammad Jubair; Mohammad Shahidul Islam; Mohammed Ziaur Rahman; Mojnu Miah; Mustafizur Rahman; Razib Mazumder; Samir Kumar Saha; Senjuti Saha; Tahmina Shirin
EPI_ISL_1009160	School of Pharmacy	School of Pharmacy	Ahmed Kandeil; Ghazi Kayali; Mina Nabil Kamel; Mohamed A Ali; Rabeh El-Shesheny; Walid Abi Habib
EPI_ISL_904004	Scientific Veterinary Institute Novi Sad	Veterinary Specialized Institute "Kraljevo", Serbia	Afonso, C.; Banovic Djeri, B.; Jankovic, M.; Jovanovic, T.; Knezevic, A.; Petrovic, T.; Sekler, M.; Tesovic, B.; Vidanovic, D.; Volkening, J.
EPI_ISL_525721	Seattle Flu Study	Seattle Flu Study	Amanda Adler; Barry R. Lutz; Benjamin Pelle; Caitlin R. Wolf; Chris D. Frazar; Deborah A. Nickerson; Elisabeth Brandstetter; Helen Y. Chu; Janet A. Englund; Jay Shendure; Jeff Duchin; Jover Lee; Kairsten Fay; Karen Cowgill; Kirsten Lacombe; Lea M. Starita; Mark J. Rieder; Matthew Richardson; Matthew Thompson; Melissa Truong; Michael Boeckh; Michael Famulare; Misja Ilcisin; Peter D. Han; Stephanie Schrag; Thomas R. Sibley; Trevor Bedford
EPI_ISL_856767, EPI_ISL_2136102, EPI_ISL_2158700, EPI_ISL_2158704, EPI_ISL_2158706, EPI_ISL_2158767, EPI_ISL_2158826			
see above	Servicio Virosis Respiratorias-Departamento Virología-INEI	Instituto Nacional Enfermedades Infecciosas C.G.Malbran	Avaro M.; Baumeister E.; Benedetti E.; Campos J.; Cisterna D.; Dattero ME; Lorenzo F.; Molina V.; Perandones C.; Poklepovich T.; Pontoriero A.; Russo M.; Tuduri E.
EPI_ISL_911290	Servicio de Microbiología, Hospital Universitario Son Espases	SeqCOVID-SPAIN consortium/IBV(CSIC)	Antonio Oliver and SeqCOVID-SPAIN consortium; Carla López-Causapé; Jordi Reina
EPI_ISL_2545298, EPI_ISL_2567037,	Shamir Medical Center (Asaf Harofe)	Shamir Medical Center (Asaf Harofe)	Abu Hamad Ramzia; Adina Bar Chaim; Anna Vishnevsky; Chen Weiner; Netta Zuckerman; Nir Rainy; Patricia Benveniste-Lekovitz; Reut Sorek Abramovich; Yevgeni Yegorov

EPI_ISL_2834318, EPI_ISL_2834420, EPI_ISL_3667329, EPI_ISL_3667406			
EPI_ISL_1468958	Sharp HealthCare Laboratory	Andersen lab at Scripps Research	Art Mendoza; Cathy Woerle; Jacquelyn Berumen; Liam McGinnis; Omid Bakhtar; SEARCH Alliance San Diego with Aaron Harding
EPI_ISL_582641, EPI_ISL_582648	Sheikh Khalifa Medical City	Molecular/Surveillance lab Sheikh Khalifa Medical City	Amirtharaj Francis; Hala Imambaccus; Hiba Saud; Sahar Almarzooqi; Sajeed Abdul; Stefan Weber
EPI_ISL_2432957, EPI_ISL_2931282	Shenzhen Center for Disease Control and Prevention	National Institute for Viral Disease Control and Prevention, China CDC	Can Zhu; Fang Yang; Kai Nie; Long Chen; Peihua Niu; QingJu Lu; RenLi Zhang; Renli Zhang; Shaoyu Deng and Yaqing He; ShiSong Fang; Weihua Wu; Xiang Zhao; XiaoLiang Xiao; XinYi Wei; Xinyi Wei; YaQing He; Yenan Feng; Yue Li
EPI_ISL_2106272	Siem Reap Provincial Laboratory	Virology Unit, Institut Pasteur du Cambodge	Cecile Troupin; Chau Darapheak; Chin Savuth; Erik A Karlsson; Jurre Y Siegers; Kraing Sidonn; Lamleav Leak; Leakhena Pum; Ly Sovann; Veasna Duong; Yi Sengdoeurn
EPI_ISL_500540	Singapore General Hospital	Department of Microbiology	Chenhao Li; Karrie Ko; Kern Rei Chng; Kian Sing Chan; Kun Lee Lim; Lynette Oon; Niranjan Nagarajan; Nurdyana Abdul Rahman
EPI_ISL_1285461, EPI_ISL_3495619	Sonic - Bioscientia - MVZ Labor Saar GmbH	Robert Koch Institute	
EPI_ISL_1155027	Sonic - MVZ Medizinisches Labor Bremen GmbH	Robert Koch Institute	
EPI_ISL_3216891	South Dakota Department Of Health Laboratory	Minnesota Department of Health, Public Health Laboratory	Alexandra Lorentz; Jacob Garfin; Matt Plumb; and Xiong Wang
EPI_ISL_1017035	South Dakota Public Health Laboratory	University of Minnesota Genomics Center	Benjamin Auch; Corbin Dirkx; Daryl M. Gohl; Jaquelyn Kuriger-Laber; John Garbe; and Chris Carlson
EPI_ISL_667803, EPI_ISL_1383237, EPI_ISL_2405113, EPI_ISL_2828071	South Eastern Area Laboratory Services (SEALS)	NSW Health Pathology - Institute of Clinical Pathology and Medical Research; Westmead Hospital; University of Sydney	CIDM-PH et al.
EPI_ISL_2928016, EPI_ISL_2928020, EPI_ISL_2928022, EPI_ISL_2928026, EPI_ISL_2928027, EPI_ISL_3546327, EPI_ISL_3546329, EPI_ISL_3546331, EPI_ISL_3546332, EPI_ISL_3546334, EPI_ISL_3546336, EPI_ISL_3546337, EPI_ISL_3546338, EPI_ISL_3546341, EPI_ISL_3546350, EPI_ISL_3546351			
see above	South Sudan Ministry of Health, WHO South Sudan, MRC/UVRI & LSHTM Uganda Research Unit	MRC/UVRI & LSHTM Uganda Research Unit, South Sudan Ministry of Health, WHO South Sudan	Abe G. Abias; Dan Lule Bugembe; Dennis Kenyi Lodiongo; James Ayei; John Rumunu; Joseph Francis Wamala; Juma John HM; Lul Lojok Deng; Matthew Cotten; My V.T. Phan; Pontiano Kaleebu; Richard Lino Loro Lako; Sudhir Bunga
EPI_ISL_456162, EPI_ISL_456297, EPI_ISL_3164095, EPI_ISL_3164113, EPI_ISL_3164114, EPI_ISL_3164115, EPI_ISL_3164116, EPI_ISL_3164117, EPI_ISL_3164119, EPI_ISL_3164122			
see above	Southern Community Labs Dunedin	Institute of Environmental Science and Research (ESR)	Anja Werno; Antje van der Linden; Arlo Upton; Chris Mansell; David Hammer; Dragana Drinkovic; Erasmus Smit; Gary McAuliffe; Hana Sofia Andersson; Hermes Perez; James Ussher; Jill Sherwood; Jing Wang; Joep de Ligt; Josh Freeman; Julia Howard; Juliet Elvy; Lauren Jelly; Mary DeAlmeida; Matt Blakiston; Matt Storey; Matthew Rogers; Max Bloomfield; Michael Addidle; Michelle Balm; Muhammad Faisal; Nikki Freed; Olin Silander; Olivia Stroeven; Paula scholes; Rachel Boyle; Sally Roberts; SallyAnn Harbison; Sarah Jefferies; Sharmini Muttaiyah; Susan Lin; Susan Morpeth; Susan Taylor; Timothy Blackmore; Vani Sathyendran; Veronica Playle; Virginia Hope; Xiaoyun Ren
EPI_ISL_3465414	Southern. IML Pathology	NSW Health Pathology - Institute of Clinical Pathology and Medical Research; Westmead Hospital; University of Sydney	Arnott A.; Draper J.; Gall M.; Martinez E.; Rockett R.; Sintchenko V.; on behalf of ICPMR
EPI_ISL_513364, EPI_ISL_2812885, EPI_ISL_3185235	St Vincent's Pathology (SydPath)	NSW Health Pathology - Institute of Clinical Pathology and Medical Research; Westmead Hospital; University of Sydney	CIDM-PH et al.
EPI_ISL_3769556, EPI_ISL_3827508	State Hygienic Laboratory at the University of Iowa	State Hygienic Laboratory at the University of Iowa	Alankar Kampoowale; Anna Yakos; Cindy Toll; Davis Rieckenberg; Erik Twait; Jeff Benfer; Kris Eveland; Kristen Zanon; Mariah Knutson; Mohammed Allam; Valerie Reeb; Wes Hottel
EPI_ISL_1495112	State Institution «Public Health Center of Ministry of Health of Ukraine»	Robert Koch Institute, ZBS1 Highly Pathogenic Viruses, Berlin, Germany	Andreas Nitsche; Annika Brinkmann; Iryna Demchyshyna; Janine Michel; Liudmyla Chernenko; Roman Rodyna; Steven Uddin
EPI_ISL_2724751, EPI_ISL_3033311, EPI_ISL_3307845	State Laboratories Division, Hawaii State Department of Health	State Laboratories Division, Hawaii State Department of Health	Ayana Garnet; Daniel Strange; Drew Kuwazaki; Edward Desmond; Pamela O'Brien; Razvan Sultana
EPI_ISL_2378161	State Testing Facility	Altius Institute for Biomedical Research	Alex Isner; Alex Nguyen; Amanda Gale; Audra Johnson; Clem Green; Daniel Bates; Eric Thorland; Jacob Rodriguez; Jean Robinston; Jemma Nelson; Jessica Kunder; John Stamatoyannopoulos; Joshua Richards; Julia Wald; Kneshay Harper; Lauren Mitchell; Mark Frerker; Matt Hartman; Michael Buckley; Muhammad Halimun; Rebecca Bruders; Sadie Patraw; Sofia Olsson; Tobias Ragoczy
EPI_ISL_2685167	Statens Serum Institut Bioinformatics and Microbial Genomics	Statens Serum Institut Bioinformatics and Microbial Genomics	Danish Covid-19 Genome Consortium
EPI_ISL_491087	Suceava County Emergency Hospital	"Stefan cel Mare" University Metagenomics Lab	Antoniadis Panagiotis et al.; Lobiuc Andrei
EPI_ISL_3333656	Sultanah Bahiyah Hospital, Alor Setar	Institute for Medical Research, Infectious Disease Research Centre, National Institutes of Health, Ministry of Health Malaysia	Anasir MI; Azizan MA; Kamel K; Mohd Zawawi Z; Ramly N; Robert F; Suppiah J; Thayan R
EPI_ISL_1618581, EPI_ISL_1618717, EPI_ISL_1659723, EPI_ISL_2206051, EPI_ISL_2208089, EPI_ISL_2278840, EPI_ISL_2416113, EPI_ISL_2416224, EPI_ISL_2793372, EPI_ISL_2980825, EPI_ISL_3157117, EPI_ISL_3157751, EPI_ISL_3783217, EPI_ISL_3784811			
see above	Swedish national genomic surveillance program of SARS-CoV-2	The Public Health Agency of Sweden	Alma Brolund; Maria Lind Karlberg; Maximilian Riess; Swedish national genomic surveillance program of SARS-CoV-2
EPI_ISL_2544248	Swiss Analysis AG	Institute of Medical Virology	Alexandra Trkola; Annette Audigé; Cyril Shah; Gabriela Ziltener; Guido Bloemberg; Jon Huder; Jürg Böni; Kevin Steiner; Maria Grünberg; Maryam Zaheri; Michael Huber; Riccarda Capaul; Stefan Schmutz; Verena Kufner
EPI_ISL_2812822, EPI_ISL_3465688	Sydney South West Pathology Service (SSWPS) - Liverpool Hospital - NSW Health Pathology	NSW Health Pathology - Institute of Clinical Pathology and Medical Research; Westmead Hospital; University of Sydney	Arnott A.; CIDM-PH et al.; Draper J.; Gall M.; Martinez E.; Rockett R.; Sintchenko V.; on behalf of ICPMR
EPI_ISL_545039, EPI_ISL_2462426, EPI_ISL_2828059, EPI_ISL_2828067	Sydney South West Pathology Service (SSWPS) - Royal Prince Alfred Hospital - NSW Health Pathology	NSW Health Pathology - Institute of Clinical Pathology and Medical Research; Westmead Hospital; University of Sydney	CIDM-PH et al.
EPI_ISL_2546970, EPI_ISL_2547009, EPI_ISL_2547049, EPI_ISL_2788840, EPI_ISL_3025707, EPI_ISL_3087661, EPI_ISL_3505850, EPI_ISL_3507062, EPI_ISL_3507076, EPI_ISL_3507229, EPI_ISL_3665778, EPI_ISL_3665822			
see above	Synlab Eesti OÜ	1. Laboratory of Communicable Diseases (Estonia); 2. Eurofins Genomics Europe Sequencing GmbH	Liidia Dotsenko et al.
EPI_ISL_3236951	TAMBO MEMORIAL LABORATORY	National Institute for Communicable Diseases of the National Health Laboratory Service	Amoako DG; Bhiman JN; Everatt J; Ismail A; Mahlangu B; Mnguni A; Mohale T; Ntuli N; Scheepers C
EPI_ISL_914813	TAMIZAJE COMUNITARIO - PASO CANOAS	Inciensa, Instituto Costarricense de Investigación y Enseñanza en Nutrición y Salud	Adriana Godínez; Claudio Soto-Garita; Estela Cordero; Francisco Duarte; Hebleen Porras; Melany Calderón & Mariel López
EPI_ISL_3088724, EPI_ISL_3325748	TGen North	TGen North	"Jolene Bowers; Brett Van Tassel; Chris French; Darrin Lemmer; Dave Engelthaler; Dave Engelthaler"; Hayley Yaglom; Heather Centner; Jolene Bowers
EPI_ISL_2156332	THE LORD'S GRACE MEDICAL AND INDUSTRIAL CLINIC	Philippine Genome Center	Alethea R. de Guzman; Anna Ong-Lim; Arianne A. Zamora; Asia Louisa U. Chong; Benedict A. Maralit; Carlo Francheska B. Tambaoan; Carlo M. Lapid; Celia Carlos; Devon Ray Pacial; Edsel Maurice Salvaña; El King D. Morado; Elcid Aaron R. Pangilinan; Eva Maria Cutiongco-de la Paz; Francis A. Tablizo; Irish Coleen A. Asin; Jaime C. Montoya; Jan Michael C. Yap; Jo-Hannah S. Llames; John Q. Wong; Joshua Gregor A. Dizon; Juan Antonio R. Magalang; Karol Sophia Agape R. Padilla; Kenneth M. Kim; Kris P. Punayan; Marc Edsel C. Ayes; Marc Jerrone R. Castro; Maria Rosario Singh-Vergeire and Cynthia P. Saloma; Maria Sofia L. Yangzon; Marissa Alejandria; Razel Nikka M. Hao; Rianna Patricia S. Cruz; Sheila Mae M. Araiza
EPI_ISL_436099, EPI_ISL_2693000	TSGH-CP molecular lab	TSGH-CP molecular lab	Cherng-Lih Perng; Chien-Wen Chen; Chih-Kai Chang; Feng-Yee Chang; Hsing-Yi Chung; Hung-Sheng Shang; Jung-Chung Lin; Kuo-Ming Yeh; Kuo-Sheng Hung; Ming-Jr JIAN; Sheng-Kang Chiu; Shih-Hung Tsai; Tien-Yao Chang
EPI_ISL_2364005, EPI_ISL_3230306	TYKS, Kliininen mikrobiologia	Expert Microbiology, National Institute for Health and Welfare	Carita Savolainen-Kopra; Erika Lindh; Haider al-Hello; Jani Halkilahti; Kirsi Liitsola; Niina Ikonen; Olli Vapalahti; Pekka Ellonen; Phuoc Truong; Päivi Laurila; Ravi Kant; Sari Hannula; Soile Blomqvist; Teemu Smura
EPI_ISL_2931333, EPI_ISL_3019481, EPI_ISL_3019485, EPI_ISL_3547090, EPI_ISL_3547094	Temporary Specimen Collection Centre at the AsiaWorld-Expo	Hong Kong Department of Health	Alan K.L. Tsang; Edman T.K. Lam; Ken H.L. Ng; Peter C.W. Yip; Rickjason C.W. Chan
EPI_ISL_756311, EPI_ISL_1490226, EPI_ISL_2230692, EPI_ISL_2230696, EPI_ISL_2230698, EPI_ISL_2478954, EPI_ISL_2478969, EPI_ISL_2478992, EPI_ISL_2478994, EPI_ISL_2545198			
see above	The Caribbean Public Health Agency	Carrington Lab, Department of PreClinical Sciences, Faculty of Medical Sciences, The University of the West Indies	Adesh Ramsubhag; Anushka Ramjag; Arianne Brown-Jordan; Avery Hinds; Ayoola Oyinloye; Chinna Chinnadurai; Christine V. F. Carrington; Christopher Oura; Gabriel Escobar; Jaya Jayaraman; Jerome Foster; Karla Georges; Marsha Ivey; Naresh Nandram; Narine Singh; Nikita S. D. Sahadeo; Nuno Faria; Oliver Pybus; Rahul Naidu; Rajini Haraksingh; Risha Singh; Roshan Parasram; Sarah Hill; Sharon Belmar-George; Shawn Charles; Simone Keizer-Beache; Stanley Giddings; SueMin Nathaniel; Vernie Ramkissoon
EPI_ISL_2631433, EPI_ISL_2631435, EPI_ISL_2631440	The Caribbean Public Health Agency	Carrington Lab, Department of PreClinical Sciences, Faculty of Medical Sciences, The University of the West Indies, St Augustine Campus	Anushka Ramjag; Arianne Brown-Jordan; Avery Hinds; Christine V. F. Carrington; Christopher Oura; David Johnson; Gabriel Escobar; Karla Georges; Naresh Nandram; Nikita S. D. Sahadeo; Nuno Faria; Oliver Pybus; Risha Singh; Roshan Parasram; Sarah Hill; SueMin Nathaniel; Vernie Ramkissoon
EPI_ISL_2621682, EPI_ISL_2621699	The Caribbean Public Health Agency	Carrington Lab, Department of Preclinical Sciences, Faculty of Medical Sciences, The University of the	Anushka Ramjag; Arianne Brown-Jordan; Avery Hinds; Christine V. F. Carrington; Christopher Oura; Gabriel Escobar; Karla Georges; Kenneth George; Nikita S. D. Sahadeo; Nuno Faria; Oliver Pybus; Risha Singh; Roshan Parasram; Sarah Hill; SueMin Nathaniel; Vernie Ramkissoon

		West Indies	
EPI_ISL_2617930, EPI_ISL_2678143, EPI_ISL_2678171, EPI_ISL_2678174, EPI_ISL_2678180, EPI_ISL_2716571, EPI_ISL_2716576, EPI_ISL_2716577, EPI_ISL_2716580, EPI_ISL_2716592, EPI_ISL_2716595, EPI_ISL_2716602, EPI_ISL_2756569, EPI_ISL_2756582, EPI_ISL_2967979, EPI_ISL_2967981, EPI_ISL_2967991, EPI_ISL_2967993, EPI_ISL_3610771, EPI_ISL_3642741, EPI_ISL_3655553, EPI_ISL_3655572, EPI_ISL_3655576, EPI_ISL_3688254			
see above	The Caribbean Public Health Agency	Carrington Lab, Department of Preclinical Sciences, Faculty of Medical Sciences, The University of the West Indies, St Augustine Campus	Anushka Ramjag; Arianne Brown-Jordan; Avery Hinds; Christine V. F. Carrington; Christopher Oura; Gabriel Escobar; Hazel Laws; Karla Georges; Kenneth George; Naresh Nandram; Nikita S. D. Sahadeo; Nuno Faria; Oliver Pybus; Rhonda Sealey-Thomas; Risha Singh; Roshan Parasram; Sarah Hill; Sharon Belmar-George; Shawn Charles; Simone Keizer-Beache; SueMin Nathaniel; Vernie Ramkissoon
EPI_ISL_2154525	The Lord's Grace Medical and Industrial Clinic	Philippine Genome Center	Alethea R. de Guzman; Anna Ong-Lim; Arianne A. Zamora; Asia Louisa U. Chong; Benedict A. Maralit; Candice Francheska B. Tambaoan; Carlo M. Lapid; Celia Carlos; Devon Ray Pacial; Edsel Maurice Salvaña; El King D. Morado; Eva Maria Cutiongco-de la Paz; Francis A. Tablizo; Irish Coleen A. Asin; Jaime C. Montoya; Jan Michael C. Yap; Jo-Hannah S. Llames; John Q. Wong; Joshua Gregor A. Dizon; Juan Antonio R. Magalang; Karol Sophia Agape R. Padilla; Kenneth M. Kim; Kris P. Punayan; Marc Edsel C. Ayes; Marc Jerrone R. Castro; Maria Rosario Singh-Vergeire and Cynthia P. Saloma; Maria Sofia L. Yangzon; Marissa Alejandria; Razel Nikka M. Hao; Rianna Patricia S. Cruz; Sheila Mae M. Araiza
EPI_ISL_541334, EPI_ISL_960436	The National Institute of Public Health	State Veterinary Institute Prague	A; Cernikova; D; H; J; Jirincova; L; M; Nagy; Novakova; Stara; Trnka; Vecerova
EPI_ISL_826855, EPI_ISL_828505	The National University Hospital of Iceland	deCODE genetics	Agnar Helgason; Alma Moller; Arna B Agustsdottir; Arnaldur Gylfason; Asgeir Sigurdsson; Aslaug Jonasdottir; Berglind Eiriksdottir; Bjarni Thorbjornsson; Brynjar O Jensson; Daniel F Gudbjartsson; Droplaug N Magnusdottir; Elisabet E Gardarsdottir; Emil A Thorarensen; Gardar Sveinbjornsson; Gisli Masson; Gudmundur Georgsson; Gudmundur L Norddahl; Gudrun Sigmundsdottir; Hakon Jonsson; Hannes Eggertsson; Hilma Holm; Ingileif Jonsdottir; Jona Saemundsdottir; Kamilla S Josefsdottir; Kari Stefansson; Karl G Kristinsson; Kjartan R Gudmundsson; Kristin E Sveinsdottir; Louise le Roux; Maney Sveinsdottir; Olafia S Gretarsdottir; Olafur T Magnusson; Pall Melsted; Patrick Sulem; Run Fridriksdottir; Solvi Rognvaldsson; Thora R Gunnarsdottir; Thordur Kristjansson; Thorolfur Gudnason; Unnur Thorsteinsdottir
EPI_ISL_3620743	The Ohio State University Applied Microbiology Services Laboratory	The Ohio State University Applied Microbiology Services Laboratory	Seth A. Faith PhD
EPI_ISL_754231	The Republican Research and Practical Center for Epidemiology and Microbiology (RRPCEM)	WHO National Influenza Centre Russian Federation	Anatoly Krasko; Andrey Komissarov; Anna Ivanova; Artem Fadeev; Daria Danilenko; Dmitry Bazhenov; Dmitry Lioznov; Elena Gasich; Elena Nabieva; Georgii Bazykin; Kirill Bulda; Ksenia Safina; Kseniya Komissarova
EPI_ISL_2631429	The Trinidad and Tobago Public Health Laboratory	Carrington Lab, Department of PreClinical Sciences, Faculty of Medical Sciences, The University of the West Indies, St Augustine Campus	Anushka Ramjag; Arianne Brown-Jordan; Avery Hinds; Christine V. F. Carrington; Christopher Oura; Gabriel Escobar; Karla Georges; Naresh Nandram; Nikita S. D. Sahadeo; Nuno Faria; Oliver Pybus; Risha Singh; Roshan Parasram; Sarah Hill; SueMin Nathaniel; Vernie Ramkissoon
EPI_ISL_2383206	The teaching Baghdad hospital	PCR lab	Abdulhussein Thair; Fadhil Hula
EPI_ISL_3150205	Tivoli Fondazione Policlinico Universitario Agostino Gemelli IRCCS	Fondazione Policlinico Universitario Agostino Gemelli	Istituto di Microbiologia e Virologia
EPI_ISL_690802	Tokyo Metropolitan Institute of Public Health	Pathogen Genomics Center, National Institute of Infectious Diseases	Kentaro Itokawa; Makoto Kuroda; Masanori Hashino; Rina Tanaka; Tsuyoshi Sekizuka
EPI_ISL_586278, EPI_ISL_933649	Toronto Invasive Bacterial Diseases Network	McMaster University	Ahmed Draia; Allison McGeer; Andrew G. McArthur; Angel Li; Emily Panousis; Hooman Derakhshani; Jalees Nasir; Kuganya Nirmalarajah; Michael Surette; Patryk Aftanas; Samira Mubareka
EPI_ISL_3274703	TriCore Reference Laboratories	Center for Global Health, University of New Mexico Health Sciences Center	Cecilia Thompson; Darrell Dinwiddie; Daryl Domman; Karissa Culbreath; Kendra Pesko; Kurt Schwalm; Valerie Morley
EPI_ISL_717692, EPI_ISL_717698, EPI_ISL_2230681	Trinidad Public Health Laboratory	Carrington Lab, Department of PreClinical Sciences, Faculty of Medical Sciences, The University of the West Indies	Adesh Ramsubhag; Arianne Brown-Jordan; Avery Hinds; Chinna Chinnadurai; Christine V. F. Carrington; Christopher Oura; Gabriel Escobar; Jaya Jayaraman; Jerome Foster; Karla Georges; Marsha Ivey; Naresh Nandram; Nikita S. D. Sahadeo; Nuno Faria; Oliver Pybus; Rahul Naidu; Rajini Haraksingh; Risha Singh; Roshan Parasram; Sarah Hill; Stanley Giddings; SueMin Nathaniel; Vernie Ramkissoon
EPI_ISL_3610769, EPI_ISL_3610772, EPI_ISL_3634447, EPI_ISL_3634448, EPI_ISL_3642745, EPI_ISL_3655577, EPI_ISL_3845997, EPI_ISL_3845999, EPI_ISL_3846004, EPI_ISL_3846011, EPI_ISL_3846017			
see above	Trinidad Public Health Laboratory	Carrington Lab, Department of PreClinical Sciences, Faculty of Medical Sciences, The University of the West Indies, St Augustine Campus	Anushka Ramjag; Arianne Brown-Jordan; Avery Hinds; Christine V. F. Carrington; Christopher Oura; Gabriel Escobar; Nikita S. D. Sahadeo; Nuno Faria; Oliver Pybus; Risha Singh; Roshan Parasram; Sarah Hill; SueMin Nathaniel; Vernie Ramkissoon
EPI_ISL_539819	Tuen Mun Hospital	Hong Kong Department of Health	Alan K.L. Tsang; Dominic N.C. Tsang; Edman T.K. Lam; Peter C.W. Yip; Rickjason C.W. Chan
EPI_ISL_3082641	Tuzla University Clinical Center, Department of Microbiology	University of Sarajevo, Veterinary Faculty, Laboratory for Molecular Diagnostic and Research Laboratory	Goletic S; Goletic T; Hodzic A.; Jazic A.; Nicevic M.; Sabic E.; Softic A; Terzic I.; Tihic N
EPI_ISL_3184720	U.O. Microbiologia Laboratorio Unico Centro Servizi - AUSL della Romagna	U.O. Microbiologia, Laboratorio Unico Centro Servizi - AUSL della Romagna	Giorgio Dirani
EPI_ISL_2625912	UAB "Rezus.lt"	Institute of Biotechnology, Life Sciences Center, Vilnius University	Emilija Vasiliunaite__ Milda Norkiene__ Danguole Ziogiene__ Albertas Timinskas__ Alma Gedvilaite
EPI_ISL_2694306	UAB "Rezus.lt"	National Public Health Surveillance Laboratory	Ana Steponkiene; Danas Baksa; Jelena Razmuk; Lukas Vasionis; Lukas Zemaitis; Migle Gabrielaite; Svajune Muralyte
EPI_ISL_3060118	UAB "Rezus.lt"	Vilnius University Hospital Santaros Klinikos, Center of Laboratory Medicine	Daniel Naumovas; Dovile Juozapaite; Gytis Dudas; Ingrida Olendraite; Ligita Raugaite; Monika Katenaite; Rimvydas Norvilas
EPI_ISL_3711466	UAB Diagnostikos laboratorija	National Public Health Surveillance Laboratory	Ana Steponkiene; Danas Baksa; Jelena Razmuk; Lukas Vasionis; Lukas Zemaitis; Migle Gabrielaite; Svajune Muralyte
EPI_ISL_3481592	UAB InMedica	unknown	Astra Vitkauskiene; Darius Cereskevicius; Inga Nasvytiene; Mantas Sarauskas; Marius Sukys; Rasa Ugenskiene; Renaldas Jurkevicius; Rima Vainoriene; Zivile Zemeckiene
EPI_ISL_2227176	UAB Medicina practica laboratorija	Lithuanian University of Health Sciences	Arnoldas Pautienius; Dovydas Gecys; Gediminas Alzbutas; Kamile Tamusauskaite; Lukas Zemaitis; Vaiva Lesauskaite
EPI_ISL_2082432	UAB Medicina practica laboratorija	National Public Health Surveillance Laboratory	Ana Steponkiene; Danas Baksa; Jelena Razmuk; Lukas Vasionis; Lukas Zemaitis; Migle Gabrielaite; Svajune Muralyte
EPI_ISL_2345671	UBS II DE ALVARES MACHADO	Instituto Butantan / Mendelics	Antonio Jorge Martins; Claudia Renata dos Santos Barros; David Schlesinger; Debora Botequio Moretti; Dimas Tadeu Covas; Elaine Cristina Marqueze; Elaine Vieira Santos; Evandra Strazza Rodrigues; Heidge Fukumasu; Jayme Augusto de Souza-Neto; José Salvatore Leister Patané; Luiz Alcantara; Luiz Lehmann Coutinho; Maria Carolina Elias; Maurício Lacerda Nogueira; Rafael dos Santos Bezerra; Raul Machado Neto; Rejane Maria Tommasini Grotto; Ricardo Haddad; Sandra Coccuzzo Sampaio Vessoni; Simone Kashima; Svetoslav Nanev Slavov; Vincent Louis Viala
EPI_ISL_2445202	UBS II DE TANABI MILTON MARTINS PERCHES	Instituto Butantan	Antonio Jorge Martins; Claudia Renata dos Santos Barros; David Schlesinger; Debora Botequio Moretti; Dimas Tadeu Covas; Elaine Cristina Marqueze; Elaine Vieira Santos; Evandra Strazza Rodrigues; Heidge Fukumasu; Jayme Augusto de Souza-Neto; José Salvatore Leister Patané; Luiz Alcantara; Luiz Lehmann Coutinho; Maria Carolina Elias; Maurício Lacerda Nogueira; Rafael dos Santos Bezerra; Raul Machado Neto; Rejane Maria Tommasini Grotto; Ricardo Haddad; Sandra Coccuzzo Sampaio Vessoni; Simone Kashima; Svetoslav Nanev Slavov; Vincent Louis Viala
EPI_ISL_3370431, EPI_ISL_3535719	UC-Christus Clinical Hospital Laboratory	Laboratory of Molecular Virology, School of Medicine, Pontificia Universidad Catolica de Chile	Ana Maira Guzman; Andres E. Munoz-Marcos; Catalina Pardo-Roa; Eileen Serrano; Erick Salinas; Estefany Poblete; Francisco Melo; Jorge Levican; Leonardo I. Almonacid; Maria Jose Avendano; Maria Patricia Vega; Rafael A. Medina; Ricardo Enrique de la Barra; Tamara Garcia-Salum
EPI_ISL_3104798	UCC (Universidad Central del Caribe)	Grubaugh Lab - Yale School of Public Health	Alejandaro Vallejo Degaudenzi; Anderson Brito; Annie Watkins; Chaney Kalinich; Chantal Vogels; Elisa Contreras; Esperanza Mendoza; Isabel Ott; Jessica Rothman; Joseph Fauver; Kendall Billig; Mallery Breban; Mary Petrone; Nathan Grubaugh; Robert Paulino-Ramirez; Tara Alpert; Tobias Koch; Victor Virgilio Calderon
EPI_ISL_628761, EPI_ISL_648124, EPI_ISL_648125	UHAS COVID-19 Lab	UHAS COVID-19 Lab	John O. Gyapong and the UHAS COVID-19 Lab Team; Jones Gyamfi; Kwabena O. Duedu; Reuben Ayivor-Djanie
EPI_ISL_3071388	ULS Castelo Branco	Instituto Nacional de Saude (INSA)	Borges et al
EPI_ISL_2249196, EPI_ISL_2249198	ULS Castelo Branco	Instituto Nacional de Saude (INSA) and Centro de Investigaçao em Biodiversidade e Recursos Geneticos (CIBIO), Universidade do Porto	Borges et al
EPI_ISL_2455132	UM im. Karola Marcinkowskiego w Poznaniu Laboratorium UCA_Covid-19 Centrum Biologii Medycznej	1. Tricity SARS-CoV-2 sequencing consortium: University of Gdansk, Medical University of Gdansk, Vaxican Ltd., Invicta Ltd. 2. National Institute of Public Health - National Institute of Hygiene, Warsaw, Poland	Celina Cybulska; Karolina Gackowska; Katarzyna Groth; Katarzyna Zacharczuk; Krystyna Bienkowska Szewczyk; Lukasz Rabalski; Maciej Grzybek; Maciej Kosinski; Magdalena Nowakowska; Marcin Lubocki; Małgorzata Sadkowska-Todys; Tomasz Wołkowicz
EPI_ISL_2284514	UMC Groningen, Clinical Virology, Department of Medical Microbiology and Infection Prevention	UMC Groningen, Clinical Virology, Department of Medical Microbiology and Infection Prevention	Alexander Friedrich; Coretta Van Leer-Buter; Erley Lizarazo-Forero; Hubert Niesters; Lilli Gard; Marjolein Knoester; Monika Fliss; Sigrid Rosema; Xuewei Zhou
EPI_ISL_733234, EPI_ISL_770647	UMMC-Health	WHO National Influenza Centre Russian Federation	Andrey Komissarov; Anna Ivanova; Artem Fadeev; Daria Danilenko; Dmitry Bazhenov; Dmitry Lioznov; Elena Nabieva; Georgii Bazykin; Ksenia Safina; Kseniya Komissarova; Tatiana Platonova
EPI_ISL_1937993, EPI_ISL_1938084	UNC Charlotte COVID-19 Testing Lab	UNC Charlotte Environmental Monitoring Laboratory	Angelica Martins; Cynthia Gibas; Jannatul Ferdous; Jessica Schlueter; Kevin Lambirth; Visva Barua
EPI_ISL_3066764	UNIDAD DE DIAGNOSTICO HEMATO ONCOLOGICA	Instituto Nacional de Salud	Carlos Franco-Muñoz; Carmen Osorio; Diana Malo; Diego A. Álvarez-Díaz; Diego Andrés Prada; Gerardo Santamaría; Hector Alejandro Ruiz-Moreno; Jhonnatan Reales-González; Jorge Rivera; Juan Camilo Martinez; Julian Naizaque; Katherine Laiton-Donato; Lisseth Pardo; Magdalena Wiesner; Marcela Mercado-Reyes; Maria T.Herrera-Sepúlveda; Marta Lopez Blanco; Martha Lucia Ospina Martinez; Paola Rojas; Sergio Gomez; Sheryll Corchuelo; Ángela Alarcon Cruz
EPI_ISL_2646114, EPI_ISL_2895591	UNILABS	Instituto Nacional de Saude (INSA)	Borges et al
EPI_ISL_2796490	UNILABS	Instituto Nacional de Saude (INSA) and BioSystems & Integrative Sciences Institute (BioISI) Genomics Unit, FCUL	Borges et al
EPI_ISL_1820926	UNIVERSIDAD DE Magdalena	Instituto Nacional de Salud- Dirección de Investigación en Salud Pública	Carlos Franco-Muñoz; Carmen Osorio; Christian Romero; Diana Malo; Diego A. Álvarez-Díaz; Diego Andrés Prada; Gerardo Santamaría; Hector Alejandro Ruiz-Moreno; Jhonnatan Reales-González; Jorge Rivera; Juan Camilo Martinez; Julian Naizaque; Katherine Laiton-Donato; Lisseth Pardo; Magdalena Wiesner; Marcela Mercado-Reyes; Maria T. Herrera-Sepúlveda; Marta Lopez Blanco; Martha Lucia Ospina Martinez; Paola Rojas; Patricia del Portillo; Sergio Gomez; Sheryll Corchuelo; Ángela Alarcon Cruz
EPI_ISL_982575, EPI_ISL_1038796, EPI_ISL_2383376, EPI_ISL_2661466, EPI_ISL_3048168, EPI_ISL_3048171, EPI_ISL_3375857			

see above	US Air Force School of Aerospace Medicine	US Air Force School of Aerospace Medicine	Amanda Javorina; Anthony Fries; Carol Garrett; Clarise Starr; Cole Anderson; Elizabeth Macias; Fritz Castillo; Jennifer Black; Jennifer Meyer; Sarah Purves; William Buggele; William Gruner
EPI_ISL_1324136, EPI_ISL_2340786, EPI_ISL_2432917, EPI_ISL_2604039, EPI_ISL_2773920, EPI_ISL_2803153, EPI_ISL_3412153			
see above	UW Virology Lab	UW Virology Lab	Alexander Greninger; Hong Xie; Keith R Jerome; Lasata Shrestha; Margaret Mills; Maria Lukes; Meei-Li Huang; Michelle Lin; Nathan Breit; Noah Baker; Noah R. Baker; Patrick Mathias; Pavitra Roychoudhury; Ricardo Perez; Robert J. Livingston; Saraswathi Sathees; Sean Ellis; Shah Mohamed Bakhash; Tien V. Nguyen
EPI_ISL_734958	UZ Leuven, National Reference Laboratory for Coronaviruses, Laboratory Medicine, Leuven, Belgium	KU Leuven, Rega Institute, Clinical and Epidemiological Virology	Bert Vanmechelen; Joan Marti-Carerras; Piet Maes; Tony Wawina-Bokalanga
EPI_ISL_737952, EPI_ISL_737965	Uganda Central Public Health Lab and Uganda Virus Research Institute	MRC/UVRI & LSHTM Uganda Research Unit	Dan Lule Bugembe; Matthew Cotten; My V.T. Phan; Pontiano Kaleebu et al.
EPI_ISL_2671760, EPI_ISL_2942750, EPI_ISL_2970038, EPI_ISL_3805647, EPI_ISL_3805653	Unidad de Investigacion Biomedica de Zacatecas (UIBZ)	Unidad de Genomica Avanzada	; Alejandra Garcia-Gasca; Alejandra Hernandez-Teran; Alejandro Sanchez-Flores; Alfredo Herrera-Estrella; Alicia Ocaña-Mondragon; Andreu Comas-Garcia; Angel Gustavo Salas-Lais; Antonio Loza Roman; Bernardo Martinez-Miguel; Blanca Taboada; Brenda Irasema Maldonado-Meza; Bruno Gomez-Gil; Carla Ivon Herrera-Najera; Carlos F. Arias; Celia Boukadida; Celida Duque Molina; Celida Martinez- Rodriguez; Clara Esperanza Santacruz-Tinoco; Concepcion Grajales-Muñiz; Consorcio Mexicano de Vigilancia Genomica (CoViGen-Mex). Authors (in alphabetical order): Julio Elias Alvarado-Yaah; Cristobal Chaidez-Quiroz; Daniel Fregoso-Rueda; Daniel Lira Morales; Eduardo Becerril-Vargas; Fernando Fontove-Herrera; Fidencio Mejia-Nepomuceno; Francisco Pulido; Gloria Elena Espinosa-Ayala; Gloria Maria Molina-Salinas; Gloria Vazquez; Hector Esteban Paz-Juarez; Hector Montoya-Fuentes; Helen Haydee Fernanda Ramirez-Plascencia; Irvin Gonzalez-Lopez; Jean Pierre Gonzalez; Jesus Hernandez; Joel Armando Vazquez-Perez.; Jorge Salas-Hernandez; Jose Antonio Enciso-Moreno; Jose Arturo Martinez-Orozco; Jose Esteban Muñoz-Medina; Jose de Jesus Nuñez-Contreras; Juan Bautista Chale-Dzul; Julissa Enciso-Ibarra; Luis Alberto Ochoa-Carrera; Margarita Matias-Florentino; Maria Guadalupe Santiago-Mauricio; Maria Guadalupe de Jesus Mireles-Rivera; Mario Mujica-Sanchez; Marissa Perez-Garcia; Nelly Selem-Mojica; Pavel Isa; Ricardo Ciria Merce; Ricardo Grande; Rosa Maria Gutierrez Rios; Santiago avila-Rios; Selene Zarate; Susana Lopez; Veronica Mata-Haro; Victor Eduardo Garcia-Arias; Victor Hugo Borja-Aburto
EPI_ISL_2942636, EPI_ISL_2942641, EPI_ISL_2970044, EPI_ISL_3805660, EPI_ISL_3805743	Unidad de Investigacion Medica de Yucatan (UIMY)	Unidad de Genomica Avanzada	; Alejandra Garcia-Gasca; Alejandra Hernandez-Teran; Alejandro Sanchez-Flores; Alfredo Herrera-Estrella; Alicia Ocaña-Mondragon; Andreu Comas-Garcia; Angel Gustavo Salas-Lais; Antonio Loza Roman; Bernardo Martinez-Miguel; Blanca Taboada; Brenda Irasema Maldonado-Meza; Bruno Gomez-Gil; Carla Ivon Herrera-Najera; Carlos F. Arias; Celia Boukadida; Celida Duque Molina; Celida Martinez- Rodriguez; Clara Esperanza Santacruz-Tinoco; Concepcion Grajales-Muñiz; Consorcio Mexicano de Vigilancia Genomica (CoViGen-Mex). Authors (in alphabetical order): Julio Elias Alvarado-Yaah; Cristobal Chaidez-Quiroz; Daniel Fregoso-Rueda; Daniel Lira Morales; Eduardo Becerril-Vargas; Fernando Fontove-Herrera; Fidencio Mejia-Nepomuceno; Francisco Pulido; Gloria Elena Espinosa-Ayala; Gloria Maria Molina-Salinas; Gloria Vazquez; Hector Esteban Paz-Juarez; Hector Montoya-Fuentes; Helen Haydee Fernanda Ramirez-Plascencia; Irvin Gonzalez-Lopez; Jean Pierre Gonzalez; Jesus Hernandez; Joel Armando Vazquez-Perez.; Jorge Salas-Hernandez; Jose Antonio Enciso-Moreno; Jose Arturo Martinez-Orozco; Jose Esteban Muñoz-Medina; Jose de Jesus Nuñez-Contreras; Juan Bautista Chale-Dzul; Julissa Enciso-Ibarra; Luis Alberto Ochoa-Carrera; Margarita Matias-Florentino; Maria Guadalupe Santiago-Mauricio; Maria Guadalupe de Jesus Mireles-Rivera; Mario Mujica-Sanchez; Marissa Perez-Garcia; Nelly Selem-Mojica; Pavel Isa; Ricardo Ciria Merce; Ricardo Grande; Rosa Maria Gutierrez Rios; Santiago avila-Rios; Selene Zarate; Susana Lopez; Veronica Mata-Haro; Victor Eduardo Garcia-Arias; Victor Hugo Borja-Aburto
EPI_ISL_2490537	Unidad de Investigación Biomédica de Zacatecas (UIBZ)	Centro de Investigación en Enfermedades Infecciosas (CIENI), Instituto Nacional de Enfermedades Respiratorias (INER)	; Alejandra Garcia-Gasca; Alejandra Hernández-Terán; Alejandro Sanchez-Flores; Alfredo Herrera-Estrella; Alicia Ocaña-Mondragón; Andreu Comas-García; Angel Gustavo Salas-Lais; Antonio Loza Román; Bernardo Martínez-Miguel; Blanca Taboada; Brenda Irasema Maldonado-Meza; Bruno Gomez-Gil; Carla Ivón Herrera-Najera; Carlos F. Arias; Celia Boukadida; Clara Esperanza Santacruz-Tinoco; Concepción Grajales-Muñiz; Consorcio Mexicano de Vigilancia Genómica (CoViGen-Mex). Authors (in alphabetical order): Julio Elias Alvarado-Yaah; Cristóbal Cháidez-Quiróz; Célida Duque Molina; Célida Martínez- Rodríguez; Daniel Fregoso-Rueda; Daniel Lira Morales; Eduardo Becerril-Vargas; Fernando Fontove-Herrera; Fidencio Mejía-Nepomuceno; Francisco Pulido; Gloria Elena Espinosa-Ayala; Gloria María Molina-Salinas; Gloria Vazquez; Hector Esteban Paz-Juárez; Hector Montoya-Fuentes; Helen Haydee Fernanda Ramírez-Plascencia; Irvin González-López; Jean Pierre González; Joel Armando Vázquez-Pérez.; Jorge Salas-Hernández; José Antonio Enciso-Moreno; José Arturo Martínez-Orozco; José Esteban Muñoz-Medina; José de Jesús Nuñez-Contreras; Juan Bautista Chale-Dzul; Julissa Enciso-Ibarra; Luis Alberto Ochoa-Carrera; Margarita Matías-Florentino; Mario Mújica-Sánchez; Marissa Perez-Garcia; María Guadalupe Santiago-Mauricio; María Guadalupe de Jesús Mireles-Rivera; Nelly Sélem-Mojica; Pavel Isa; Ricardo Ciria Merce; Ricardo Grande; Rosa María Gutierrez Rios; Santiago Ávila-Ríos; Selene Zárate; Susana Lopez; Victor Eduardo Garcia-Arias; Víctor Hugo Borja-Aburto
EPI_ISL_3347603, EPI_ISL_3347906	Unidad de Investigación Biomédica de Zacatecas (UIBZ)	Instituto de Biotecnología de la UNAM	; Alejandra García-Gasca; Alejandra Hernández-Terán; Alejandro Sánchez-Flores; Alfredo Herrera-Estrella; Alicia Ocaña-Mondragón; Andreu Comas-García; Angel Gustavo Salas-Lais; Antonio Loza Román; Bernardo Martínez-Miguel; Blanca Taboada; Brenda Irasema Maldonado-Meza; Bruno Gómez-Gil; Carla Ivón Herrera-Najera; Carlos F. Arias; Celia Boukadida; Clara Esperanza Santacruz-Tinoco; Concepción Grajales-Muñiz; Consorcio Mexicano de Vigilancia Genómica (CoViGen-Mex). Authors (in alphabetical order): Julio Elias Alvarado-Yaah; Cristóbal Cháidez-Quiróz; Célida Duque Molina; Célida Martínez- Rodríguez; Daniel Fregoso-Rueda; Daniel Lira Morales; Eduardo Becerril-Vargas; Fernando Fontove-Herrera; Fidencio Mejía-Nepomuceno; Francisco Pulido; Gloria Elena Espinosa-Ayala; Gloria María Molina-Salinas; Gloria Vazquez; Hector Esteban Paz-Juárez; Hector Montoya-Fuentes; Helen Haydee Fernanda Ramírez-Plascencia; Irvin González-López; Jean Pierre González; Jesús Hernández; Joel Armando Vázquez-Pérez).; Jorge Salas-Hernández; José Antonio Enciso-Moreno; José Arturo Martínez-Orozco; José Esteban Muñoz-Medina; José de Jesús Nuñez-Contreras; Juan Bautista Chale-Dzul; Julissa Enciso-Ibarra; Kathia Elizabeth Tapia-Diaz; Luis Alberto Ochoa-Carrera; Margarita Matías-Florentino; Mario Mújica-Sánchez; Marissa Perez-Garcia; María Guadalupe Santiago-Mauricio; María Guadalupe de Jesús Mireles-Rivera; Nelly Sélem-Mojica; Pavel Isa; Ricardo Ciria Merce; Ricardo Grande; Rosa María Gutiérrez Rios; Santiago Ávila-Ríos; Selene Zárate; Susana Lopez; Verónica Mata-Haro; Victor Eduardo García-Arias; Víctor Hugo Borja-Aburto
EPI_ISL_2402120, EPI_ISL_2402124	Unidad de Investigación Biomédica de Zacatecas (UIBZ)	Unidad de Genomica Avanzada	Alejandro Sanchez-Flores; Alfredo Herrera-Estrella; Alicia Ocana-Mondragon; Angel Gustavo Salas-Lais; Bernardo Martinez-Miguel; Blanca Taboada; Brenda Irasema Maldonado-Meza; Carla Ivon Herrera-Najera; Carlos F. Arias; Celia Boukadida; Clara Esperanza Santacruz-Tinoco; Concepcion Grajales-Muniz; Consorcio Mexicano de Vigilancia Genomica (CoViGen-Mex). Authors (in alphabetical order): Julio Elias Alvarado-Yaah; Fernando Fontove-Herrera; Francisco Pulido; Gloria Elena Espinoza-Ayala; Gloria Maria Molina-Salinas; Gloria Vazquez; Hector Esteban Paz-Juarez; Hector Montoya-Fuentes; Helen Haydee Fernanda Ramirez-Plascencia; Jorge Ivan Salinal-Nevarez; Jose Antonio Enciso-Moreno; Jose Esteban Munoz-Medina; Jose de Jesus Nunez-Contreras; Juan Bautista Chale-Dzul; Luis Alberto Ochoa-Carrera; Margarita Matias-Florentino; Maria Guadalupe Santiago-Mauricio; Maria Guadalupe de Jesus Mireles-Rivera; Nelly Selem-Mojica; Pavel Isa; Ricardo Grande; Santiago Avila-Rios; Victor Hugo Borja-Aburto
EPI_ISL_2490368, EPI_ISL_2490474, EPI_ISL_3556968	Unidad de Investigación Médica de Yucatán (UIMY)	Centro de Investigación en Enfermedades Infecciosas (CIENI), Instituto Nacional de Enfermedades Respiratorias (INER)	; Alejandra Garcia-Gasca; Alejandra García-Gasca; Alejandra Hernández-Terán; Alejandro Sanchez-Flores; Alejandro Sánchez-Flores; Alfredo Herrera-Estrella; Alicia Ocaña-Mondragón; Andreu Comas-García; Angel Gustavo Salas-Lais; Antonio Loza Román; Bernardo Martínez-Miguel; Blanca Taboada; Brenda Irasema Maldonado-Meza; Bruno Gomez-Gil; Bruno Gómez-Gil; Carla Ivón Herrera-Najera; Carlos F. Arias; Celia Boukadida; Clara Esperanza Santacruz-Tinoco; Concepción Grajales-Muñiz; Consorcio Mexicano de Vigilancia Genómica (CoViGen-Mex). Authors (in alphabetical order): Julio Elias Alvarado-Yaah; Cristóbal Cháidez-Quiróz; Célida Duque Molina; Célida Martínez- Rodríguez; Daniel Fregoso-Rueda; Daniel Lira Morales; Eduardo Becerril-Vargas; Fernando Fontove-Herrera; Fidencio Mejía-Nepomuceno; Francisco Pulido; Gloria Elena Espinosa-Ayala; Gloria María Molina-Salinas; Gloria Vazquez; Hector Esteban Paz-Juárez; Hector Montoya-Fuentes; Helen Haydee Fernanda Ramírez-Plascencia; Irvin González-López; Jean Pierre González; Jesús Hernández; Joel Armando Vázquez-Pérez.; Jorge Salas-Hernández; José Antonio Enciso-Moreno; José Arturo Martínez-Orozco; José Esteban Muñoz-Medina; José de Jesús Nuñez-Contreras; Juan Bautista Chale-Dzul; Julissa Enciso-Ibarra; Kathia Elizabeth Tapia-Diaz; Luis Alberto Ochoa-Carrera; Margarita Matías-Florentino; Mario Mújica-Sánchez; Marissa Perez-Garcia; María Guadalupe Santiago-Mauricio; María Guadalupe de Jesús Mireles-Rivera; Nelly Sélem-Mojica; Pavel Isa; Ricardo Ciria Merce; Ricardo Grande; Rosa María Gutierrez Rios; Rosa María Gutiérrez Rios; Santiago Ávila-Ríos; Selene Zárate; Susana Lopez; Verónica Mata-Haro; Victor Eduardo Garcia-Arias; Victor Eduardo García-Arias; Víctor Hugo Borja-Aburto
EPI_ISL_2391578, EPI_ISL_2681363	Unidad de Investigación Médica de Yucatán (UIMY)	Instituto de Biotecnología de la UNAM	; Alejandra Garcia-Gasca; Alejandra García-Gasca; Alejandra Hernández-Terán; Alejandro Sanchez-Flores; Alejandro Sánchez-Flores; Alfredo Herrera-Estrella; Alicia Ocaña-Mondragón; Andreu Comas-García; Angel Gustavo Salas-Lais; Antonio Loza Román; Bernardo Martínez-Miguel; Blanca Taboada; Brenda Irasema Maldonado-Meza; Bruno Gomez-Gil; Bruno Gómez-Gil; Carla Ivón Herrera-Najera; Carlos F. Arias; Celia Boukadida; Clara Esperanza Santacruz-Tinoco; Concepción Grajales-Muñiz; Consorcio Mexicano de Vigilancia Genómica (CoViGen-Mex). Authors (in alphabetical order): Julio Elias Alvarado-Yaah; Cristóbal Cháidez-Quiróz; Célida Duque Molina; Célida Martínez- Rodríguez; Daniel Fregoso-Rueda; Daniel Lira Morales; Eduardo Becerril-Vargas; Fernando Fontove-Herrera; Fidencio Mejía-Nepomuceno; Francisco Pulido; Gloria Elena Espinosa-Ayala; Gloria María Molina-Salinas; Gloria Vazquez; Hector Esteban Paz-Juárez; Hector Montoya-Fuentes; Helen Haydee Fernanda Ramírez-Plascencia; Irvin González-López; Jean Pierre González; Jesús Hernández; Joel Armando Vázquez-Pérez.; Jorge Salas-Hernández; José Antonio Enciso-Moreno; José Arturo Martínez-Orozco; José Esteban Muñoz-Medina; José de Jesús Nuñez-Contreras; Juan Bautista Chale-Dzul; Julissa Enciso-Ibarra; Luis Alberto Ochoa-Carrera; Margarita Matías-Florentino; Mario Mújica-Sánchez; Marissa Perez-Garcia; María Guadalupe Santiago-Mauricio; María Guadalupe de Jesús Mireles-Rivera; Nelly Sélem-Mojica; Pavel Isa; Ricardo Ciria Merce; Ricardo Grande; Rosa María Gutierrez Rios; Rosa María Gutiérrez Rios; Santiago Ávila-Ríos; Selene Zárate; Susana Lopez; Verónica Mata-Haro; Victor Eduardo Garcia-Arias; Victor Eduardo García-Arias; Víctor Hugo Borja-Aburto
EPI_ISL_775358	Unilabs Laboratory Medicine	Norwegian Institute of Public Health, Department of Virology	Atiya R Ali; Hilde Elshaug; Hilde Vollan; Kamilla Heddeland Instefjord; Karoline Bragstad; Kathrine Stene-Johansen; Marie Paulsen Madsen; Olav Hungnes; Rasmus Riis Kopperud
EPI_ISL_763000	Unit 17: Influenza & Other Respiratory Viruses, German National Influenza Center	Project group Epidemiology of Highly Pathogenic Microorganisms, Robert Koch-Institute	Andreas Sachse; Ariane Düx; Djin-Ye Oh; Fabian Leendertz; Grit Schubert; Marianne Wedde; Ralf Dürrwald; Sébastien Calvignac-Spencer; Thorsten Wolff
EPI_ISL_3123559, EPI_ISL_3123560, EPI_ISL_3123564, EPI_ISL_3123566	Unit of laboratory surveillance of viral emergent disease Control of Communicable Diseases Departament National Institute of Public Health Albania	Charité Universitätsmedizin Berlin, Institut für Virologie	Barbara Mühlemann; Christian Drosten; Iris Hasibra; Julia Schneider; Jörn Beheim-Schwarzbach; Talitha Veith; Terry Jones; Victor M Corman
EPI_ISL_812310	United States Air Force School of Aerospace Medicine	United States Air Force School of Aerospace Medicine	Amanda Javorina; Anthony Fries; Clarise Starr; Elizabeth Macias; Jennifer Meyer; Sarah Purves; William Gruner
EPI_ISL_2823743	Unity Health Toronto	Ontario Institute for Cancer Research	Bernard Lam; Felicia Vincelli; Ilinca Lungu; Jared T. Simpson; Jeremy Johns; Karel Boissinot; Larissa M. Matukas; Le Luu; Mark Downing; Paul Krzyzanowski; Philip Zuzarte; Ramzi Fattouh; Richard de Borja; Samira Mubareka; TIBDN; Trina Otterman; Yan Chen
EPI_ISL_3023831	Universidad Autonoma de Yucatan	New York Genome Center	Amy Baldwin; Andre Corvelo; Aviles-Gomez E; Ayora-Talavera G; Chan-Gasca M; Dayna M. Oschwald; Flores-Quintal F; Granja-Perez P; Lopez-Coral L.; Michael Zody; Samantha Fennessey; Tom Maniatis; Villanueva-Jorge S; Yam-Pool E
EPI_ISL_3076941	University Clinical Hospital of Mostar, Department of Microbiology and Molecular Diagnostics	University of Sarajevo, Veterinary Faculty, Laboratory for Molecular Diagnostic and Research Laboratory	Goletic S; Goletic T; Hodzic A.; Jazic A.; Nicevic M.; Ostojic M; Sabic E.; Softic A; Terzic I.
EPI_ISL_2895002	University College Hospital, Ibadan	African Centre of Excellence for Genomics of Infectious Diseases (ACEGID), Redeemer's University, Nigeria	A.T.; Abechi; Ajogbasile; Akano; C.A.; C.T.; Eromon; F.V.; Folarin, O.; Fowotade, A.; Happi; I.B.; J.N.; J.U.; K.O.; Kayode; Nosamiefan, I.; Oguzie; Olawoye; Olumade; Oluniyi; P.E.; P.S.; T.J.; Ugwu; Uwanibe
EPI_ISL_3570553	University College London, Great Ormond Street Hospital for Children NHS Foundation Trust, Imperial College Healthcare NHS Trust	COVID-19 Genomics UK (COG-UK) Consortium	Charlotte Williams; Helena Tutill; Judith Breuer; Marius Cotic; Mark Kristiansen; Nadua Bayzid; Patricia Dyal; Rachel Williams; Sergi Castellano; Sunando Roy
EPI_ISL_3739726	University Hospital Brno, OKMI	University Hospital Brno, CMBG	Jan Svaton; Kristyna Dufkova; Martina Lengerova; Matej Bezdicek; Pavlina Volfova
EPI_ISL_2104489, EPI_ISL_2343089	University Hospitals of Geneva, Laboratory of Virology	HUG, Laboratory of Virology and the Health2030 Genome Center	Ana Rita Goncalves; Deborah Penet; Emmanouil Dermitzakis; Henri Pegeot; Ioannis Xenarios; Keith Harshman; Laurent Kaiser; Lorenzo Cerutti; Melyssa Elies; Samuel Cordey
EPI_ISL_671431	University of Debrecen, Department of Medical Microbiology	National Laboratory of Virology, Szentágothai Research Centre	Balázs Somogyi; Brigitta Zana; Endre Gábor Tóth; Eszter Csoma; Ferenc Jakab; Gábor Kemenesi
EPI_ISL_3483805, EPI_ISL_3483878, EPI_ISL_3484042, EPI_ISL_3484089	University of Health Sciences	Quadram Institute Bioscience	Aamir Ashiq; Abdul Sattar; Alexander J Trotter; Alison E. Mather; Almina Shafiq; Alp Aydin; Ana P. Tedim; Anastasia Kolyva; Andrew Bell; Andrew J. Page; Asghar javeed; Claire Stuart; Dave J. Baker; Gemma L. Kay; Hazir Rahman; Javed Akram; John Wain; Justin Grady; Khushbakth kirn; Leonardo de Oliveira Martins; Lizzie Meadows; Maria Diaz; Mark Webber; Muhammad Asad Ali; Muhammad Asif Naveed; Muhammad Bilal Sarwar; Muhammad Nawaz; Muhammad Roman; Muhammd Shahbaz Hussain; Muhammed Yasir; Muhhmad Ilyas; Nabil-Fareed Alikhan; Nadeem Afzal; Nadia Mukhtar; Naila Tariq; Ngozi Elumogo; Nicholas M. Thomson; Qadeer Ahmad; Rachael Stanley; Rachel Gilroy; Reenesh Prakash; Samir Dervisevic; Samuel Bloomfield; Shah Jahan; Sidra-tul-muntaha; Steven Rudder; Tahir Yaqoob; Thanh Le-Viet; Waseem Iqbal; Zahid Latif
EPI_ISL_2224303, EPI_ISL_3758388	University of Mississippi Medical Center, Department of Pathology	University of Mississippi Medical Center, Molecular and Genomics Core Facility	Ashley C. Johnson; D. Ashley Robinson; Ithiel J. Frame; Krishna K. Ayyalasomayajula; Michael R. Garrett; Wenjie Wu
EPI_ISL_955150	University of Sarajevo, Veterinary Faculty, Laboratory for Molecular Diagnostic and Research Laboratory	University of Sarajevo, Veterinary Faculty, Laboratory for Molecular Diagnostic and Research Laboratory	Alić-Šeho A.; Goletić T.; Goletić Š.; Hodžić A.; Jažić A.; Nicević M.; Softić A.; Terzić I.; Šabić E.
EPI_ISL_3099079	University of South Bohemia, Faculty of Science	State Veterinary Institute Prague	Alexander Nagy; Hana Maskova; Jan Sterba; Jindrich Chmelar; Lenka Cernikova; Martina Stara
EPI_ISL_476078, EPI_ISL_677760	University of Szeged, Institute of Clinical Microbiology	National Laboratory of Virology, Szentágothai Research Centre	Balázs Somogyi; Brigitta; Brigitta Zana; Endre Gábor Tóth; Ferenc Jakab; Gabriella Terhes; Gábor Kemenesi; Terhes Gabriella
EPI_ISL_3825375	University of Wisconsin-Madison AIDS Vaccine Research Laboratories	University of Wisconsin-Madison AIDS Vaccine Research Laboratories	Gage Moreno; Katarina Braun; et al. AIDS Vaccine Research Laboratories
EPI_ISL_977357, EPI_ISL_977379, EPI_ISL_977411, EPI_ISL_977469, EPI_ISL_2803586, EPI_ISL_2803663, EPI_ISL_2803712, EPI_ISL_2803718, EPI_ISL_2803721, EPI_ISL_2803734			
see above	University of Zambia, School of Veterinary Medicine	UNZAVET and PATH	Daniel Bridges; Mulenga Mwenda-Chimfwembe; Ngonda Saasa; ZNPHI and ZGSC

EPI_ISL_1692773	Università Federico II - Dipartimento di scienze mediche traslazionali - Napoli	Telethon Institute of Genetics and Medicine (TIGEM)	Antonio Grimaldi Patrizia Annunziata Francesco Panariello Teresa Giuliano Michele Cennamo Valentina Bouche Chiara Colantuono Lucio Di Filippo Mariano Fiorenza Anna Manfredi Marcello Salvi Giuseppe Portella Andrea Ballabio Davide Cacchiarelli
EPI_ISL_3546572	Universität Innsbruck, Institut für Mikrobiologie	Bergthaler laboratory, CeMM Research Center for Molecular Medicine of the Austrian Academy of Sciences	Andreas Bergthaler; Anna Schedl; Bekir Erguner; Benedikt Agerer; Christoph Bock; Fabian Amman; Jan Laine; Lukas Endler; Maelle Le Moing; Martin Senekowitsch; Matthew Thornton; Michael Schuster; Petr Triska; Thomas Penz
EPI_ISL_3667037	Uniwersyteckie Centrum Kliniczne	WSSEw Warszawie	Dorota Wągrocka - Roczniak
EPI_ISL_513699, EPI_ISL_2291699, EPI_ISL_3263632, EPI_ISL_3833149	Utah Public Health Laboratory	Utah Public Health Laboratory	Erin L. Young; Erin Young; Heidi Butz; Kelly F. Oakeson; Kelly Oakeson; Olinto Linares-Perdomo; Pooja Gupta; Tara Gallagher
EPI_ISL_3045997, EPI_ISL_3046033	VIDOH	Grubaugh Lab - Yale School of Public Health	Anderson Brito; Annie Watkins; Brett Ellis; Chaney Kalinich; Chantal Vogels; Esther Ellis; Isabel Ott; Jendai Richards; Jessica Rothman; Joseph Fauver; Kendall Billig; Mallery Breban; Marlon Lawrence; Mary Petrone; Nathan Grubaugh; TaLesa Aderohunmu; Tara Alpert; Tobias Koch
EPI_ISL_3347344, EPI_ISL_3841007	VIDOH (Virgin Islands Department of Health)	Grubaugh Lab - Yale School of Public Health	Anderson Brito; Annie Watkins; Brett Ellis; Chaney Kalinich; Chantal Vogels; Esther Ellis; Isabel Ott; Jendai Richards; Jessica Rothman; Joseph Fauver; Kendall Billig; Mallery Breban; Marlon Lawrence; Mary Petrone; Nathan Grubaugh; TaLesa Aderohunmu; Tara Alpert; Tobias Koch
EPI_ISL_1095188	VT Dept. of Health Laboratory	Respiratory Viruses Branch, Division of Viral Diseases, Centers for Disease Control and Prevention	Anna Montmayeur; Anna Uehara; Ben L. Rambo-Martin; Clinton R. Paden; Dhwani Batra; Haibin Wang; Jasmine Padilla; Jing Zhang; Justin Lee; Krista Queen; Lori Rowe; Mark Burroughs; Mili Sheth; Peter W. Cook; Rachel Marine; Sarah Nobles; Suxiang Tong; Yan Li; Ying Tao
EPI_ISL_3725871	VUMC Molecular Infectious Diseases Laboratory (MIDL)	Dr. Suman Das Lab - Vanderbilt University Medical Center (VUMC) (https://my.vanderbilt.edu/daslab/)	Bookyung Park; Grant Vestal; Helen Boone; Hunter Brown; Jonathan E. Schmitz; Meghan Shilts; Seesandra Rajagopala; Suman B. Pakala; Suman Das
EPI_ISL_2360475	Vaccines and Infectious Diseases Analytics Research Unit (VIDA)	KRISP, KZn Research Innovation and Sequencing Platform	Baillie Vicky; Giandhari Jennifer; Madhi Shabir; Naidoo Yeshnee; Pillay Sureshnee; San James; Tegally Houriiyah; Wilkinson Eduan; de Oliveira Tulio; du Plessis Jeanine
EPI_ISL_2425984	Vestfold Hospital, Toensberg Department of Microbiology	Norwegian Institute of Public Health, Department of Virology	Atiya R Ali; Debech Nadia; Engebretsen Serina Beate; Garcia Llorente Ignacio; Hilde Elshaug; Hilde Vollan; Jon Bråte; Kamilla Heddeland Instefjord; Karoline Bragstad; Kathrine Stene-Johansen; Line Victoria Moen; Marie Paulsen Madsen; Olav Hungnes; Pedersen Benedikte Nevjen; Rasmus Riis Kopperud
EPI_ISL_3259626	Victor Babes Hospital	National Institute of Infectious Diseases-Prof. Dr. Matei Bals Molecular Diagnostics Laboratory	Corina Casangiu; Dan Otelea; Leontina Banica; Marius Surleac; Ovidiu Vlaicu; Petre Milu; Robert Hohan; Simona Paraschiv
EPI_ISL_426723	Victorian Infectious Diseases Reference Laboratory (VIDRL)	Microbiological Diagnostic Unit Public Health Laboratory and Victorian Infectious Diseases Reference Laboratory, Doherty Institute	Caly L.; Druce J.; Sait, M.; Schultz M.; Seemann T.; Sherry, N.
EPI_ISL_480637, EPI_ISL_591890, EPI_ISL_779632, EPI_ISL_1913212, EPI_ISL_2920935, EPI_ISL_2920951, EPI_ISL_2920973, EPI_ISL_2920997, EPI_ISL_2978349, EPI_ISL_3134919, EPI_ISL_3134920			
see above	Victorian Infectious Diseases Reference Laboratory (VIDRL)	VIDRL and MDU-PHL	Caly L.; Druce J.; M. B.; M.L.; N.L.; Sait; Sait, M.; Schultz; Schultz M.; Seemann T.; Sherry; Sherry, N.
EPI_ISL_416411, EPI_ISL_419734	Victorian Infectious Diseases Reference Laboratory (VIDRL)	Victorian Infectious Diseases Reference Laboratory and Microbiological Diagnostic Unit Public Health Laboratory, Doherty Institute	Caly L.; Druce J.; Sait, M.; Schultz M.; Seemann T.; Sherry, N.; Taiaroa, G.
EPI_ISL_2694098	Viesoji istaiga Klaipedos universitetine ligonine	National Public Health Surveillance Laboratory	Ana Steponkiene; Danas Baksa; Jelena Razmuk; Lukas Vasionis; Lukas Zemaitis; Migle Gabrielaite; Svajune Muralyte
EPI_ISL_3863396	Viesoji istaiga Respublikine Siauliu ligonine	National Public Health Surveillance Laboratory	Ana Steponkiene; Danas Baksa; Jelena Razmuk; Lukas Vasionis; Lukas Zemaitis; Migle Gabrielaite; Svajune Muralyte
EPI_ISL_3711353	Viesoji istaiga Vilniaus universiteto ligonine Santaros klinikos	National Public Health Surveillance Laboratory	Ana Steponkiene; Danas Baksa; Jelena Razmuk; Lukas Vasionis; Lukas Zemaitis; Migle Gabrielaite; Svajune Muralyte
EPI_ISL_2339764, EPI_ISL_2428847, EPI_ISL_2510745	Viesoji istaiga Vilniaus universiteto ligonine Santaros klinikos	Vilnius University Hospital Santaros Klinikos, Center of Laboratory Medicine	Daniel Naumovas; Dovile Ezerskyte; Gytis Dudas; Ingrida Olendraite; Laimonas Griskevicius; Ligita Raugaite; Mindaugas Stoskus; Monika Katenaite; Rimvydas Norvilas
EPI_ISL_3341256	Viešoji įstaiga Vilniaus universiteto ligoninė Santaros klinikos	National Public Health Surveillance Laboratory	Ana Steponkiene; Danas Baksa; Jelena Razmuk; Lukas Vasionis; Lukas Zemaitis; Migle Gabrielaite; Svajune Muralyte
EPI_ISL_933829	Vilnius university hospital Santaros Klinikos, Center of Laboratory Medicine	Vilnius university hospital Santaros Klinikos, Center of Laboratory Medicine	Daniel Naumovas; Dovile Ezerskyte; Gytis Dudas; Ingrida Olendraite; Justinas Slikas; Rimvydas Norvilas
EPI_ISL_1913959	Viollier AG	Department of Biosystems Science and Engineering, ETH Zurich	Chaoran Chen; Christiane Beckmann; Christoph Noppen; David Dreifuss; Deborah Penet; Emmanouil Dermitzakis; Henri Pegeot; Ioannis Xenarios; Ivan Topolsky; Katharina Jahn; Keith Harshman; Lara Fuhrmann; Lorenzo Cerutti; Maurice Redondo; Niko Beerenwinkel; Noemie Santamaria de Souza; Olivier Kobel; Philipp Jablonski; Sarah Nadeau; Sophie Seidel; Tanja Stadler
EPI_ISL_466959, EPI_ISL_1001477, EPI_ISL_1002431, EPI_ISL_1260458, EPI_ISL_2212174, EPI_ISL_2212240, EPI_ISL_2462597, EPI_ISL_2544318, EPI_ISL_2611096, EPI_ISL_2938432, EPI_ISL_3295897, EPI_ISL_3297505, EPI_ISL_3853912			
see above	Viollier AG	Department of Biosystems Science and Engineering, ETH Zürich	Andrea Patrignani; Andreia Cabral de Gouvea; Catharine Aquino; Chaoran Chen; Christian Beisel; Christiane Beckmann; Christoph Noppen; Daniel Ehrsam; David Dreifuss; Doris Popovic; Elodie Burcklen; Griffin White; Ina Nissen; Isabel Stürmer; Ivan Topolsky; Jay Tracy; Katharina Jahn; Kim Philipp Jablonski; Lara Fuhrmann; Laura Neff; Lennart Opitz; Louis du Plessis; Maria Domenica Moccia; Maurice Redondo; Mirjam Feldkamp; Natascha Santacroce; Niko Beerenwinkel; Noemie Santamaria de Souza; Olivier Kobel; Pedro Ferreira; Philipp Jablonski; Ralph Schlapbach; Rebecca Denes; Sarah Nadeau; Simon Grüter; Sophie Seidel; Susana Posada-Céspedes; Tanja Stadler; Timothy Sykes; Tobias Schär
EPI_ISL_2017861	Viral Respiratory Infections Laboratory, Cantacuzino National Military-Medical Institute	Cantacuzino Institute Virology	Catalina Pascu; Luiza Ustea; Mihaela Lazar; Mihaela Oprea; Nicoleta Paraschiv; Sorin Dinu
EPI_ISL_420851, EPI_ISL_2135840, EPI_ISL_2135845, EPI_ISL_2966635, EPI_ISL_2966637, EPI_ISL_2966640, EPI_ISL_2966644, EPI_ISL_2966653, EPI_ISL_2966656, EPI_ISL_2968556, EPI_ISL_2968689, EPI_ISL_2968726, EPI_ISL_3086912, EPI_ISL_3086923, EPI_ISL_3086927, EPI_ISL_3086928			
see above	Viral Respiratory Lab, National Institute for Biomedical Research (INRB)	Pathogen Sequencing Lab, National Institute for Biomedical Research (INRB)	Allison Black; Amuri Aziza; Andrew Rambaut; Catherine Pratt; Eddy Kinganda-Lusamaki; Edith Nkwembe; Emmanuel Lokilo Lofiko; Francisca Muyembe Mawete; Gabriel Kabamba; Ian Goodfellow; James Hadfield; Jean Claude Makangara; Jean-Jacques Muyembe Tamfum; Josh Quick; Kristian Andersen; Matthias Pauthner; Michael Wiley; Nick Loman; Placide Mbala-Kingebeni; Raphaël Lumembe; Steve Ahuka-Mundeke; Trevor Bedford
EPI_ISL_2920804	Virginia Division of Consolidated Laboratory Services	Virginia Division of Consolidated Laboratory Services	Virginia Division of Consolidated Laboratory Services
EPI_ISL_914842	Virginia Division of Consolidated Laboratory Services (DCLS)	Virginia Division of Consolidated Laboratory Services (DCLS)	Virginia DCLS
EPI_ISL_2834930, EPI_ISL_2834932	Virology Department, Central Health Laboratory	Central Health Laboratory ,Victoria Hospital, Candos,Ministry of Health and Wellness, Mauritius	Bahadoor BS; Jannoo N; Manraj SS; Mathur H; Ramuth M; Sonoo J; Sujeewon C
EPI_ISL_2499903, EPI_ISL_2499914, EPI_ISL_2657332, EPI_ISL_2657343, EPI_ISL_2657346	Virology Department, Central Health Laboratory	The Francis Crick Institute	Bahadoor BS; Crawford M; Daniels RS; Goldstone R; Harvey R; Manraj SS; Nicod J; Patel H; Ramuth M; Sonoo J
EPI_ISL_3231384	Virology Department,Central Health Laboratory	UMR PIMIT	Bahadoor BS; David Wilkinson; Manraj SS; Patrick Mavingui; Ramuth M; Sonoo J
EPI_ISL_3839615, EPI_ISL_3839635, EPI_ISL_3839644, EPI_ISL_3839657	Virology Department,Central Health Laboratory	Virology Department,Central Health Laboratory	Bahadoor BS; Jannoo N; Manraj SS; Mathur H; Ramuth M; Sonoo J; Sujeewon C
EPI_ISL_1123281	Virology Laboratory of Praia	Institut Pasteur de Dakar	Dia Ndongo; Diagne Moussa Moïse; Diallo Amadou; Diop Mamadou; Faye Ousmane; Kevin Sanders; Loucoubar Cheikh; Mbengue Safietou Sankhe; Ndiaye Ndack; Sall Amadou Alpha; Tordo Noel
EPI_ISL_3506353	Virology Laboratory, International Centre for Diarrhoeal Disease Research, Bangladesh (ICDDR,B)	Virology Laboratory, International Centre for Diarrhoeal Disease Research, Bangladesh (ICDDR,B)	Md. Mahfuzur Rahman; Mohammad Enayet Hossain; Mohammed Ziaur Rahman; Mojnu Miah; Mustafizur Rahman
EPI_ISL_1855000	Virology Laboratory, Scientific Department, Army Medical Center	Virology Laboratory, Scientific Department, Army Medical Center	Anella Monte; Anna Anselmo; Antonella Fortunato; Filippo Molinari; Florigio Lista; Francesco Giordani; Giancarlo Petralito; Giandomenico Cerreto; Riccardo De Sanctis; Silvia Fillo; Vanessa Vera Fain
EPI_ISL_677634, EPI_ISL_1660265, EPI_ISL_1660267, EPI_ISL_1660283, EPI_ISL_1660290, EPI_ISL_1660310	Virology Unit, Institut Pasteur de Madagascar	Virology Unit, Institut Pasteur de Madagascar	Angela Brisebarre; Camille Capel; Cara E. Brook; Christian Ranaivoson; Christophe Malabat; Corinne Maufrais; Cristina M. Tato; Emmanuelle Permal; Etienne Simon-Lorière; Frédéric Lemoine; Helisoa Razafimanjato; Jean-Michel Heraud; Joseph L. DeRisi; Louise Lefrançois; Marion Barbet; Maud Vanpeene; Michelle Tan; Méline Bizard; Norosoa Razanajatovo; Philippe Dussart; Soa Fy Andriamandimby; Sylvie Behillil; Sylvie van der Werf; Tsiry Randriambolamanantsoa; Vida Ahyong; Vincent Enouf; Vololoniaina Raharinosy
EPI_ISL_1532805, EPI_ISL_1532815, EPI_ISL_1706602, EPI_ISL_1711980, EPI_ISL_1969684, EPI_ISL_2106247, EPI_ISL_2106262, EPI_ISL_2231577, EPI_ISL_2406476, EPI_ISL_2693927, EPI_ISL_3045595, EPI_ISL_3387384, EPI_ISL_3570901			
see above	Virology Unit, Institut Pasteur du Cambodge	Virology Unit, Institut Pasteur du Cambodge	Cecile Troupin; Chau Darapheak; Chin Savuth; Erik A Karlsson; Jurre Y Siegers; Kraing Sidonn; Leakhena Pum; Ly Sovann; Sokhoun Yann; Sophoannadedh Rath; Teyputita Ou; Veasna Duong; Yi Sengdoeurn
EPI_ISL_411902	Virology Unit, Institut Pasteur du Cambodge.	Virology Unit, Institut Pasteur du Cambodge (Sequencing done by: Jessica E Manning/Jennifer A Bohl at Malaria and Vector Research Research Laboratory, National Institute of Allergy and Infectious Diseases and Vida Ahyong from Chan-	Erik A Karlsson; Jennifer A Bohl; Jessica E Manning.; Philippe Dussart; Veasna Duong; Vida Ahyong

		Zuckerberg Biohub)	
EPI_ISL_2140348	Vitomed Sp. z o.o.	1. Tricity SARS-CoV-2 sequencing consortium: University of Gdansk, Medical University of Gdansk, Vaxican Ltd., Invicta Ltd. 2. National Institute of Public Health - National Institute of Hygiene, Warsaw, Poland	Celina Cybulska; Karolina Gackowska; Katarzyna Groth; Katarzyna Zacharczuk; Krystyna Bienkowska Szewczyk; Lukasz Rabalski; Maciej Grzybek; Maciej Kosinski; Magdalena Nowakowska; Małgorzata Sadkowska-Todys; Tomasz Wołkowicz
EPI_ISL_3867292, EPI_ISL_3867300, EPI_ISL_3867303, EPI_ISL_3867310, EPI_ISL_3867311, EPI_ISL_3867312	WACCBIP, University of Ghana	WACCBIP, University of Ghana	Abdul-Karim Abass; Bright K. Yemi; Collins M. Morang'a; Deborah N. A. Mettle; Dennis Adu-Gyasi; Dominic S. Y. Amuzu; Edward Danso Fenteng; Emmanuel Kudjo; Emmanuella Amoako; Evelyn B. Quansah; Frederick Tei-Maya; Israel Osei-Wusu; Joe K. Mutungi; Joyce M. Ngoi; Kwado Poku Asante; Lawrence Ofori-Boadu; Lucas N. Amenga-Etego; Michael Owus; Nicaise T. Ndam; Oliver Commey; Oliver D. Boakye; Patrick Tetteh Ababio; Paul Owusu-Oduro; Peter K. Quashie; Philip M. Soglo; Richard Odame Phillips; Samirah Saiid; Samuel Armoo; Samuel Kaba Akoriyea; Sylvester Dassah; Theophilus Odoom; Victor Asoala; Vincent Appiah; Violette V. Mâ€™cormack; William K. Ampofo; Yaw Bediako; and Gordon A. Awandare
EPI_ISL_2836897, EPI_ISL_2836916, EPI_ISL_2836918, EPI_ISL_2836953, EPI_ISL_2968009, EPI_ISL_2968012, EPI_ISL_3268047, EPI_ISL_3268050, EPI_ISL_3268100, EPI_ISL_3268122, EPI_ISL_3268124			
see above	WACCBIP, University of Ghana, Accra, Ghana	WACCBIP, University of Ghana, Volta Road, Legon-Accra, Ghana	; Bright K. Yemi; Collins M. Morang'a; Deborah N. A. Mettle; Dominic S. Y. Amuzu; Dominic S.Y. Amuzu; Evelyn B. Quansah; Evelyn Y. Bonney; Frederick M. Tei-Maya; Frederick Tei-Maya; Israel Osei-Wusu; Ivy A. Asante; Joe K. Mutungi; John K. Odoom; Joseph H.K. Bonney; Joyce M. Ngoi; Lawrence Ofori-Boadu; Lucas N. Amenga-Etego; Lucas N. Amenga-Etego and Gordon A. Awandare; Michael Owusu; Mildred Adusei-Poku; Nicaise T. Ndam; Oliver Commey; Paul Owusu-Oduro; Peter K Quashie; Peter K. Quashie; Philip M. Soglo; Richard Odame Phillips; Samirah Saiid; Samuel Armoo; Sylvester Dassah; Victor Asoala; Vincent Appiah; Violette V. M'cormack; Violette V. M’cormack; William K. Ampofo; Yaw Bediako; and Gordon A. Awandare
EPI_ISL_3268105	WACCBIP, University of Ghana, Volta Road, Legon-Accra, Ghana	WACCBIP, University of Ghana, Volta Road, Legon-Accra, Ghana	; Bright K. Yemi; Collins M. Morang'a; Deborah N. A. Mettle; Dominic S. Y. Amuzu; Evelyn B. Quansah; Frederick M. Tei-Maya; Israel Osei-Wusu; Joe K. Mutungi; Joyce M. Ngoi; Lucas N. Amenga-Etego; Michael Owusu; Nicaise T. Ndam; Oliver Commey; Paul Owusu-Oduro; Peter K. Quashie; Philip M. Soglo; Richard Odame Phillips; Samirah Saiid; Samuel Armoo; Sylvester Dassah; Victor Asoala; Vincent Appiah; Violette V. M’cormack; William K. Ampofo; Yaw Bediako; and Gordon A. Awandare
EPI_ISL_2385177, EPI_ISL_2816183, EPI_ISL_3454793, EPI_ISL_3454797, EPI_ISL_3454801	WHO National Influenza Centre Russian Federation	WHO National Influenza Centre Russian Federation	Alexey Masharsky; Andrey Komissarov; Artem Fadeev; Daria Danilenko; Dmitry Lioznov; Elena Nabieva; Georgii Bazykin; Kirill Varchenko; Ksenia Safina; Kseniya Komissarova; Maria Baturova; Maria Pisareva; Maria Timofeeva; Mikhail Bakaev; Nikita Yolshin; Oksana Stanevich; Oula Mansour; Oula Masour; Tamila Musaeva; Veronika Eder
EPI_ISL_3446540, EPI_ISL_3446541	WHO/Country Office in the Republic of Moldova	Charité Universitätsmedizin Berlin, Institut für Virologie	Ala Halacu; Barbara Mühlemann; Christian Drosten; Julia Schneider; Jörn Beheim-Schwarzbach; Mariana Apostol; Talitha Veith; Terry Jones; Victor M Corman
EPI_ISL_3838171	WSSE Katowice	Wojewódzka Stacja Sanitarno-Epidemiologiczna w Katowicach	Beata Rozwadowska
EPI_ISL_754811	Wadsworth Center, New York State Department.of Health	Wadsworth Center, New York State Department.of Health	Alexis Russel; Daryl M. Lamson; Erasmus Schneider; Erica Lasek-Nesselquist; John Kelly; Jonathan Plitnick; Kirsten St. George; Matthew Shudt; Melissa A Leisner; Navjot Singh; Sara Griesemer
EPI_ISL_456193, EPI_ISL_548102, EPI_ISL_649124, EPI_ISL_2103200, EPI_ISL_2964929	Waikato Hospital	Institute of Environmental Science and Research (ESR)	Anja Werno; Antje van der Linden; Arlo Upton; Chris Mansell; David Hammer; Dragana Drinkovic; Erasmus Smit; Gary McAuliffe; Hana Sofia Andersson; Hermes Perez; James Ussher; Jill Sherwood; Jing Wang; Joep de Ligt; Josh Freeman; Julia Howard; Juliet Elvy; Lauren Jelly; Mary DeAlmeida; Matt Blakiston; Matt Storey; Matthew Rogers; Max Bloomfield; Michael Addidle; Michelle Balm; Muhammad Faisal; Nikki Freed; Olin Silander; Olivia Stroeven; Rachel Boyle; Sally Roberts; SallyAnn Harbison; Sarah Jefferies; Sharmini Muttaiyah; Susan Morpeth; Susan Taylor; Timothy Blackmore; Vani Sathyendran; Veronica Playle; Virginia Hope; Xiaoyun Ren
EPI_ISL_3656831	Washington State Department of Health Public Health Laboratories	Washington State Department of Health Public Health Laboratories	Avi Singh; Darren Lucas; Denny Russell; Drew MacKellar; Geoff Melly; Hannah Gray; Joenice Gonzalez; JohnAric Peterson; Philip Dykema; Rebecca Cao; Vanessa De Los Santos
EPI_ISL_456165, EPI_ISL_456188, EPI_ISL_456334	Wellington SCL	Institute of Environmental Science and Research (ESR)	Anja Werno; Antje van der Linden; Arlo Upton; Chris Mansell; David Hammer; Dragana Drinkovic; Erasmus Smit; Gary McAuliffe; Hana Sofia Andersson; James Ussher; Jill Sherwood; Joep de Ligt; Josh Freeman; Julia Howard; Juliet Elvy; Lauren Jelly; Mary DeAlmeida; Matt Blakiston; Matt Storey; Matthew Rogers; Max Bloomfield; Michael Addidle; Michelle Balm; Sally Roberts; Sarah Jefferies; Sharmini Muttaiyah; Susan Morpeth; Susan Taylor; Timothy Blackmore; Vani Sathyendran; Veronica Playle; Virginia Hope; Xiaoyun Ren
EPI_ISL_649125, EPI_ISL_1016878, EPI_ISL_1250695, EPI_ISL_2964931, EPI_ISL_2964938, EPI_ISL_2964939, EPI_ISL_2964940, EPI_ISL_3164093, EPI_ISL_3164098, EPI_ISL_3164099, EPI_ISL_3164102, EPI_ISL_3164103, EPI_ISL_3164104, EPI_ISL_3164106, EPI_ISL_3164109, EPI_ISL_3664455, EPI_ISL_3664456, EPI_ISL_3709185, EPI_ISL_3709188, EPI_ISL_3709190, EPI_ISL_3709192, EPI_ISL_3709194, EPI_ISL_3709200			
see above	Wellington SCL (WN)	Institute of Environmental Science and Research (ESR)	Anja Werno; Antje van der Linden; Arlo Upton; Chris Mansell; David Hammer; Dragana Drinkovic; Erasmus Smit; Gary McAuliffe; Hana Sofia Andersson; Hermes Perez; James Ussher; Jill Sherwood; Jing Wang; Joep de Ligt; Josh Freeman; Julia Howard; Juliet Elvy; Lauren Jelly; Mary DeAlmeida; Matt Blakiston; Matt Storey; Matthew Rogers; Max Bloomfield; Michael Addidle; Michelle Balm; Muhammad Faisal; Nikki Freed; Olin Silander; Olivia Stroeven; Rachel Boyle; Sally Roberts; SallyAnn Harbison; Sarah Jefferies; Sharmini Muttaiyah; Susan Morpeth; Susan Taylor; Timothy Blackmore; Vani Sathyendran; Veronica Playle; Virginia Hope; Xiaoyun Ren
EPI_ISL_1255176, EPI_ISL_1255251	West African Centre for Cell Biology of Infectious Pathogens (WACCBIP), University of Ghana, Accra, Ghana	West African Centre for Cell Biology of Infectious Pathogens (WACCBIP), University of Ghana, Volta Road, Legon-Accra, Ghana	; Abdoulaye B Diallo; Abdul-Karim Abass; Aisha Mohammed; Benjamin Demah Nuertey; Collins M. Morang'a; Dam Kenneth Mibut; Dominic S.Y. Amuzu; Emmanuella Amoako4; Evelyn B. Quansah; Frederick Kumi-Ansah; Frederick Tei-Maya; Gordon A Awandare; Joyce M. Ngoi; Kesego Tapela; Lucas N. Amenga-Etego; Nelson Kibinge; Oliver D Boakye5; Peter K Quashie; Philip M. Soglo; Samirah Saiid; Samuel Kaba Akoriyea; Theophilus Odoom; Vanessa Magnussen; Vincent Appiah; Yaw Bediako
EPI_ISL_3091525	Wexner Medical Center	The Ohio State University College of Medicine	Aidja, Z.; Koenig, S.
EPI_ISL_1921876	Wichita State University - Molecular Diagnostics Lab	Kansas Health and Environmental Lab	Ben Olsen; Jonathan Barnell; Mike Grose; and Phil Adam
EPI_ISL_2833939	Wichita State University- Molecular Diagnostic Lab	Kansas Health and Environmental Lab	Ben Olsen; Jonathan Barnell; Katherine Wiggins; Mike Grose; and Phil Adam
EPI_ISL_3398275	Willis-Knighton Medical Center Hospital Laboratory	LSUHS Emerging Viral Threat Laboratory	Alexander Mijalis; Andrew D. Yurochko; April N. Johnson; Christopher G. Kevil; Gregory L. Ware; Jennifer L. Carroll; Jeremy P. Kamil; John A. Vanchiere; Joseph A. Bocchini; Lorie M. Atkins; Maarten Van Diest; Rona S. Scott
EPI_ISL_3131926	Wisconsin State Laboratory of Hygiene Communicable Disease Division	Wisconsin State Laboratory of Hygiene Communicable Disease Division	Abigail C. Shockey; Alicia J. Mooney; Erika M. Hanson; Kelsey R. Florek; Richard Griesser; Sara Wagner; Tonya Danz
EPI_ISL_2142039	Wojewódzki Specjalistyczny Szpital im. dr Wł. Biegańskiego	1. National Institute of Public Health - National Institute of Hygiene; 2. Eurofins Genomics Europe Sequencing GmbH	ECDC COVID-19 WGS support team; Eurofins Genomics Europe Sequencing Team; Gierczyńki Rafał; Sadkowska-Todys Małgorzata; Wołkowicz Tomasz; Zacharczuk Katarzyna
EPI_ISL_402130	Wuhan Jinyintan Hospital	Wuhan Institute of Virology, Chinese Academy of Sciences	Ding-Yu Zhang; Hao-Rui Si; Lei Zhang; Peng Zhou; Xing-Lou Yang; Yan Zhu; Zhengli Shi
EPI_ISL_1335800, EPI_ISL_2226826, EPI_ISL_2247990, EPI_ISL_3062475, EPI_ISL_3751174, EPI_ISL_3758804	Wyoming Public Health Laboratory	Wyoming Public Health Laboratory	Ashley Norberg; Brian Dominguez; Brittany Oher; Cari Sloma; Channing Weber; Chayse Rowley; Elliot Thomasson; Jim Mildenberger; Lynette Gumbleton; Marley Goetz; Noah Hull; Robert Petit; Sam Britz; Taylor Fearing; Wanda Manley; and Rob Christensen
EPI_ISL_2159597, EPI_ISL_2296466	Yale Clinical Virology Lab	Grubaugh Lab - Yale School of Public Health	Anderson Brito; Annie Watkins; Chaney Kalinich; Chantal Vogels; Isabel Ott; Jessica Rothman; Joseph Fauver; Mallery Breban; Marie L. Landry; Mary Petrone; Nathan Grubaugh; Tara Alpert
EPI_ISL_3188967, EPI_ISL_3188998	ZANGIATA INFECTIOUS CLINICAL HOSPITAL №2	Biotechnology laboratory, Center for advanced technology	Abror Abdurakhimov; Alisher Abdullaev; Dilbar Dalimova; Diyora Dalimova; Elena Tsay; Gul Esonova; Ibragimova Shahnoza; Shakhlo Turdikulova; Sharof Nuriddinov; Vladimir Tsoy; Zebinisa Mirakbarova
EPI_ISL_3098925	ZOL	Jessa	Berden et al. on behalf of the Jessa_cmdLab
EPI_ISL_666626, EPI_ISL_3210091	ZOTZ KLIMAS MVZ Düsseldorf-Centrum GbR ÜBAG für Labormedizin, Genetik, Zytologie, Pathologie	Center of Medical Microbiology, Virology, and Hospital Hygiene, University of Duesseldorf	Alexander Dilthey; Andreas Walker; Ashley-Jane Duplessis; Daniel Strelow; Jessica Nicolai; Jörg Timm; Katrin Hoffmann; Klaus Pfeffer; Lisanna Hülse; Malte Kohns Vasconcelos; Marek Korencak; Maximilian Damagnez; Nadine Lübke; Patrick Finzer; Rainer Zotz; Tobias Wienemann; Torsten Houwaart
EPI_ISL_2726784	Zakład Mikrobiologii Klinicznej WSZ im dr R. Ostrzyckiego w Koninie	1. National Institute of Public Health - National Institute of Hygiene, Warsaw, Poland 2. Biobank Lab, University of Lodz 3. Laboratory of Respiratory Viruses, Teaching and Clinical Center of the Medical University of Lodz	Dominik Strapagiel; Izabela Dróżdż; Jakub Lach; Katarzyna Zacharczuk; Klaudyna Królikowska; Maciej Borowiec; Magdalena Nowakowska; Magdalena Traczyk-Borszyńska; Marcin Słomka; Marta Sobalska-Kwapis; Małgorzata Sadkowska-Todys; Tomasz Płoszaj; Tomasz Wołkowicz
EPI_ISL_2859301	Zamboanga City Medical Center GeneXpert Laboratory	Philippine Genome Center	Alethea R. de Guzman; Anna Ong-Lim; Arianne A. Zamora; Benedict A. Maralit; Carlo M. Lapid; Celia Carlos; Devon Ray Pacial; Diomedes A. Carino; Edsel Maurice Salvaña; El King D. Morado; Elcid Aaron R. Pangilinan; Eva Maria Cutiongco-de la Paz; Francis A. Tablizo; Henrietta Marie Rodriguez; Jaime C. Montoya; Jan Michael C. Yap; Jarvin E. Nipales; Jo-Hannah S. Llames; John Q. Wong; Joshua Gregor A. Dizon; Juan Antonio R. Magalang; Karol Sophia Agape R. Padilla; Kenneth M. Kim; Kris P. Punayan; Krisitna Patriz Dela Cruz; Lindsay Claire D.L. Carandang; Ma. Exanil Plantig; Marc Edsel C. Ayes; Maria Rosario Singh-Vergeire and Cynthia P. Saloma; Maria Sofia L. Yangzon; Marielle M Gamboa; Marissa Alejandria; Nina Francesca Bustamante; Razel Nikka M. Hao; Renato Jacinto Q. Mantaring; Rianna Patricia S. Cruz; Sheila Mae M. Araiza; Yvonne Valerie Austria; Zipporah Mariebelle R. Enriquez; Zyrel V. Mollejon
EPI_ISL_3739792, EPI_ISL_3739828	Zavod za javno zdravstvo Koprivničko-križevačke županije	Hrvatski zavod za javno zdravstvo	Irena Tabain; Ivana Ferenčak
EPI_ISL_2674054, EPI_ISL_3046416	Zavod za javno zdravstvo Međimurske Županije	Hrvatski zavod za javno zdravstvo	Irena Tabain; Ivana Ferenčak
EPI_ISL_3620769	Zavod za javno zdravstvo Zagrebačke Županije	Hrvatski zavod za javno zdravstvo	Irena Tabain; Ivana Ferenčak
EPI_ISL_2674389, EPI_ISL_2674390, EPI_ISL_2693542	Zavod za javno zdravstvo Šibensko- Kninske Županije	Hrvatski zavod za javno zdravstvo	Irena Tabain; Ivana Ferenčak
EPI_ISL_3506278	Zdravotní ústav Ústí nad Labem	Institute of Medical Microbiology and Virology,	Alexa Laubner; Alexander Dalpke; Anett Zabzinski; Eva Patrasová; Fabian Rost; Grit Mehnert; Ivana Stiborová; Jitka Pohořská; Johanna Beil; Lenka Šimůnková; Leo Büttner; Marlena Stadtmüller; Montserrat Palau de Miquel; Romana Mikešová; Susanne Reinhardt; Sylke Winkler; Sylvia

		University Hospital Carl Gustav Carus, TU Dresden	Klemroth
EPI_ISL_3855348	Zentrallabor Zürich	Institute of Medical Virology	Alexandra Trkola; Annette Audigé; Catharine Aquino; Cyril Shah; Daniel Ehrsam; Gabriela Ziltener; Guido Bloemberg; Hubert Rehrauer; Isabel Stürmer; Joel Wirz; Jon Huder; Jürg Böni; Kevin Steiner; Maria Grünberg; Maryam Zaheri; Michael Huber; Riccarda Capaul; Stefan Schmutz; Verena Kufner; Weihong Qi
EPI_ISL_2115844	amedes MVZ Hannover	Robert Koch Institute	
EPI_ISL_829062	deCODE genetics	deCODE genetics	Agnar Helgason; Alma Moller; Arna B Agustsdottir; Arnaldur Gylfason; Asgeir Sigurdsson; Aslaug Jonasdottir; Berglind Eiriksdottir; Bjarni Thorbjornsson; Brynjar O Jensson; Daniel F Gudbjartsson; Droplaug N Magnusdottir; Elisabet E Gardarsdottir; Emil A Thorarensen; Gardar Sveinbjornsson; Gisli Masson; Gudmundur Georgsson; Gudmundur L Norddahl; Gudrun Sigmundsdottir; Hakon Jonsson; Hannes Eggertsson; Hilma Holm; Ingileif Jonsdottir; Jona Saemundsdottir; Kamilla S Josefsdottir; Kari Stefansson; Karl G Kristinsson; Kjartan R Gudmundsson; Kristin E Sveinsdottir; Louise le Roux; Maney Sveinsdottir; Olafia S Gretarsdottir; Olafur T Magnusson; Pall Melsted; Patrick Sulem; Run Fridriksdottir; Solvi Rognvaldsson; Thora R Gunnarsdottir; Thordur Kristjansson; Thorolfur Gudnason; Unnur Thorsteinsdottir
EPI_ISL_447637	unknown	Department of Medicine	Bampali, M.; Dovrolis, N.; Froukala, E.; Gatzidou, E.; Kassela, K.; N. and Karakasiliotis, I.; Spanakis; Stavropoulou, A.; Tsakris, A.; Veletza, S.
EPI_ISL_437614, EPI_ISL_437618, EPI_ISL_437624	unknown	Faculty of Medicine	Buathong, R.; Bunprakob, S.; Ghai, S.; Joyjinda, Y.; Mungaomklang, A.; Petcharat, S.; Plipat; Prasithsirikul, W.; Rodpan, A.; Sirichan, N.; T. and Hemachudha, T.; Wacharapluesadee, S.
EPI_ISL_507011	unknown	Infectious Diseases Research, King Abdullah International Medical Research Center (KAIMRC)	Alghoribi; M.F.
EPI_ISL_450412	unknown	Microbiology	K.-Y.; K.K.W.; To; Yuen
EPI_ISL_483060	unknown	Microbiology, Canterbury Health Laboratories	Anderson, T.; Dilcher, M.
EPI_ISL_3417525, EPI_ISL_3417526, EPI_ISL_3417527, EPI_ISL_3417528, EPI_ISL_3417529, EPI_ISL_3417530, EPI_ISL_3417531, EPI_ISL_3417532			
see above	unknown	PHV-FSS	Chenwei Wang on behalf of Q-PHIRE Genomics
